# Supplementary material for: The quest for a generic bird target to detect the presence of bird in food products and considerations for paleoprotein analysis
Source: PLoS One. 2022 Dec 20;17(12):e0279369. doi: 10.1371/journal.pone.0279369 (PMC9767367; doi:10.1371/journal.pone.0279369)
Supplement: S2 File — All relevant chromatograms and MS/MS spectra of the bird samples and negative control beef broth. (PDF) [file pone.0279369.s002.pdf]

S2 File. Chromatograms and MS/MS spectra (Fig S 001 to Fig S 111).

Raw data chromatograms (deamidated) VGPIGPAGNR

Fig S 001: ostrich tendon
Fig S 002: goose neck
Fig S 003: duck neck
Fig S 004: turkey neck
Fig S 005: chicken leg
Fig S 006: pheasant meat strip
Fig S 007: goose meat strip
Fig S 008: goose leg
Fig S 009: pheasant leg
Fig S 010: guinea fowl torso
Fig S 011: pigeon torso
Fig S 012: partridge torso
Fig S 013: duck leg
Fig S 014: quail leg
Fig S 015: turkey leg

Remarks:
-Extracted m/z range 469.75-469.77
-The retention time and m/z of the base peak are provided per peak and the provided intensity is of the highest peak
-Data recorded in June 2020

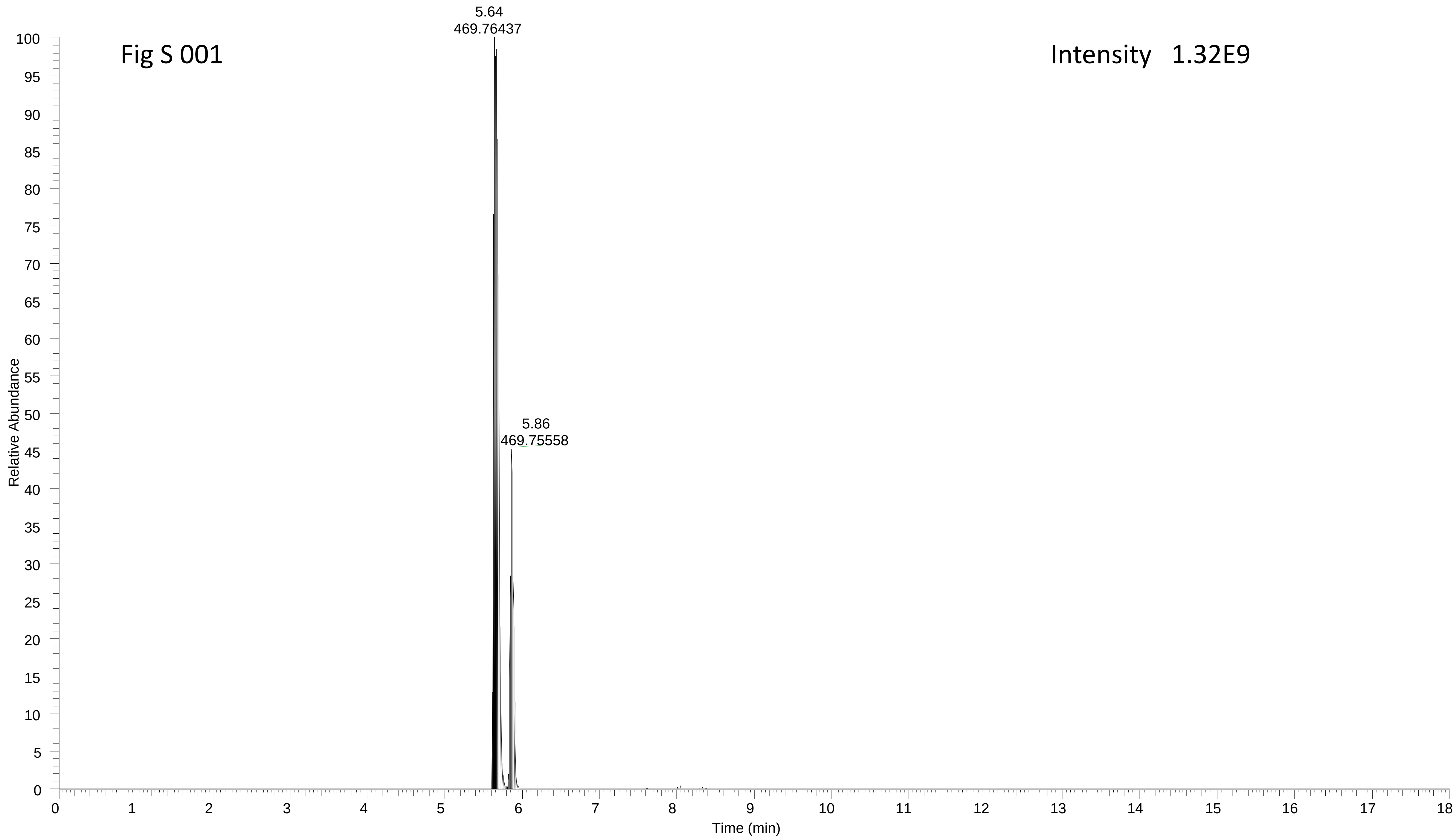
Fig S 001
Intensity 1.32E9
5.64
469.76437
5.86
469.75558
Relative Abundance
Time (min)
0 1 2 3 4 5 6 7 8 9 10 11 12 13 14 15 16 17 18
0 5 10 15 20 25 30 35 40 45 50 55 60 65 70 75 80 85 90 95 100

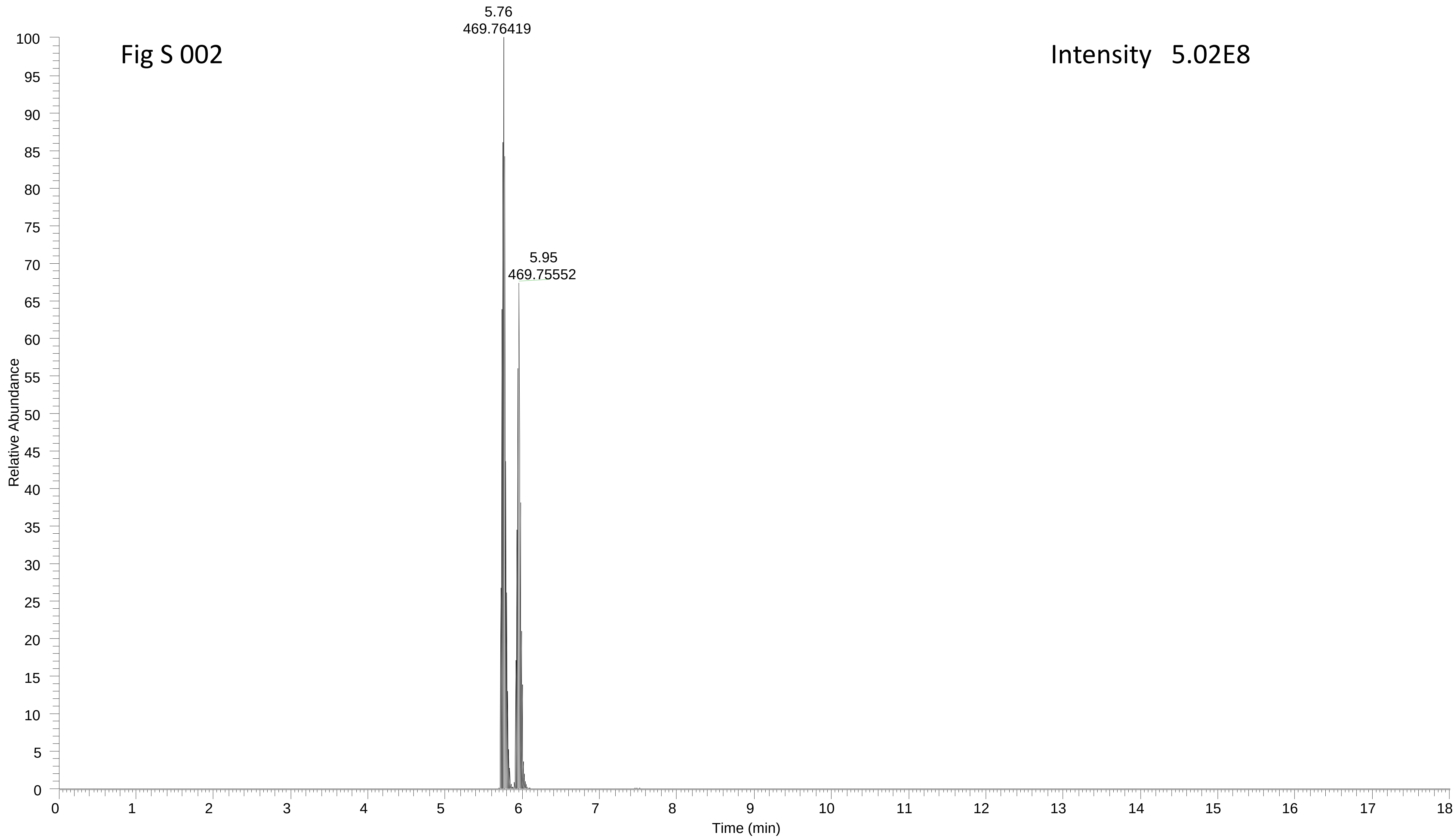
Fig S 002
Intensity 5.02E8
5.76
469.76419
5.95
469.75552
Relative Abundance
Time (min)
0
5
10
15
20
25
30
35
40
45
50
55
60
65
70
75
80
85
90
95
100
0
1
2
3
4
5
6
7
8
9
10
11
12
13
14
15
16
17
18

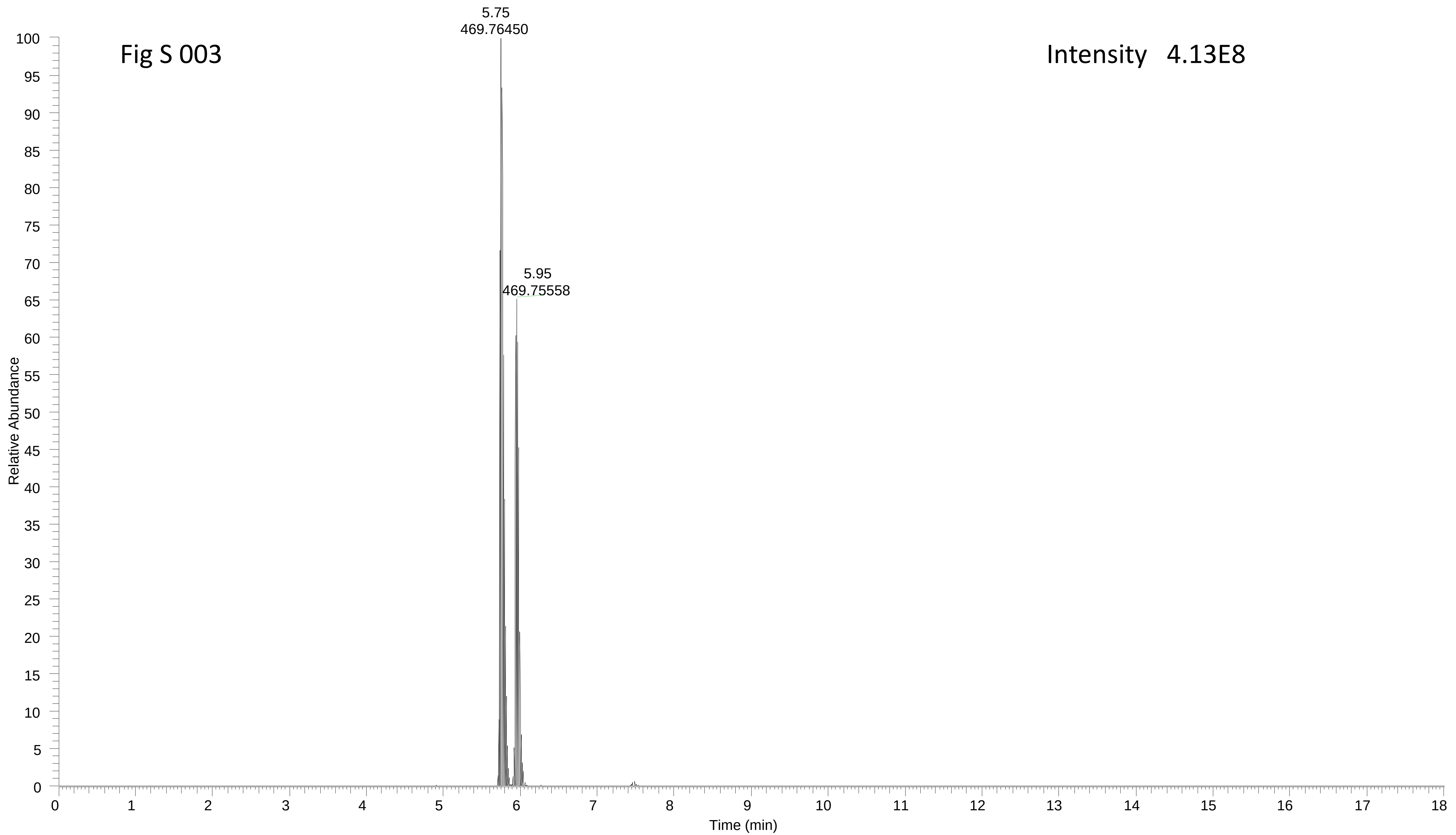
Fig S 003
Intensity 4.13E8
5.75
469.76450
5.95
469.75558
Relative Abundance
Time (min)
0
5
10
15
20
25
30
35
40
45
50
55
60
65
70
75
80
85
90
95
100
1
2
3
4
6
7
8
9
11
12
13
14
16
17
18

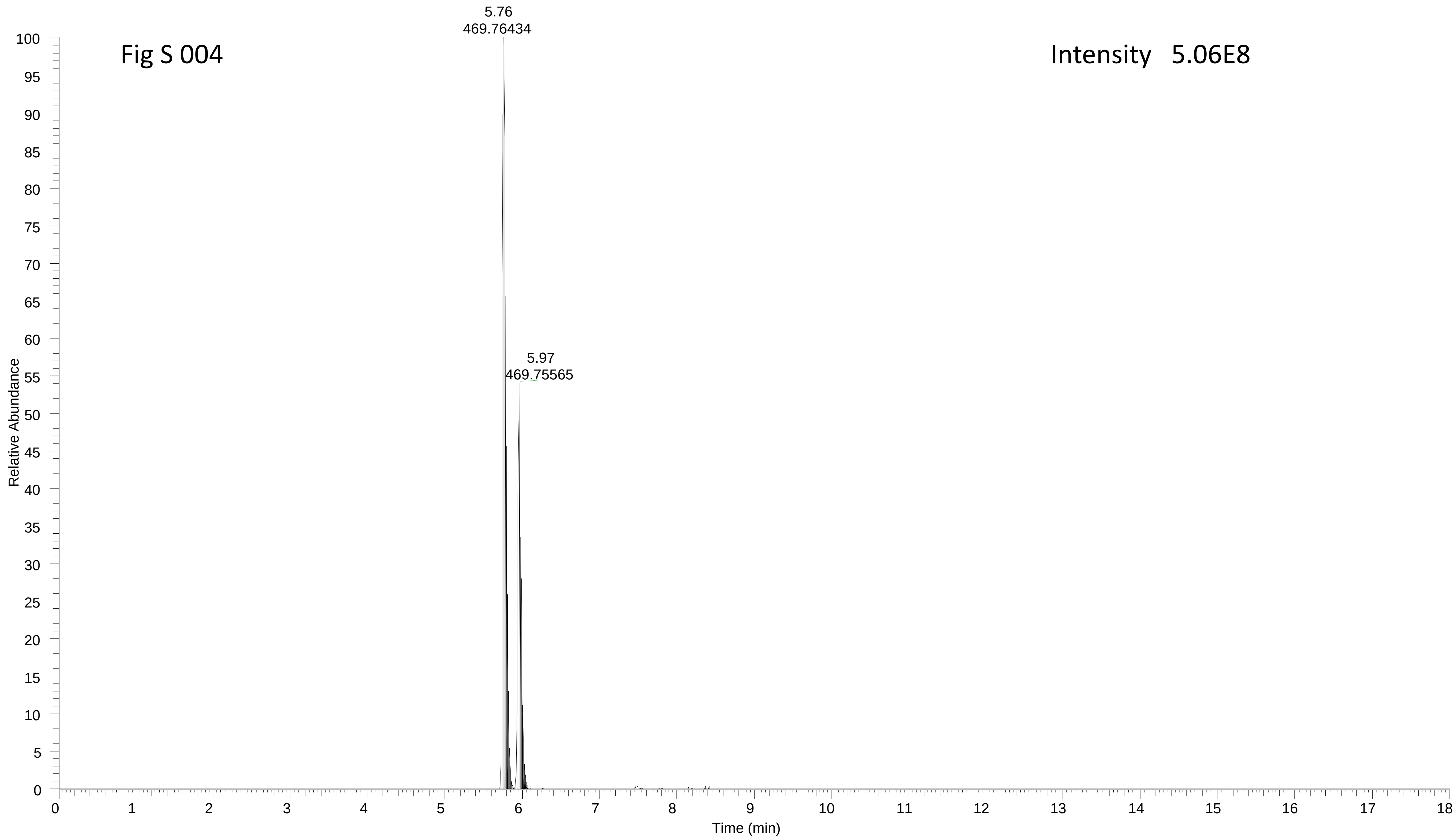
Fig S 004
Intensity 5.06E8
5.76
469.76434
5.97
469.75565
Relative Abundance
Time (min)
0
5
10
15
20
25
30
35
40
45
50
55
60
65
70
75
80
85
90
95
100
1
2
3
4
6
7
8
9
11
12
13
14
16
17
18

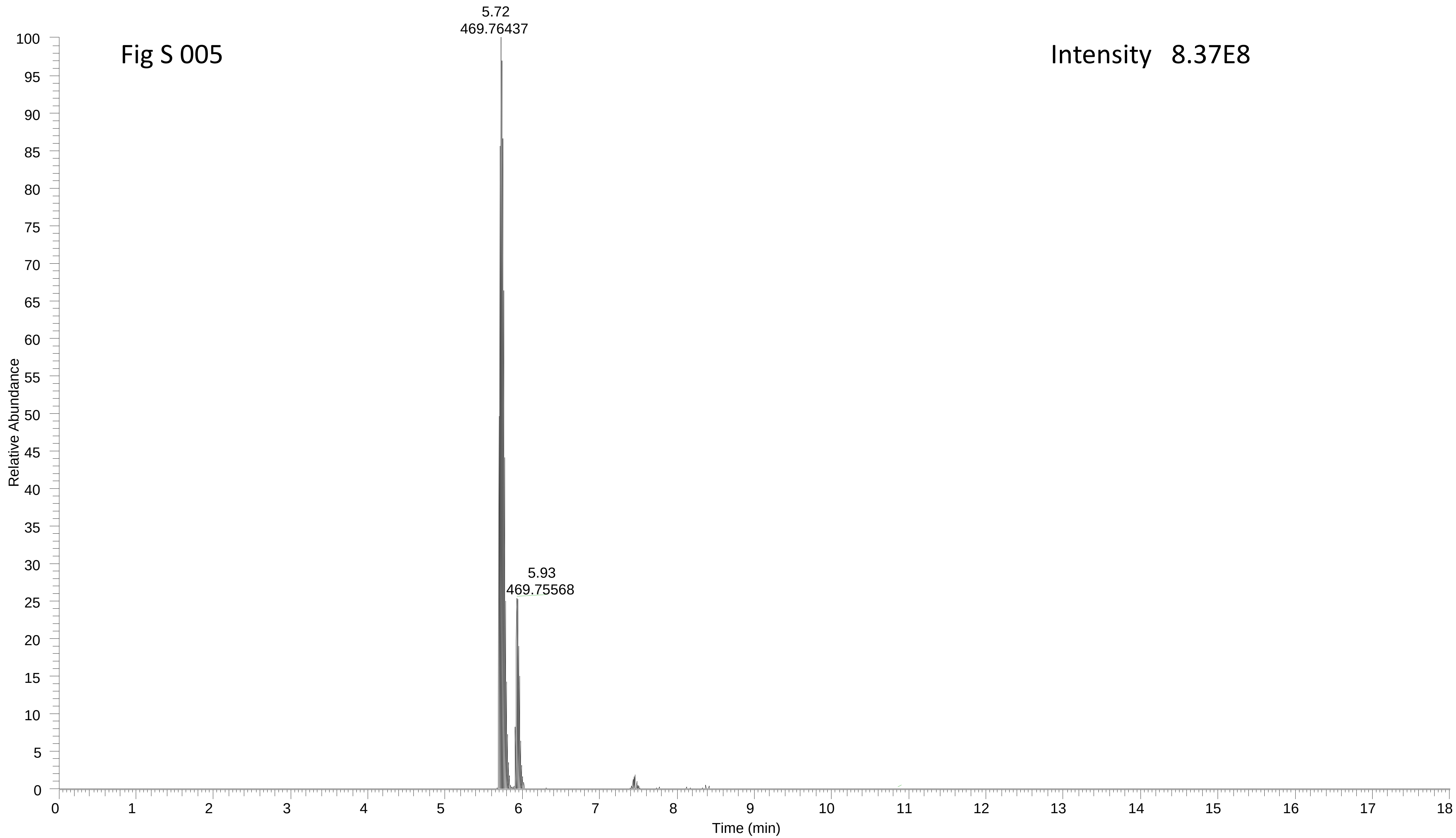
Fig S 005
Intensity 8.37E8
5.72
469.76437
5.93
469.75568
Relative Abundance
Time (min)

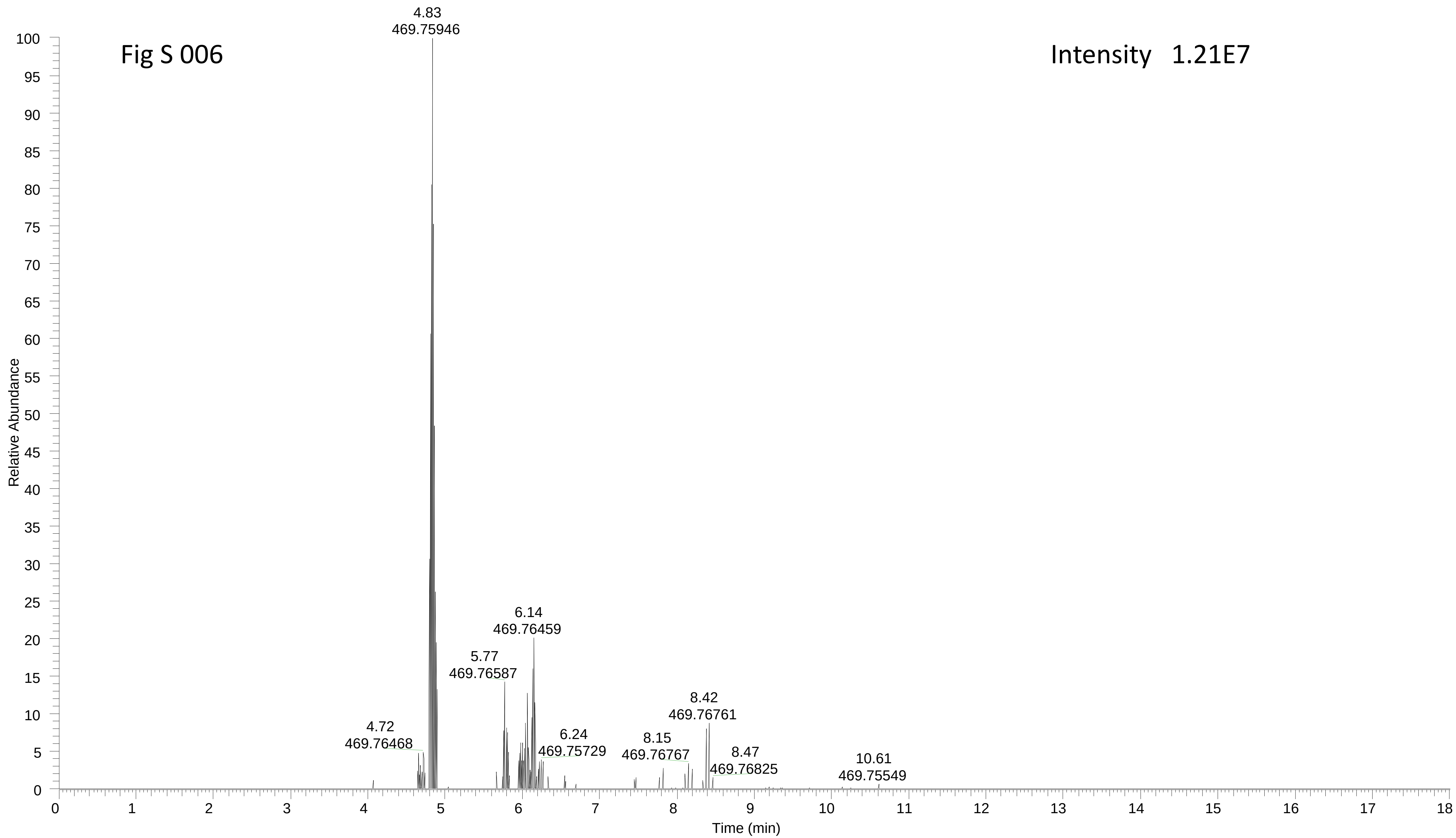

Fig S 006
Intensity 1.21E7
4.83
469.75946
4.72
469.76468
5.77
469.76587
6.14
469.76459
6.24
469.75729
8.15
469.76767
8.42
469.76761
8.47
469.76825
10.61
469.75549
Relative Abundance
Time (min)

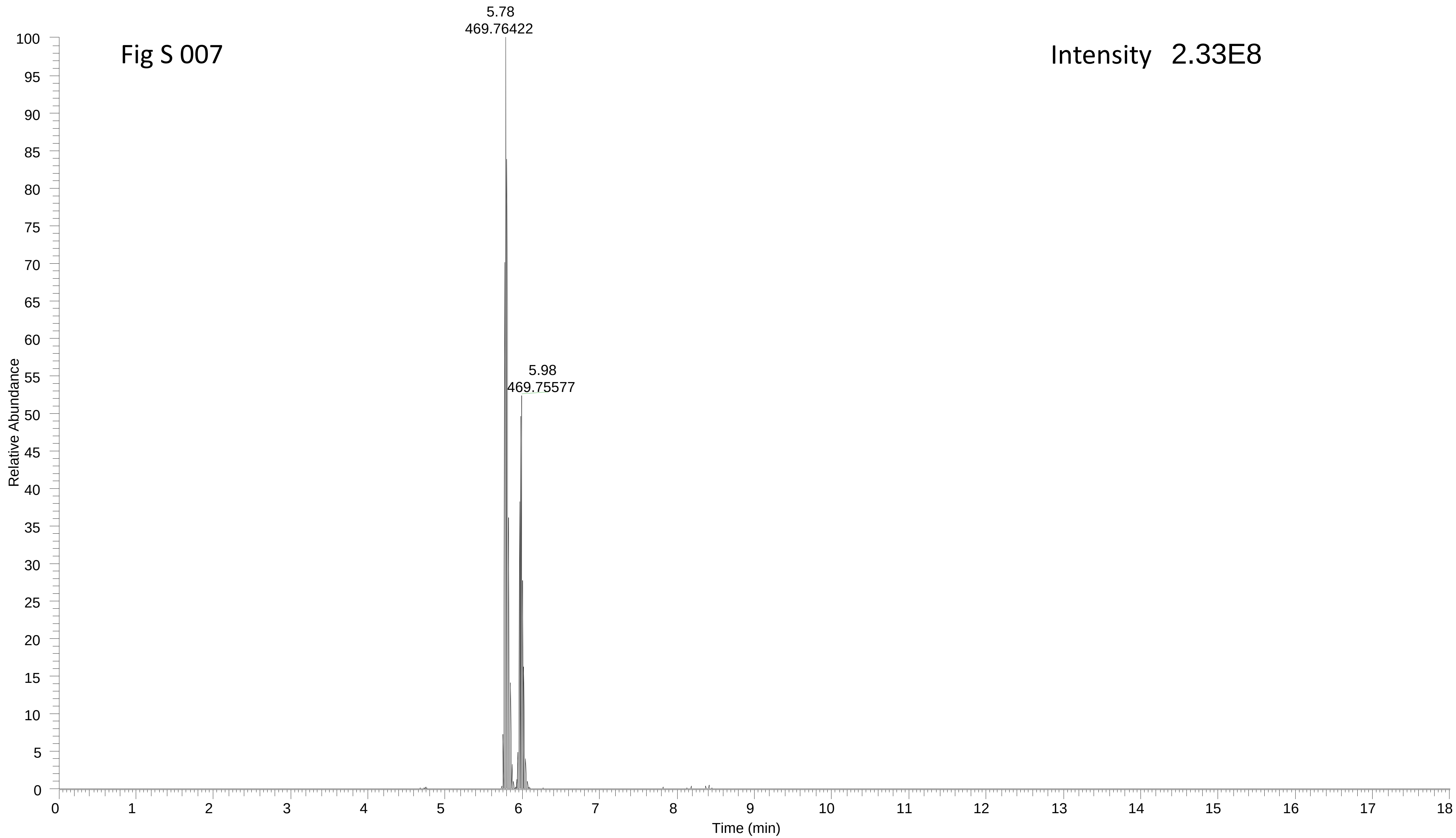
Fig S 007
Intensity 2.33E8
5.78
469.76422
5.98
469.75577
Relative Abundance
Time (min)
0
5
10
15
20
25
30
35
40
45
50
55
60
65
70
75
80
85
90
95
100
0
1
2
3
4
5
6
7
8
9
10
11
12
13
14
15
16
17
18

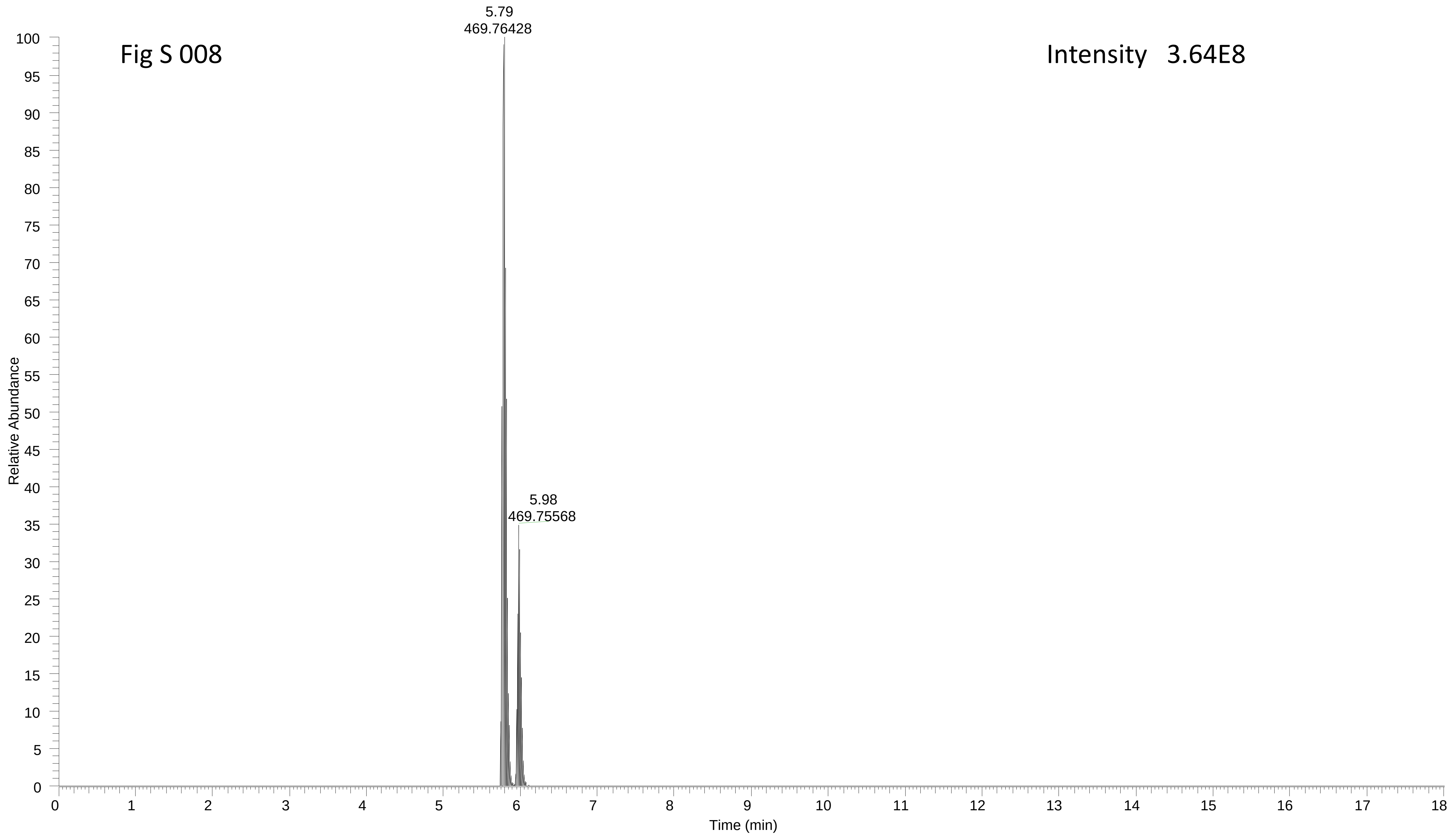
Fig S 008
Intensity 3.64E8
5.79
469.76428
5.98
469.75568
Relative Abundance
Time (min)
0
5
10
15
20
25
30
35
40
45
50
55
60
65
70
75
80
85
90
95
100
0
1
2
3
4
5
6
7
8
9
10
11
12
13
14
15
16
17
18

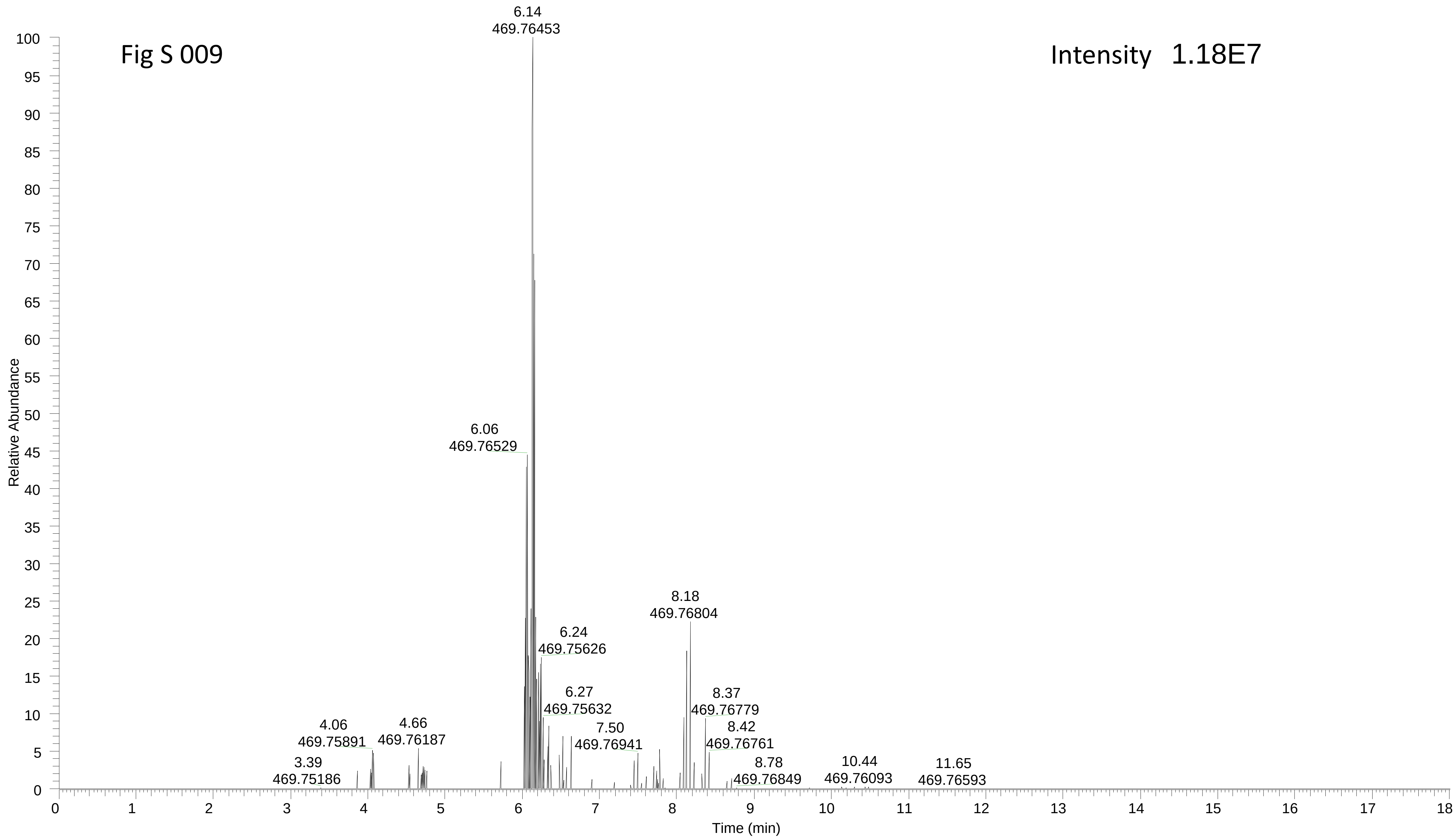
Fig S 009
Intensity 1.18E7
6.14
469.76453
6.06
469.76529
6.24
469.75626
6.27
469.75632
7.50
469.76941
8.18
469.76804
8.37
469.76779
8.42
469.76761
8.78
469.76849
10.44
469.76093
11.65
469.76593
4.06
469.75891
4.66
469.76187
3.39
469.75186
Relative Abundance
Time (min)
0
5
10
15
20
25
30
35
40
45
50
55
60
65
70
75
80
85
90
95
100
0
1
2
3
4
5
6
7
8
9
10
11
12
13
14
15
16
17
18

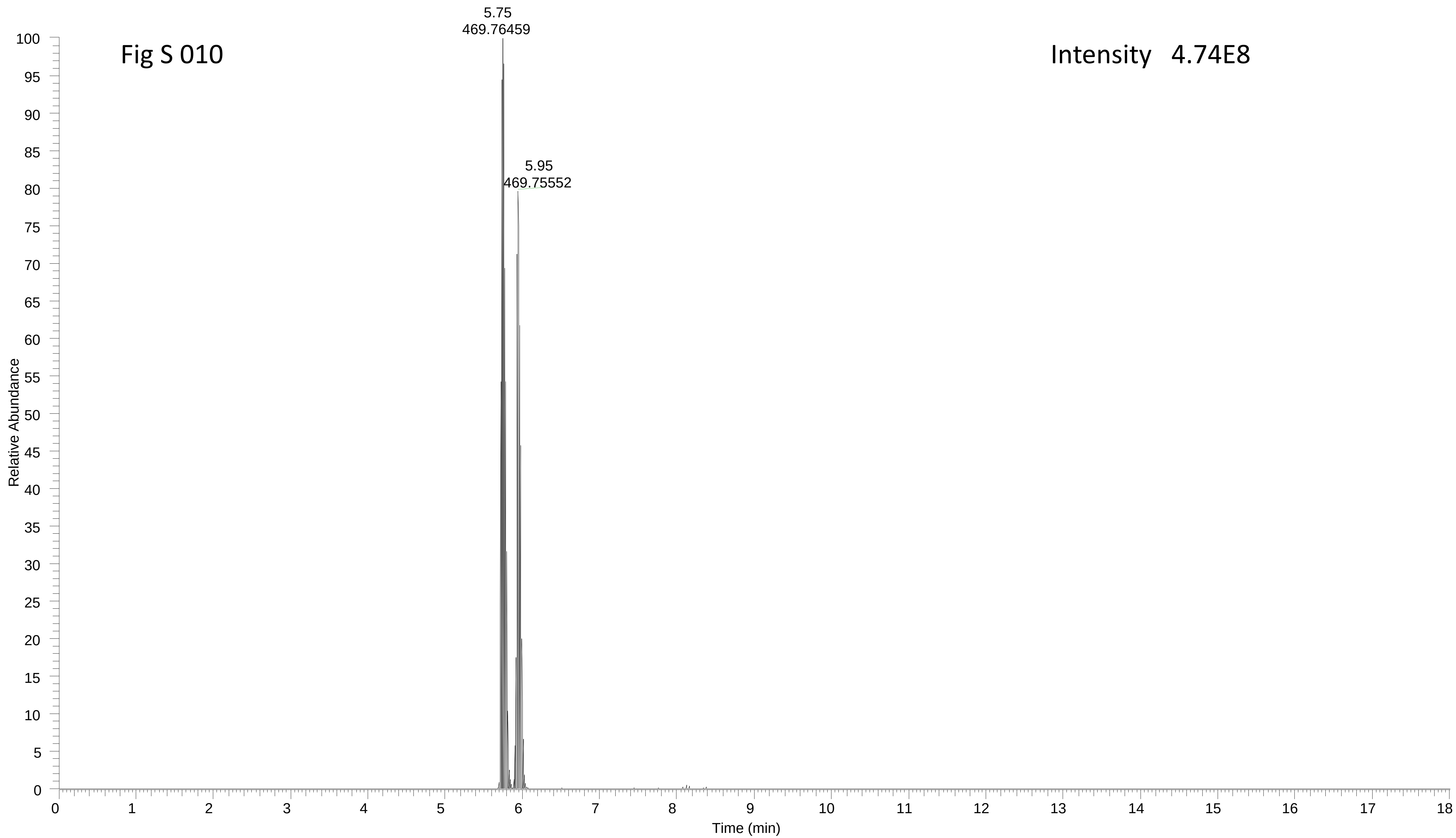
Fig S 010
Intensity 4.74E8
5.75
469.76459
5.95
469.75552
Relative Abundance
Time (min)
0
5
10
15
20
25
30
35
40
45
50
55
60
65
70
75
80
85
90
95
100
0
1
2
3
4
5
6
7
8
9
10
11
12
13
14
15
16
17
18

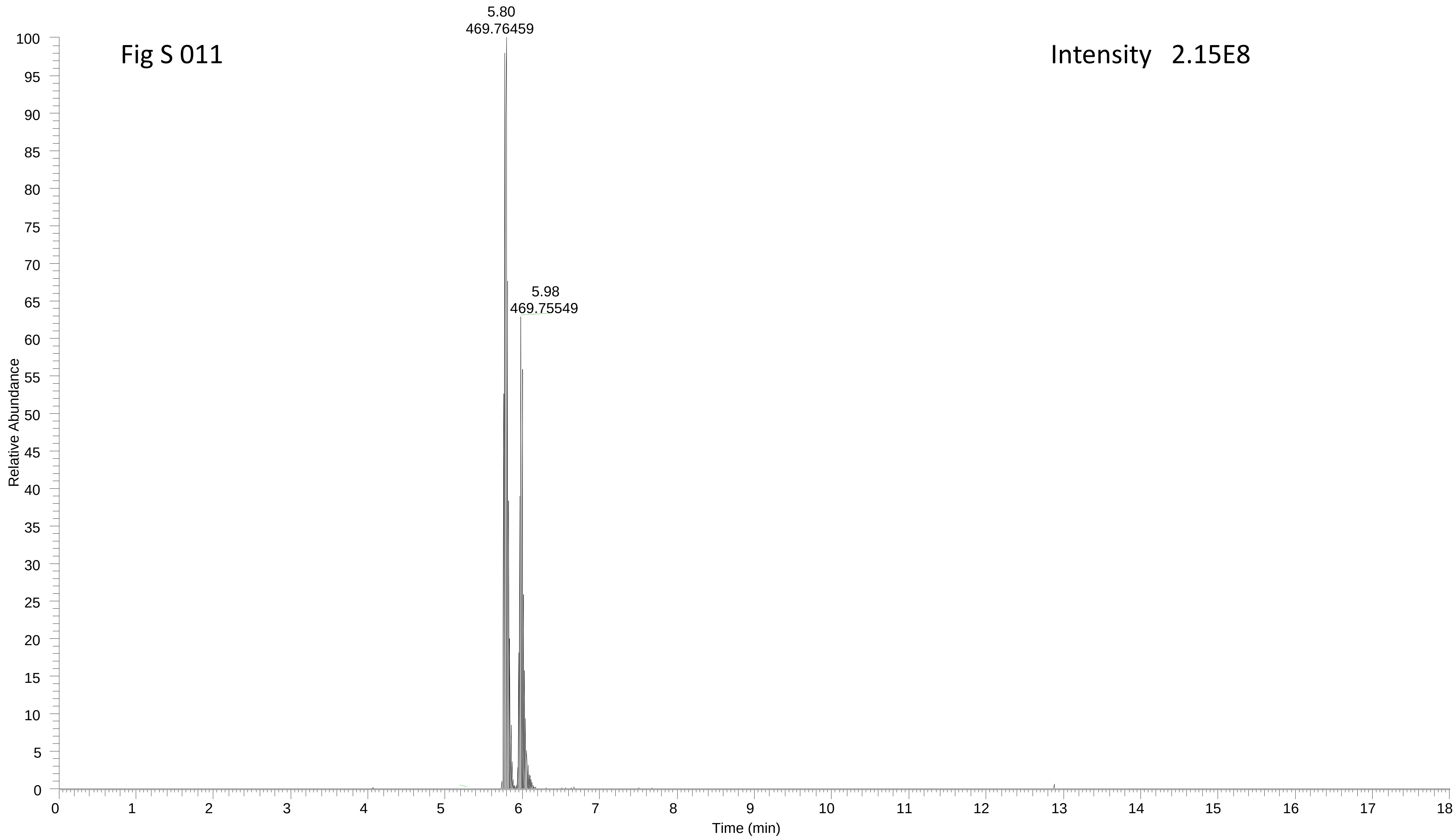
Fig S 011
Intensity 2.15E8
5.80
469.76459
5.98
469.75549
Relative Abundance
Time (min)
0
5
10
15
20
25
30
35
40
45
50
55
60
65
70
75
80
85
90
95
100
1
2
3
4
6
7
8
9
11
12
13
14
16
17
18

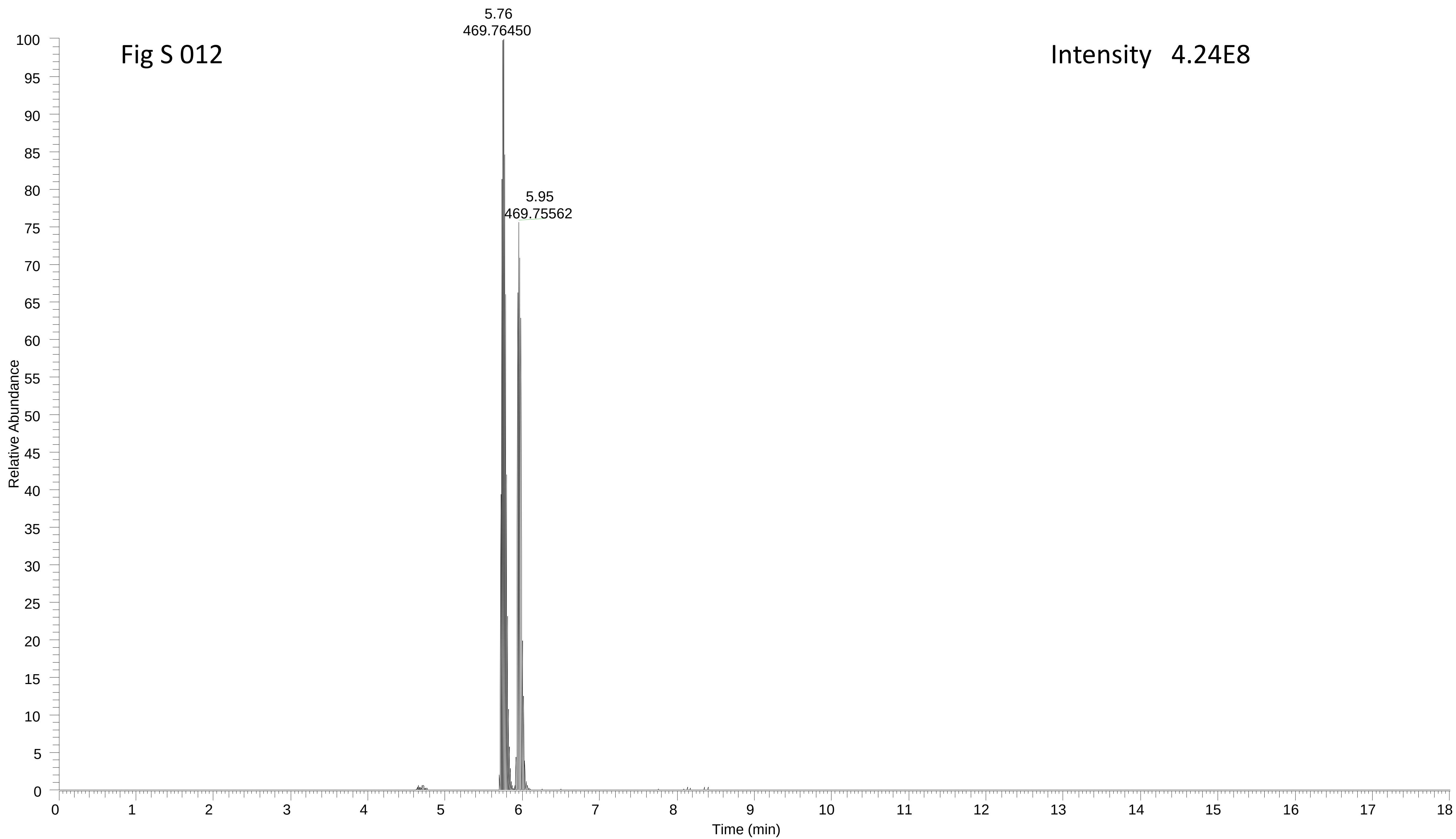
Fig S 012
Intensity 4.24E8
5.76
469.76450
5.95
469.75562
Relative Abundance
Time (min)
0
5
10
15
20
25
30
35
40
45
50
55
60
65
70
75
80
85
90
95
100
0
1
2
3
4
5
6
7
8
9
10
11
12
13
14
15
16
17
18

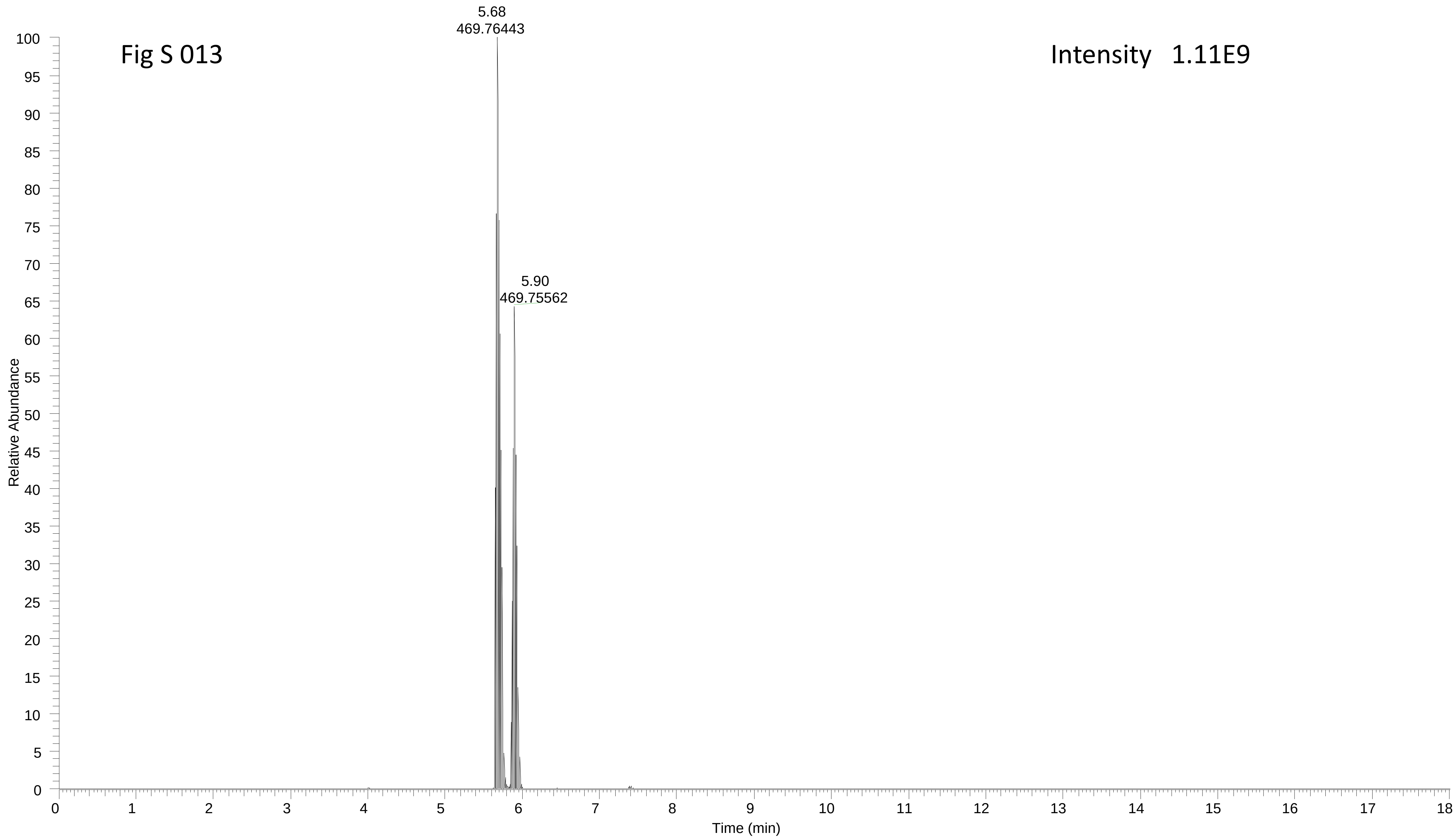
Fig S 013
Intensity 1.11E9
5.68
469.76443
5.90
469.75562
Relative Abundance
Time (min)
0
5
10
15
20
25
30
35
40
45
50
55
60
65
70
75
80
85
90
95
100
0
1
2
3
4
5
6
7
8
9
10
11
12
13
14
15
16
17
18

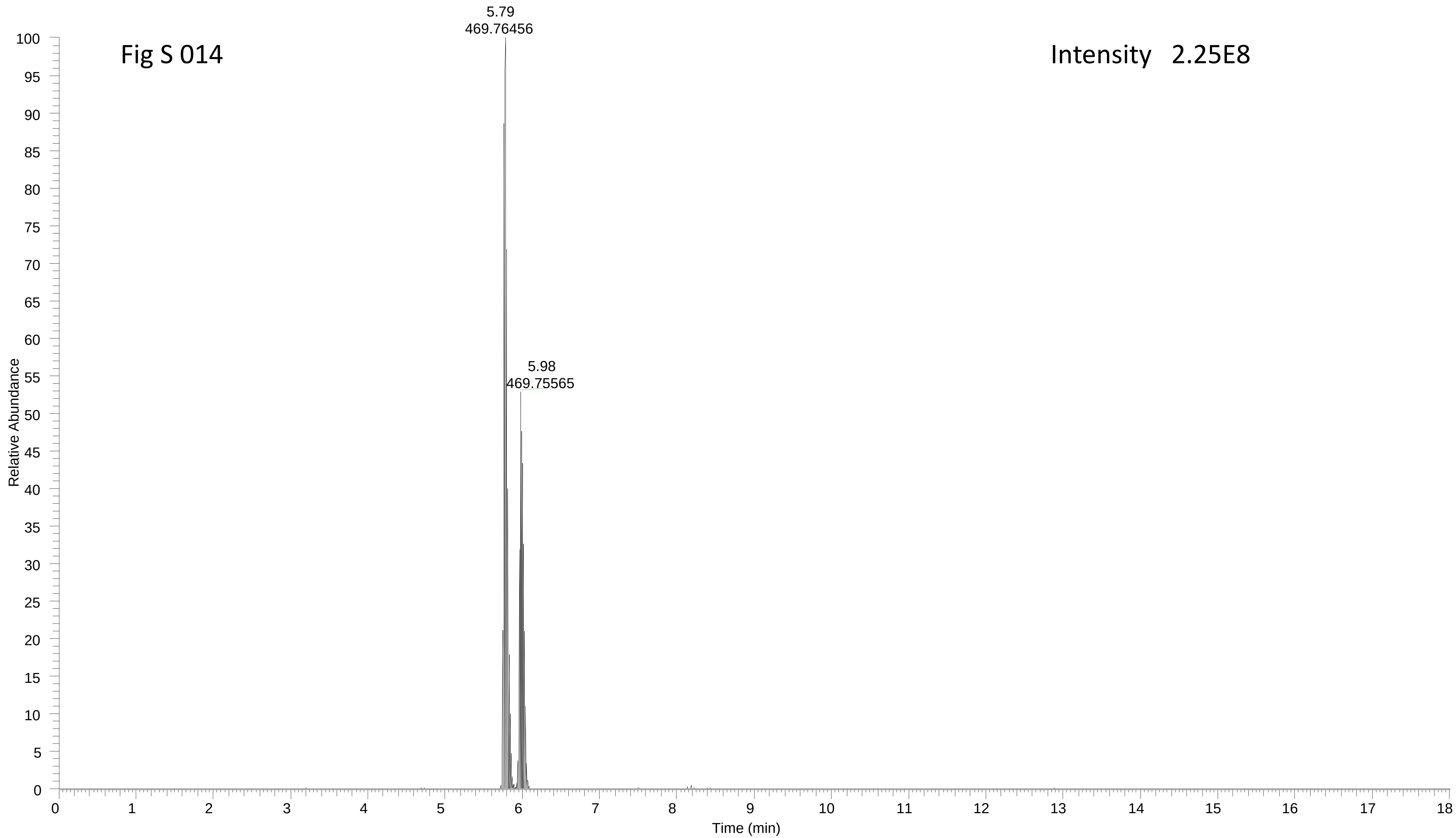

Fig S 014
Intensity 2.25E8
5.79
469.76456
5.98
469.75565
Relative Abundance
Time (min)

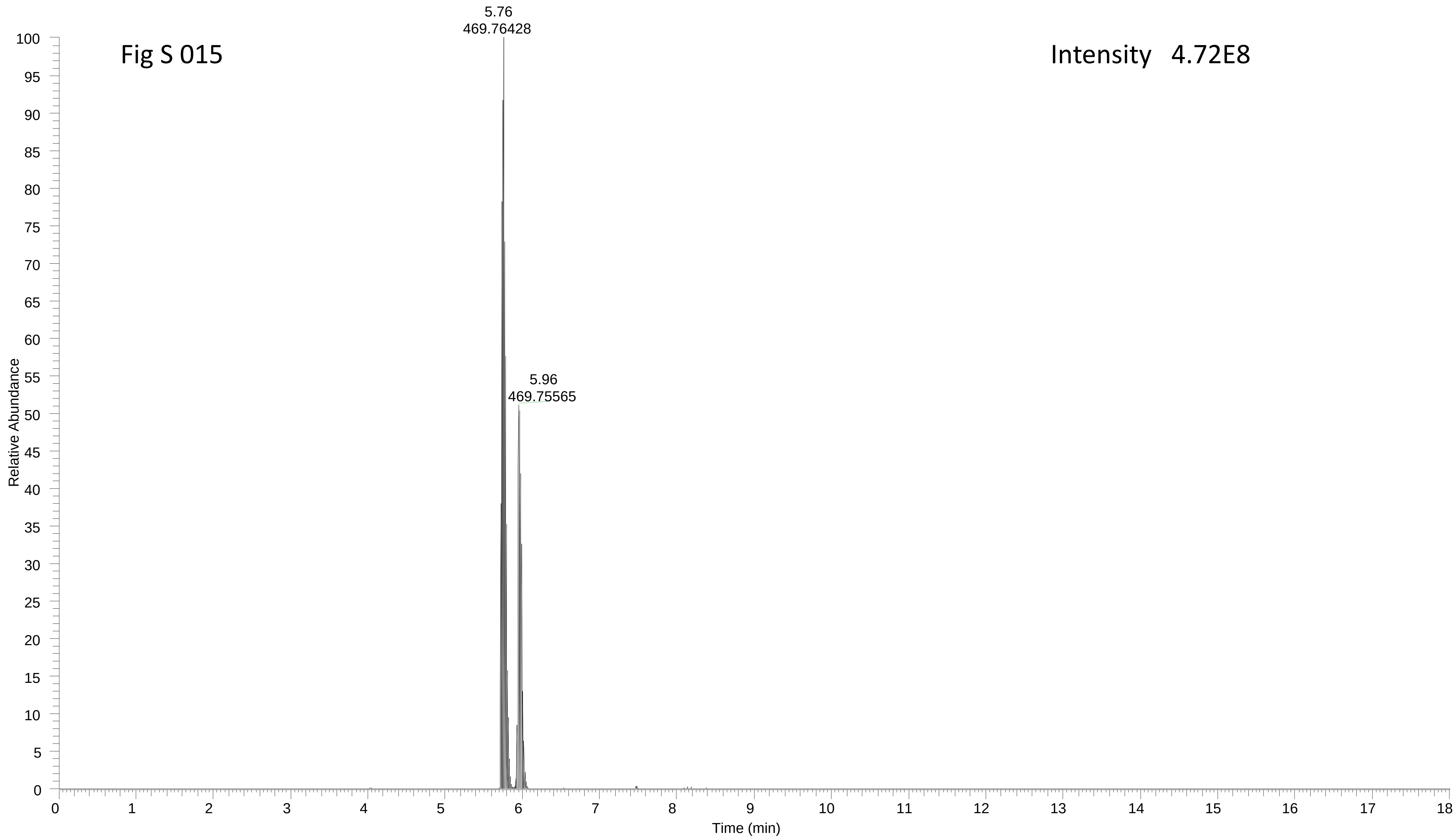
Fig S 015
Intensity 4.72E8
5.76
469.76428
5.96
469.75565
Relative Abundance
Time (min)
0
5
10
15
20
25
30
35
40
45
50
55
60
65
70
75
80
85
90
95
100
0
1
2
3
4
5
6
7
8
9
10
11
12
13
14
15
16
17
18

S2 File. Chromatograms and MS/MS spectra.

Raw data MS/MS spectra VGPIGPAGNR

Fig S 016: ostrich tendon
Fig S 017: goose neck
Fig S 018: duck neck
Fig S 019: turkey neck
Fig S 020: chicken leg
Fig S 021: goose meat strip
Fig S 022: goose leg
Fig S 023: guinea fowl torso
Fig S 024: pigeon torso
Fig S 025: partridge torso
Fig S 026: duck leg
Fig S 027: quail leg
Fig S 028: turkey leg

Remarks:
-Precursor m/z ≈ 469.26
-Pheasant samples did not provide MS/MS spectra for this peptide.
-Data recorded in June 2020.
-See Fig 4a in the main document for peak annotation.

200612_104 #3310 RT: 5.79 AV: 1 NL: 1.23E6
F: FTMS + p ESI d Full ms2 469.2631@hcd25.00 [65.0000-975.0000]

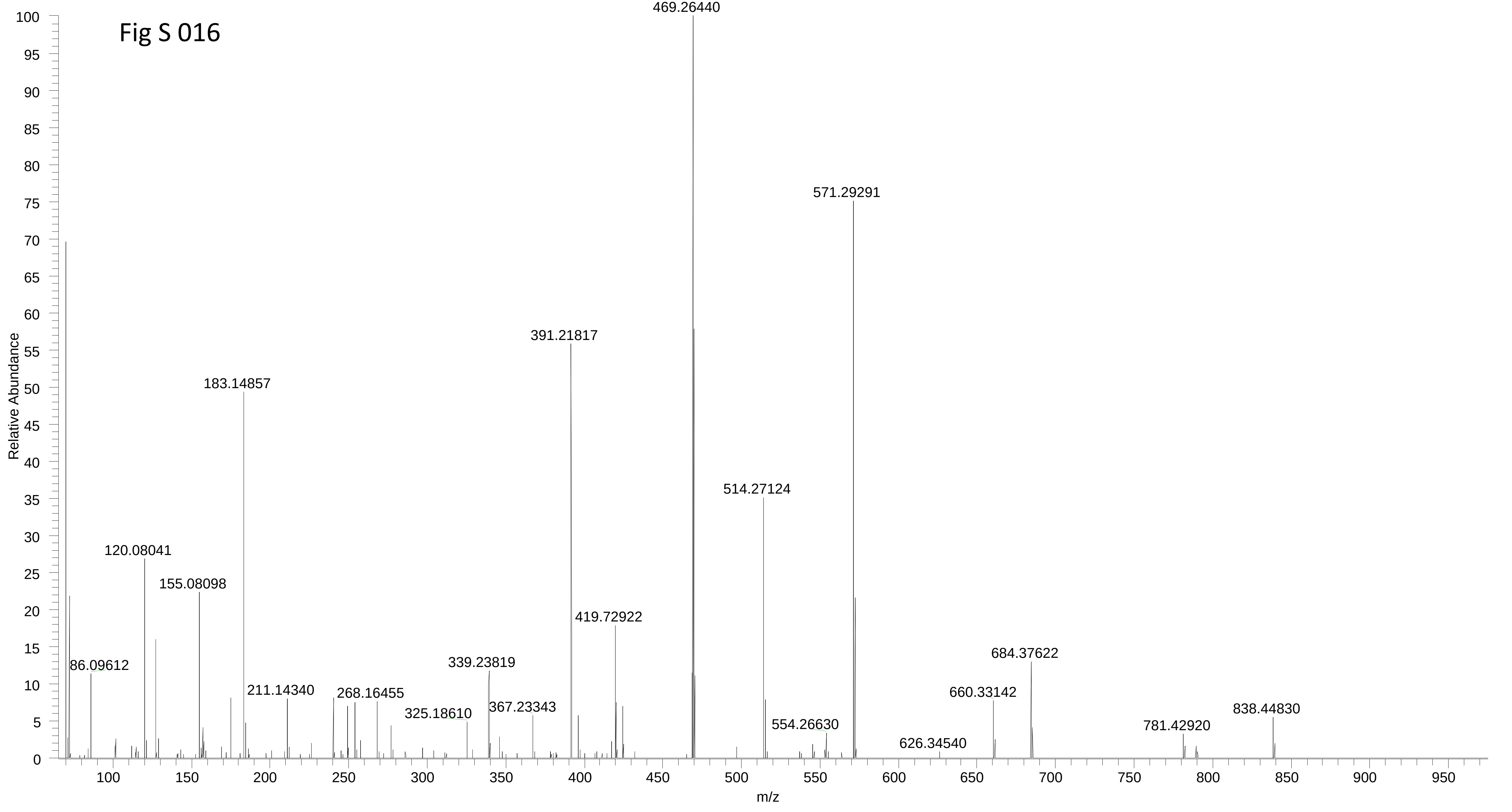

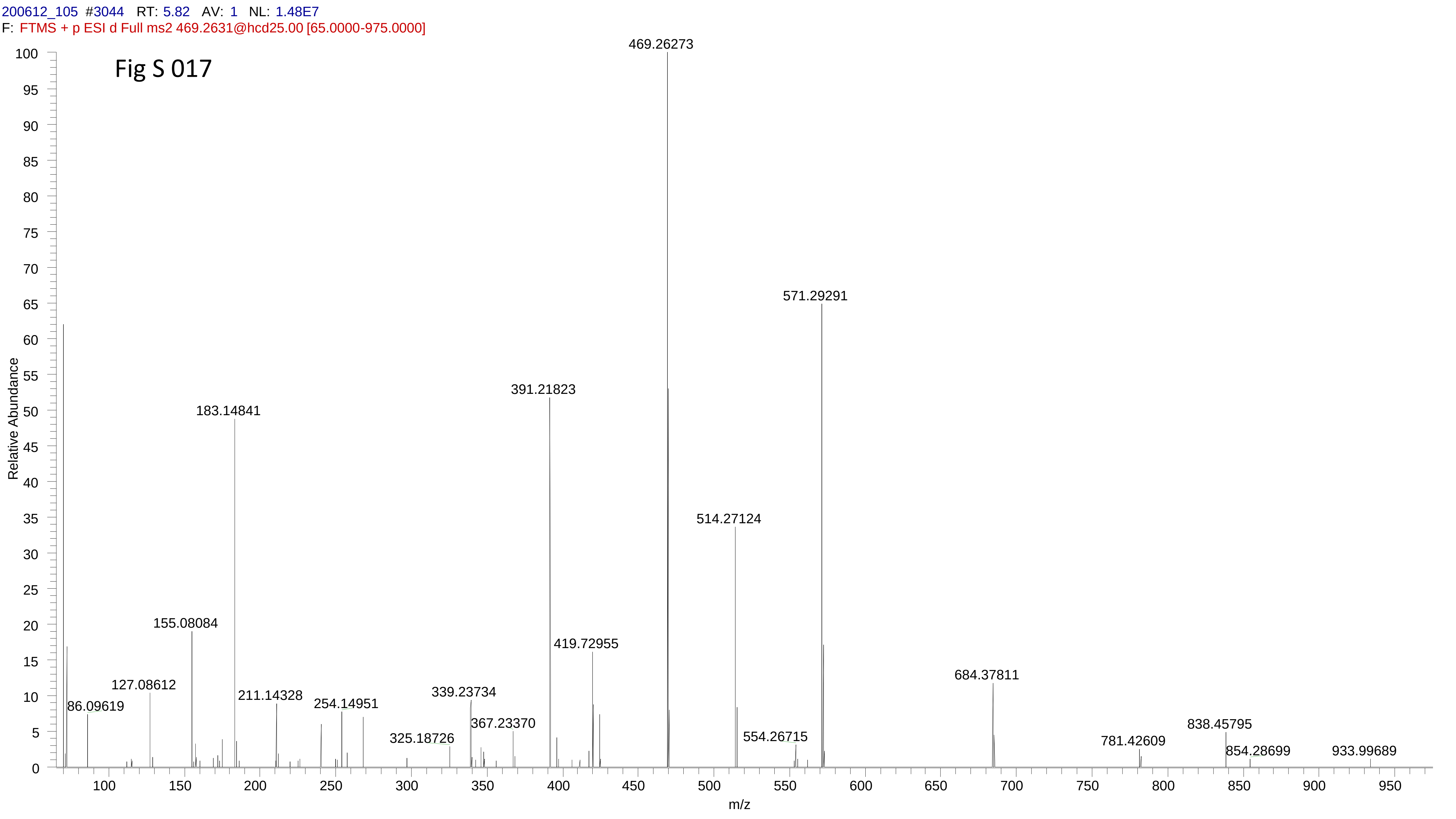

200612_105 #3044 RT: 5.82 AV: 1 NL: 1.48E7
F: FTMS + p ESI d Full ms2 469.2631@hcd25.00 [65.0000-975.0000]
Fig S 017
Relative Abundance
m/z
469.26273
571.29291
391.21823
183.14841
514.27124
155.08084
419.72955
684.37811
127.08612
86.09619
211.14328
254.14951
339.23734
367.23370
325.18726
554.26715
838.45795
781.42609
854.28699
933.99689

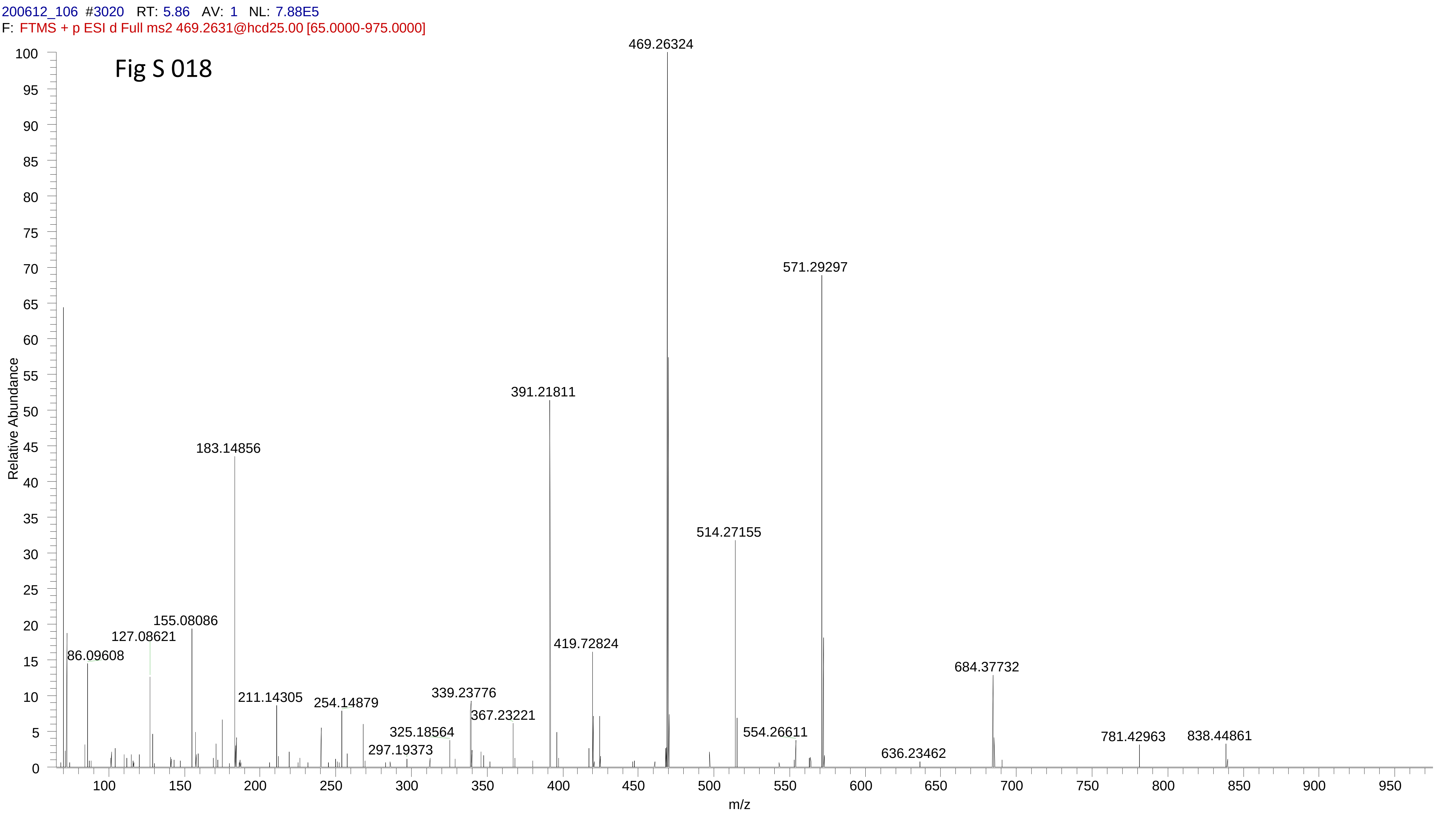
200612_106 #3020 RT: 5.86 AV: 1 NL: 7.88E5
F: FTMS + p ESI d Full ms2 469.2631@hcd25.00 [65.0000-975.0000]
Fig S 018
Relative Abundance
m/z
469.26324
571.29297
391.21811
183.14856
514.27155
155.08086
127.08621
86.09608
419.72824
684.37732
339.23776
211.14305
254.14879
367.23221
325.18564
554.26611
297.19373
781.42963
838.44861
636.23462

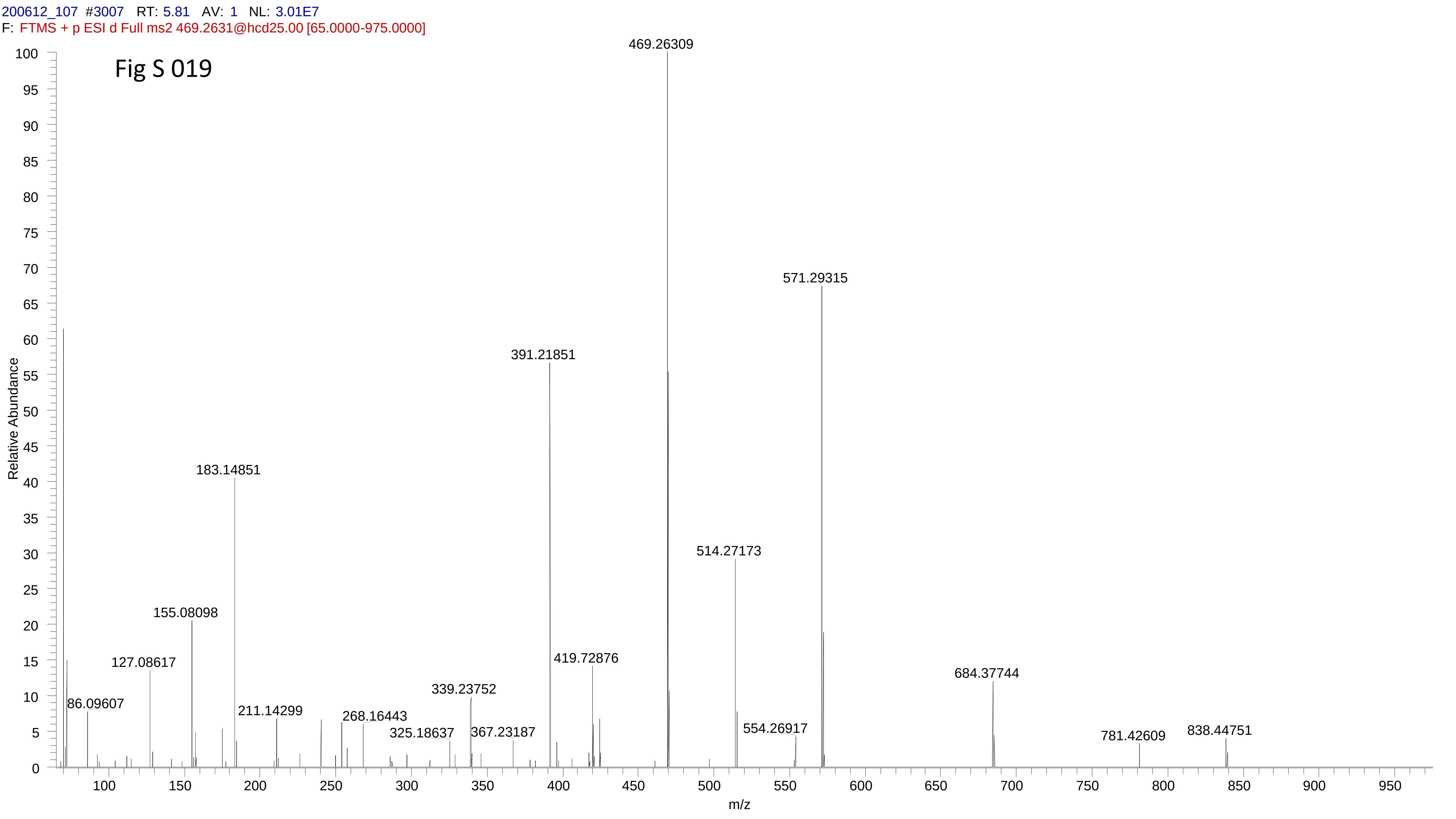
200612_107 #3007 RT: 5.81 AV: 1 NL: 3.01E7
F: FTMS + p ESI d Full ms2 469.2631@hcd25.00 [65.0000-975.0000]
Fig S 019
Relative Abundance
m/z
469.26309
571.29315
391.21851
183.14851
514.27173
155.08098
127.08617
419.72876
684.37744
339.23752
86.09607
211.14299
268.16443
325.18637
367.23187
554.26917
781.42609
838.44751

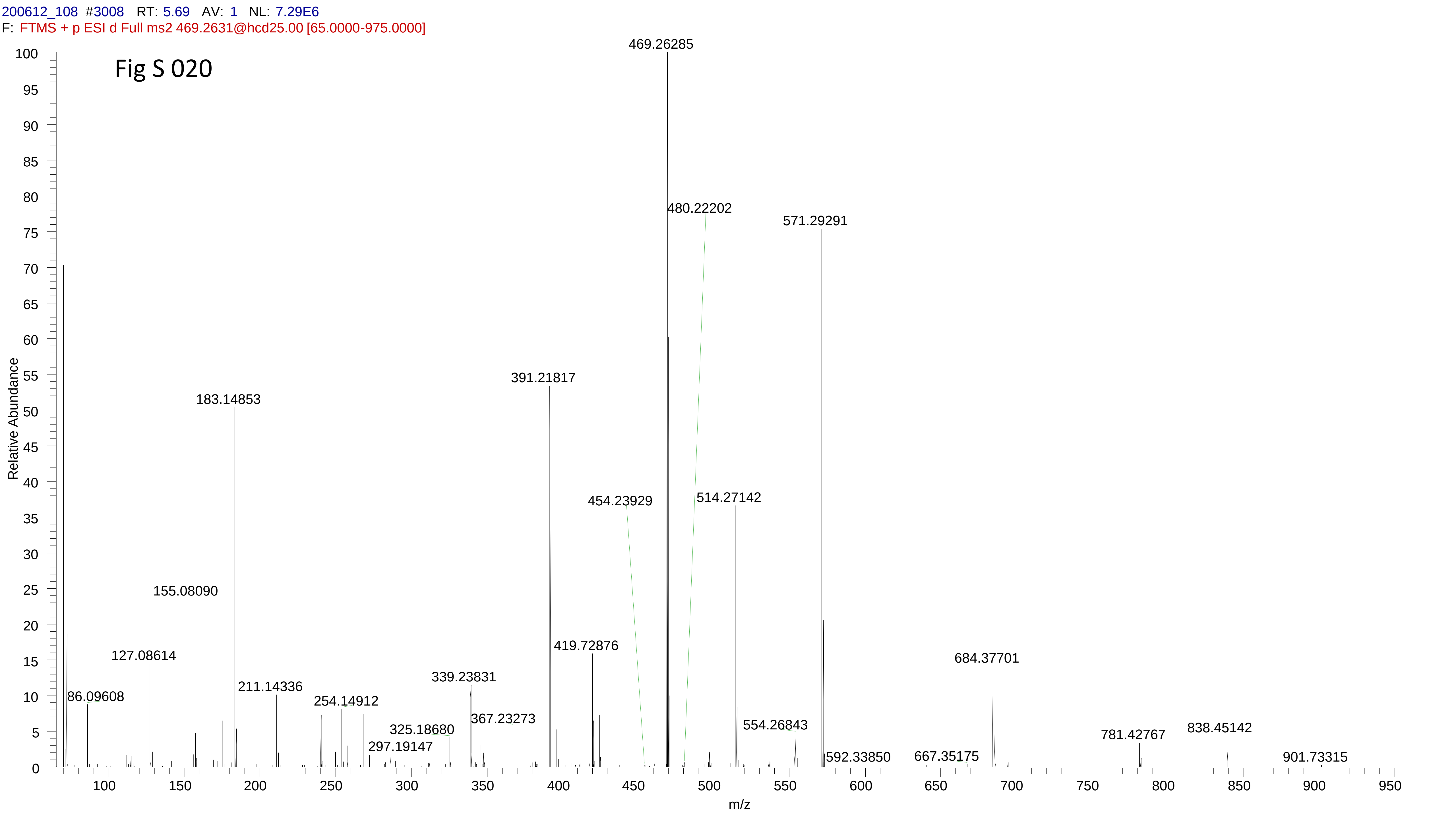
200612_108 #3008 RT: 5.69 AV: 1 NL: 7.29E6
F: FTMS + p ESI d Full ms2 469.2631@hcd25.00 [65.0000-975.0000]
Fig S 020
469.26285
480.22202
571.29291
391.21817
183.14853
454.23929
514.27142
155.08090
419.72876
127.08614
684.37701
339.23831
211.14336
86.09608
254.14912
367.23273
325.18680
554.26843
297.19147
781.42767
838.45142
592.33850
667.35175
901.73315
Relative Abundance
m/z

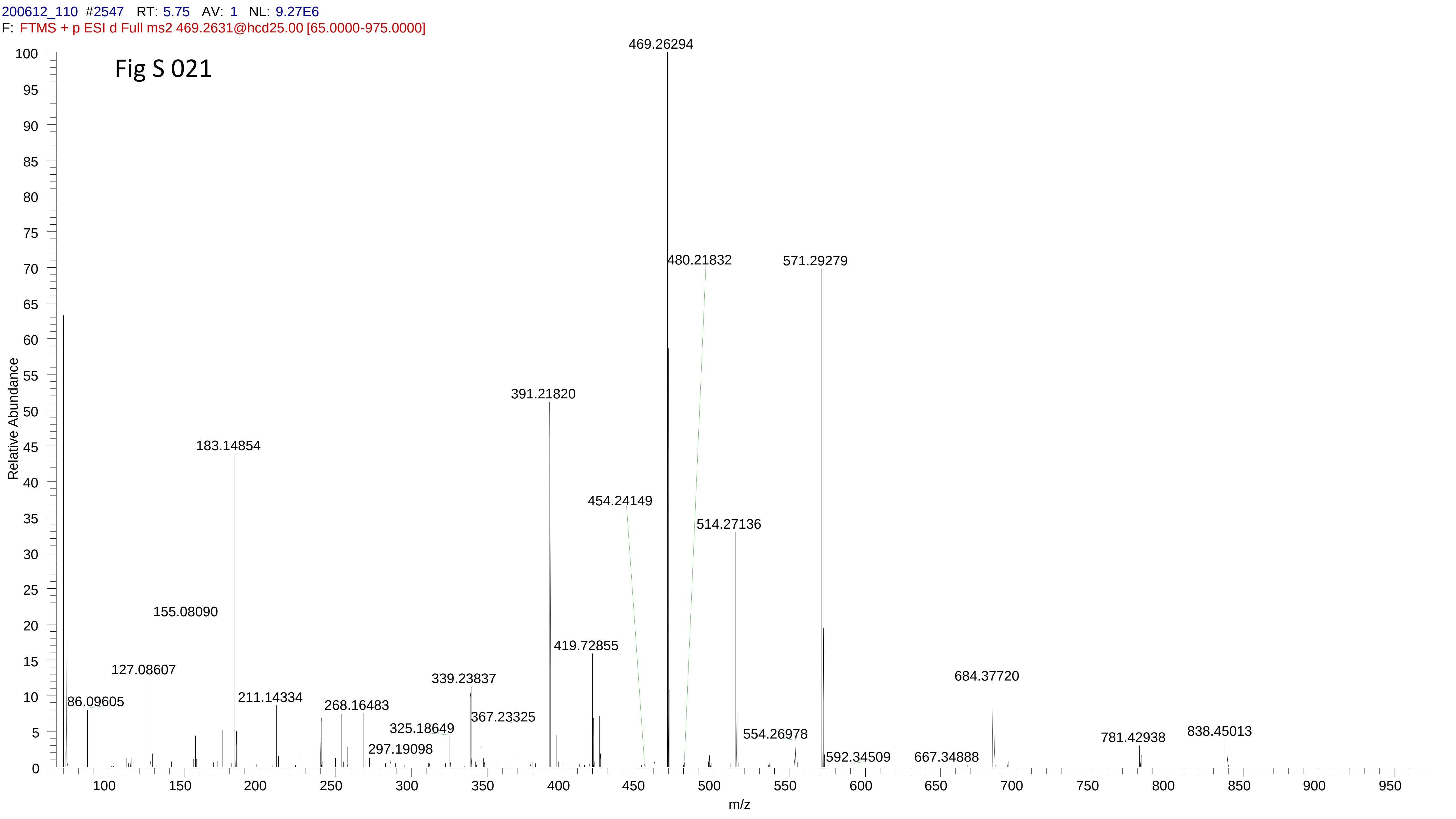
200612_110 #2547 RT: 5.75 AV: 1 NL: 9.27E6
F: FTMS + p ESI d Full ms2 469.2631@hcd25.00 [65.0000-975.0000]
Fig S 021
Relative Abundance
m/z
469.26294
480.21832
571.29279
391.21820
454.24149
514.27136
183.14854
155.08090
419.72855
127.08607
339.23837
684.37720
86.09605
211.14334
268.16483
367.23325
325.18649
554.26978
297.19098
781.42938
838.45013
592.34509
667.34888

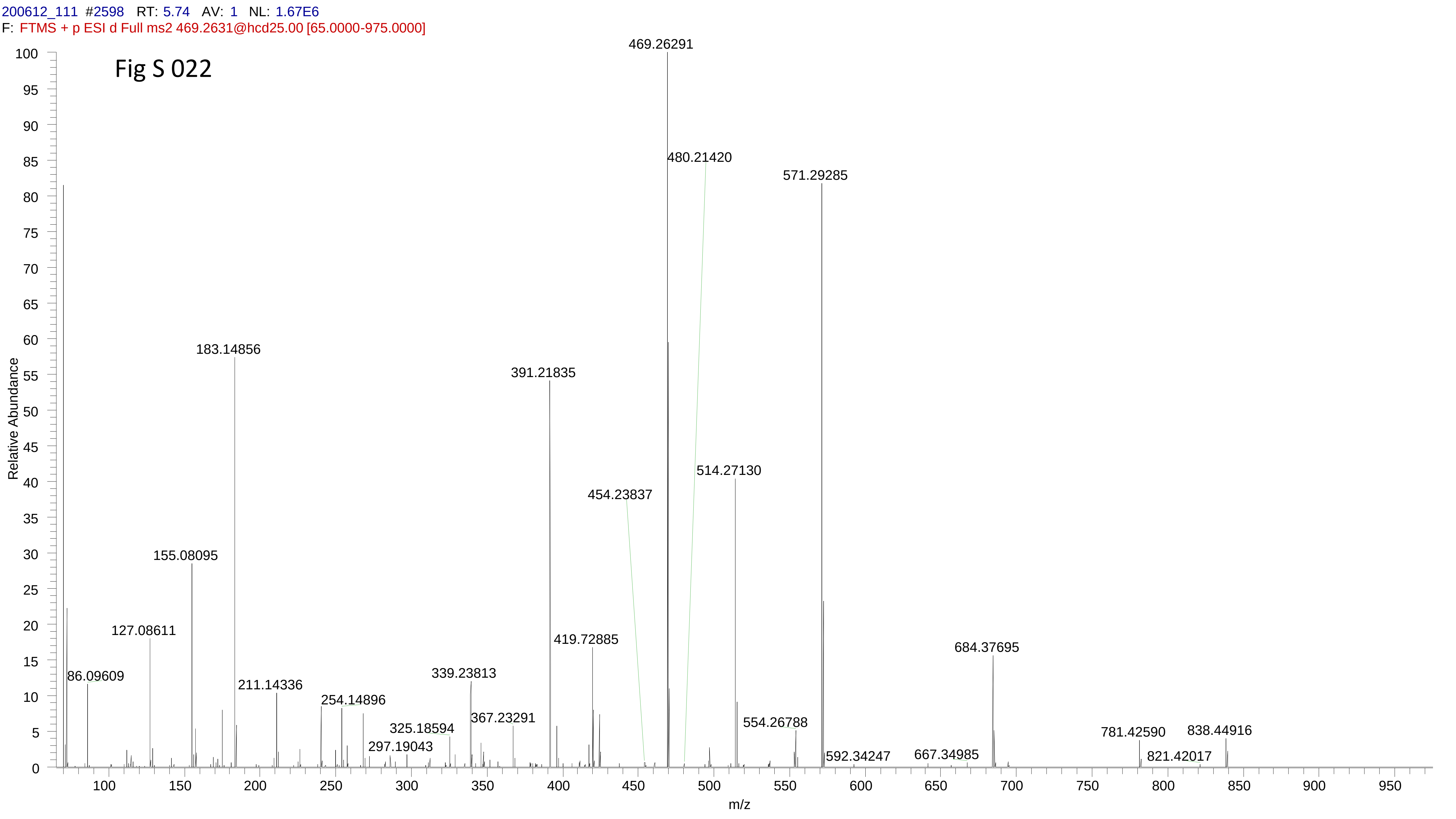
200612_111 #2598 RT: 5.74 AV: 1 NL: 1.67E6
F: FTMS + p ESI d Full ms2 469.2631@hcd25.00 [65.0000-975.0000]
Fig S 022
Relative Abundance
m/z
469.26291
480.21420
571.29285
183.14856
391.21835
514.27130
454.23837
155.08095
127.08611
419.72885
684.37695
86.09609
339.23813
211.14336
254.14896
367.23291
325.18594
554.26788
297.19043
781.42590
838.44916
592.34247
667.34985
821.42017

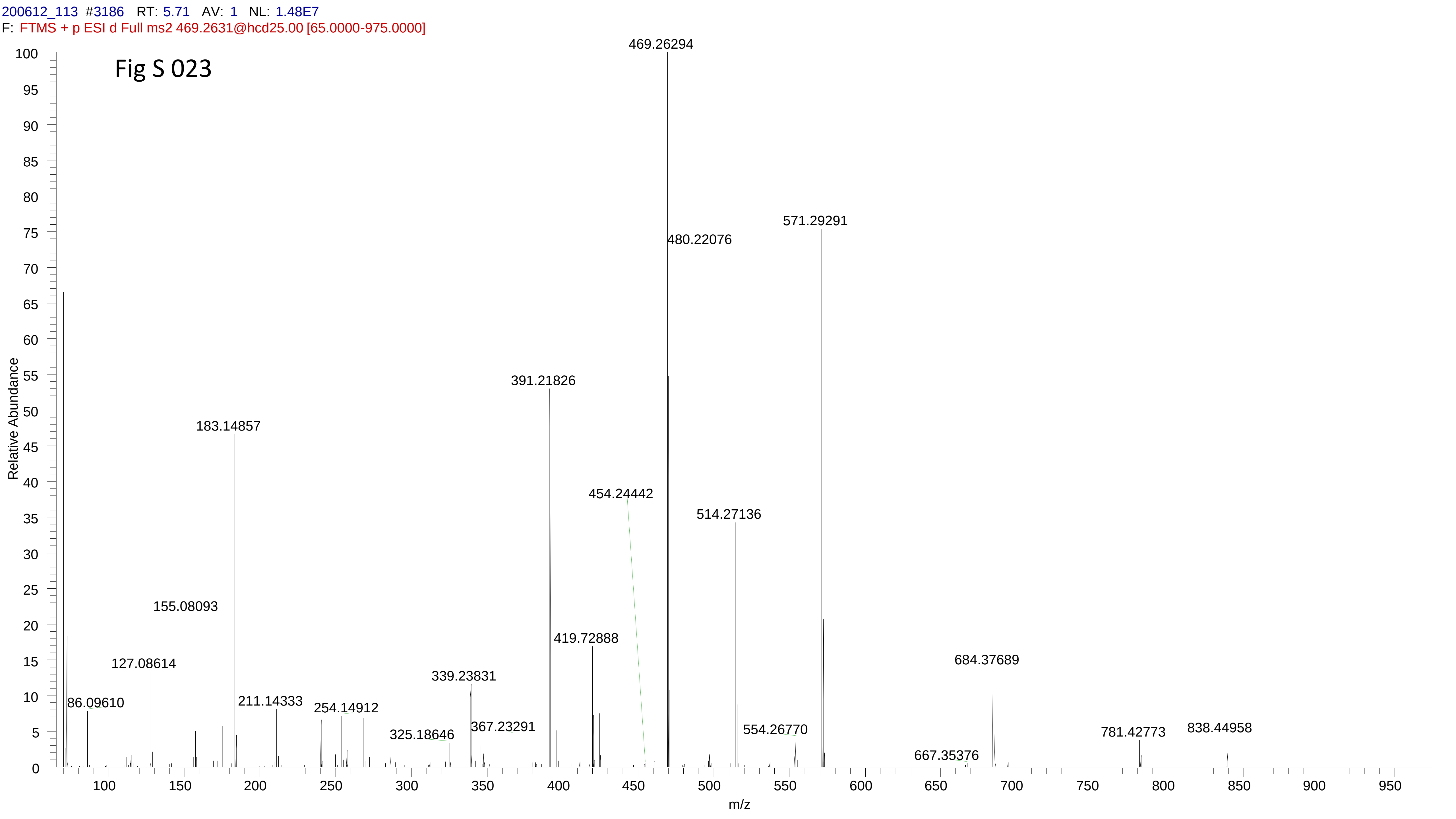
200612_113 #3186 RT: 5.71 AV: 1 NL: 1.48E7
F: FTMS + p ESI d Full ms2 469.2631@hcd25.00 [65.0000-975.0000]
Fig S 023
469.26294
480.22076
571.29291
391.21826
183.14857
454.24442
514.27136
155.08093
419.72888
127.08614
339.23831
684.37689
86.09610
211.14333
254.14912
325.18646
367.23291
554.26770
667.35376
781.42773
838.44958
Relative Abundance
m/z

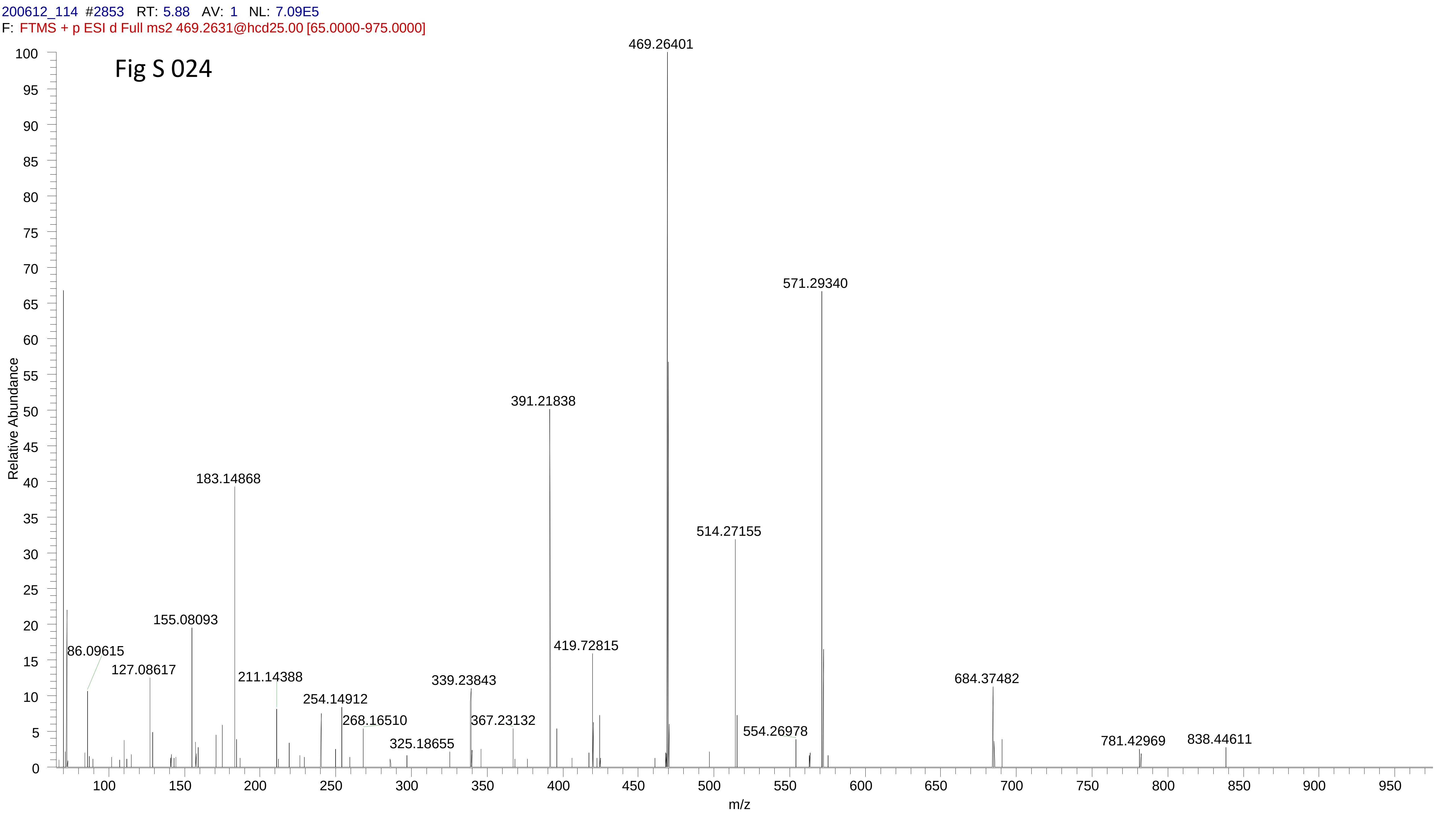
200612_114 #2853 RT: 5.88 AV: 1 NL: 7.09E5
F: FTMS + p ESI d Full ms2 469.2631@hcd25.00 [65.0000-975.0000]
Fig S 024
469.26401
571.29340
391.21838
183.14868
514.27155
155.08093
419.72815
86.09615
127.08617
211.14388
339.23843
254.14912
268.16510
367.23132
325.18655
554.26978
684.37482
781.42969
838.44611
Relative Abundance
m/z

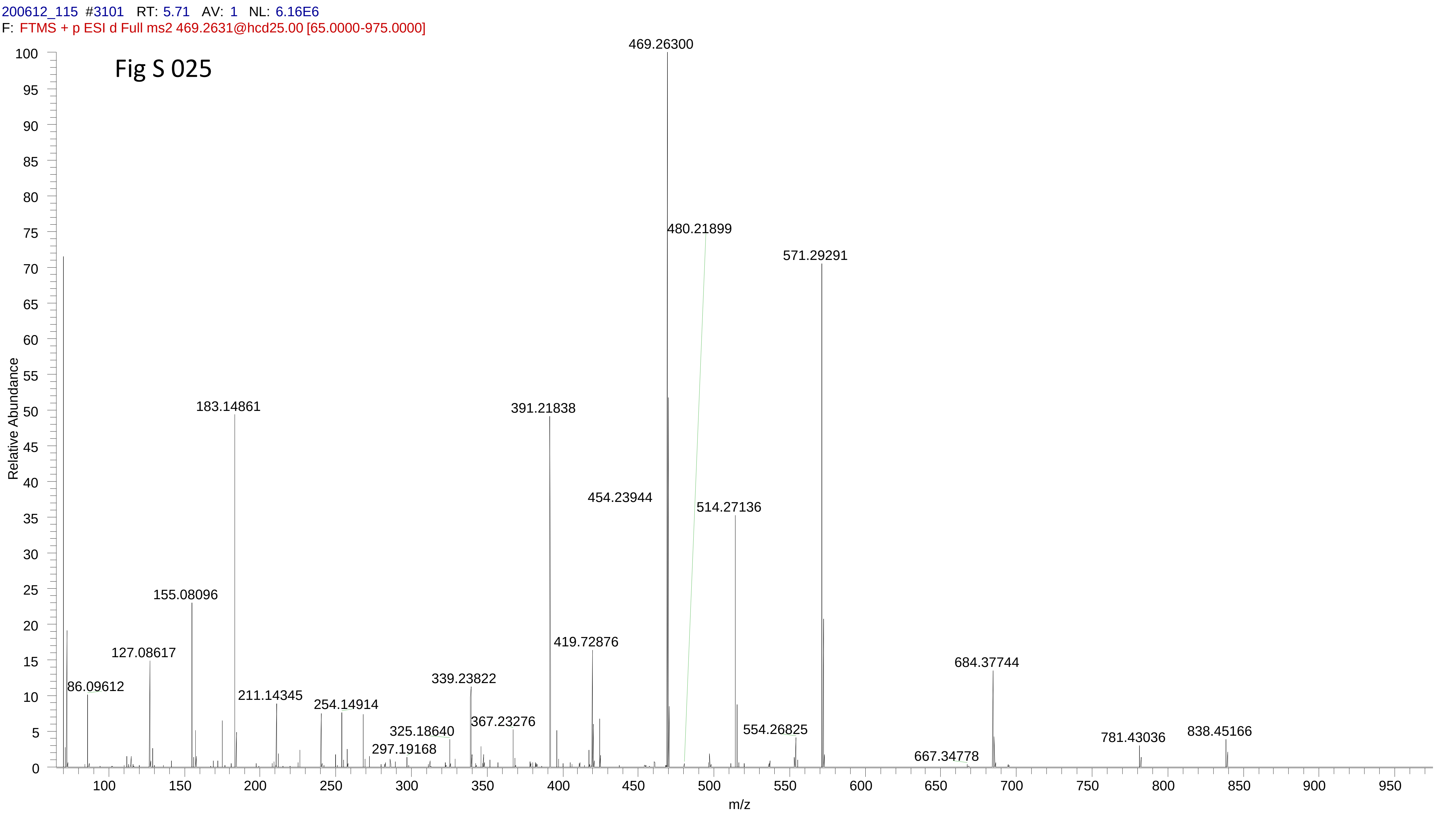
200612_115 #3101 RT: 5.71 AV: 1 NL: 6.16E6
F: FTMS + p ESI d Full ms2 469.2631@hcd25.00 [65.0000-975.0000]
Fig S 025
Relative Abundance
m/z
469.26300
480.21899
571.29291
183.14861
391.21838
454.23944
514.27136
155.08096
419.72876
127.08617
684.37744
86.09612
339.23822
211.14345
254.14914
367.23276
325.18640
554.26825
297.19168
781.43036
838.45166
667.34778

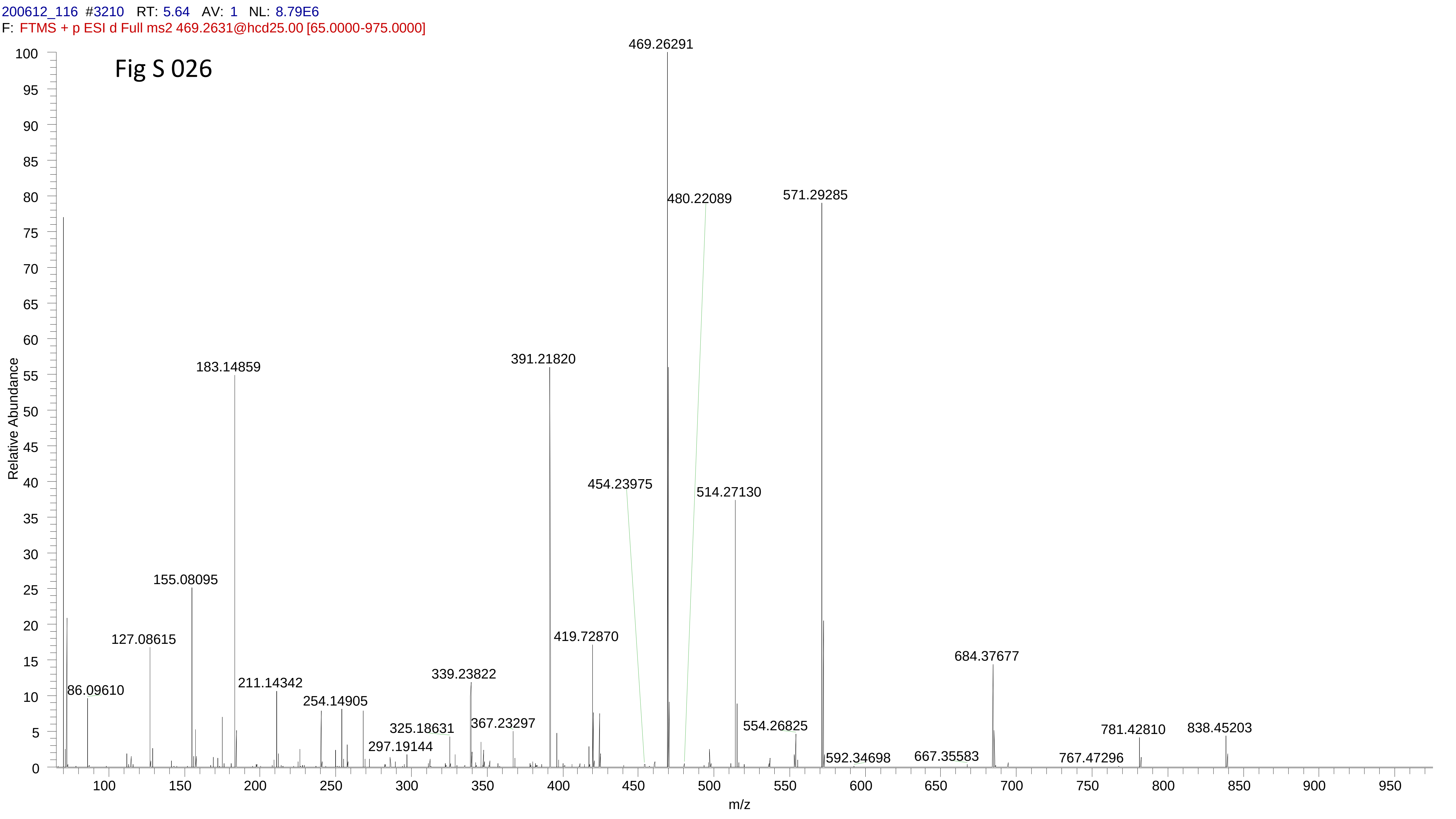
200612_116 #3210 RT: 5.64 AV: 1 NL: 8.79E6
F: FTMS + p ESI d Full ms2 469.2631@hcd25.00 [65.0000-975.0000]
Fig S 026
Relative Abundance
m/z
469.26291
480.22089
571.29285
391.21820
183.14859
454.23975
514.27130
155.08095
127.08615
419.72870
684.37677
86.09610
211.14342
339.23822
254.14905
325.18631
367.23297
297.19144
554.26825
592.34698
667.35583
767.47296
781.42810
838.45203

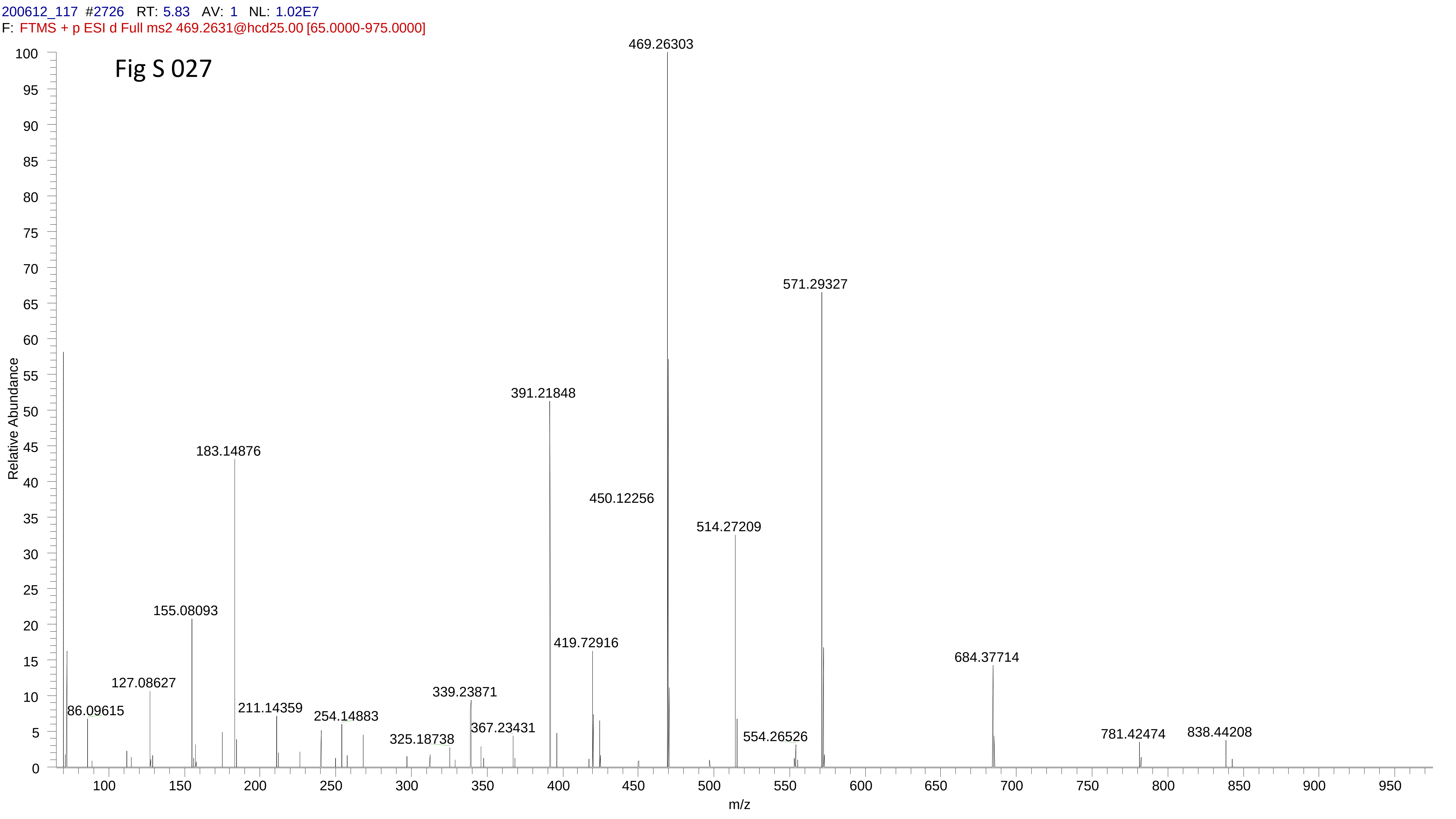
200612_117 #2726 RT: 5.83 AV: 1 NL: 1.02E7
F: FTMS + p ESI d Full ms2 469.2631@hcd25.00 [65.0000-975.0000]
Fig S 027
469.26303
571.29327
391.21848
183.14876
450.12256
514.27209
155.08093
419.72916
684.37714
127.08627
339.23871
86.09615
211.14359
254.14883
367.23431
325.18738
554.26526
781.42474
838.44208
Relative Abundance
m/z

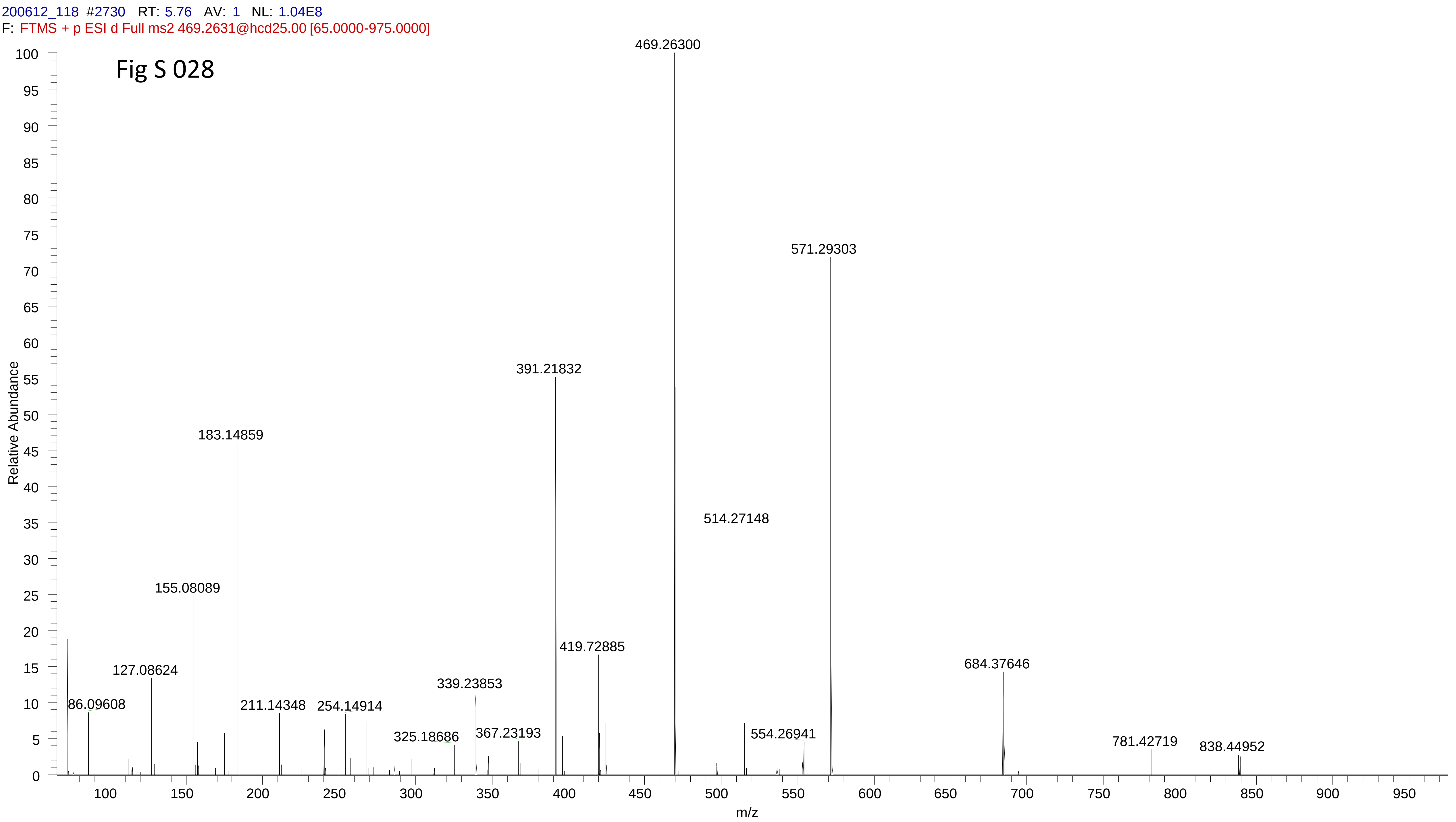
200612_118 #2730 RT: 5.76 AV: 1 NL: 1.04E8
F: FTMS + p ESI d Full ms2 469.2631@hcd25.00 [65.0000-975.0000]
Fig S 028
469.26300
571.29303
391.21832
183.14859
514.27148
155.08089
419.72885
684.37646
127.08624
339.23853
86.09608
211.14348
254.14914
325.18686
367.23193
554.26941
781.42719
838.44952
Relative Abundance
m/z

S2 File. Chromatograms and MS/MS spectra.

Raw data MS/MS spectra deamidated VGPIGPAGNR

Fig S 029: ostrich tendon
Fig S 030: goose neck
Fig S 031: duck neck
Fig S 032: turkey neck
Fig S 033: chicken leg
Fig S 034: goose meat strip
Fig S 035: goose leg
Fig S 036: guinea fowl torso
Fig S 037: pigeon torso
Fig S 038: partridge torso
Fig S 039: duck leg
Fig S 040: quail leg
Fig S 041: turkey leg

Remarks:
-Precursor $m/z \approx 469.76$
-Pheasant samples did not provide MS/MS spectra for this peptide.
-Data recorded in June 2020.
-See Fig 4a in the main document for peak annotation, corrected for the presence of deamidated N9 (+ 0.984 Da).

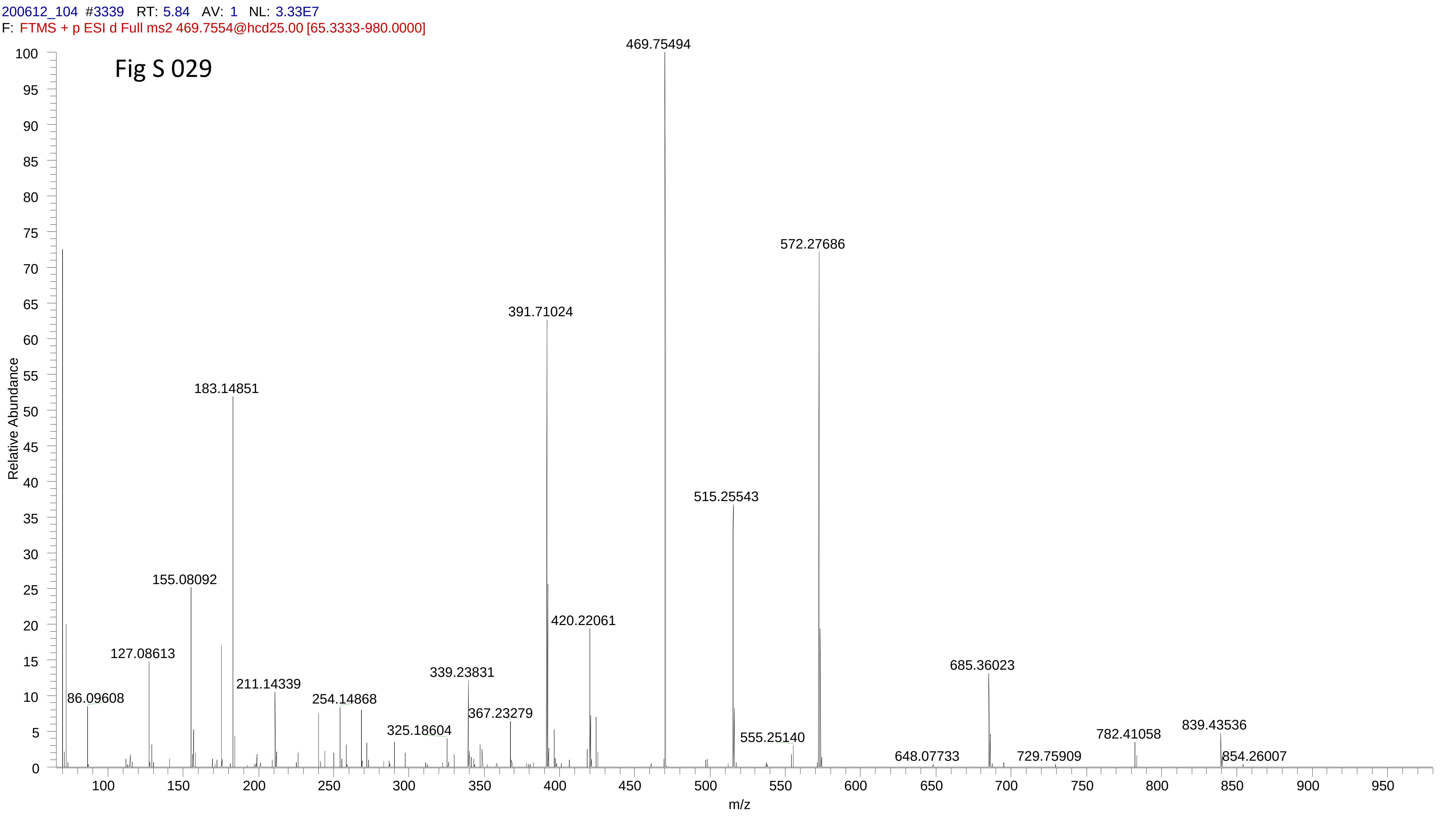
200612_104 #3339 RT: 5.84 AV: 1 NL: 3.33E7
F: FTMS + p ESI d Full ms2 469.7554@hcd25.00 [65.3333-980.0000]
Fig S 029
469.75494
572.27686
391.71024
183.14851
515.25543
155.08092
420.22061
127.08613
685.36023
339.23831
211.14339
86.09608
254.14868
367.23279
325.18604
555.25140
782.41058
839.43536
648.07733
729.75909
854.26007
Relative Abundance
m/z

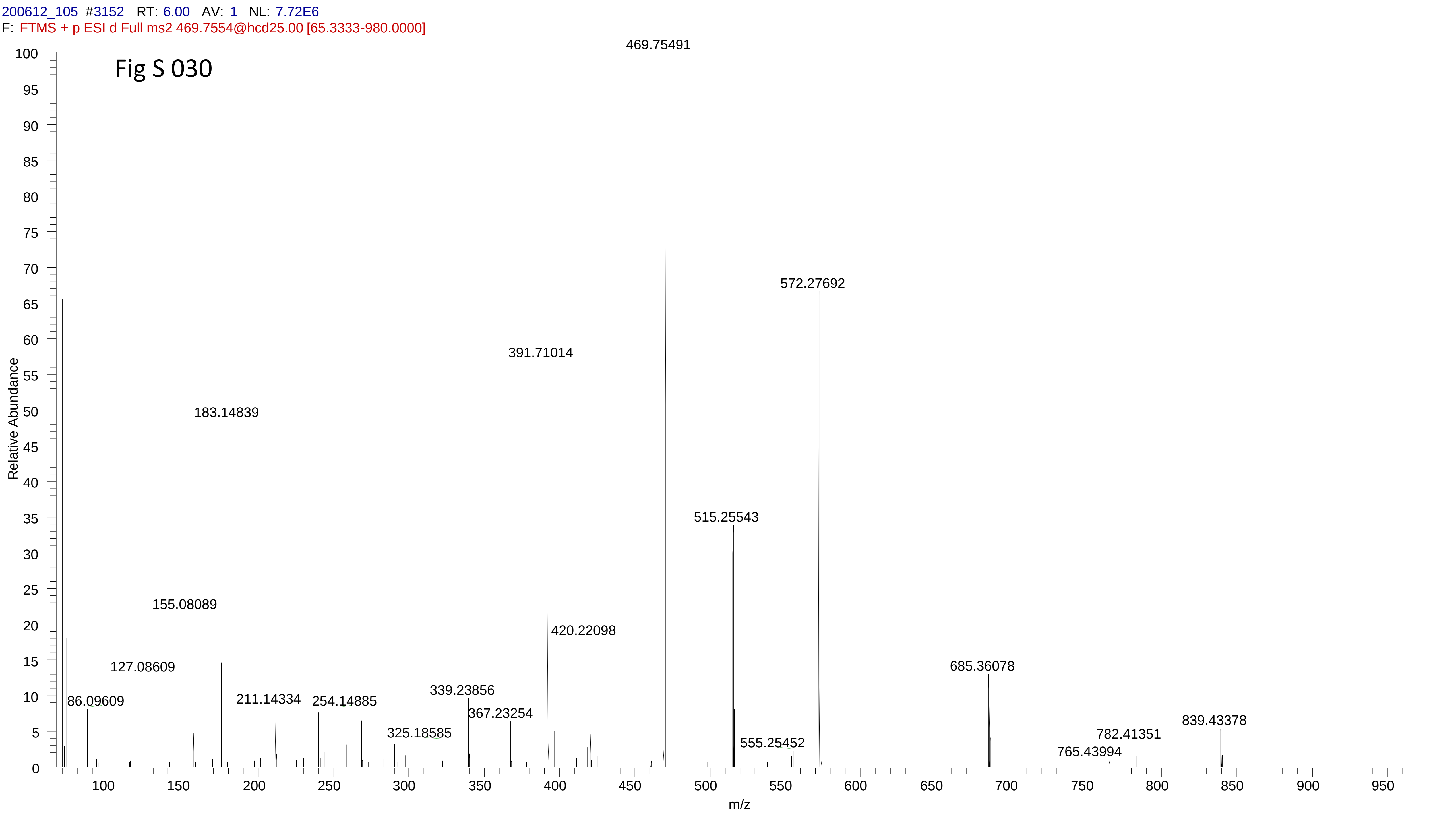
200612_105 #3152 RT: 6.00 AV: 1 NL: 7.72E6
F: FTMS + p ESI d Full ms2 469.7554@hcd25.00 [65.3333-980.0000]
Fig S 030
Relative Abundance
m/z
469.75491
572.27692
391.71014
183.14839
515.25543
420.22098
155.08089
127.08609
685.36078
339.23856
86.09609
211.14334
254.14885
367.23254
839.43378
325.18585
782.41351
555.25452
765.43994

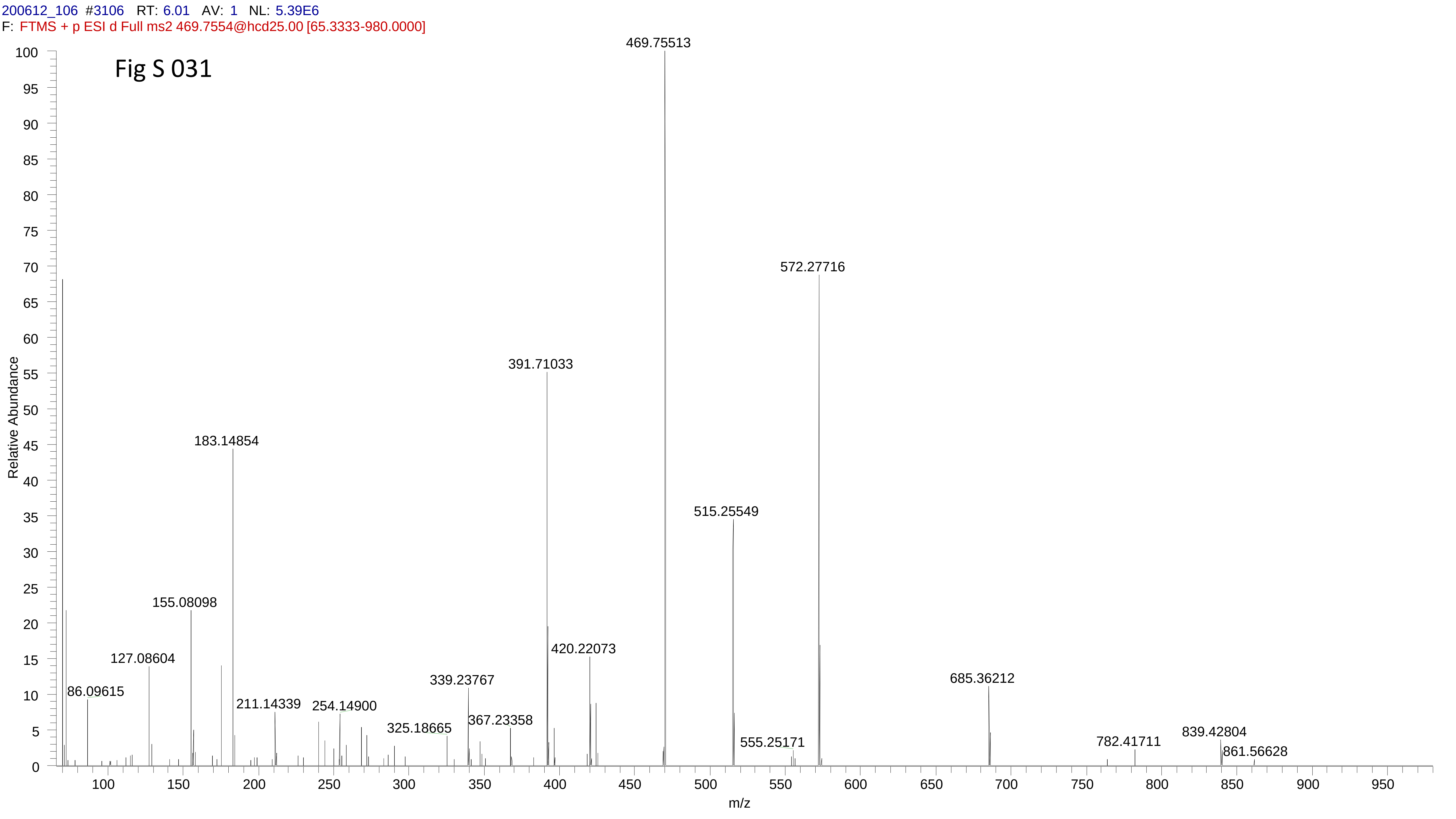
200612_106 #3106 RT: 6.01 AV: 1 NL: 5.39E6
F: FTMS + p ESI d Full ms2 469.7554@hcd25.00 [65.3333-980.0000]
Fig S 031
469.75513
572.27716
391.71033
183.14854
515.25549
155.08098
420.22073
127.08604
86.09615
339.23767
685.36212
211.14339
254.14900
367.23358
325.18665
839.42804
555.25171
782.41711
861.56628
Relative Abundance
m/z

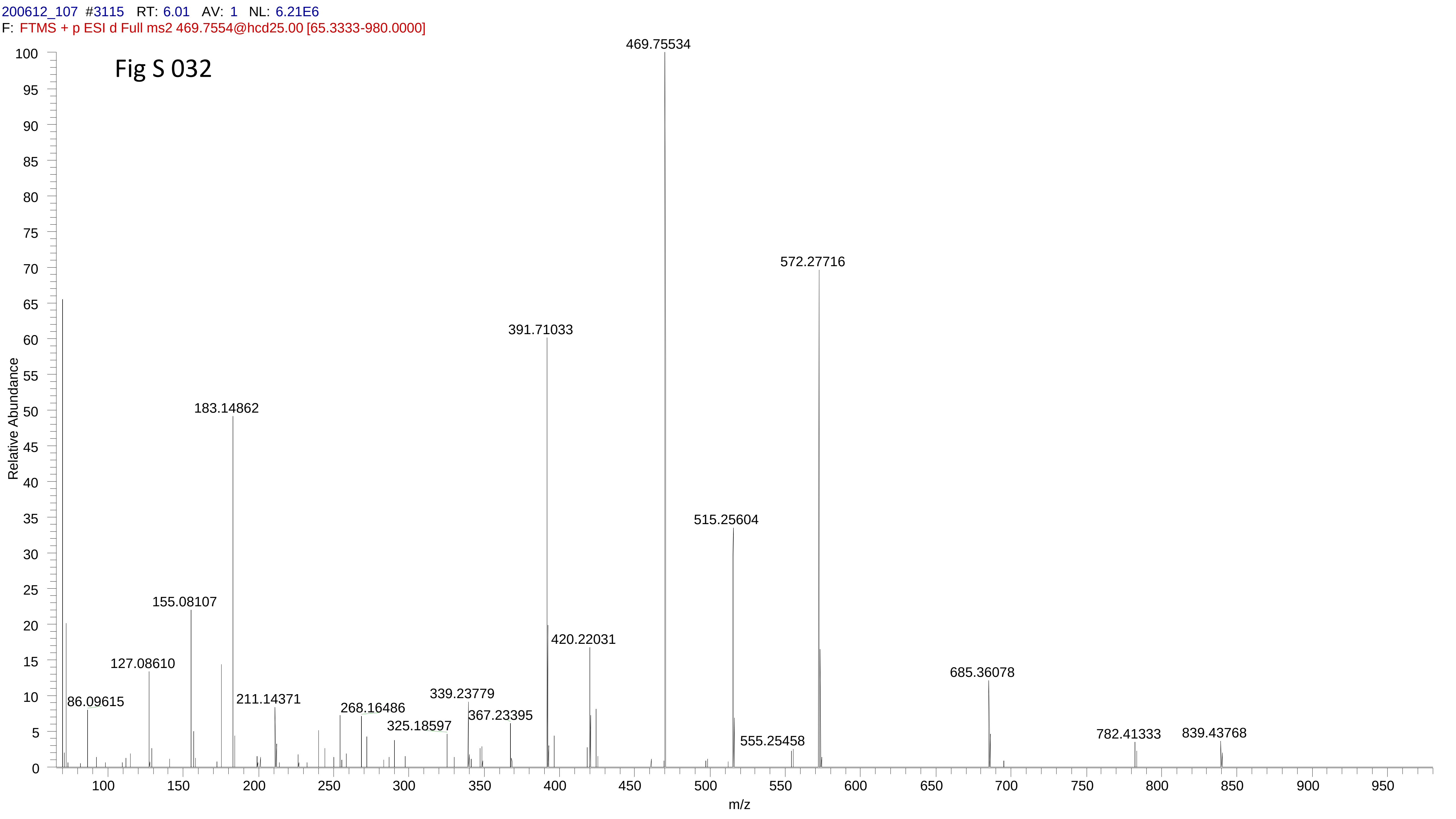
200612_107 #3115 RT: 6.01 AV: 1 NL: 6.21E6
F: FTMS + p ESI d Full ms2 469.7554@hcd25.00 [65.3333-980.0000]
Fig S 032
469.75534
572.27716
391.71033
183.14862
515.25604
420.22031
155.08107
127.08610
685.36078
339.23779
86.09615
211.14371
268.16486
367.23395
325.18597
555.25458
782.41333
839.43768
Relative Abundance
m/z

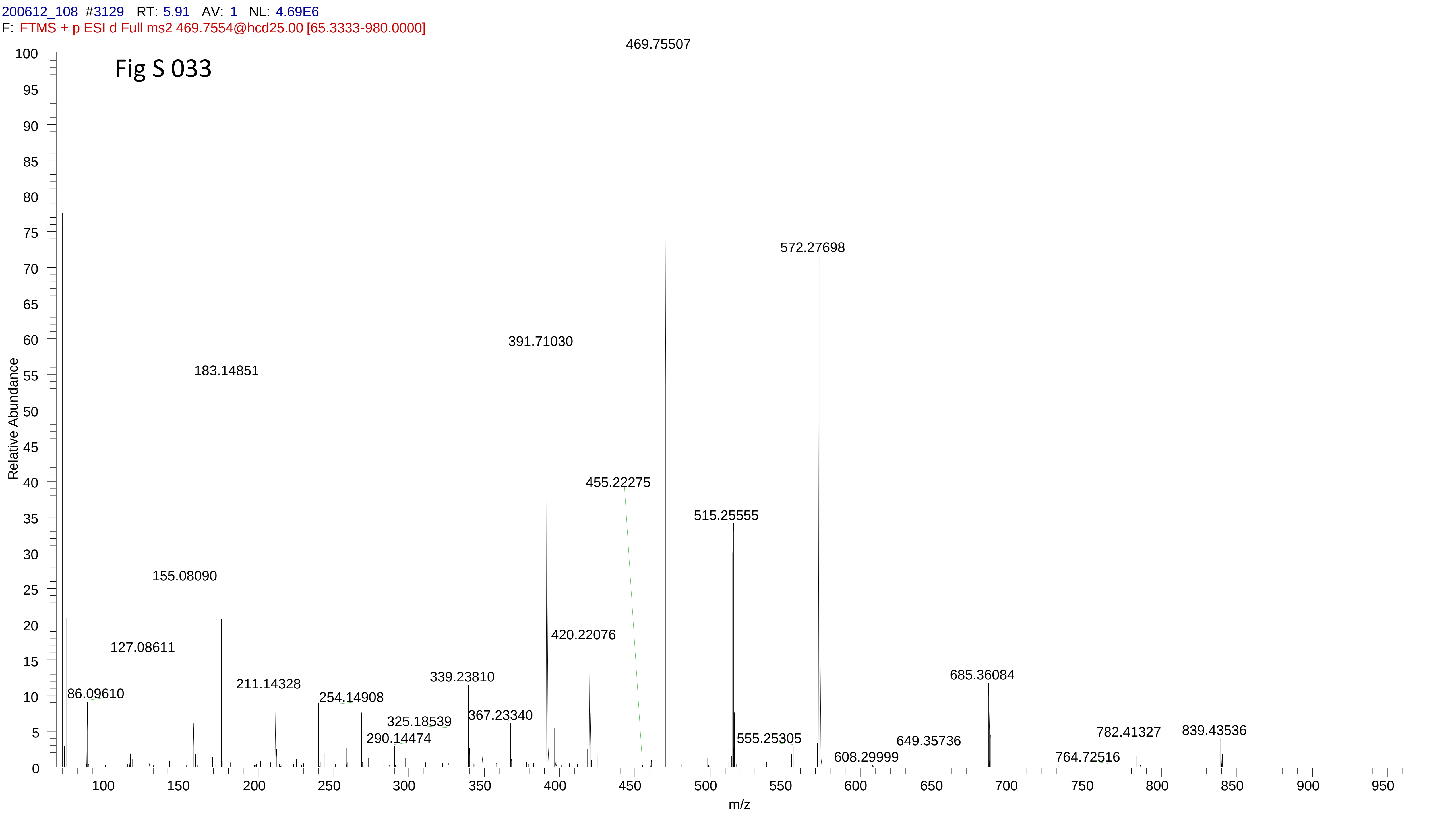
200612_108 #3129 RT: 5.91 AV: 1 NL: 4.69E6
F: FTMS + p ESI d Full ms2 469.7554@hcd25.00 [65.3333-980.0000]
Fig S 033
469.75507
572.27698
391.71030
183.14851
455.22275
515.25555
155.08090
420.22076
127.08611
339.23810
685.36084
211.14328
86.09610
254.14908
367.23340
325.18539
290.14474
555.25305
649.35736
782.41327
839.43536
608.29999
764.72516
Relative Abundance
m/z

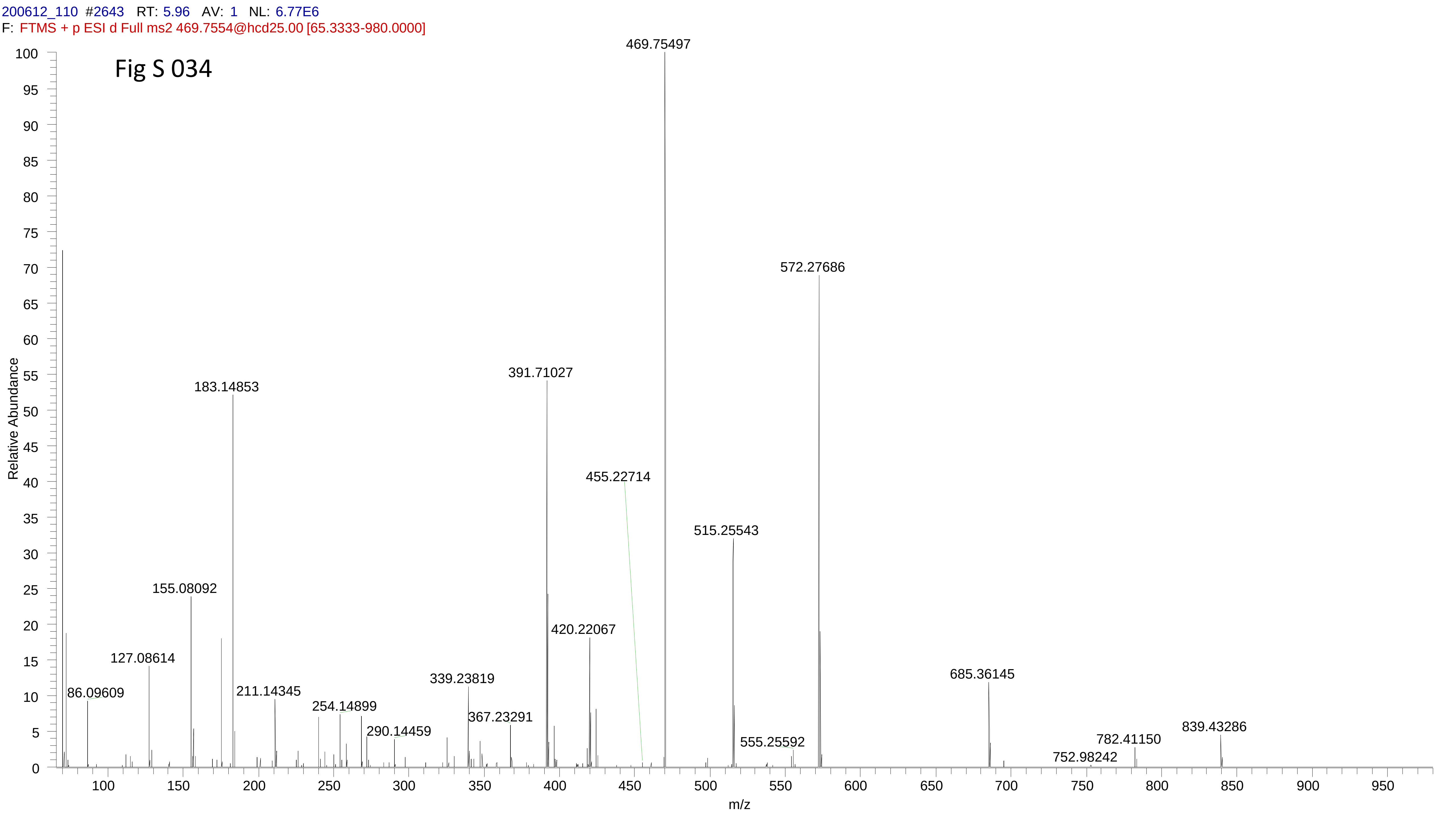
200612_110 #2643 RT: 5.96 AV: 1 NL: 6.77E6
F: FTMS + p ESI d Full ms2 469.7554@hcd25.00 [65.3333-980.0000]
Fig S 034
469.75497
572.27686
391.71027
183.14853
455.22714
515.25543
155.08092
420.22067
127.08614
685.36145
86.09609
211.14345
339.23819
254.14899
367.23291
290.14459
839.43286
782.41150
555.25592
752.98242
Relative Abundance
m/z

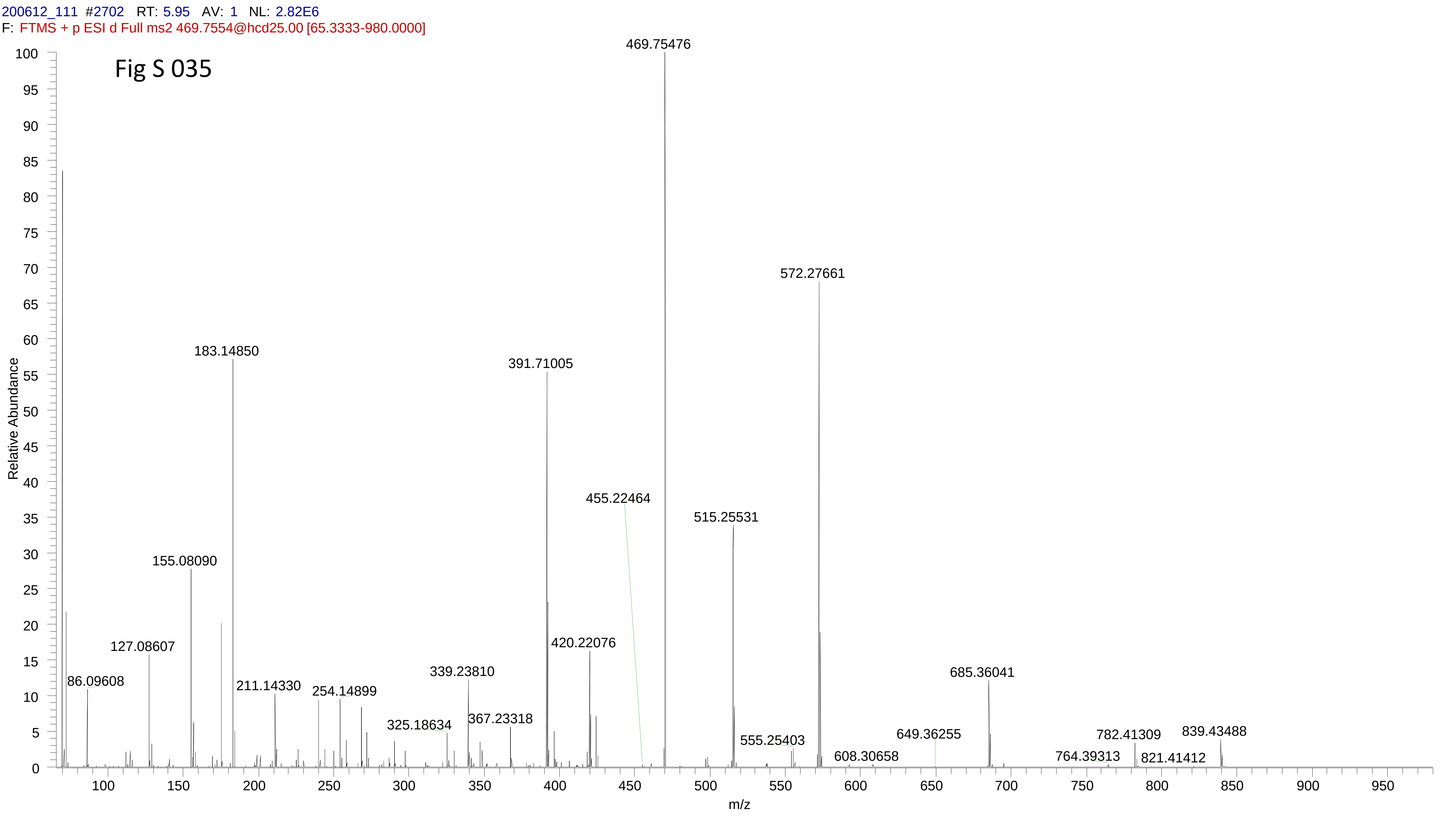
200612_111 #2702 RT: 5.95 AV: 1 NL: 2.82E6
F: FTMS + p ESI d Full ms2 469.7554@hcd25.00 [65.3333-980.0000]
Fig S 035
469.75476
572.27661
183.14850
391.71005
455.22464
515.25531
155.08090
127.08607
420.22076
86.09608
211.14330
254.14899
339.23810
685.36041
325.18634
367.23318
555.25403
649.36255
782.41309
839.43488
608.30658
764.39313
821.41412
Relative Abundance
m/z

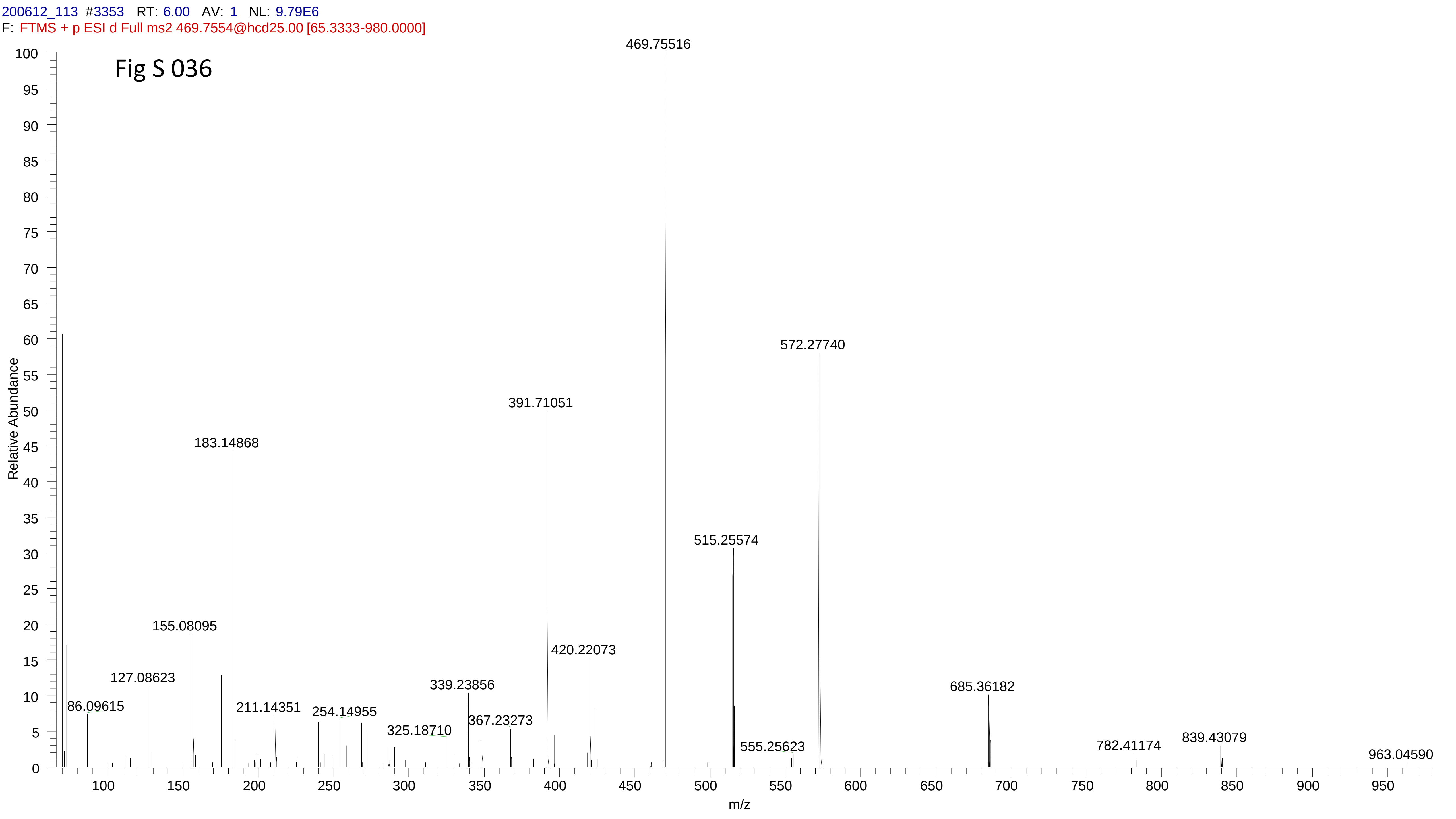
200612_113 #3353 RT: 6.00 AV: 1 NL: 9.79E6
F: FTMS + p ESI d Full ms2 469.7554@hcd25.00 [65.3333-980.0000]
Fig S 036
469.75516
572.27740
391.71051
183.14868
515.25574
155.08095
420.22073
127.08623
339.23856
685.36182
86.09615
211.14351
254.14955
367.23273
325.18710
555.25623
782.41174
839.43079
963.04590
Relative Abundance
m/z

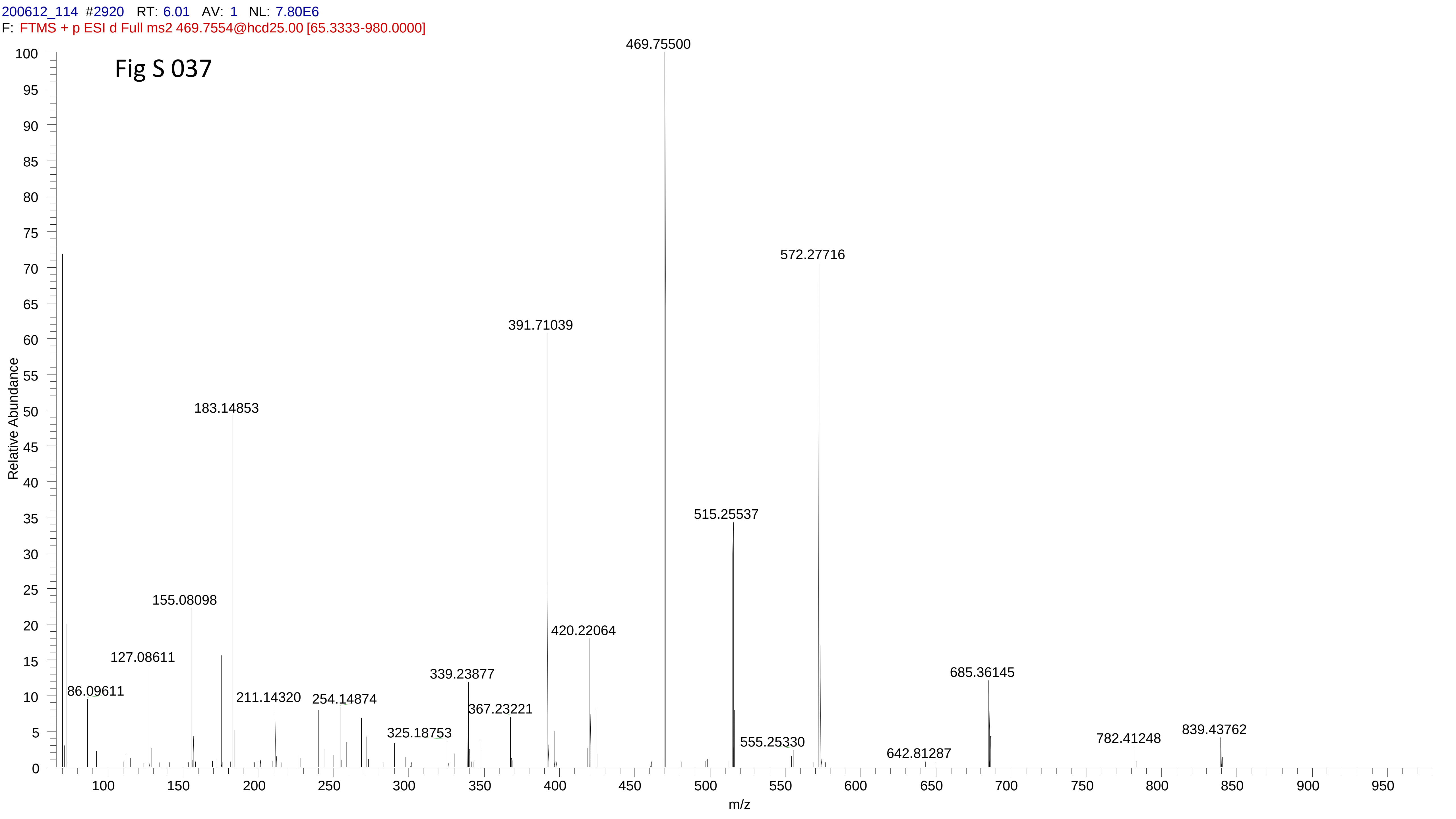
200612_114 #2920 RT: 6.01 AV: 1 NL: 7.80E6
F: FTMS + p ESI d Full ms2 469.7554@hcd25.00 [65.3333-980.0000]
Fig S 037
469.75500
572.27716
391.71039
183.14853
515.25537
155.08098
420.22064
127.08611
339.23877
685.36145
86.09611
211.14320
254.14874
367.23221
325.18753
555.25330
642.81287
782.41248
839.43762
Relative Abundance
m/z

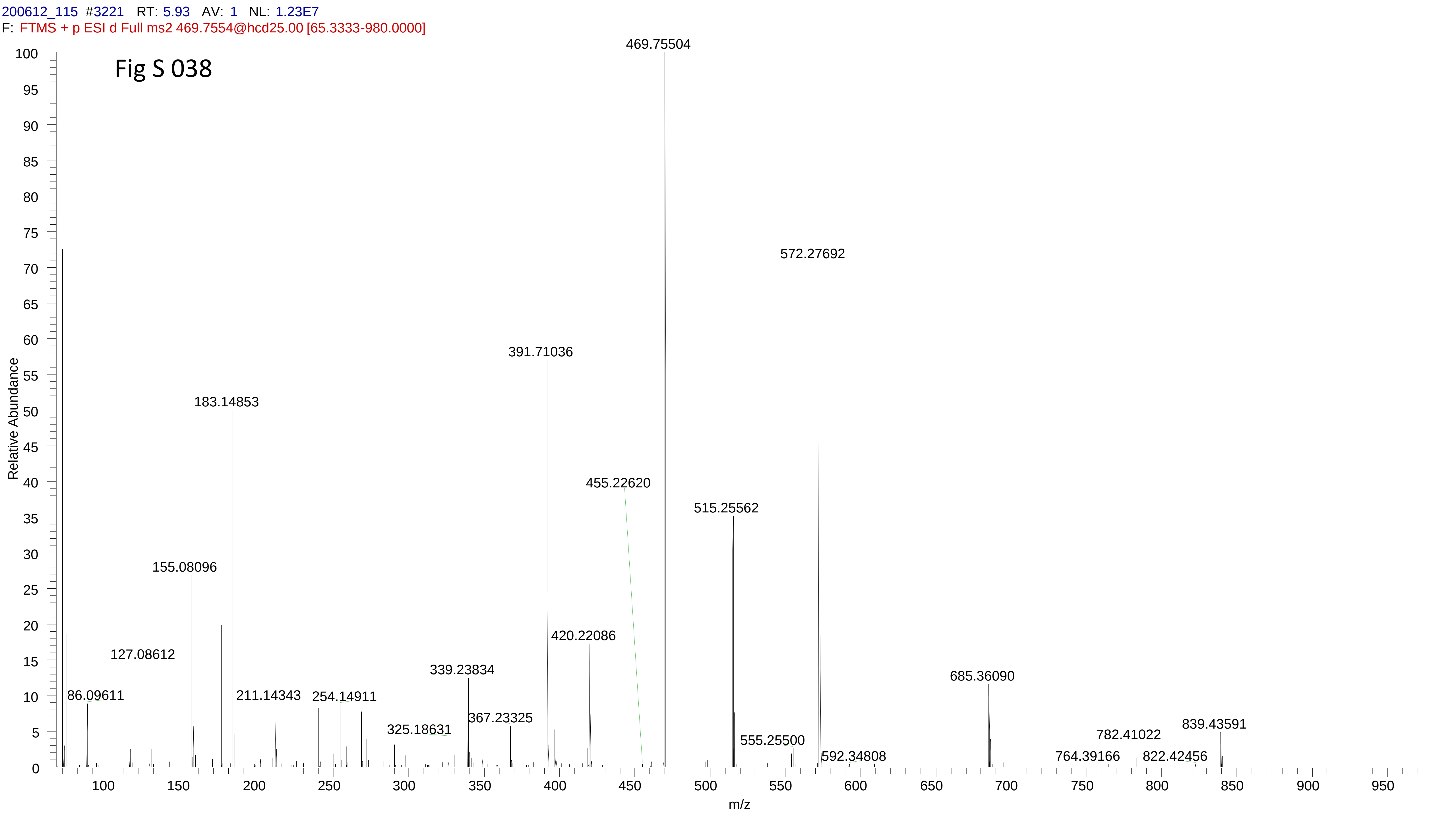
200612_115 #3221 RT: 5.93 AV: 1 NL: 1.23E7
F: FTMS + p ESI d Full ms2 469.7554@hcd25.00 [65.3333-980.0000]
Fig S 038
Relative Abundance
m/z
469.75504
572.27692
391.71036
183.14853
455.22620
515.25562
155.08096
420.22086
127.08612
339.23834
685.36090
86.09611
211.14343
254.14911
367.23325
325.18631
839.43591
782.41022
555.25500
592.34808
764.39166
822.42456

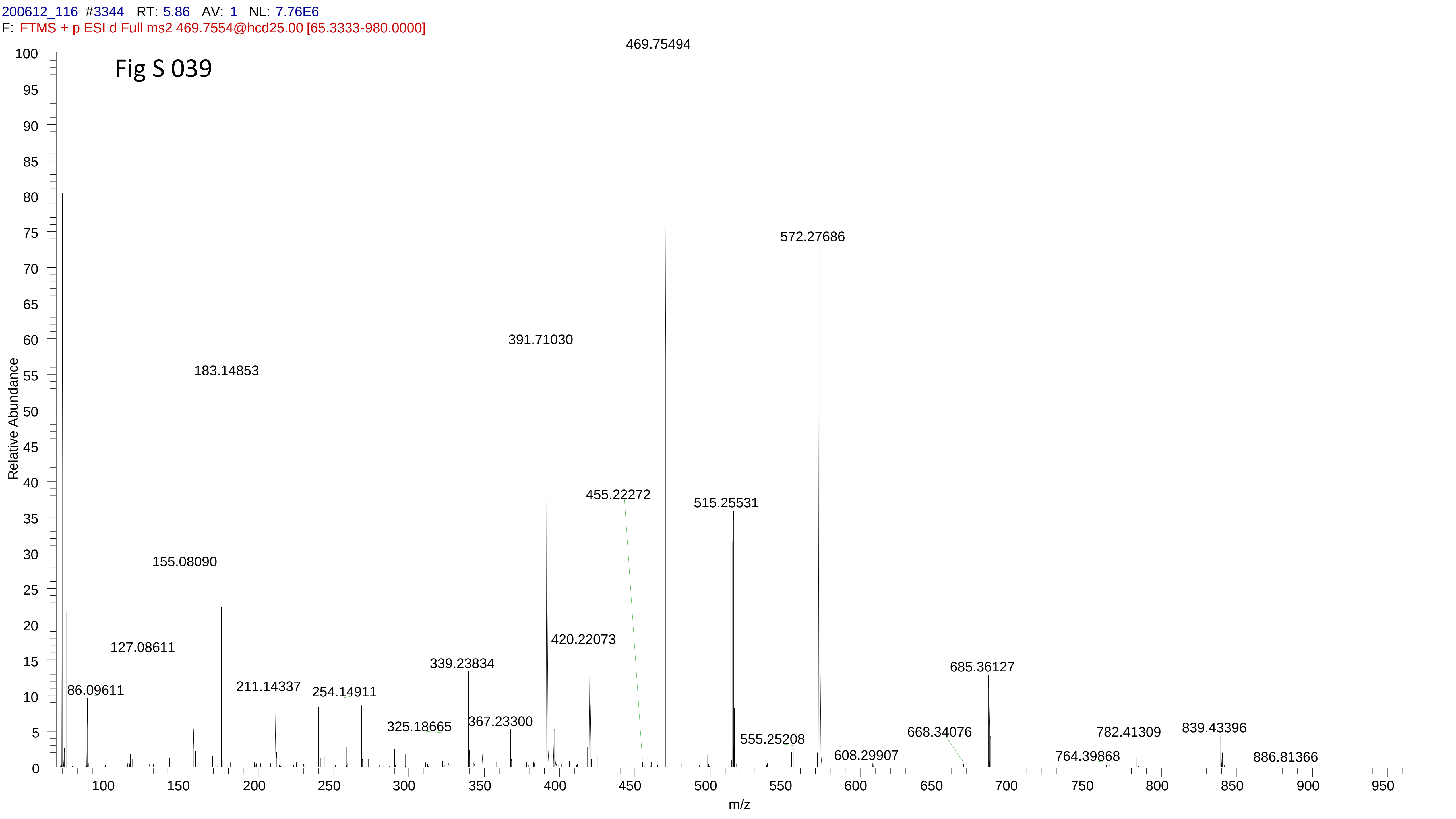
200612_116 #3344 RT: 5.86 AV: 1 NL: 7.76E6
F: FTMS + p ESI d Full ms2 469.7554@hcd25.00 [65.3333-980.0000]
Fig S 039
Relative Abundance
m/z
469.75494
572.27686
391.71030
183.14853
455.22272
515.25531
155.08090
127.08611
420.22073
339.23834
685.36127
86.09611
211.14337
254.14911
325.18665
367.23300
555.25208
608.29907
668.34076
764.39868
782.41309
839.43396
886.81366

Fig S 040

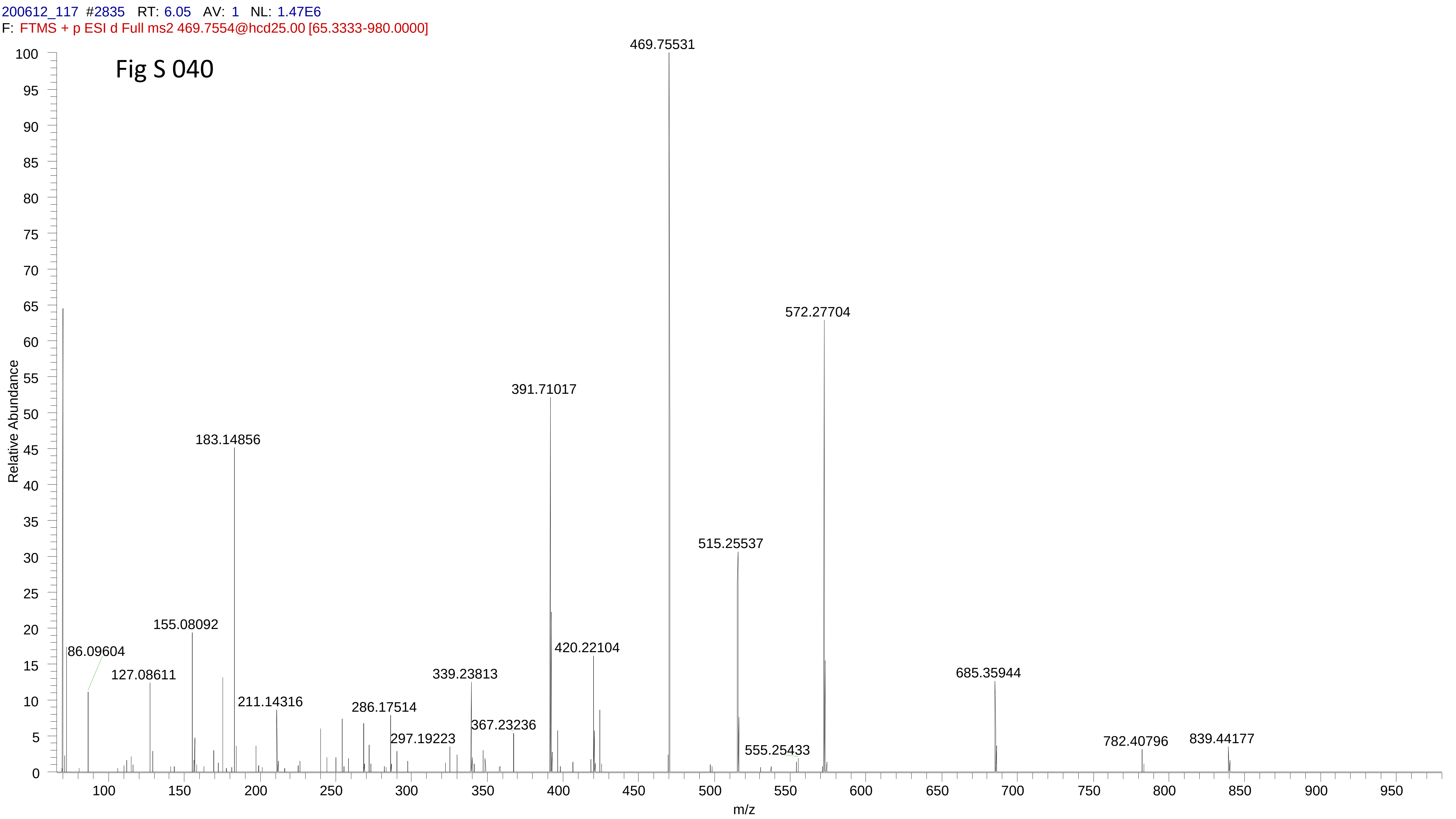
200612_117 #2835 RT: 6.05 AV: 1 NL: 1.47E6
F: FTMS + p ESI d Full ms2 469.7554@hcd25.00 [65.3333-980.0000]
Relative Abundance
m/z
469.75531
572.27704
391.71017
183.14856
515.25537
155.08092
86.09604
127.08611
420.22104
339.23813
685.35944
211.14316
286.17514
367.23236
297.19223
555.25433
782.40796
839.44177

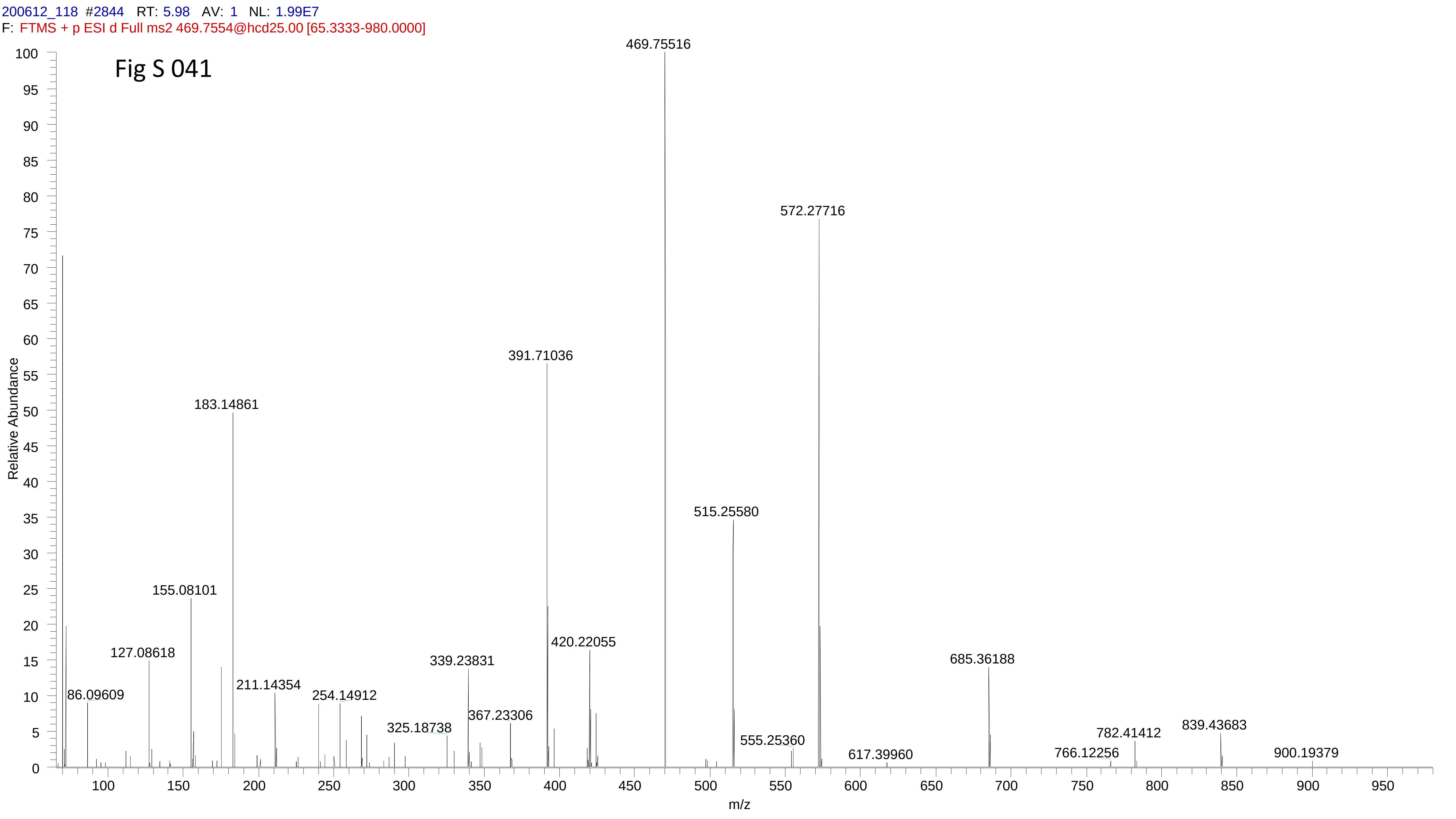
200612_118 #2844 RT: 5.98 AV: 1 NL: 1.99E7
F: FTMS + p ESI d Full ms2 469.7554@hcd25.00 [65.3333-980.0000]
Fig S 041
469.75516
572.27716
391.71036
183.14861
515.25580
155.08101
420.22055
127.08618
339.23831
685.36188
211.14354
86.09609
254.14912
367.23306
325.18738
839.43683
782.41412
555.25360
617.39960
766.12256
900.19379
Relative Abundance
m/z

S2 File. Chromatograms and MS/MS spectra.

Raw data chromatograms (deamidated) VGPIGAAGNR

Fig S 042: ostrich tendon
Fig S 043: goose neck
Fig S 044: duck neck
Fig S 045: turkey neck
Fig S 046: chicken leg
Fig S 047: pheasant meat strip
Fig S 048: goose meat strip
Fig S 049: goose leg
Fig S 050: pheasant leg
Fig S 051: guinea fowl torso
Fig S 052: pigeon torso
Fig S 053: partridge torso
Fig S 054: duck leg
Fig S 055: quail leg
Fig S 056: turkey leg

Remarks:
-Extracted m/z range 456.74-456.76
-The retention time and m/z of the base peak are provided per peak and the provided intensity is of the highest peak.
-Data recorded in June 2020.

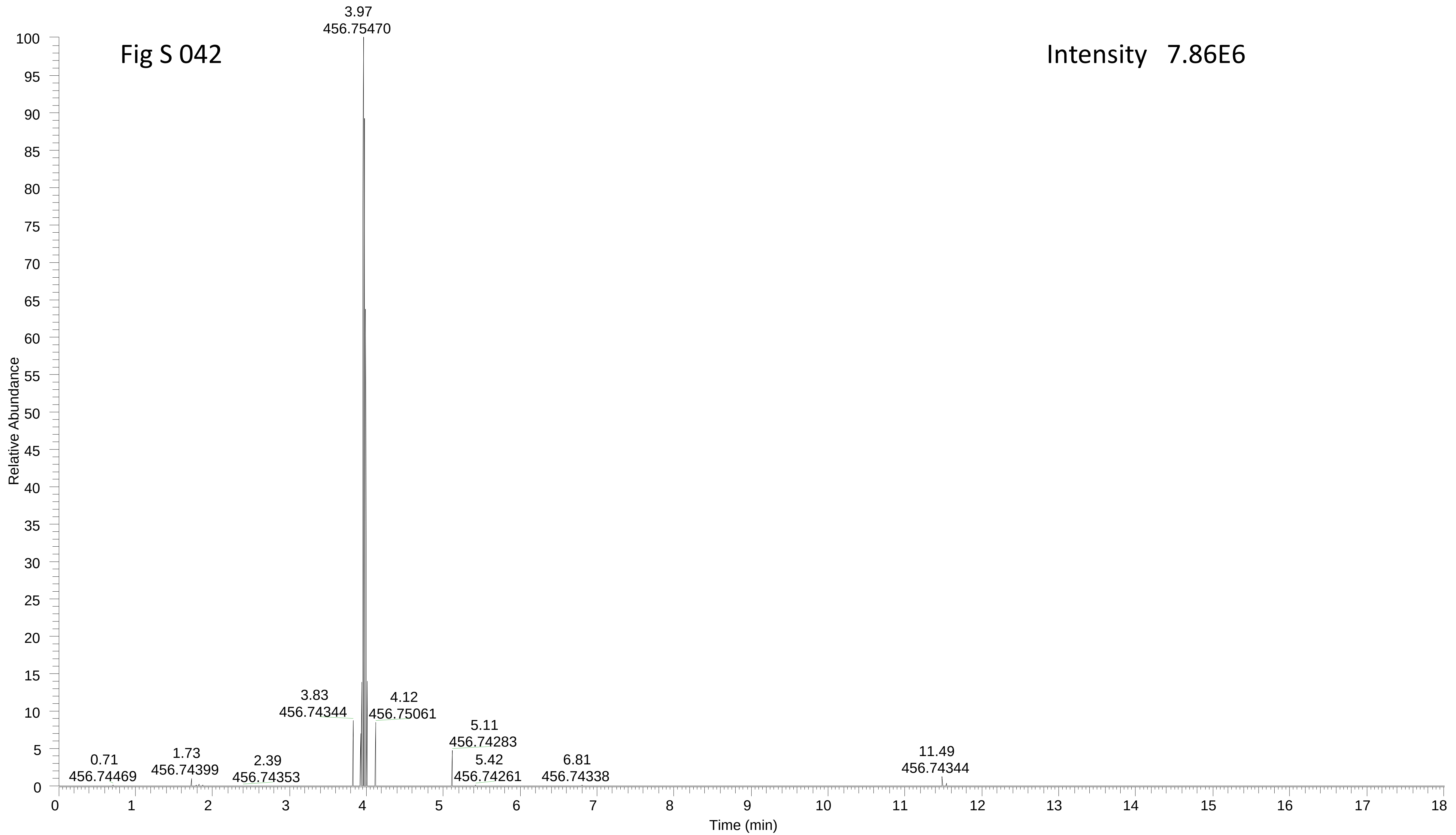
Fig S 042
Intensity 7.86E6
3.97
456.75470
3.83
456.74344
4.12
456.75061
5.11
456.74283
5.42
456.74261
6.81
456.74338
0.71
456.74469
1.73
456.74399
2.39
456.74353
11.49
456.74344
Relative Abundance
Time (min)
0
5
10
15
20
25
30
35
40
45
50
55
60
65
70
75
80
85
90
95
100
0
1
2
3
4
5
6
7
8
9
10
11
12
13
14
15
16
17
18

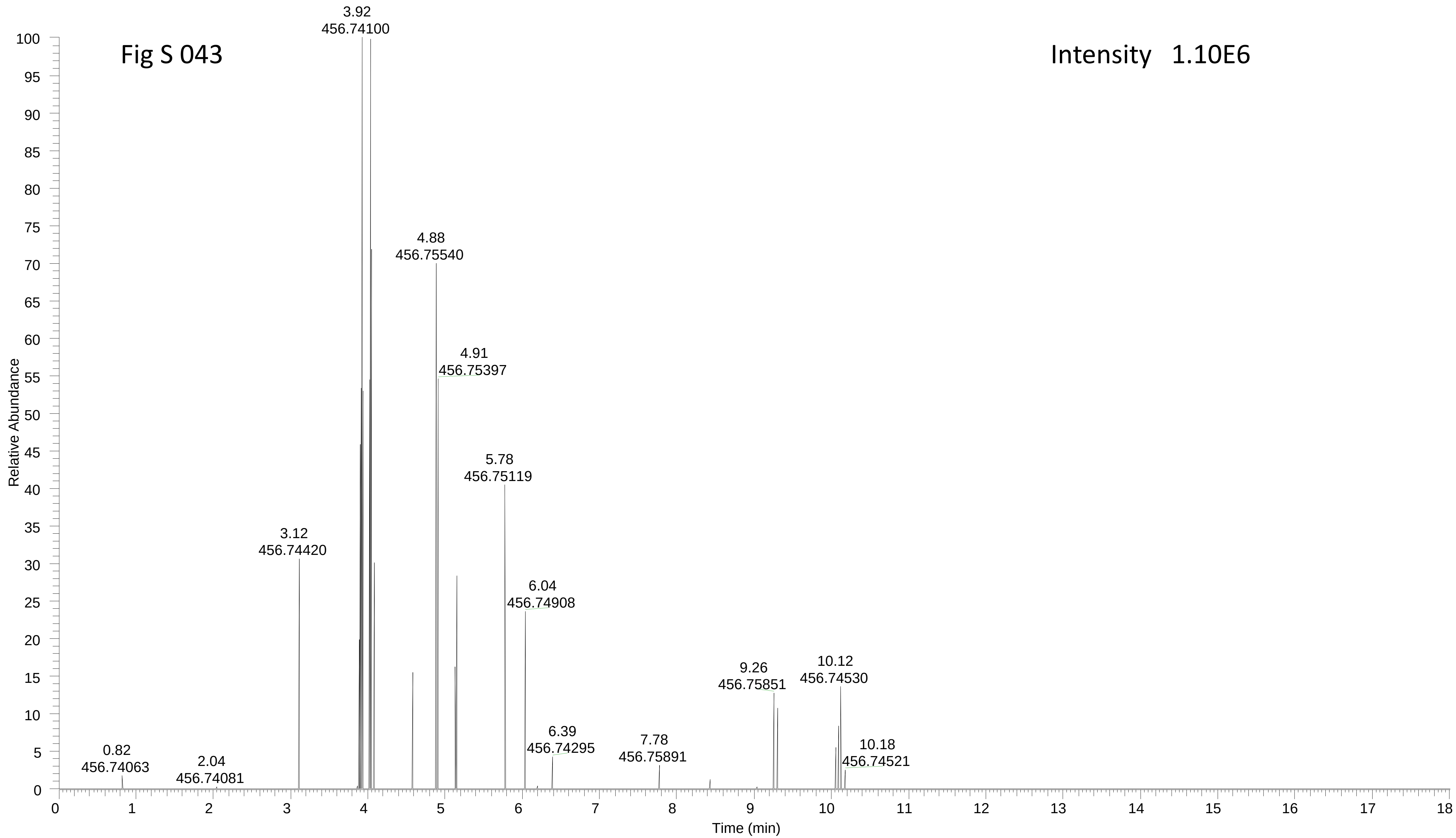
Fig S 043
Intensity 1.10E6
3.92
456.74100
4.88
456.75540
4.91
456.75397
5.78
456.75119
3.12
456.74420
6.04
456.74908
9.26
456.75851
10.12
456.74530
6.39
456.74295
7.78
456.75891
10.18
456.74521
0.82
456.74063
2.04
456.74081
Relative Abundance
Time (min)

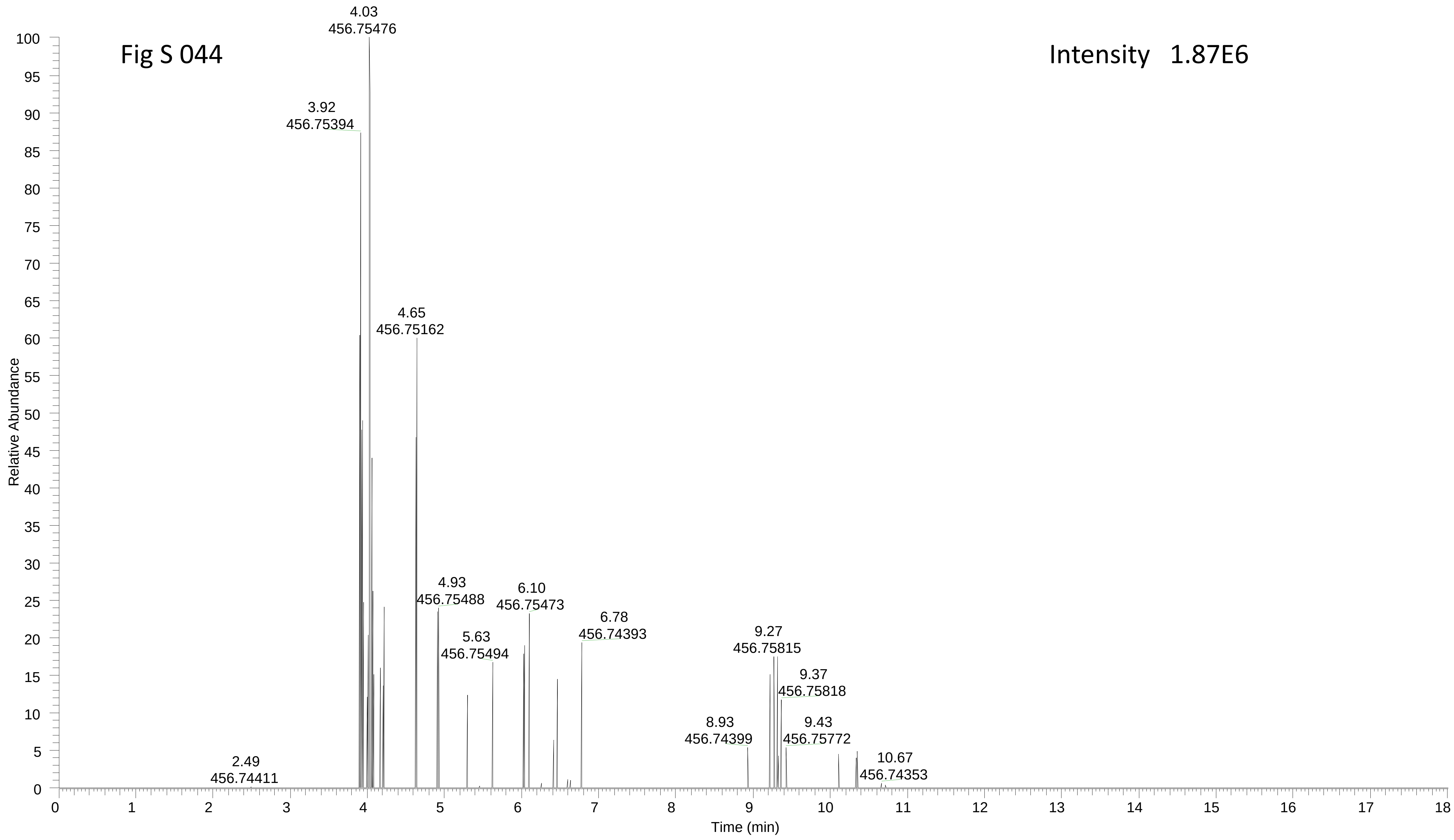
Fig S 044
Intensity 1.87E6
4.03
456.75476
3.92
456.75394
4.65
456.75162
4.93
456.75488
6.10
456.75473
5.63
456.75494
6.78
456.74393
9.27
456.75815
9.37
456.75818
8.93
456.74399
9.43
456.75772
2.49
456.74411
10.67
456.74353
Relative Abundance
Time (min)
0
5
10
15
20
25
30
35
40
45
50
55
60
65
70
75
80
85
90
95
100
0
1
2
3
4
5
6
7
8
9
10
11
12
13
14
15
16
17
18

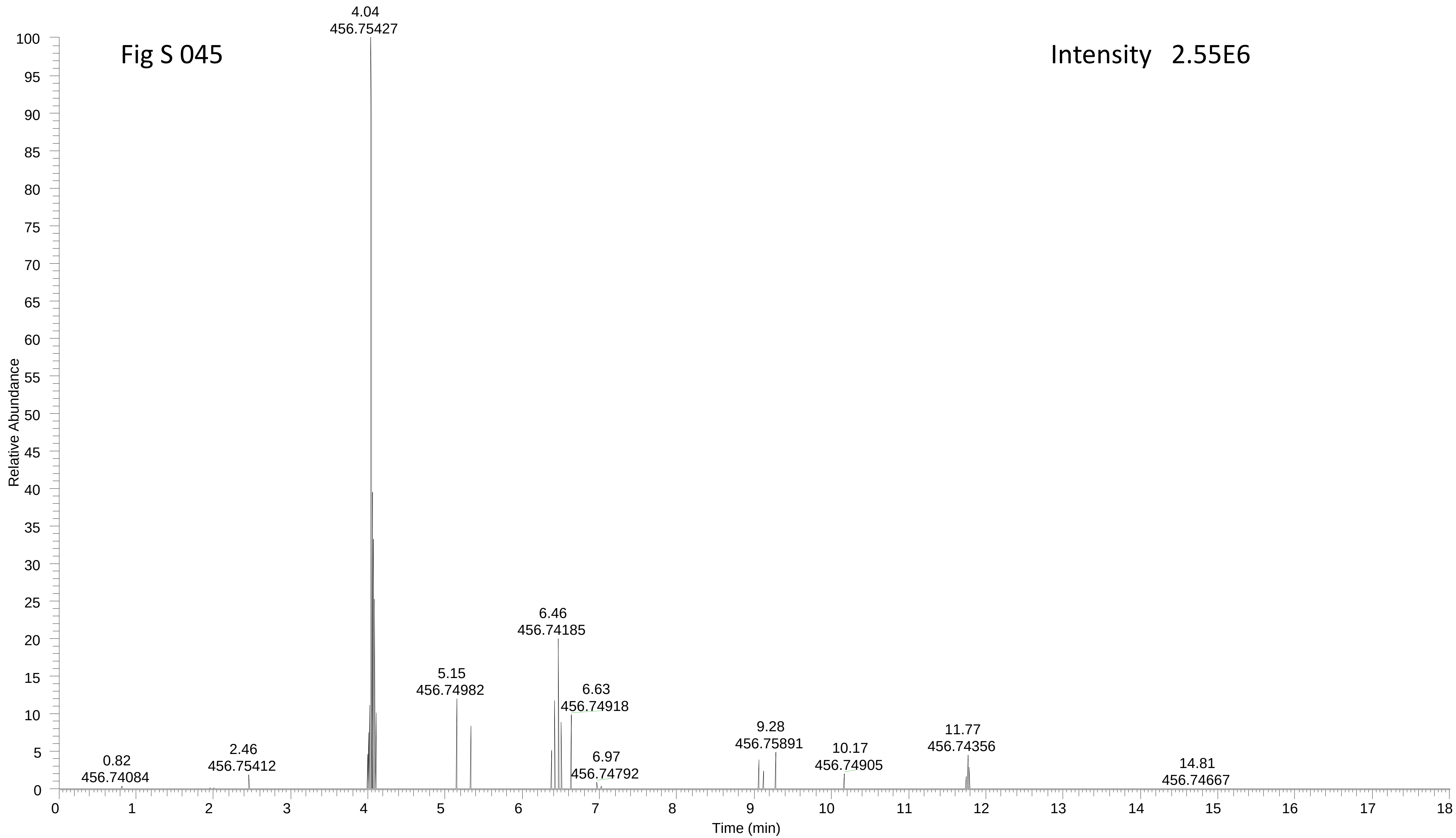
Fig S 045
Intensity 2.55E6
4.04
456.75427
0.82
456.74084
2.46
456.75412
5.15
456.74982
6.46
456.74185
6.63
456.74918
6.97
456.74792
9.28
456.75891
10.17
456.74905
11.77
456.74356
14.81
456.74667
Relative Abundance
Time (min)

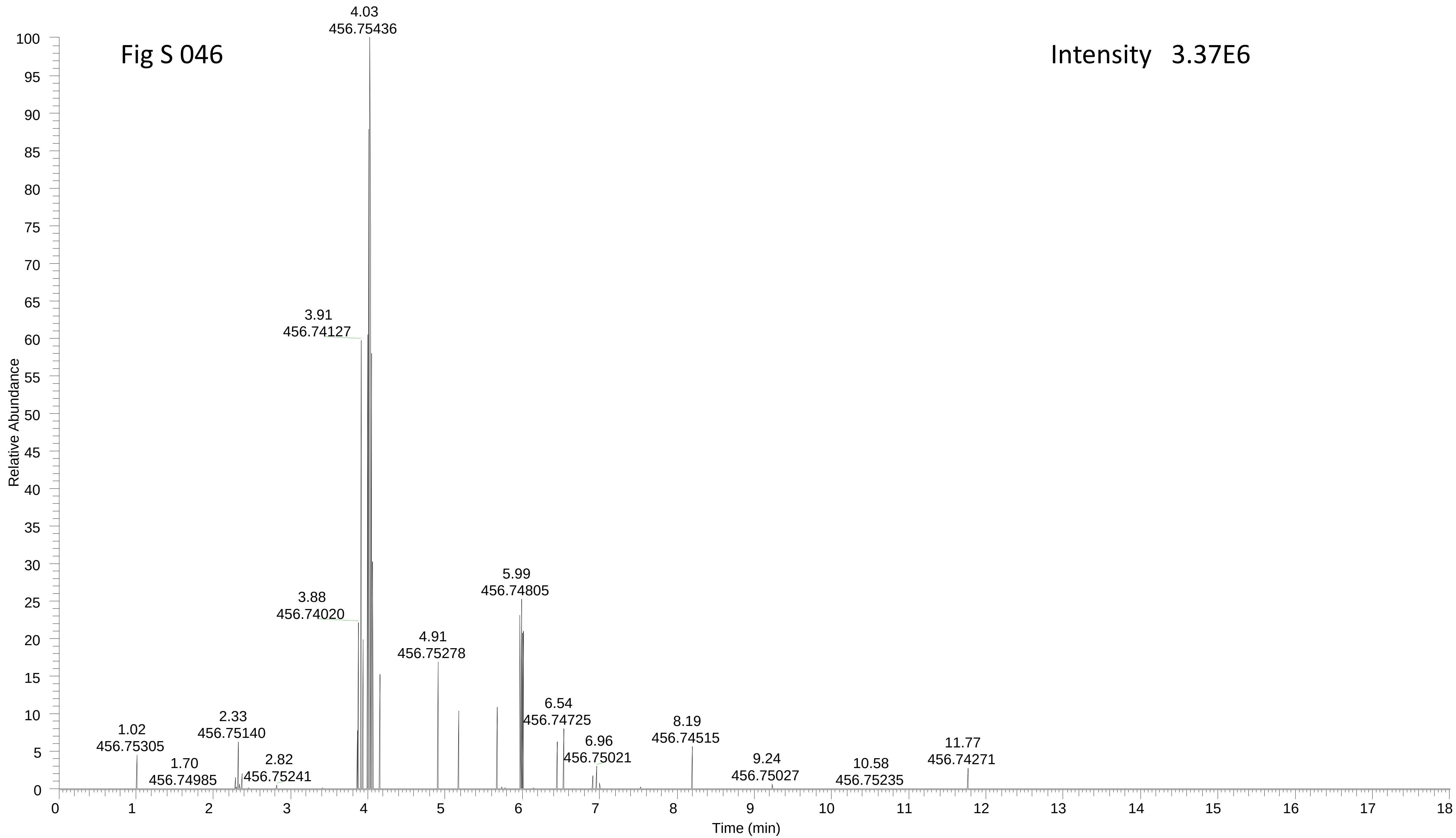
Fig S 046
Intensity 3.37E6
4.03
456.75436
3.91
456.74127
3.88
456.74020
4.91
456.75278
5.99
456.74805
6.54
456.74725
6.96
456.75021
8.19
456.74515
1.02
456.75305
1.70
456.74985
2.33
456.75140
2.82
456.75241
9.24
456.75027
10.58
456.75235
11.77
456.74271
Relative Abundance
Time (min)

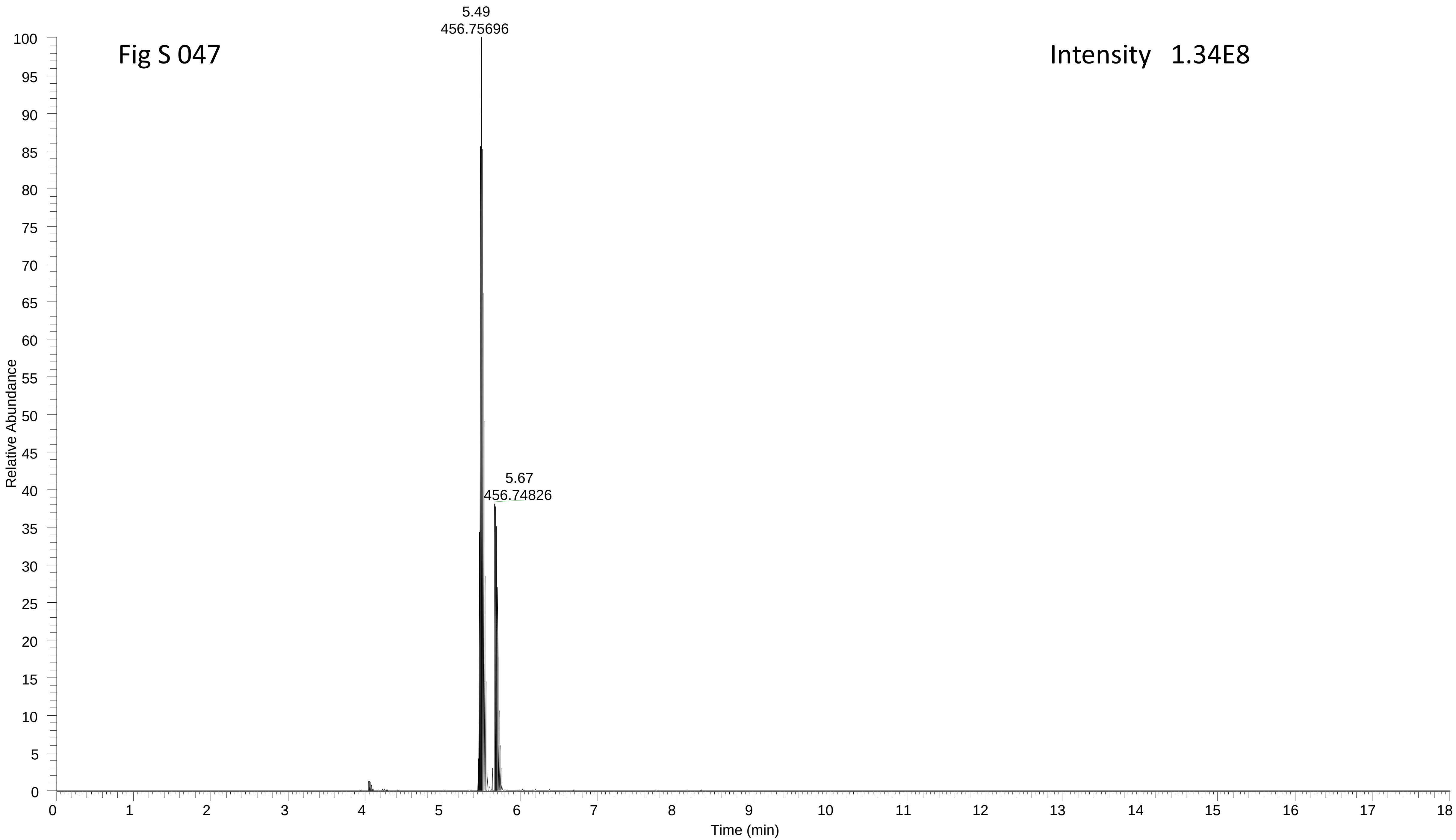
Fig S 047
Intensity 1.34E8
5.49
456.75696
5.67
456.74826
Relative Abundance
Time (min)
0
5
10
15
20
25
30
35
40
45
50
55
60
65
70
75
80
85
90
95
100
0
1
2
3
4
5
6
7
8
9
10
11
12
13
14
15
16
17
18

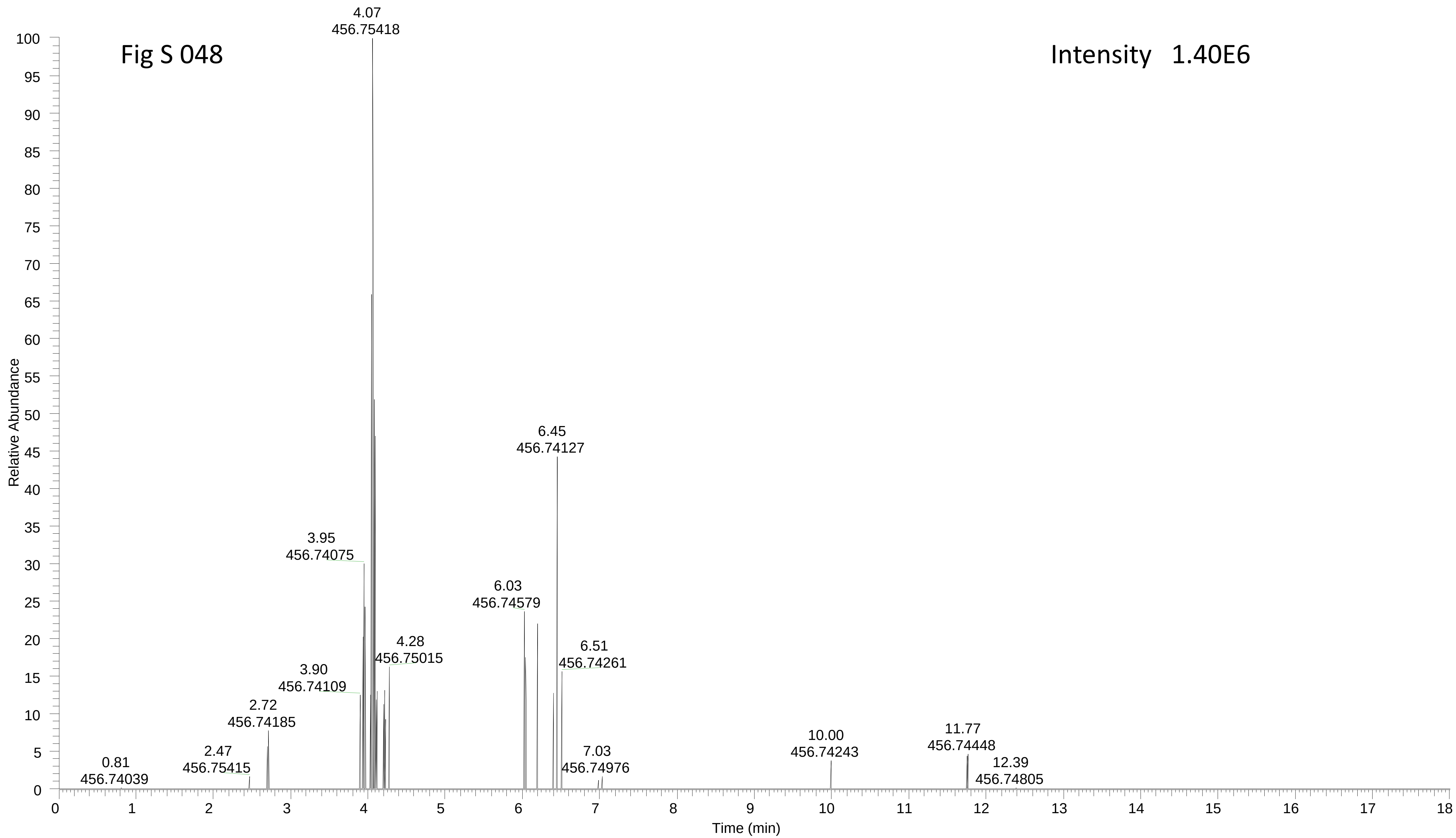
Fig S 048
Intensity 1.40E6
4.07
456.75418
6.45
456.74127
3.95
456.74075
6.03
456.74579
4.28
456.75015
6.51
456.74261
3.90
456.74109
2.72
456.74185
2.47
456.75415
0.81
456.74039
7.03
456.74976
10.00
456.74243
11.77
456.74448
12.39
456.74805
Relative Abundance
Time (min)

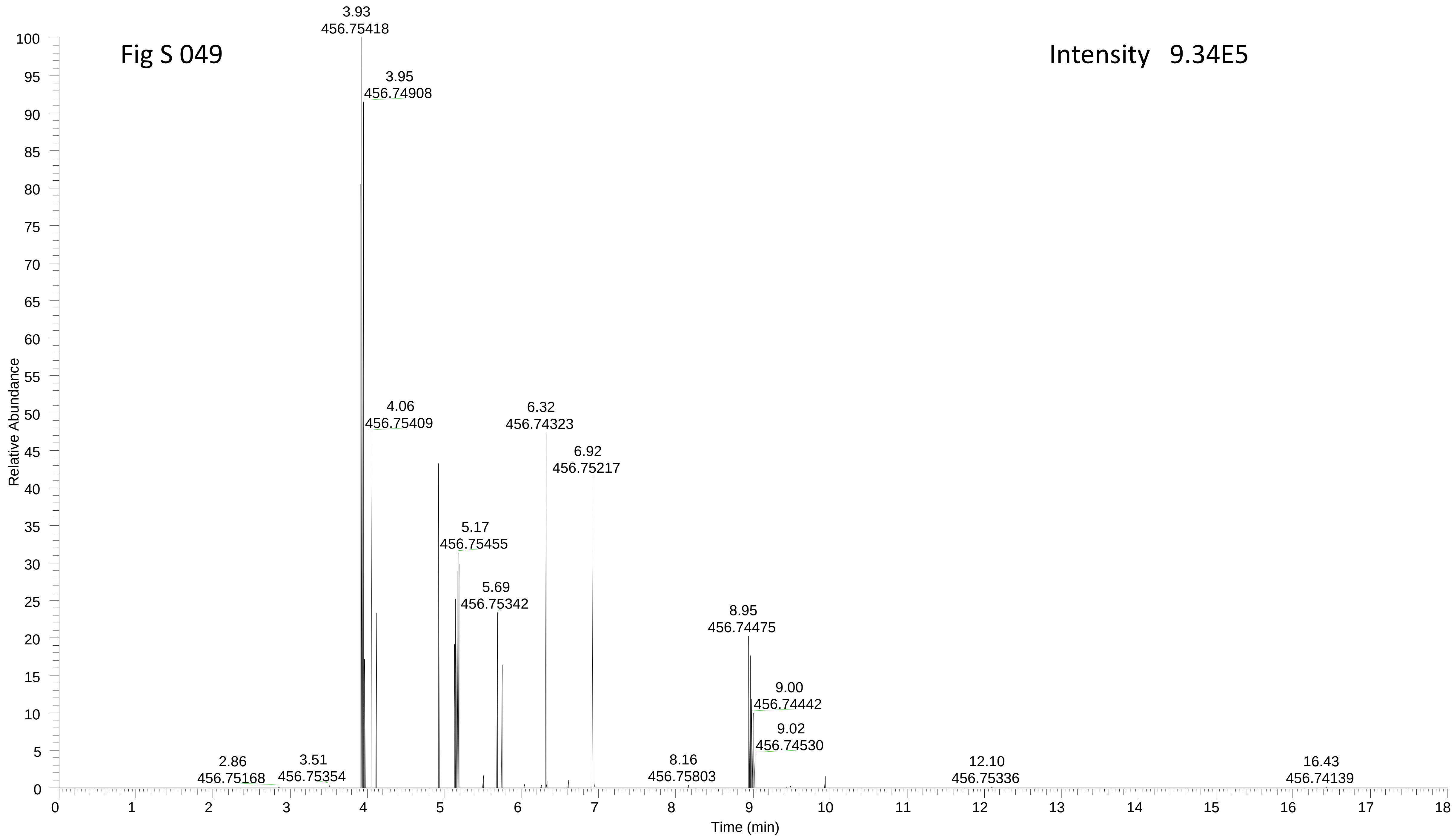
Fig S 049
Intensity 9.34E5
3.93
456.75418
3.95
456.74908
4.06
456.75409
6.32
456.74323
6.92
456.75217
5.17
456.75455
5.69
456.75342
8.95
456.74475
9.00
456.74442
9.02
456.74530
2.86
456.75168
3.51
456.75354
8.16
456.75803
12.10
456.75336
16.43
456.74139
Relative Abundance
Time (min)

Fig S 050

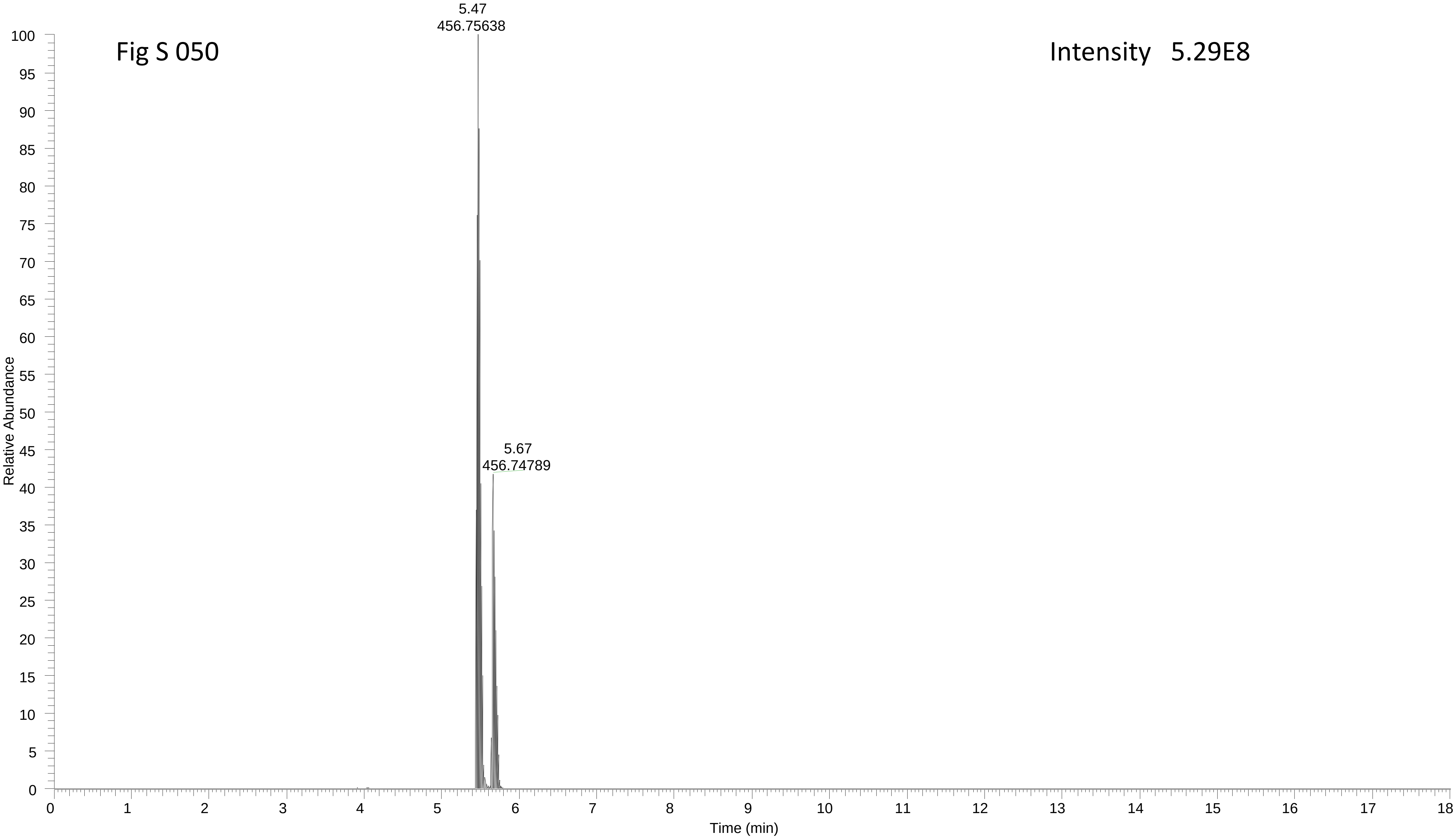
Intensity 5.29E8
5.47
456.75638
5.67
456.74789
Relative Abundance
Time (min)
0
5
10
15
20
25
30
35
40
45
50
55
60
65
70
75
80
85
90
95
100
0
1
2
3
4
5
6
7
8
9
10
11
12
13
14
15
16
17
18

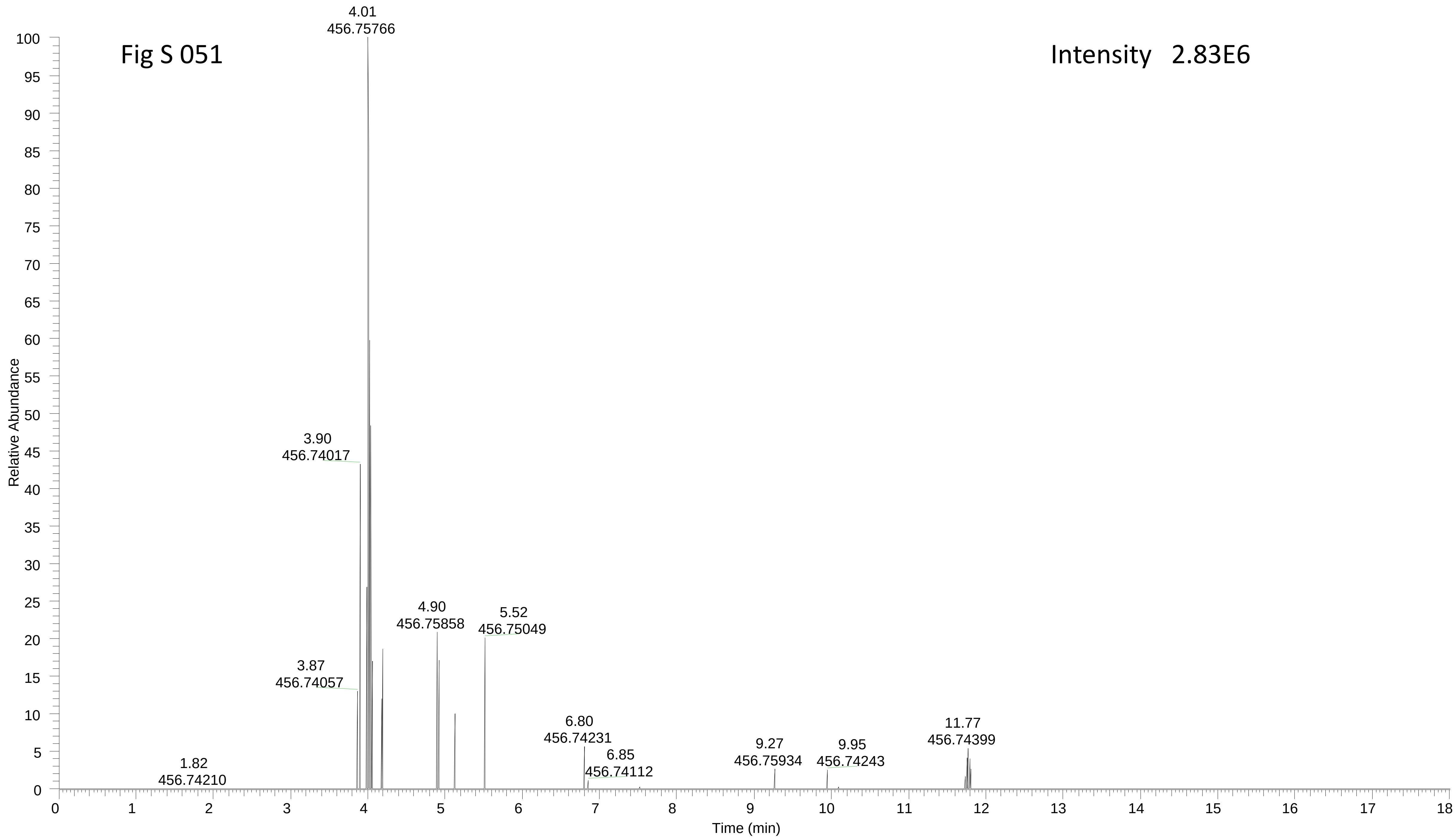
Fig S 051
Intensity 2.83E6
4.01
456.75766
3.90
456.74017
3.87
456.74057
4.90
456.75858
5.52
456.75049
1.82
456.74210
6.80
456.74231
6.85
456.74112
9.27
456.75934
9.95
456.74243
11.77
456.74399
Relative Abundance
Time (min)

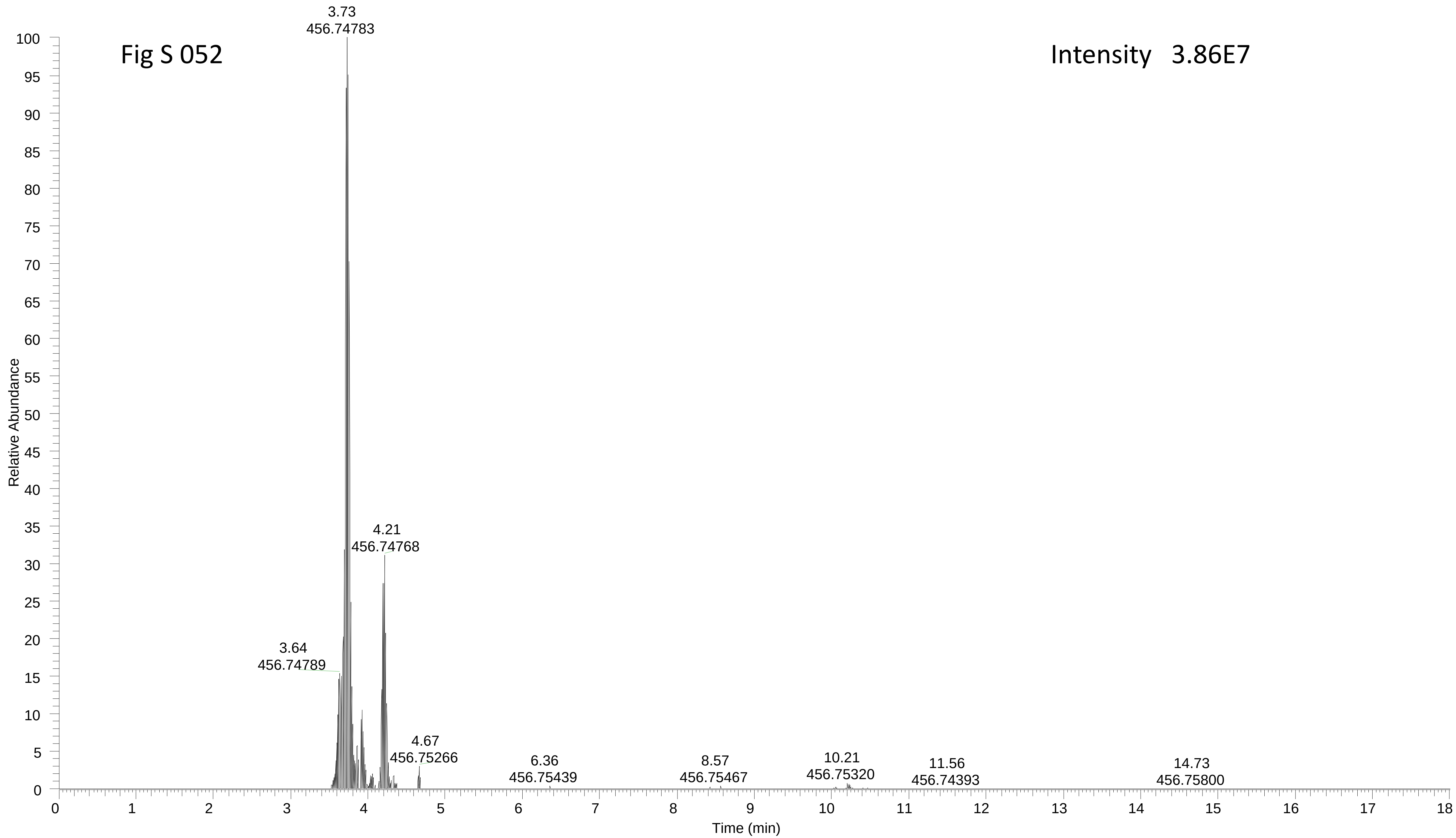

Fig S 052
Intensity 3.86E7
3.73
456.74783
4.21
456.74768
3.64
456.74789
4.67
456.75266
6.36
456.75439
8.57
456.75467
10.21
456.75320
11.56
456.74393
14.73
456.75800
Relative Abundance
Time (min)

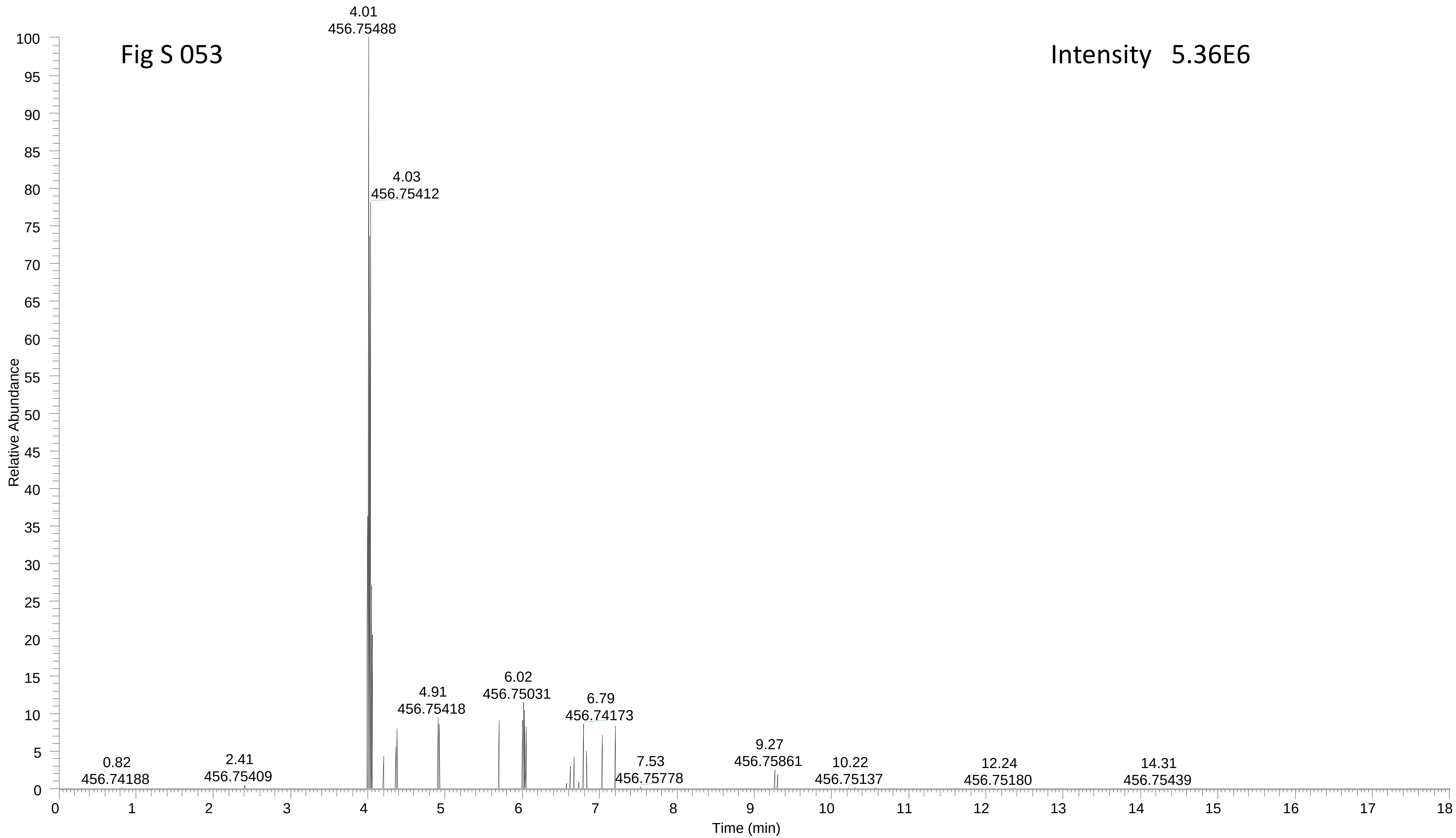
Fig S 053
Intensity 5.36E6
4.01
456.75488
4.03
456.75412
4.91
456.75418
6.02
456.75031
6.79
456.74173
7.53
456.75778
9.27
456.75861
10.22
456.75137
12.24
456.75180
14.31
456.75439
0.82
456.74188
2.41
456.75409
Relative Abundance
Time (min)

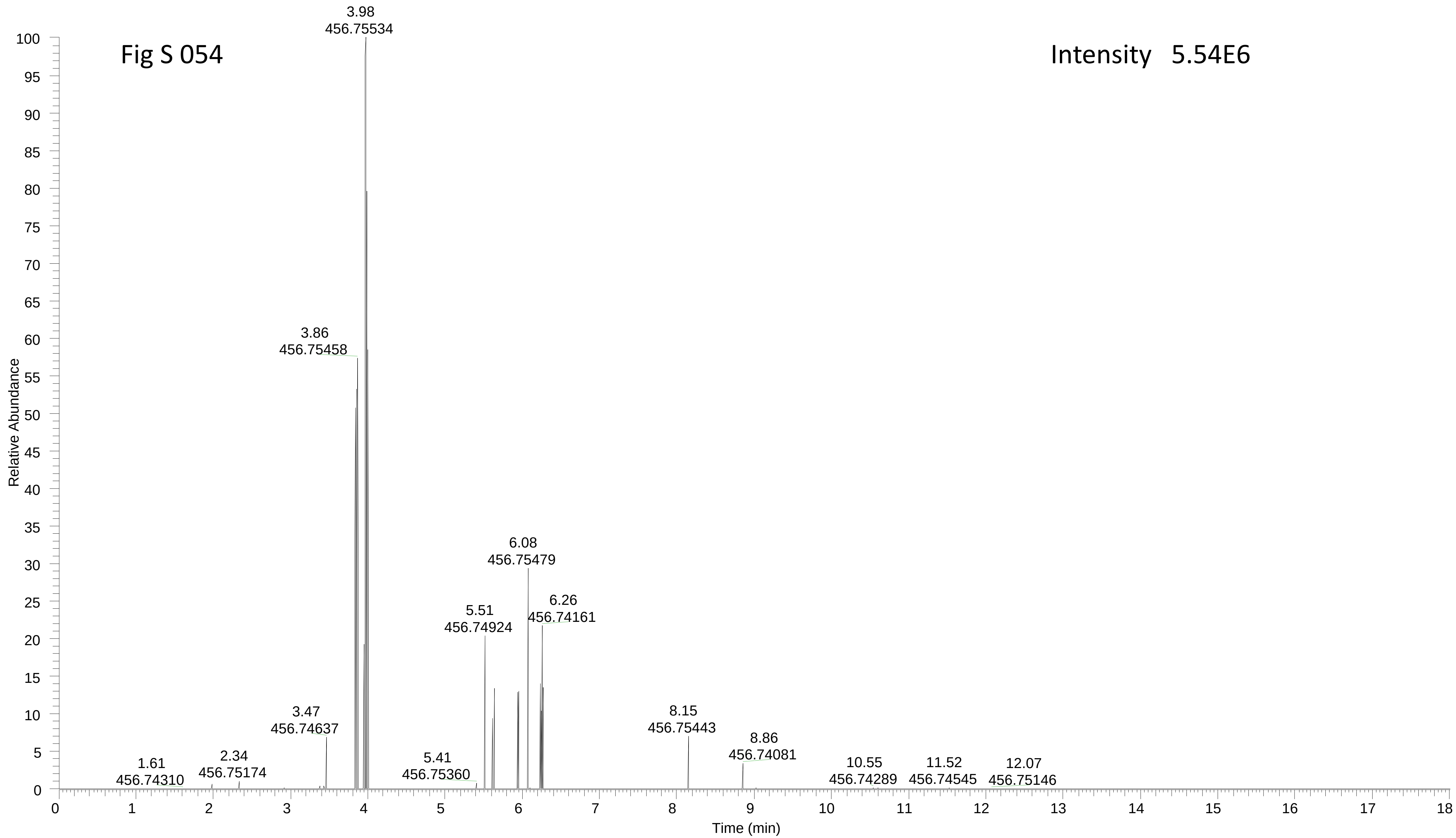

Fig S 054
Intensity 5.54E6
3.98
456.75534
3.86
456.75458
6.08
456.75479
6.26
456.74161
5.51
456.74924
3.47
456.74637
8.15
456.75443
8.86
456.74081
1.61
456.74310
2.34
456.75174
5.41
456.75360
10.55
456.74289
11.52
456.74545
12.07
456.75146
Relative Abundance
Time (min)

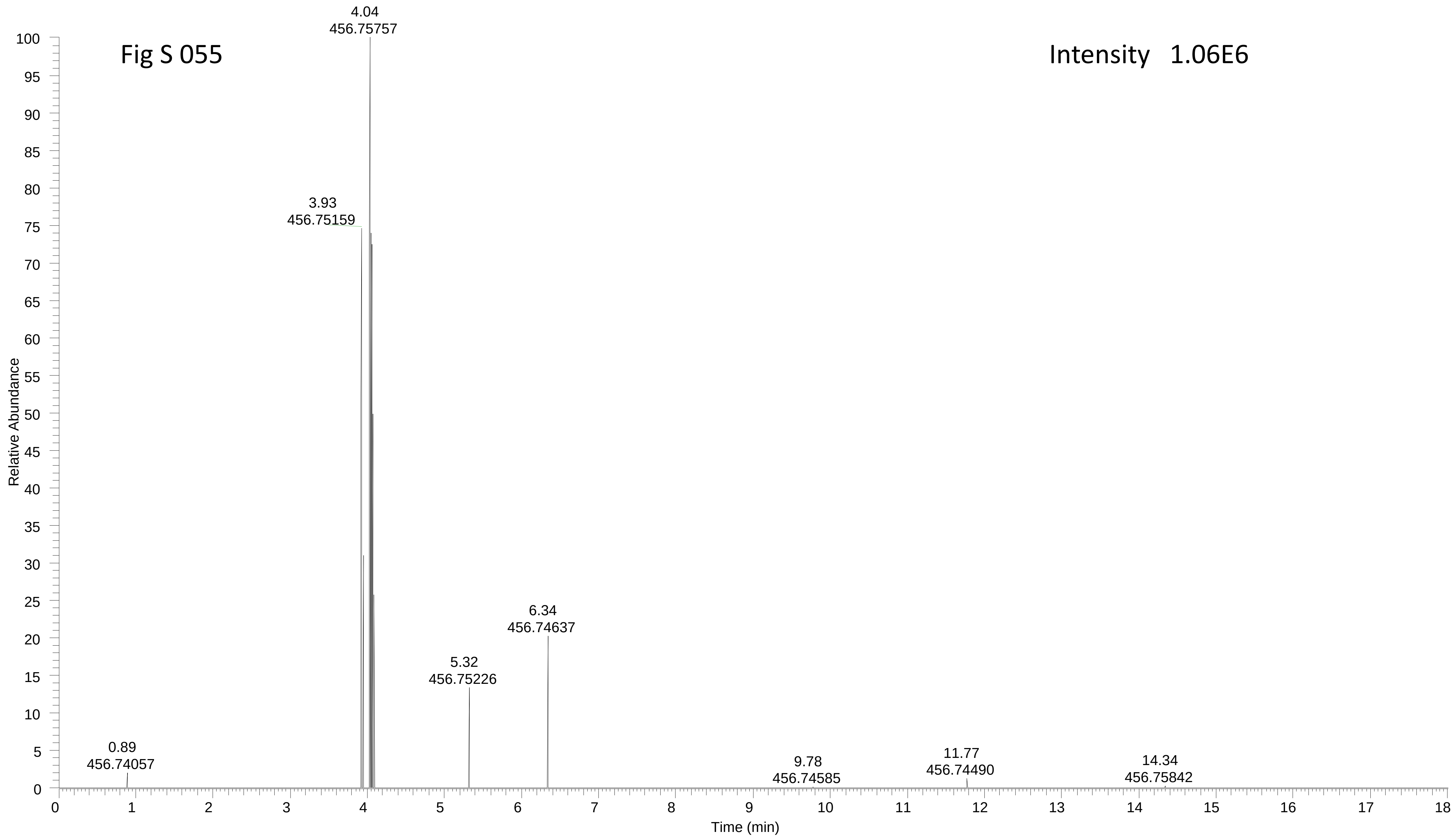

Fig S 055
Intensity 1.06E6
4.04
456.75757
3.93
456.75159
6.34
456.74637
5.32
456.75226
0.89
456.74057
9.78
456.74585
11.77
456.74490
14.34
456.75842
Relative Abundance
Time (min)

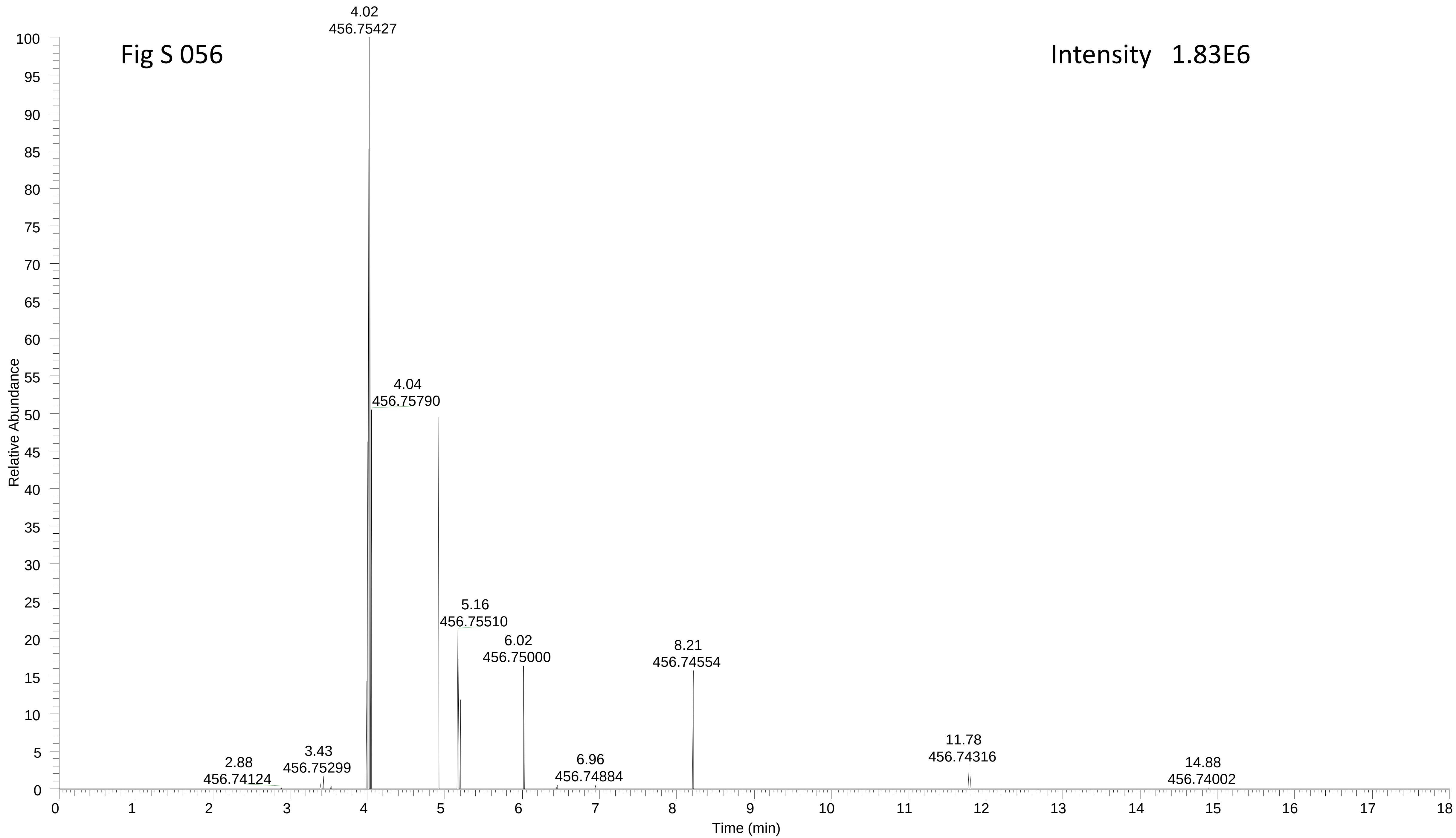

Fig S 056
Intensity 1.83E6
4.02
456.75427
4.04
456.75790
5.16
456.75510
6.02
456.75000
8.21
456.74554
2.88
456.74124
3.43
456.75299
6.96
456.74884
11.78
456.74316
14.88
456.74002
Relative Abundance
Time (min)

S2 File. Chromatograms and MS/MS spectra.

Raw data MS/MS spectra VGPIGAAGNR

Fig S 057: pheasant meat strip
Fig S 058: pheasant leg

Remarks:
-Precursor m/z ≈ 456.26
-Only pheasant samples provided MS/MS spectra for this peptide.
-Data recorded in June 2020.
-See Fig 4b in the main document for peak annotation.

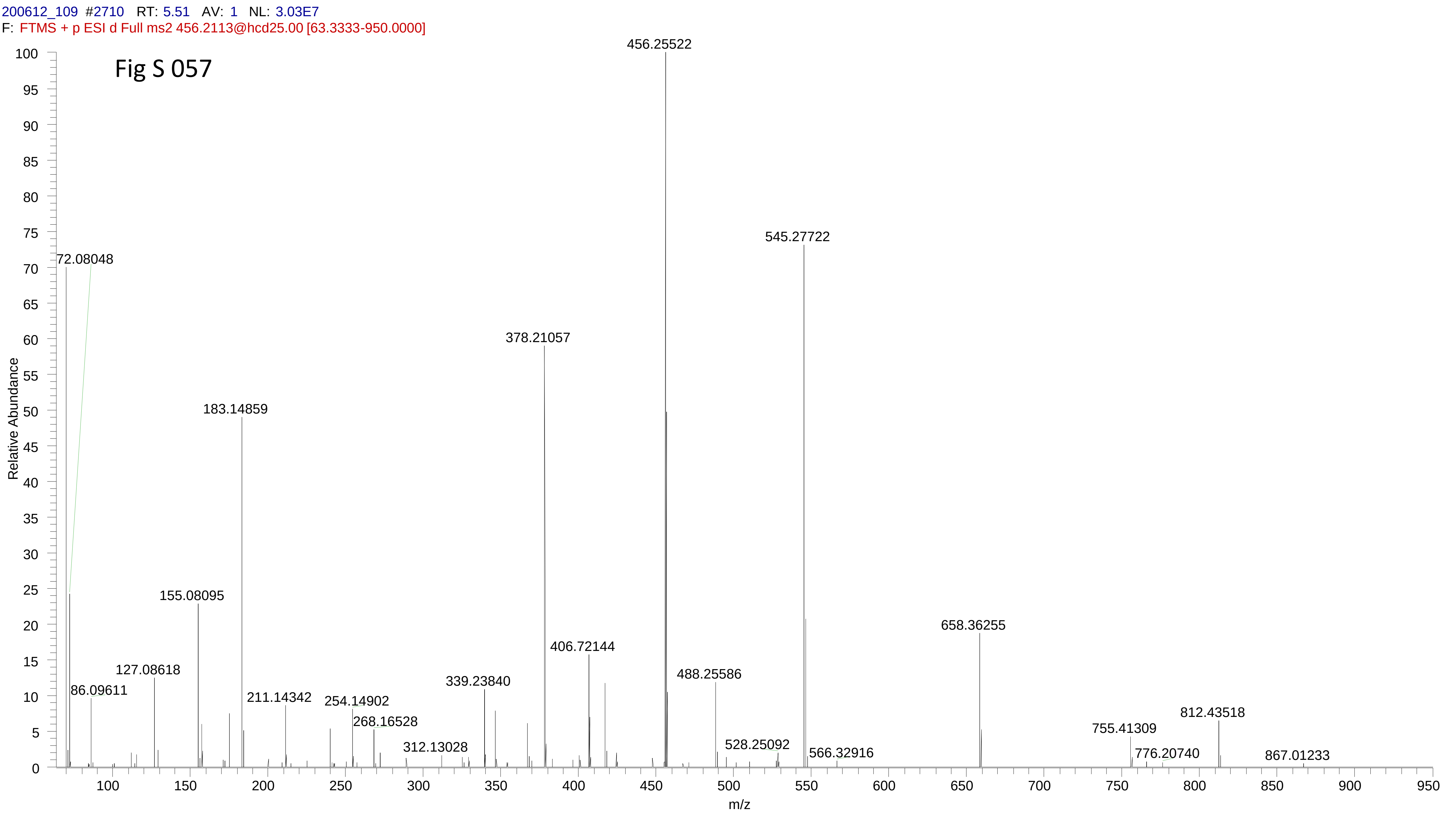
200612_109 #2710 RT: 5.51 AV: 1 NL: 3.03E7
F: FTMS + p ESI d Full ms2 456.2113@hcd25.00 [63.3333-950.0000]
Fig S 057
Relative Abundance
m/z
456.25522
545.27722
72.08048
378.21057
183.14859
155.08095
658.36255
406.72144
127.08618
488.25586
86.09611
339.23840
211.14342
254.14902
268.16528
812.43518
755.41309
528.25092
312.13028
566.32916
776.20740
867.01233

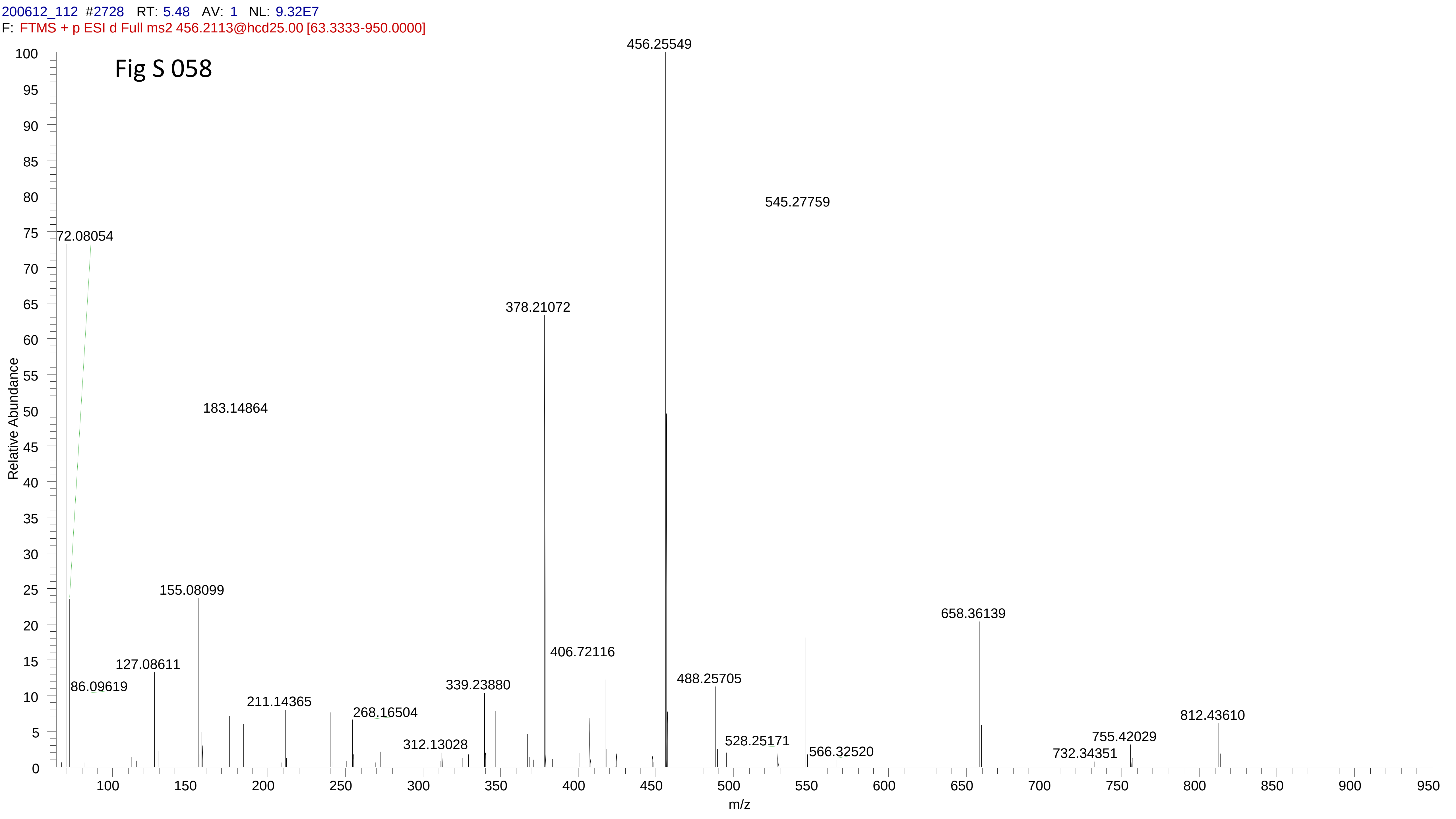
200612_112 #2728 RT: 5.48 AV: 1 NL: 9.32E7
F: FTMS + p ESI d Full ms2 456.2113@hcd25.00 [63.3333-950.0000]
Fig S 058
Relative Abundance
m/z
456.25549
545.27759
72.08054
378.21072
183.14864
155.08099
658.36139
127.08611
406.72116
86.09619
488.25705
339.23880
211.14365
268.16504
812.43610
312.13028
528.25171
755.42029
566.32520
732.34351

S2 File. Chromatograms and MS/MS spectra.

Raw data MS/MS spectra deamidated VGPIGAAGNR

Fig S 059: pheasant meat strip
Fig S 060: pheasant leg

Remarks:
-Precursor m/z ≈ 456.75
-Only pheasant samples provided MS/MS spectra for this peptide.
-Data recorded in June 2020.
-See Fig 4b in the main document for peak annotation, corrected for the presence of deamidated N9 (+ 0.984 Da).

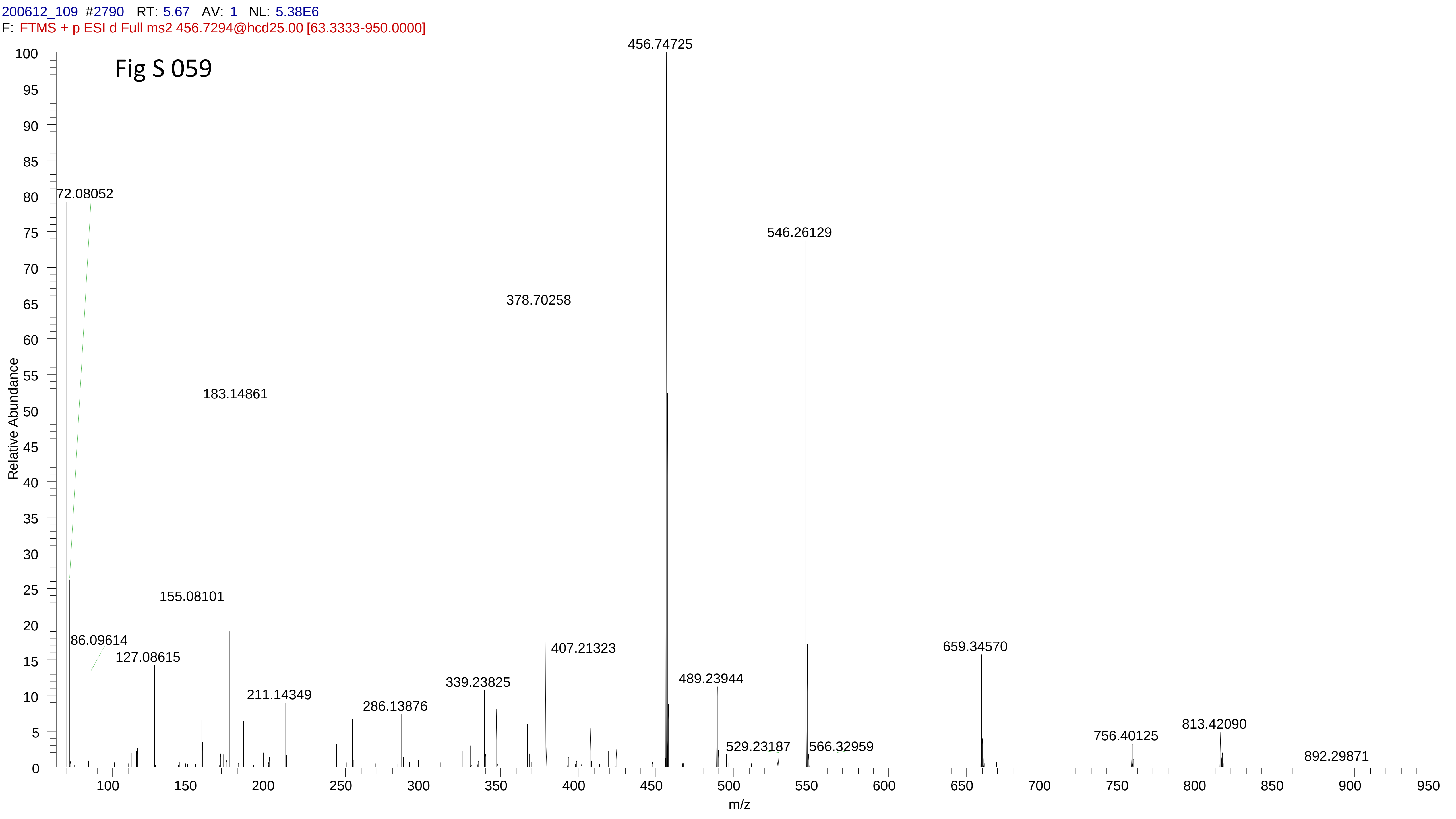
200612_109 #2790 RT: 5.67 AV: 1 NL: 5.38E6
F: FTMS + p ESI d Full ms2 456.7294@hcd25.00 [63.3333-950.0000]
Fig S 059
456.74725
72.08052
546.26129
378.70258
183.14861
155.08101
86.09614
127.08615
407.21323
659.34570
489.23944
339.23825
211.14349
286.13876
813.42090
756.40125
529.23187
566.32959
892.29871
Relative Abundance
m/z

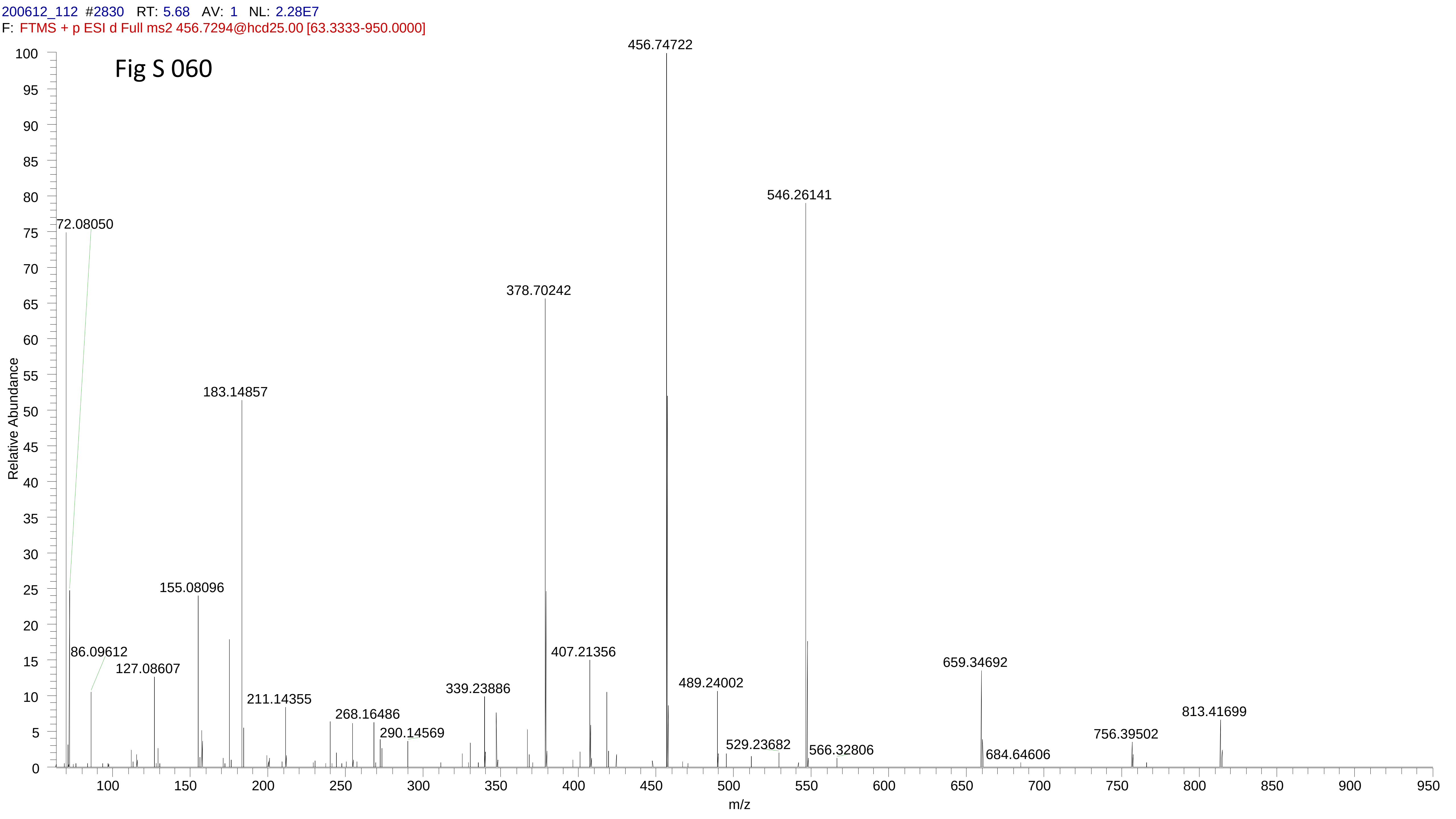
200612_112 #2830 RT: 5.68 AV: 1 NL: 2.28E7
F: FTMS + p ESI d Full ms2 456.7294@hcd25.00 [63.3333-950.0000]
Fig S 060
Relative Abundance
m/z
72.08050
86.09612
127.08607
155.08096
183.14857
211.14355
268.16486
290.14569
339.23886
378.70242
407.21356
456.74722
489.24002
529.23682
546.26141
566.32806
659.34692
684.64606
756.39502
813.41699

S2 File. Chromatograms and MS/MS spectra.

Raw data chromatograms EGPVGF**p**GADGR

Fig S 061: ostrich tendon
Fig S 062: goose neck
Fig S 063: duck neck
Fig S 064: turkey neck
Fig S 065: chicken leg
Fig S 066: pheasant meat strip
Fig S 067: goose meat strip
Fig S 068: goose leg
Fig S 069: pheasant leg
Fig S 070: guinea fowl torso
Fig S 071: pigeon torso
Fig S 072: partridge torso
Fig S 073: duck leg
Fig S 074: quail leg
Fig S 075: turkey leg

Remarks:
-Extracted m/z range 587.775-587.785
-The retention time and m/z of the base peak are provided per peak and the provided intensity is of the highest peak.
-Data recorded in June 2020.

Fig S 061

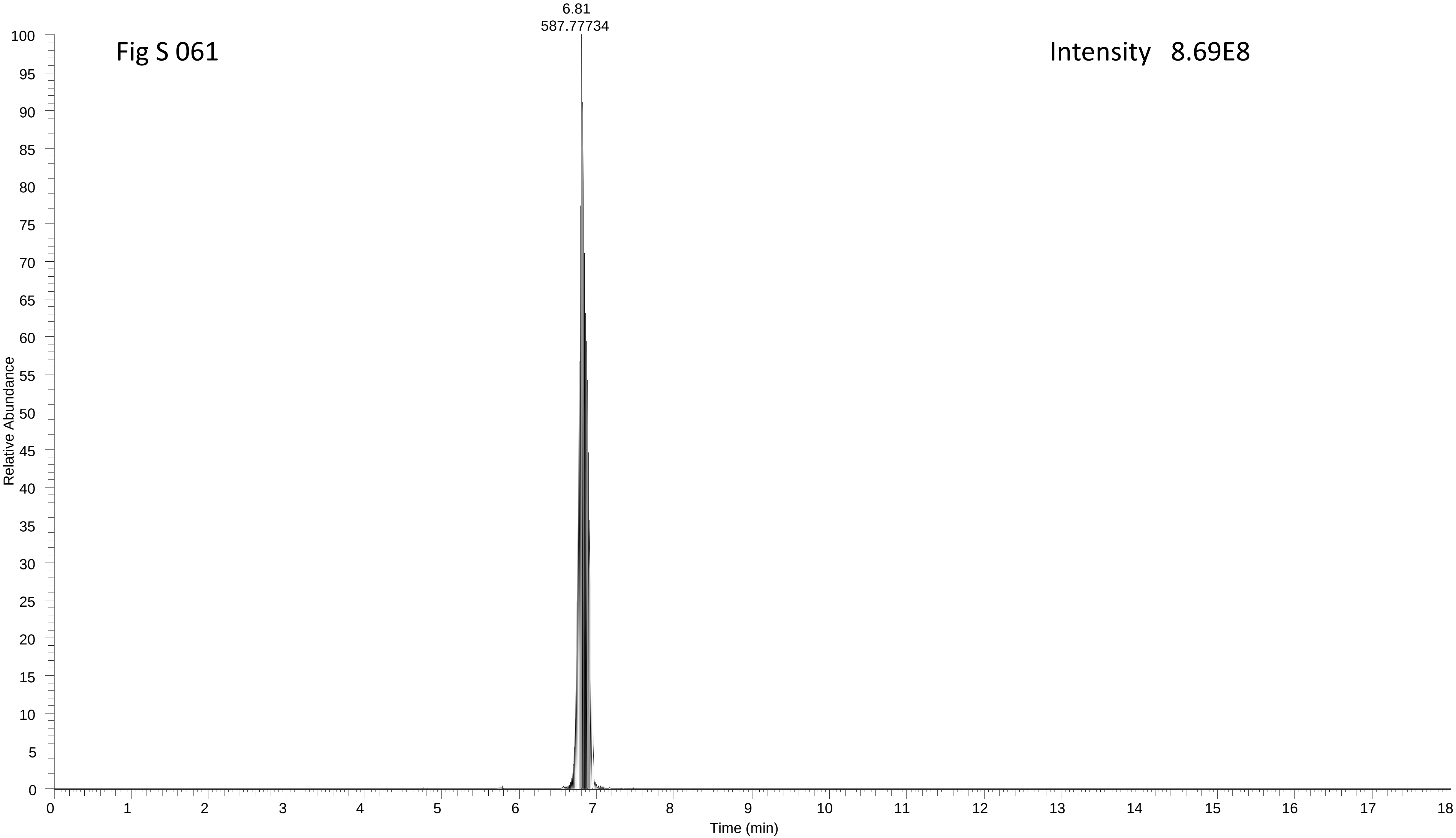
6.81
587.77734
Intensity 8.69E8
Relative Abundance
Time (min)
0 5 10 15 20 25 30 35 40 45 50 55 60 65 70 75 80 85 90 95 100
0 1 2 3 4 5 6 7 8 9 10 11 12 13 14 15 16 17 18

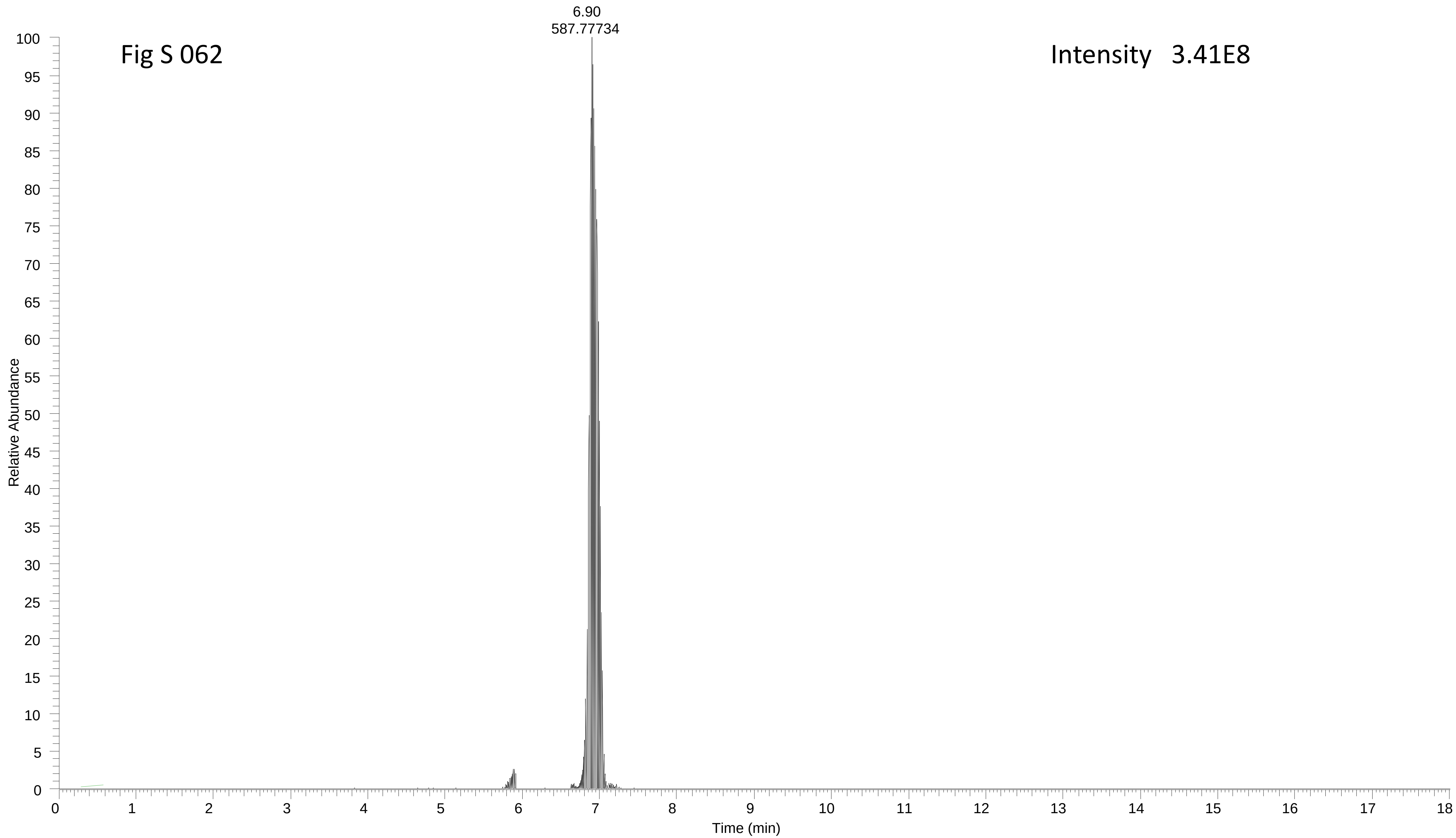
Fig S 062
Intensity 3.41E8
6.90
587.77734
Relative Abundance
Time (min)
0
5
10
15
20
25
30
35
40
45
50
55
60
65
70
75
80
85
90
95
100
0
1
2
3
4
5
6
7
8
9
10
11
12
13
14
15
16
17
18

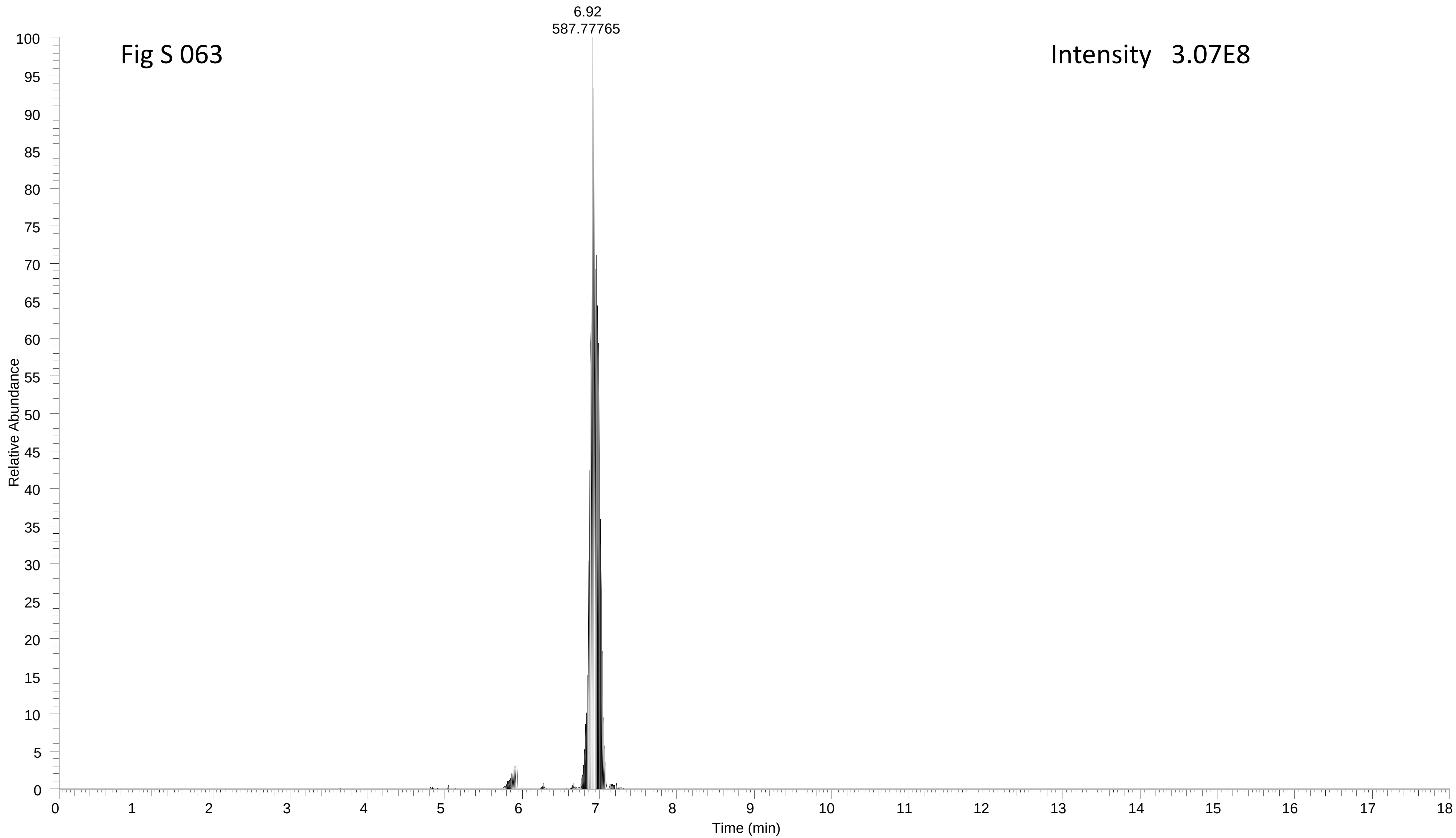
Fig S 063
Intensity 3.07E8
6.92
587.77765
Relative Abundance
Time (min)
0
5
10
15
20
25
30
35
40
45
50
55
60
65
70
75
80
85
90
95
100
0
1
2
3
4
5
6
7
8
9
10
11
12
13
14
15
16
17
18

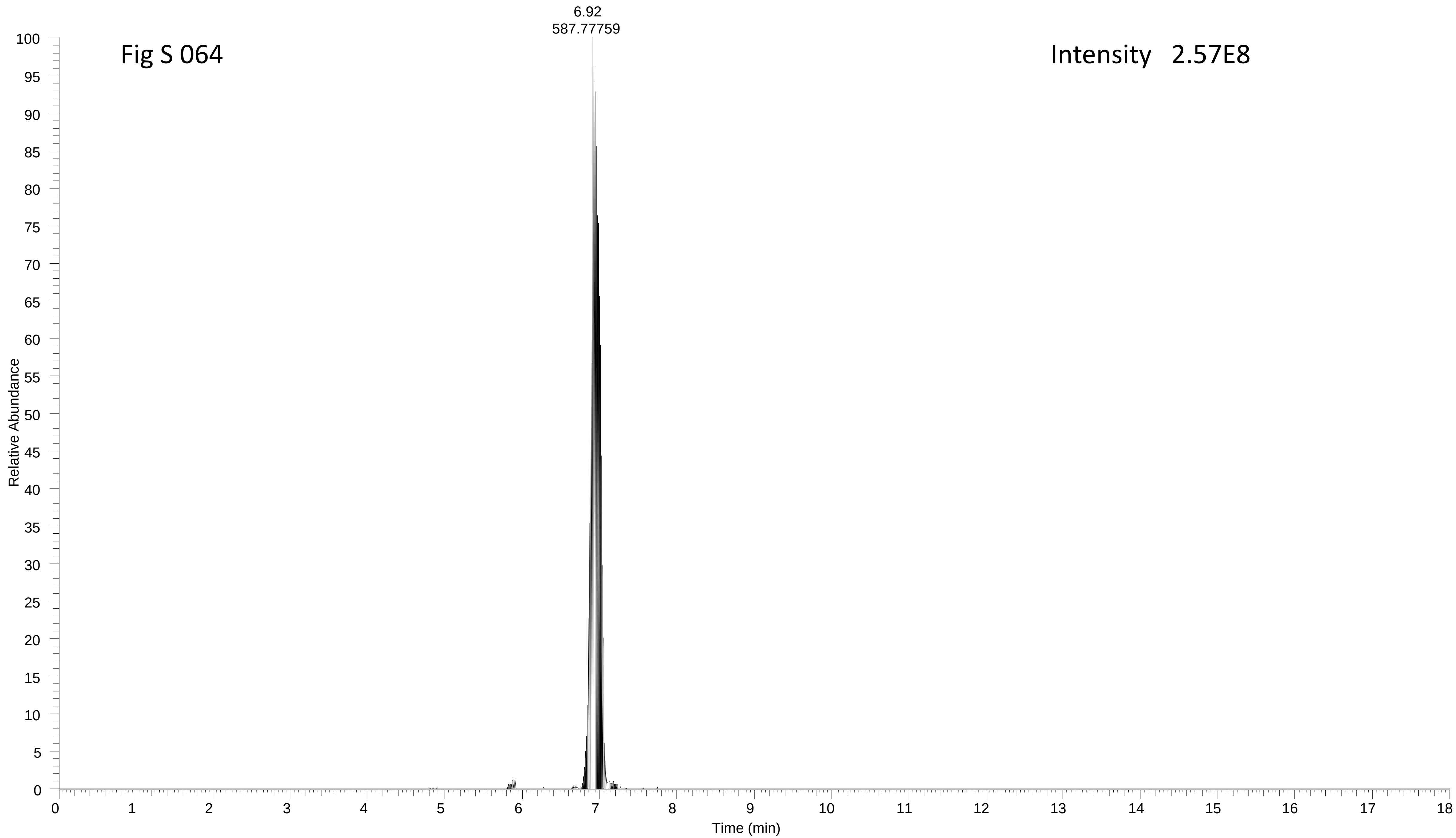
Fig S 064
Intensity 2.57E8
6.92
587.77759
Relative Abundance
Time (min)

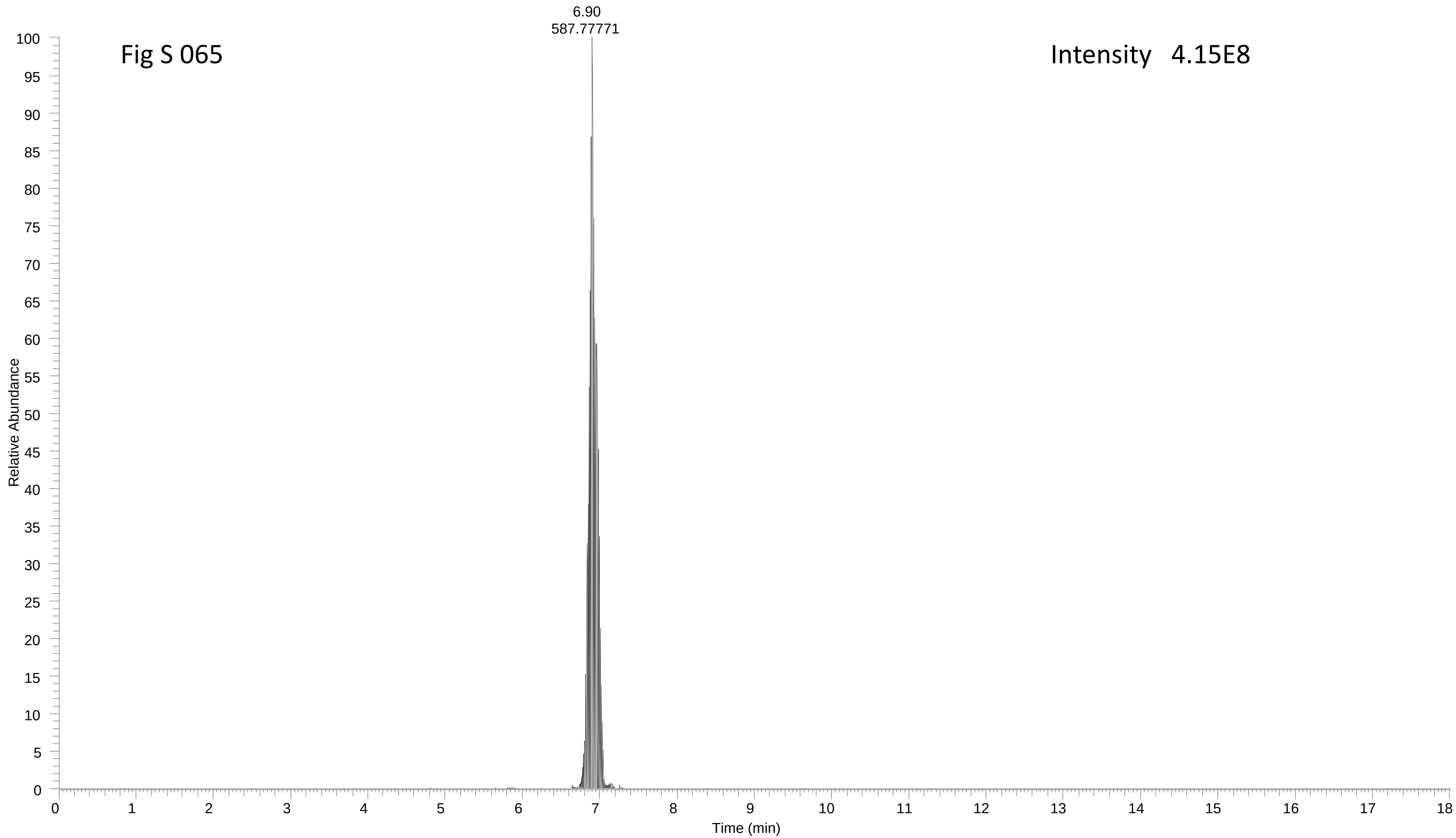
Fig S 065
Intensity 4.15E8
6.90
587.77771
Relative Abundance
Time (min)
0
5
10
15
20
25
30
35
40
45
50
55
60
65
70
75
80
85
90
95
100
1
2
3
4
5
6
7
8
9
10
11
12
13
14
15
16
17
18

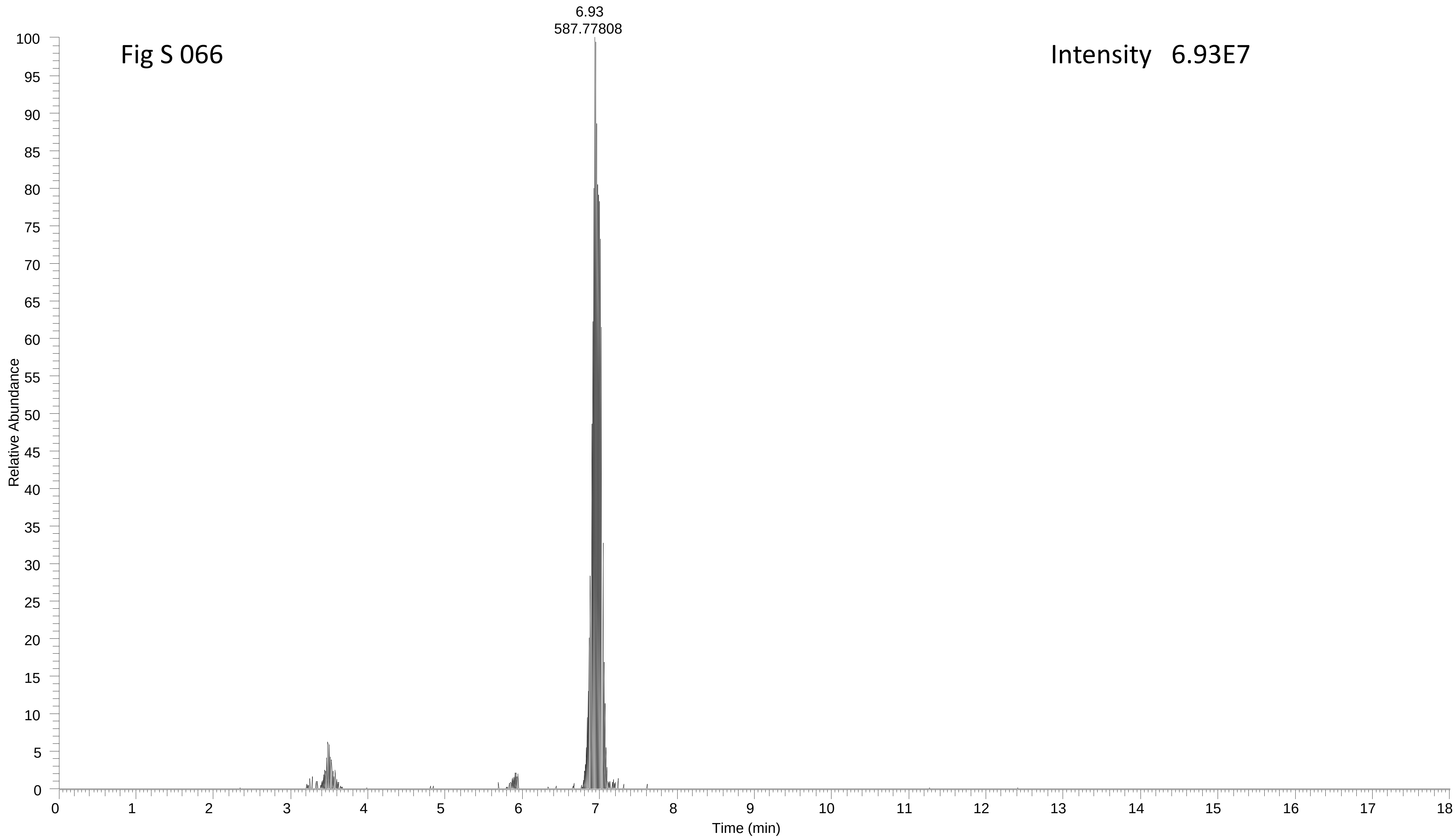

Fig S 066
Intensity 6.93E7
6.93
587.77808
Relative Abundance
Time (min)
0
5
10
15
20
25
30
35
40
45
50
55
60
65
70
75
80
85
90
95
100
1
2
3
4
5
6
7
8
9
10
11
12
13
14
15
16
17
18

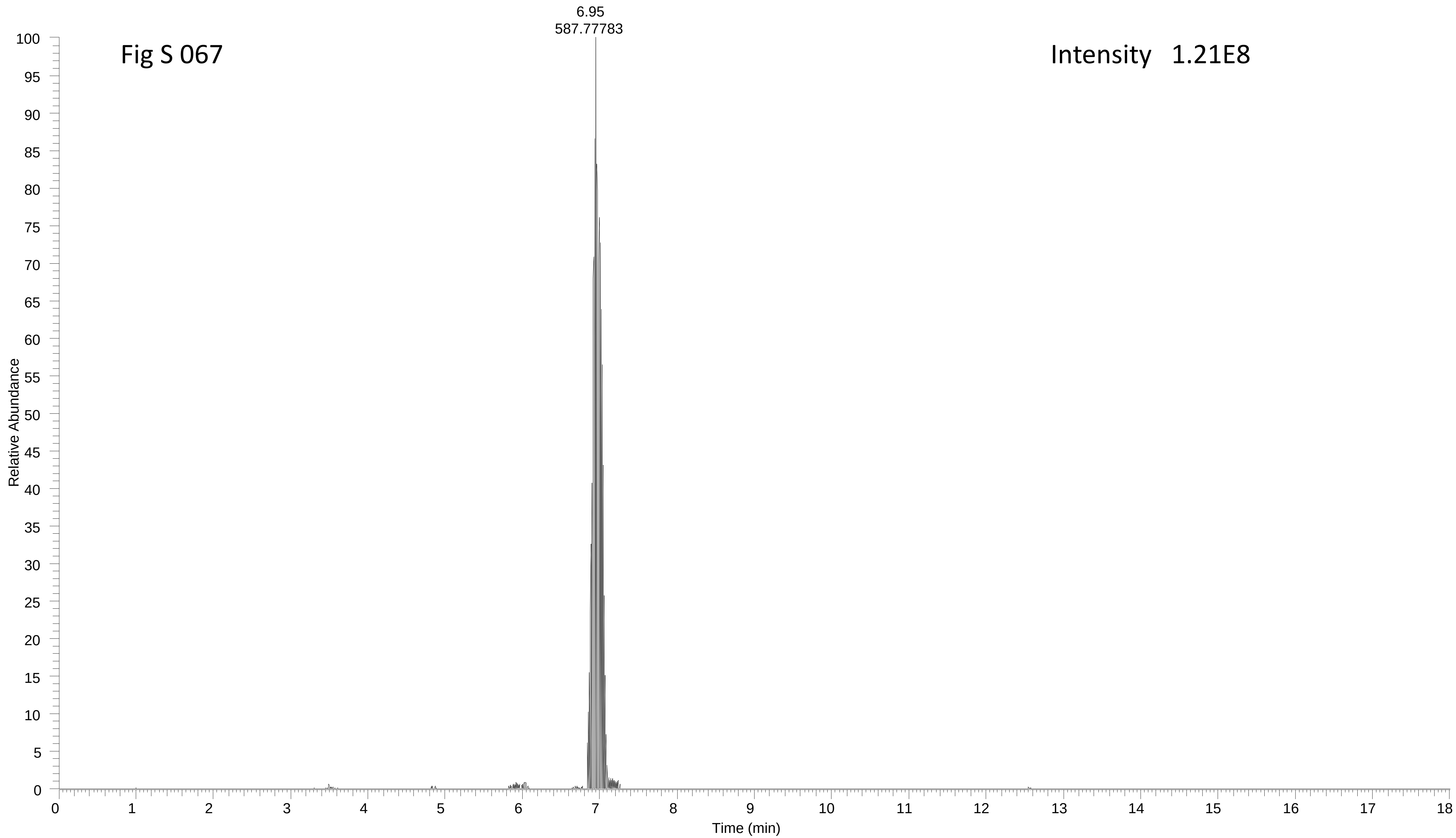
Fig S 067
Intensity 1.21E8
6.95
587.77783
Relative Abundance
Time (min)
0
5
10
15
20
25
30
35
40
45
50
55
60
65
70
75
80
85
90
95
100
0
1
2
3
4
5
6
7
8
9
10
11
12
13
14
15
16
17
18

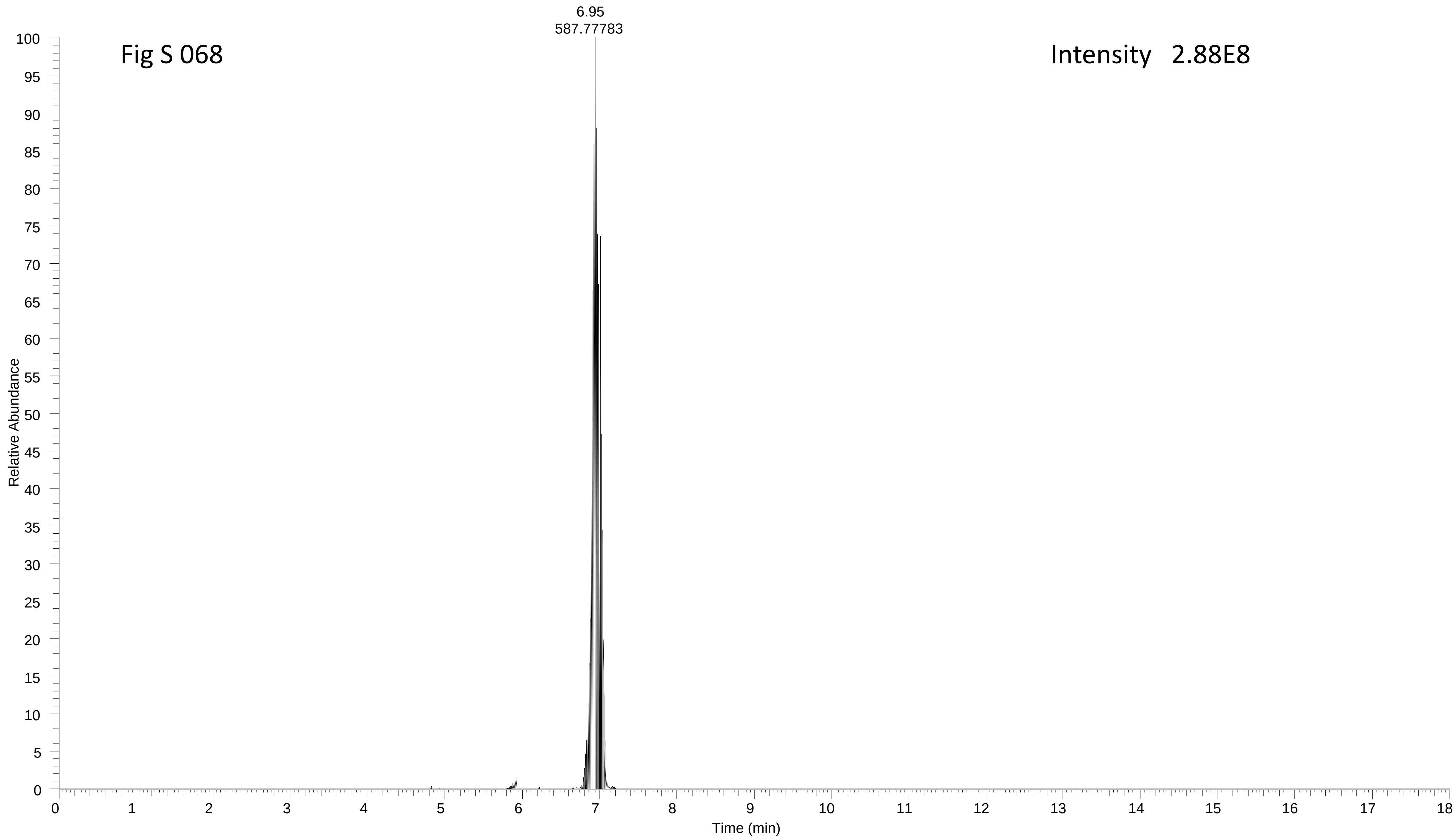
6.95
587.77783
Fig S 068
Intensity 2.88E8
Relative Abundance
Time (min)

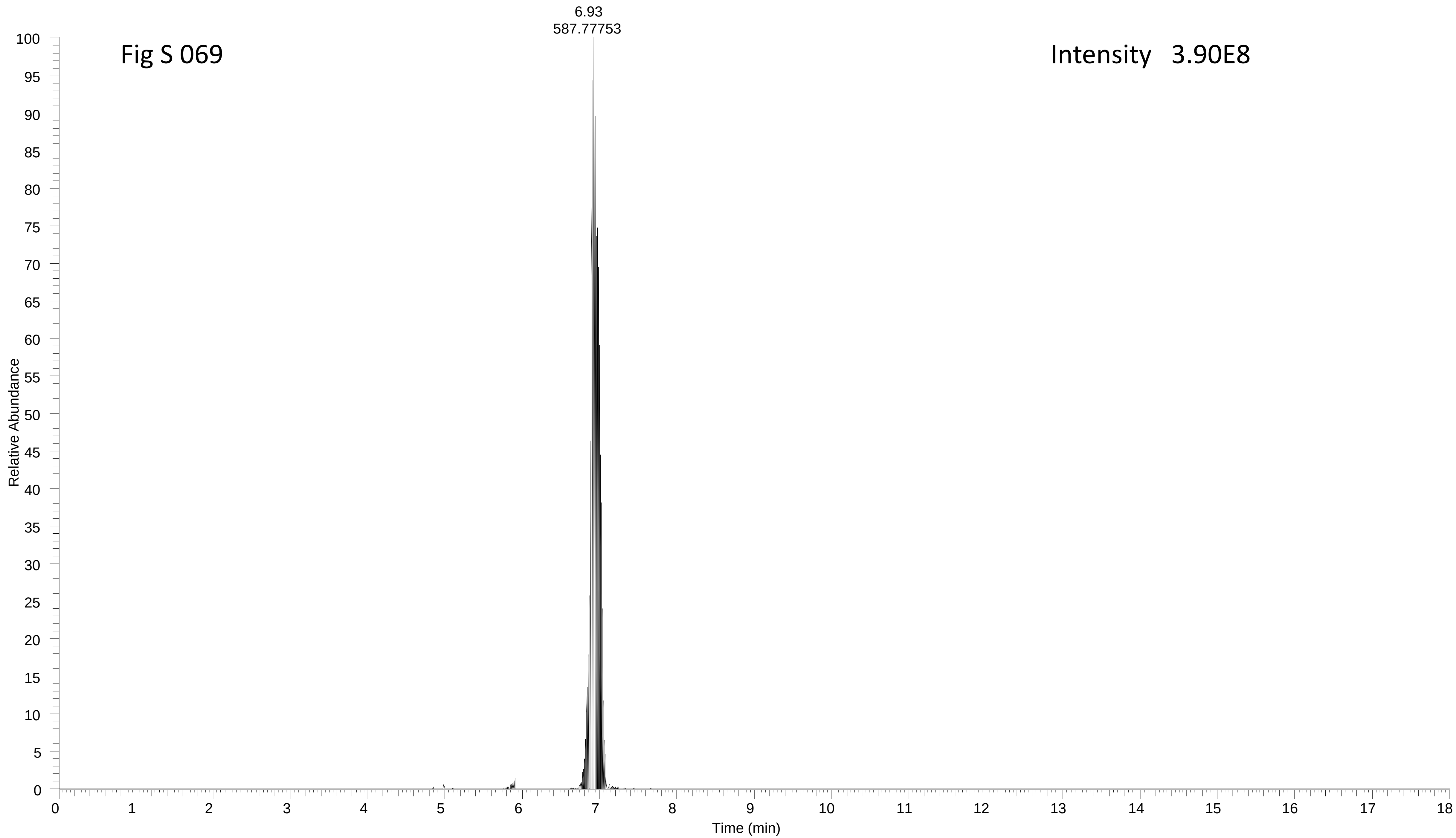
Fig S 069
Intensity 3.90E8
6.93
587.77753
Relative Abundance
Time (min)

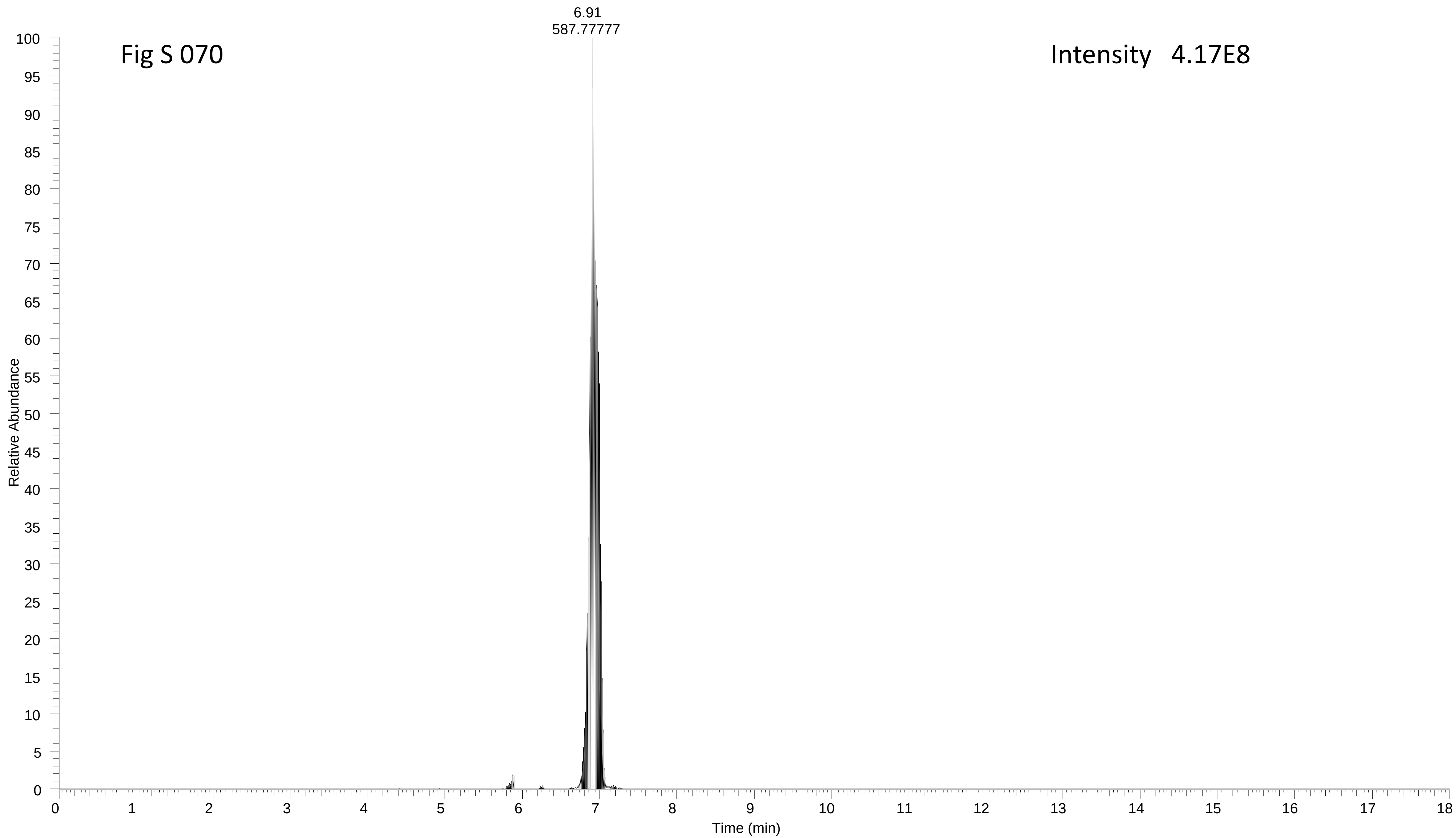
Fig S 070
Intensity 4.17E8
6.91
587.77777
Relative Abundance
Time (min)

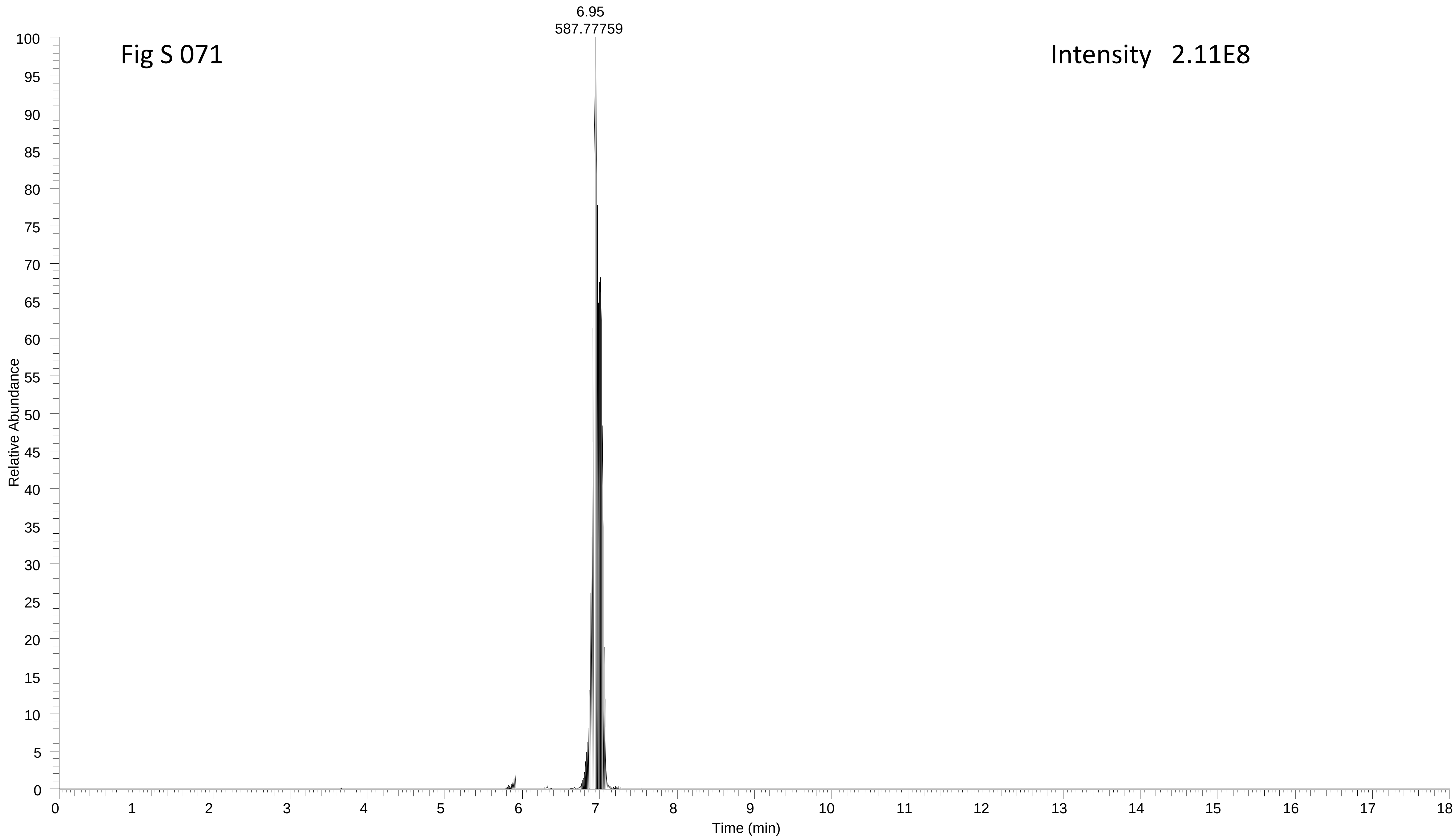

6.95
587.77759
Fig S 071
Intensity 2.11E8
Relative Abundance
Time (min)

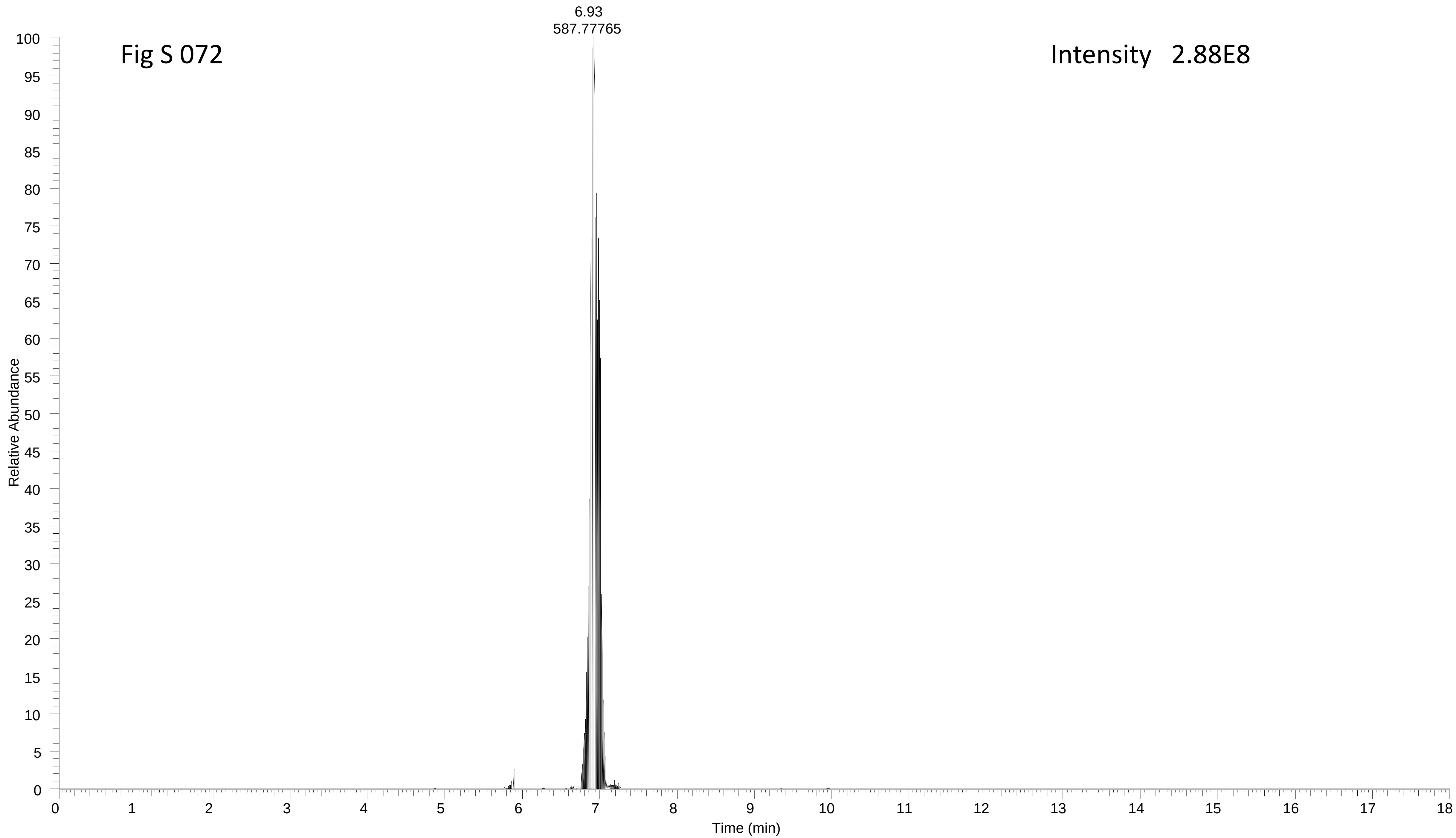

Fig S 072
Intensity 2.88E8
6.93
587.77765
Relative Abundance
Time (min)
0
5
10
15
20
25
30
35
40
45
50
55
60
65
70
75
80
85
90
95
100
1
2
3
4
6
7
8
9
11
12
13
14
16
17
18

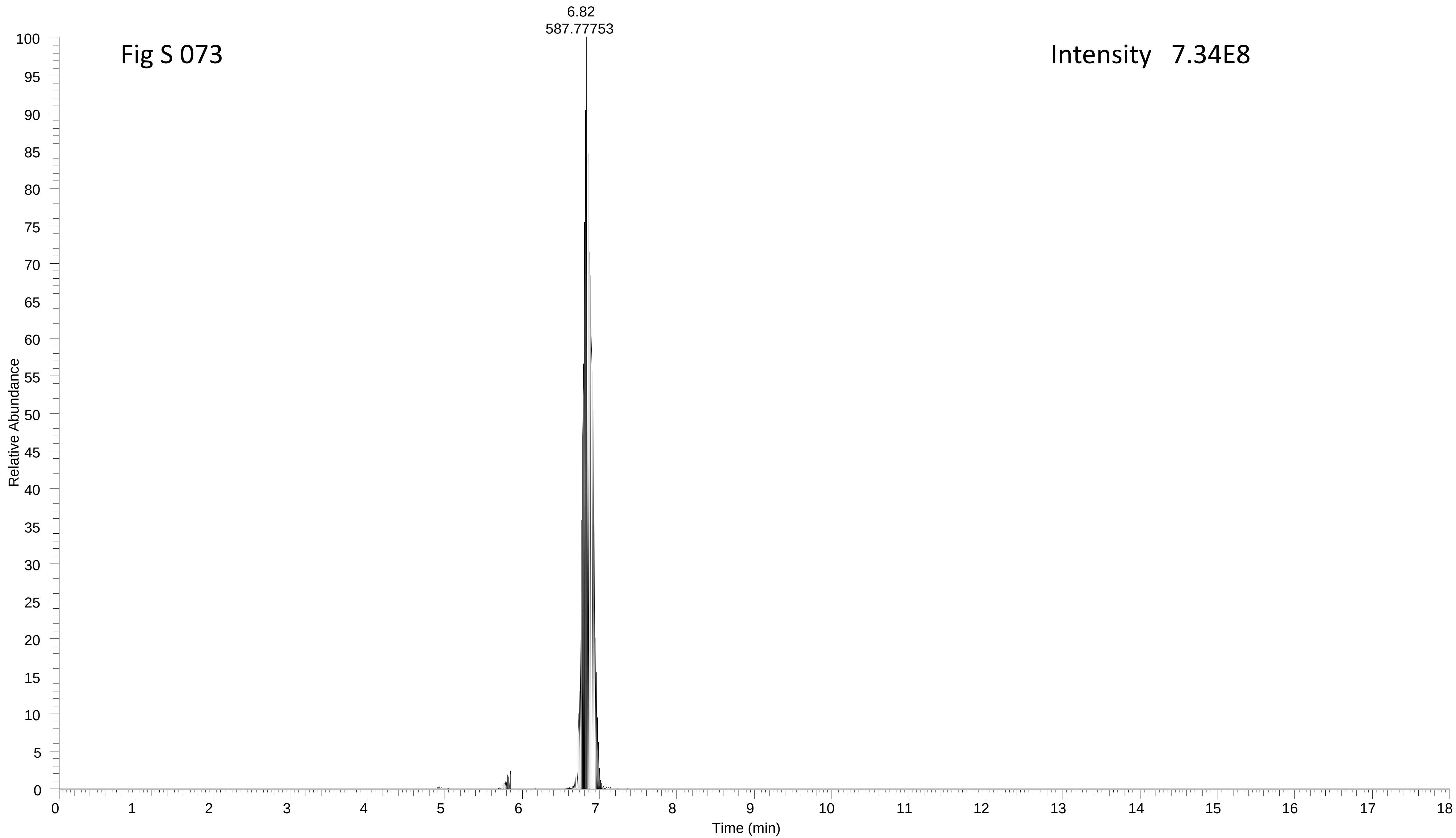
Fig S 073
Intensity 7.34E8
6.82
587.77753
Relative Abundance
Time (min)

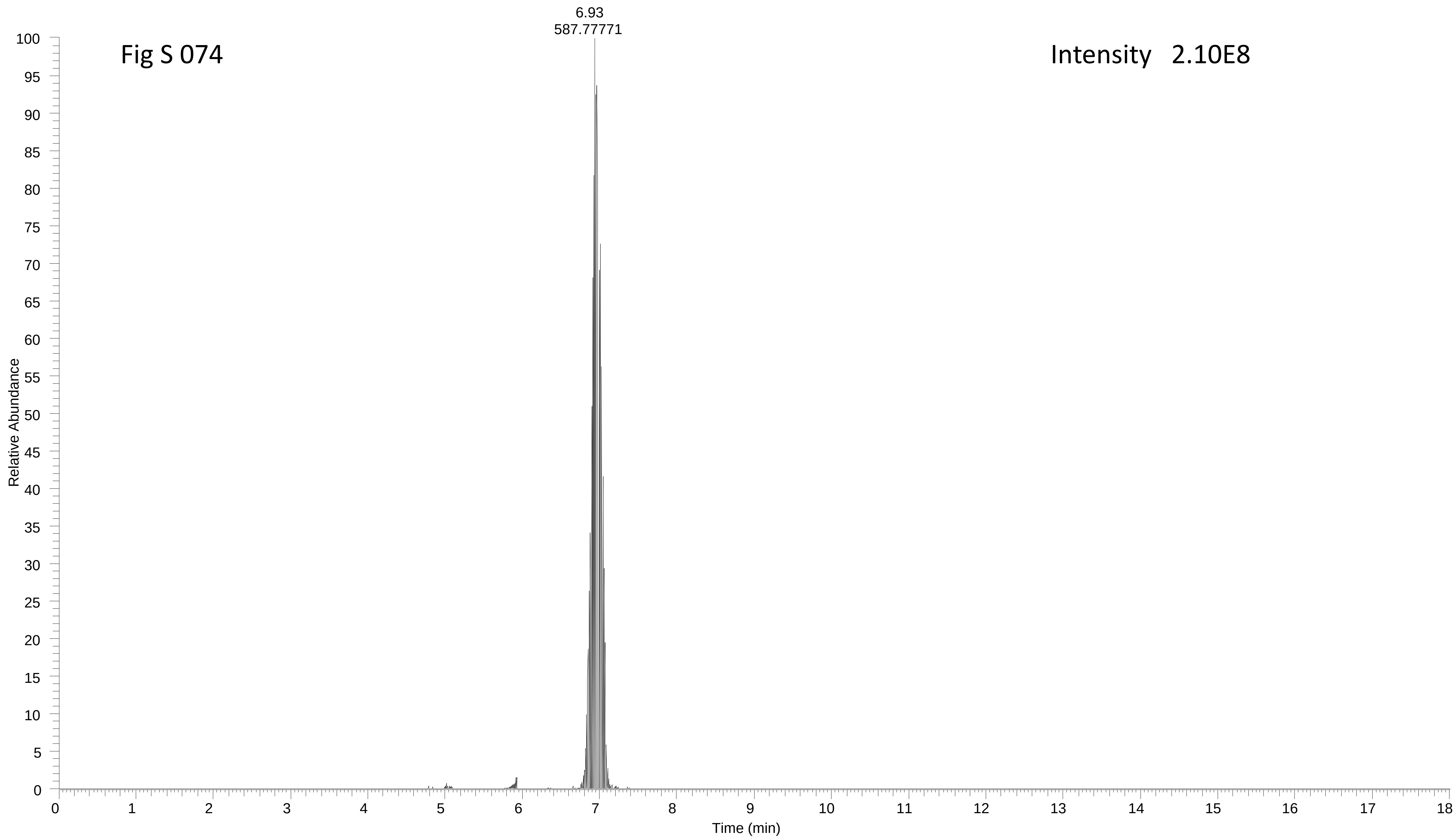

Fig S 074
Intensity 2.10E8
6.93
587.77771
Relative Abundance
Time (min)

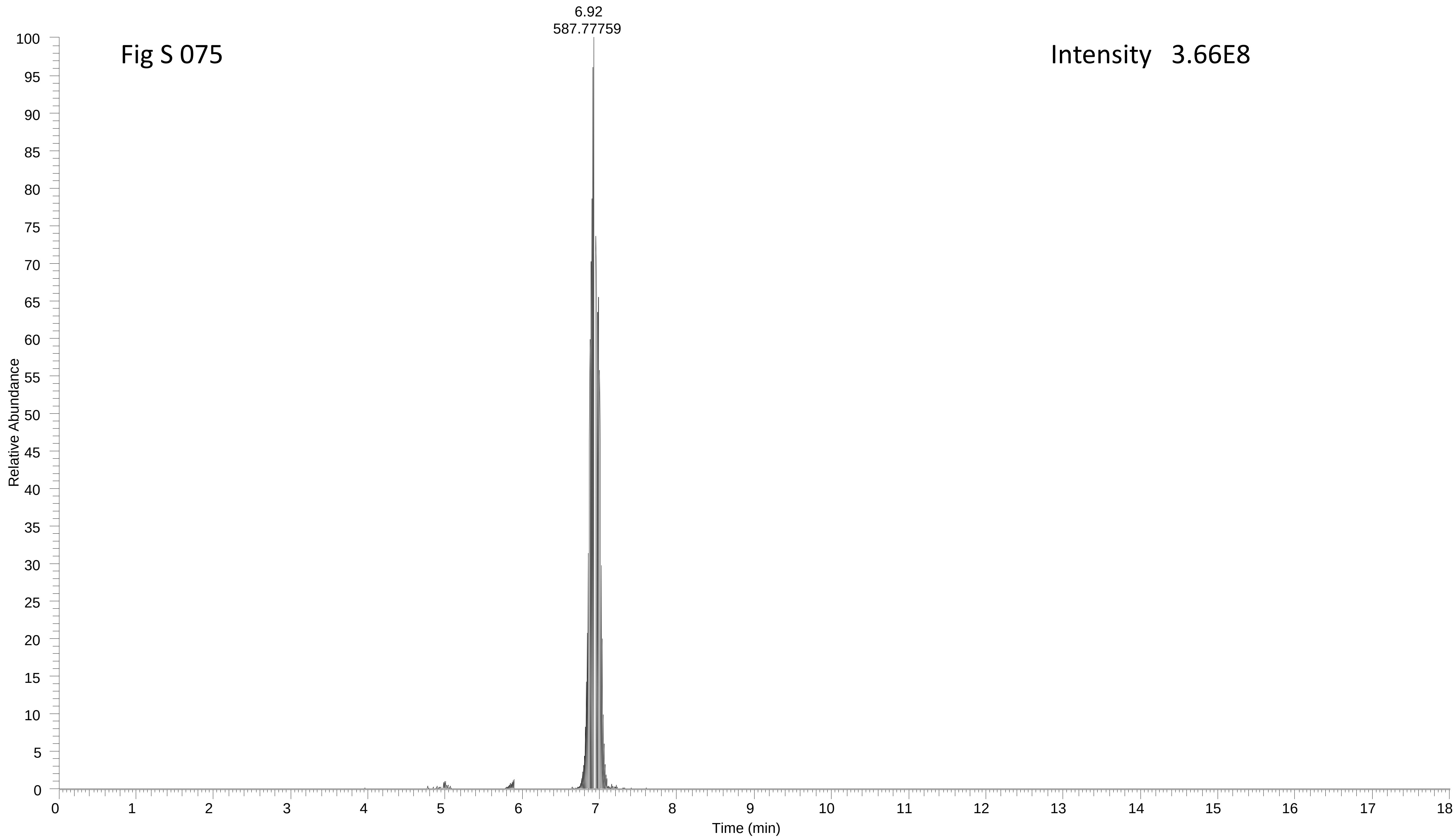

6.92
587.77759
Fig S 075
Intensity 3.66E8
Relative Abundance
Time (min)

S2 File. Chromatograms and MS/MS spectra.

Raw data MS/MS spectra EGPVGF**p**GADGR

Fig S 076: ostrich tendon
Fig S 077: goose neck
Fig S 078: duck neck
Fig S 079: turkey neck
Fig S 080: chicken leg
Fig S 081: pheasant meat strip
Fig S 082: goose meat strip
Fig S 083: goose leg
Fig S 084: pheasant leg
Fig S 085: guinea fowl torso
Fig S 086: pigeon torso
Fig S 087: partridge torso
Fig S 088: duck leg
Fig S 089: quail leg
Fig S 090: turkey leg

Remarks:
-Precursor m/z ≈ 587.78
-All bird samples provided MS/MS spectra for this peptide.
-Data recorded in June 2020.
-See Fig 6 in the main document for peak annotation.

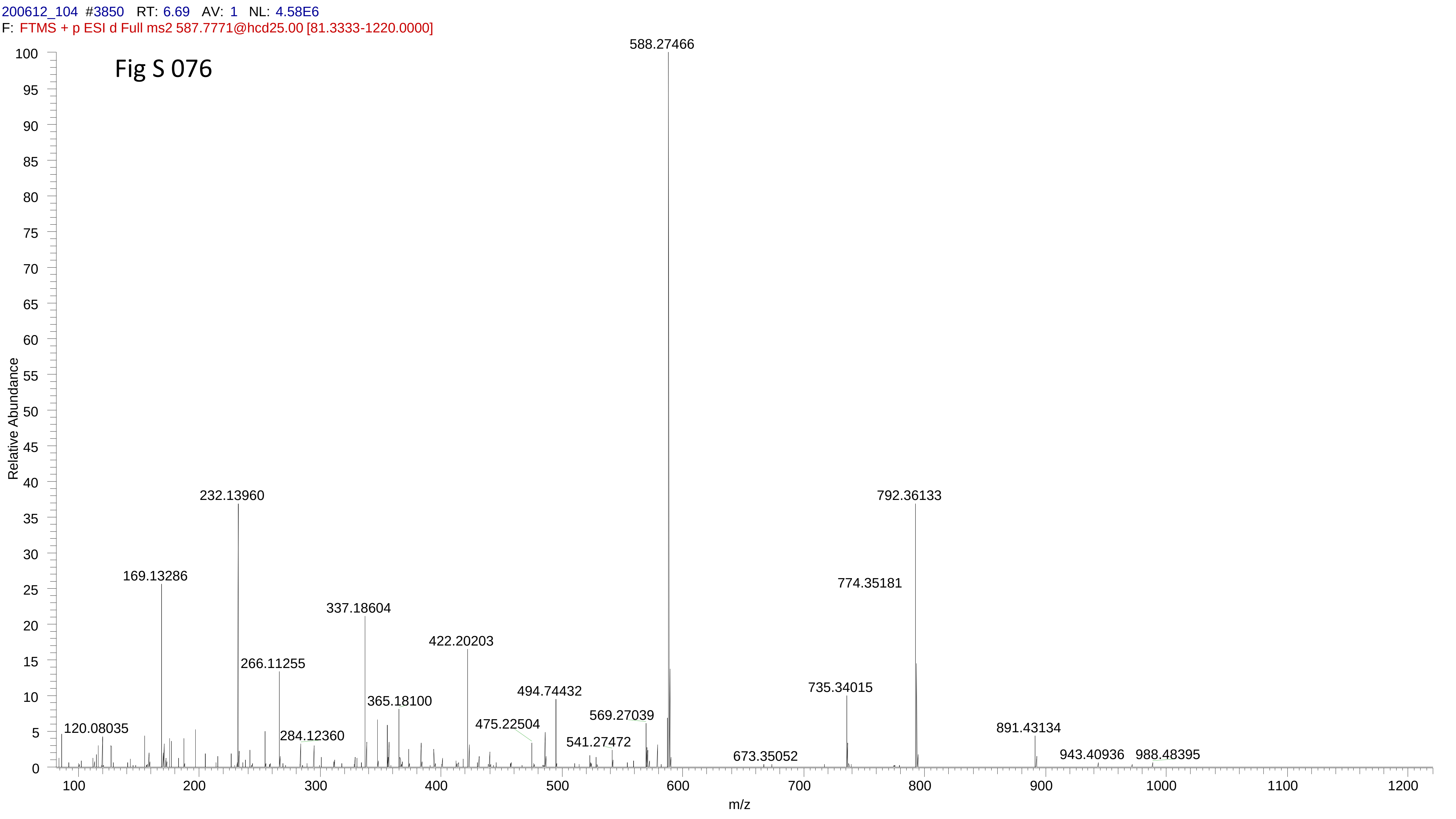
200612_104 #3850 RT: 6.69 AV: 1 NL: 4.58E6
F: FTMS + p ESI d Full ms2 587.7771@hcd25.00 [81.3333-1220.0000]
Fig S 076
588.27466
232.13960
792.36133
169.13286
774.35181
337.18604
422.20203
266.11255
494.74432
735.34015
365.18100
569.27039
120.08035
475.22504
891.43134
284.12360
541.27472
673.35052
943.40936
988.48395
Relative Abundance
m/z

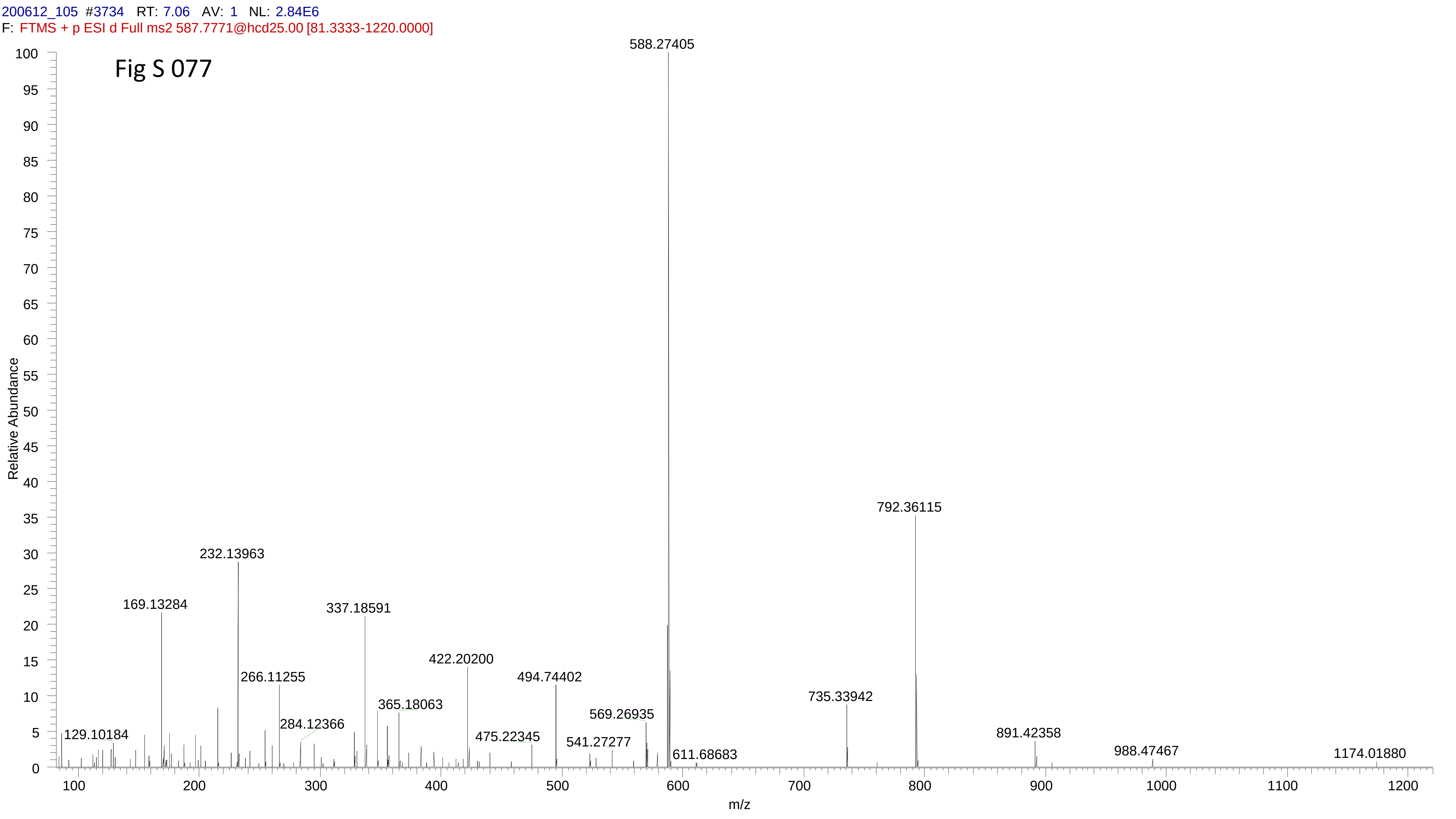
200612_105 #3734 RT: 7.06 AV: 1 NL: 2.84E6
F: FTMS + p ESI d Full ms2 587.7771@hcd25.00 [81.3333-1220.0000]
Fig S 077
588.27405
792.36115
232.13963
169.13284
337.18591
422.20200
266.11255
494.74402
735.33942
365.18063
569.26935
284.12366
129.10184
475.22345
891.42358
541.27277
611.68683
988.47467
1174.01880
Relative Abundance
m/z

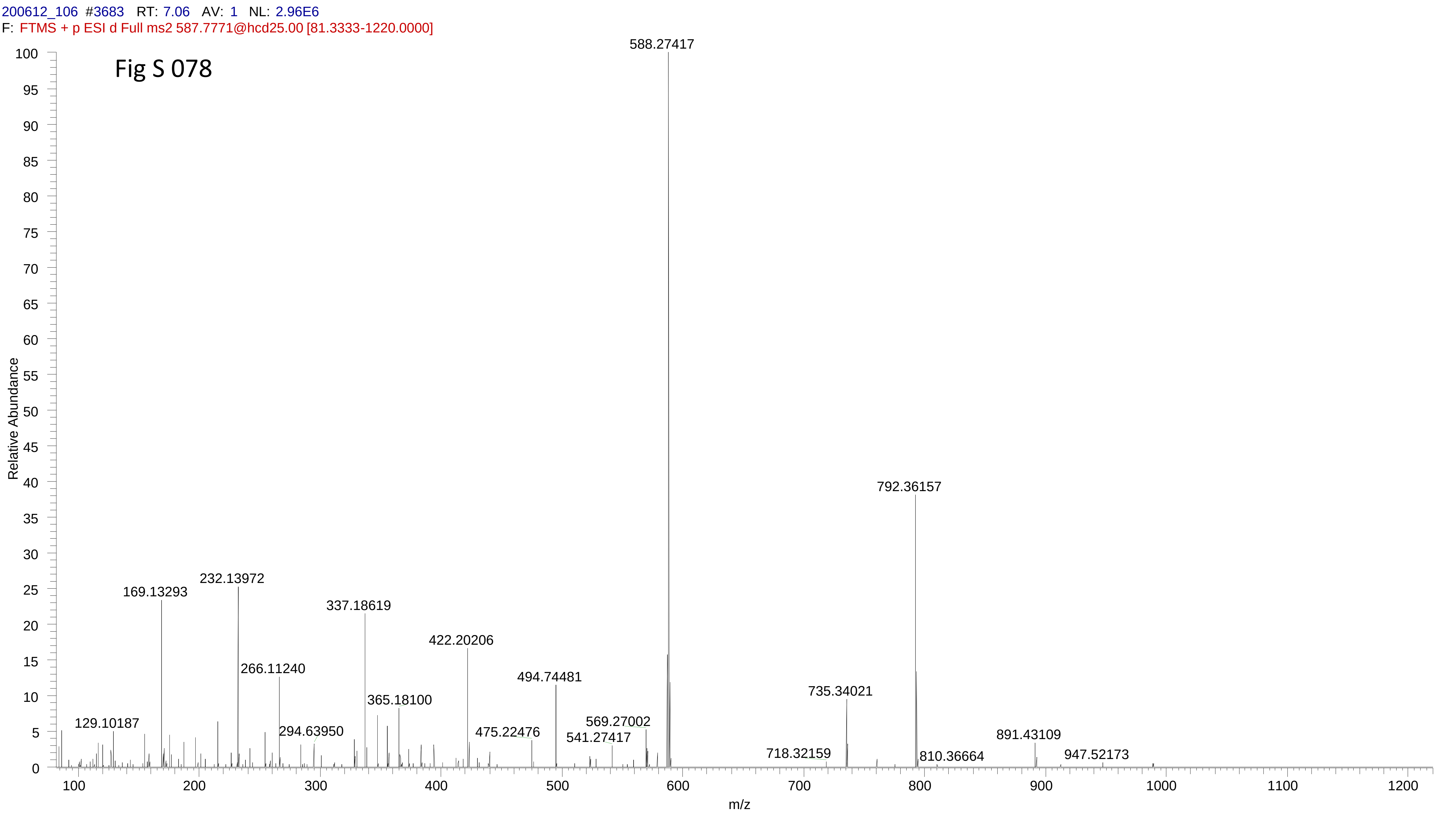
200612_106 #3683 RT: 7.06 AV: 1 NL: 2.96E6
F: FTMS + p ESI d Full ms2 587.7771@hcd25.00 [81.3333-1220.0000]
Fig S 078
588.27417
792.36157
232.13972
169.13293
337.18619
422.20206
266.11240
494.74481
735.34021
365.18100
129.10187
569.27002
294.63950
475.22476
541.27417
891.43109
718.32159
947.52173
810.36664
Relative Abundance
m/z

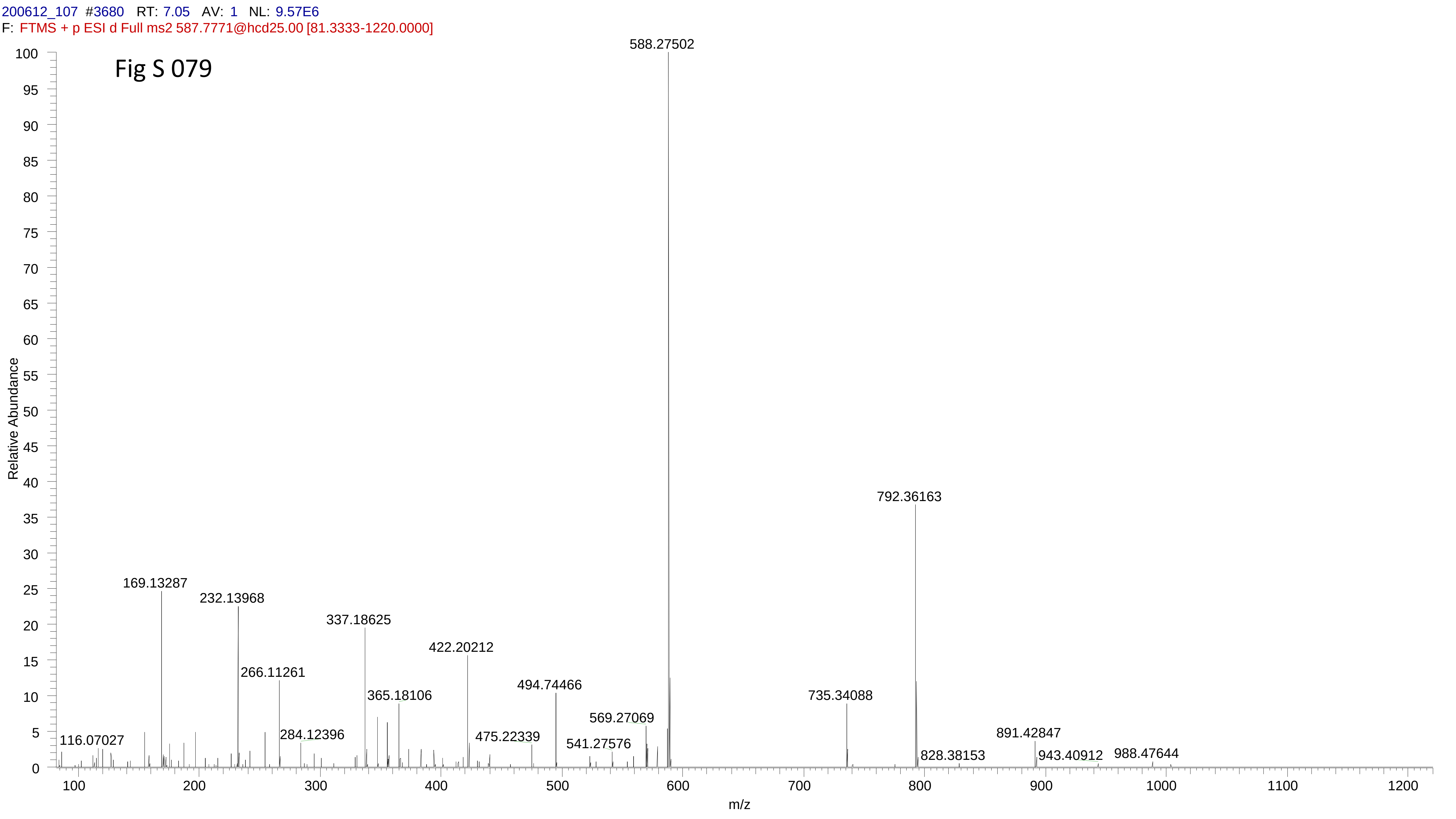
200612_107 #3680 RT: 7.05 AV: 1 NL: 9.57E6
F: FTMS + p ESI d Full ms2 587.7771@hcd25.00 [81.3333-1220.0000]
Fig S 079
588.27502
792.36163
169.13287
232.13968
337.18625
422.20212
266.11261
365.18106
494.74466
735.34088
569.27069
116.07027
284.12396
475.22339
541.27576
891.42847
828.38153
943.40912
988.47644
Relative Abundance
m/z

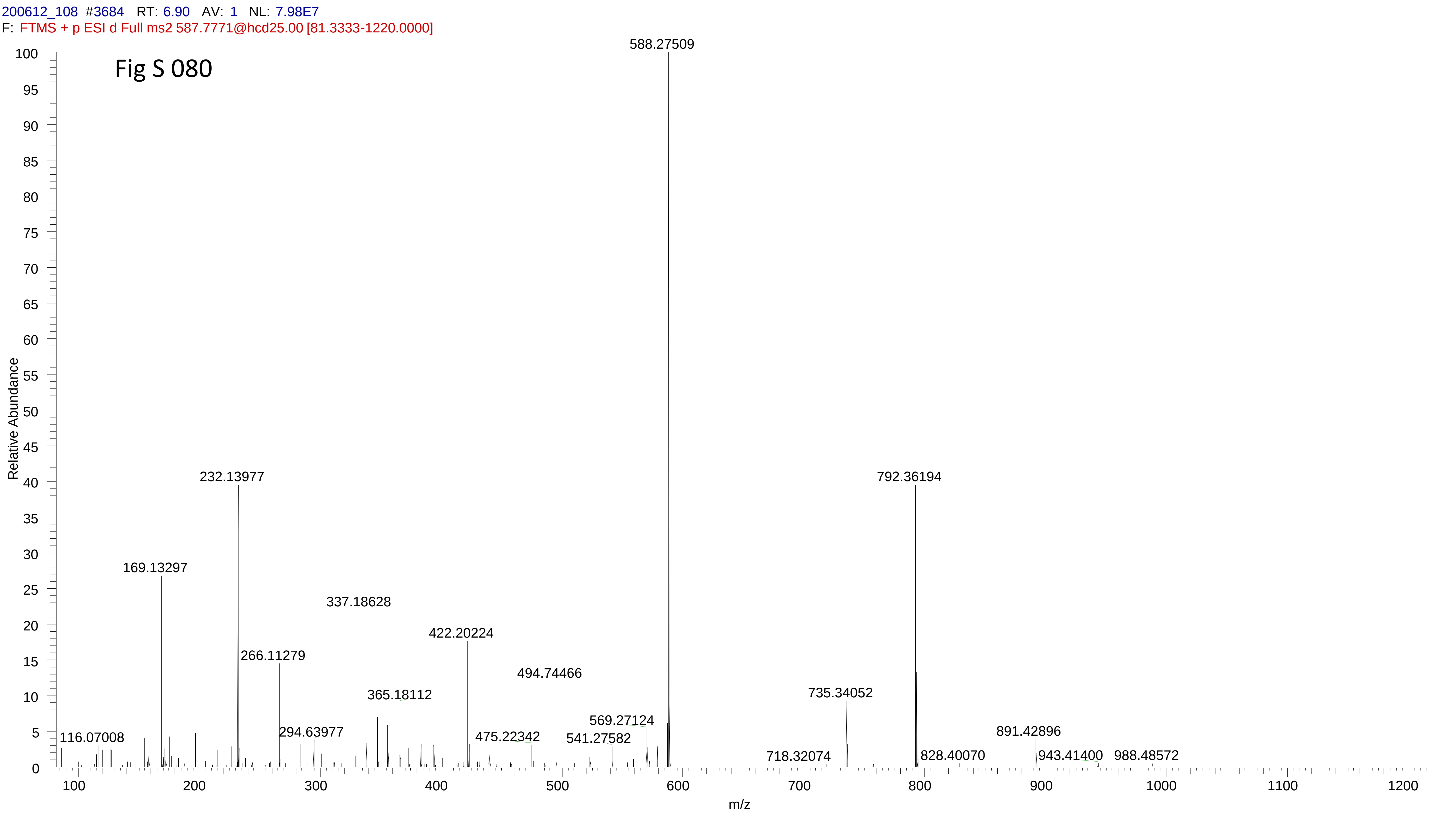
200612_108 #3684 RT: 6.90 AV: 1 NL: 7.98E7
F: FTMS + p ESI d Full ms2 587.7771@hcd25.00 [81.3333-1220.0000]
Fig S 080
588.27509
792.36194
232.13977
169.13297
337.18628
422.20224
266.11279
494.74466
365.18112
735.34052
569.27124
294.63977
475.22342
541.27582
891.42896
116.07008
718.32074
828.40070
943.41400
988.48572
Relative Abundance
m/z

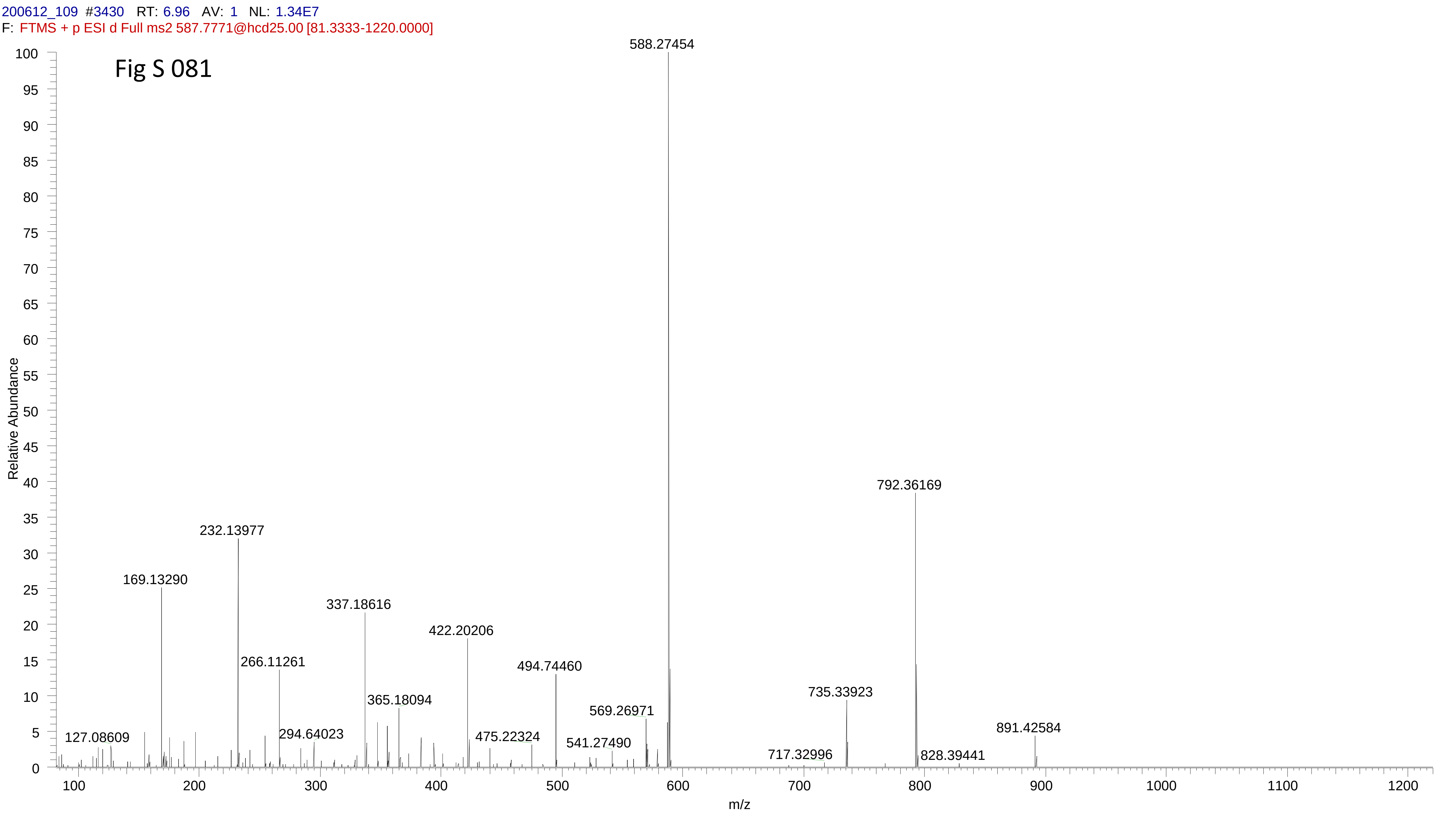

200612_109 #3430 RT: 6.96 AV: 1 NL: 1.34E7
F: FTMS + p ESI d Full ms2 587.7771@hcd25.00 [81.3333-1220.0000]
Fig S 081
588.27454
792.36169
232.13977
169.13290
337.18616
422.20206
266.11261
494.74460
365.18094
735.33923
569.26971
891.42584
127.08609
294.64023
475.22324
541.27490
717.32996
828.39441
Relative Abundance
m/z

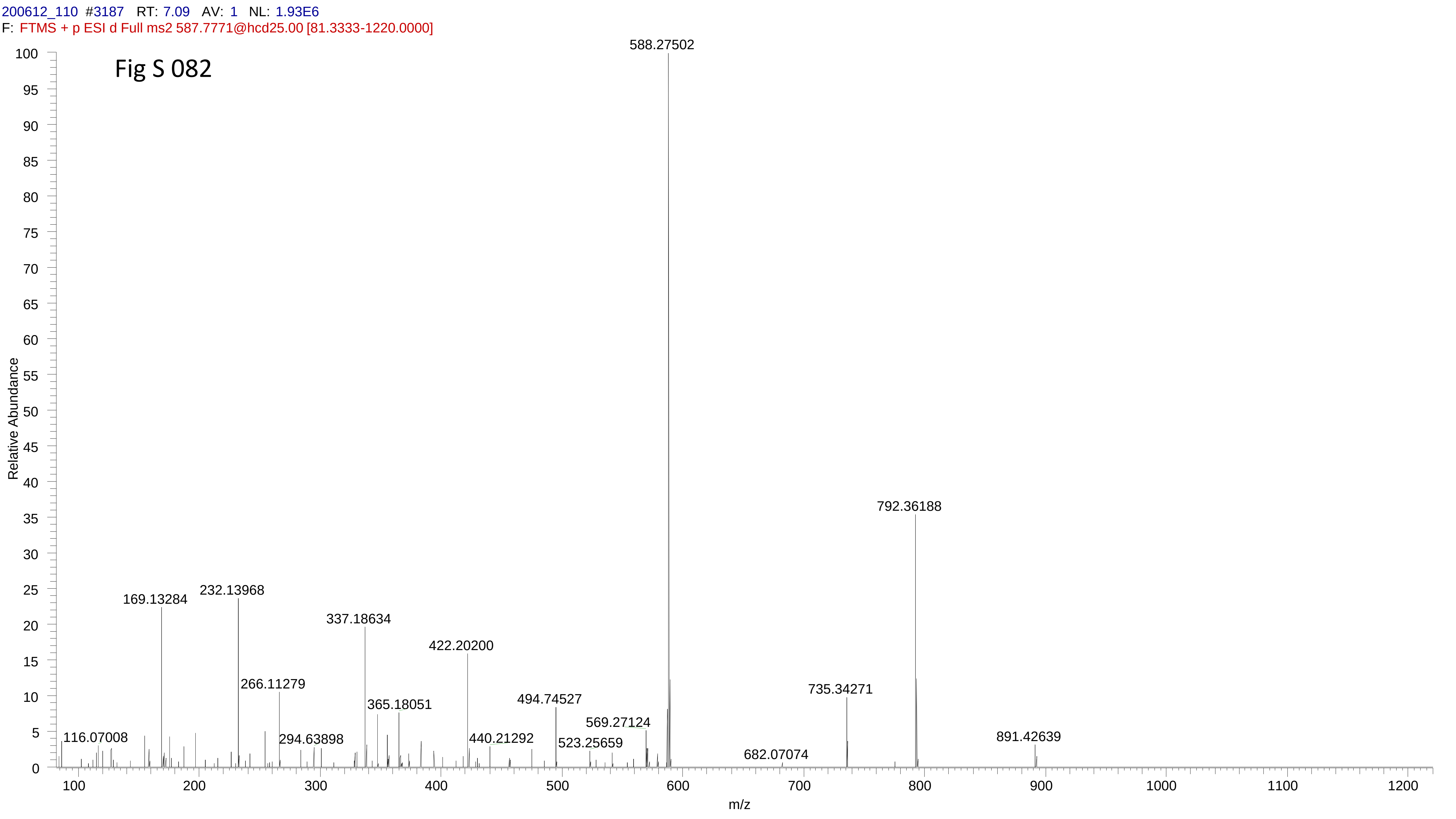
200612_110 #3187 RT: 7.09 AV: 1 NL: 1.93E6
F: FTMS + p ESI d Full ms2 587.7771@hcd25.00 [81.3333-1220.0000]
Fig S 082
Relative Abundance
m/z
588.27502
792.36188
169.13284
232.13968
337.18634
422.20200
266.11279
365.18051
494.74527
735.34271
569.27124
116.07008
294.63898
440.21292
523.25659
891.42639
682.07074

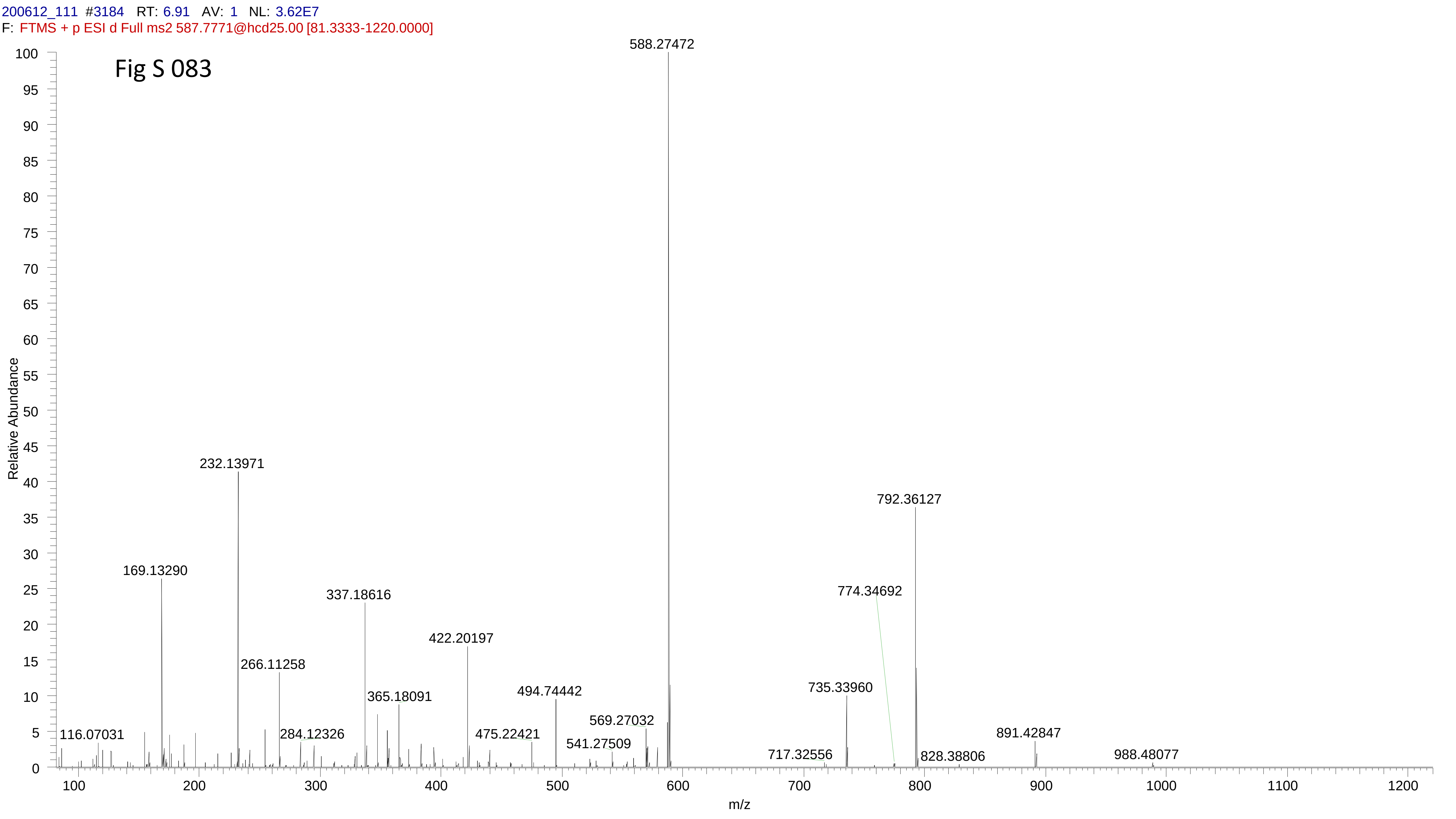
200612_111 #3184 RT: 6.91 AV: 1 NL: 3.62E7
F: FTMS + p ESI d Full ms2 587.7771@hcd25.00 [81.3333-1220.0000]
Fig S 083
Relative Abundance
m/z
588.27472
232.13971
169.13290
337.18616
422.20197
266.11258
365.18091
494.74442
116.07031
284.12326
475.22421
569.27032
541.27509
792.36127
774.34692
735.33960
717.32556
828.38806
891.42847
988.48077

200612_112 #3439 RT: 6.79 AV: 1 NL: 1.76E6
F: FTMS + p ESI d Full ms2 587.7771@hcd25.00 [81.3333-1220.0000]

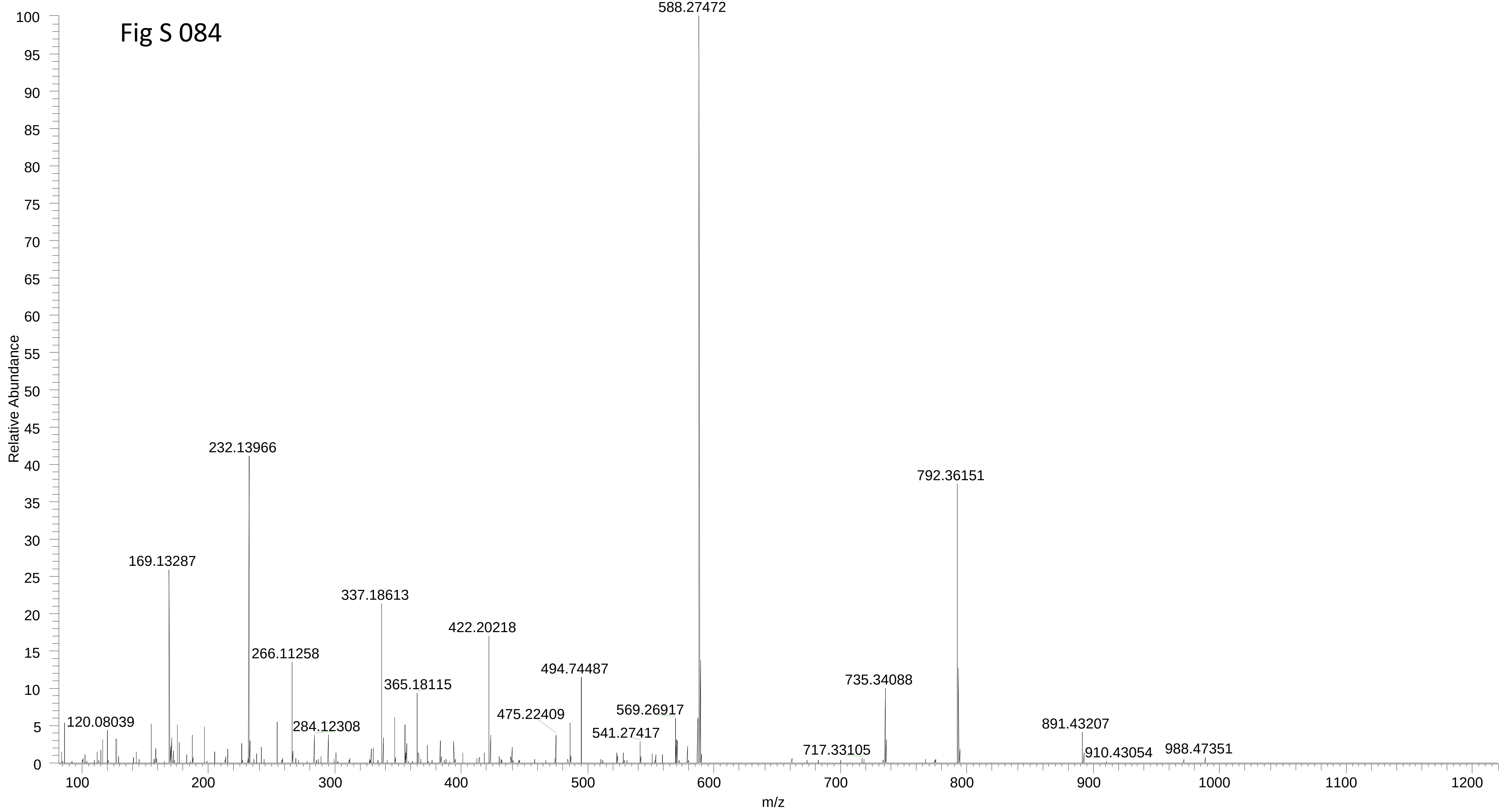

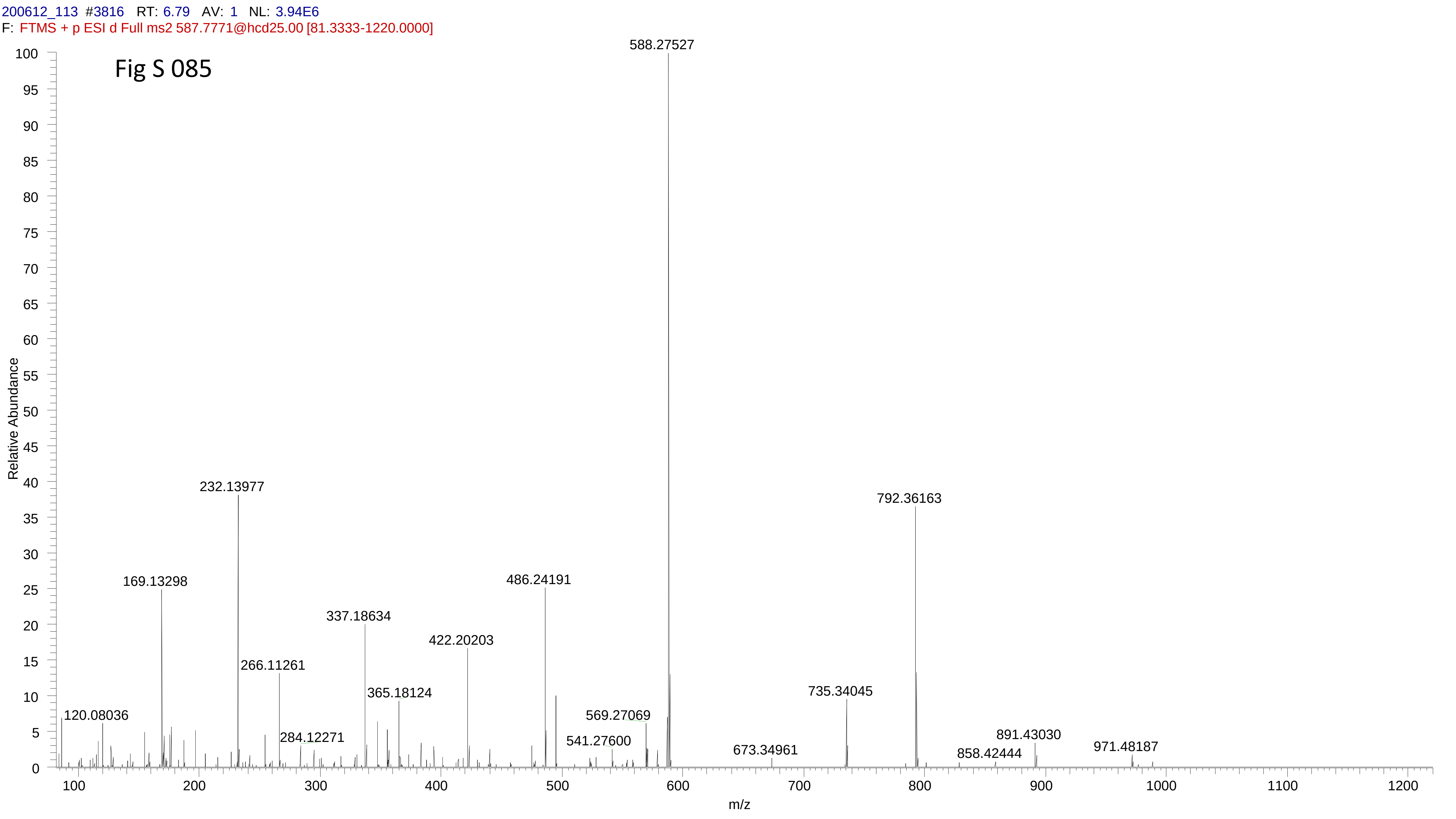
200612_113 #3816 RT: 6.79 AV: 1 NL: 3.94E6
F: FTMS + p ESI d Full ms2 587.7771@hcd25.00 [81.3333-1220.0000]
Fig S 085
588.27527
792.36163
232.13977
169.13298
486.24191
337.18634
422.20203
266.11261
365.18124
735.34045
120.08036
569.27069
284.12271
541.27600
891.43030
673.34961
858.42444
971.48187
Relative Abundance
m/z

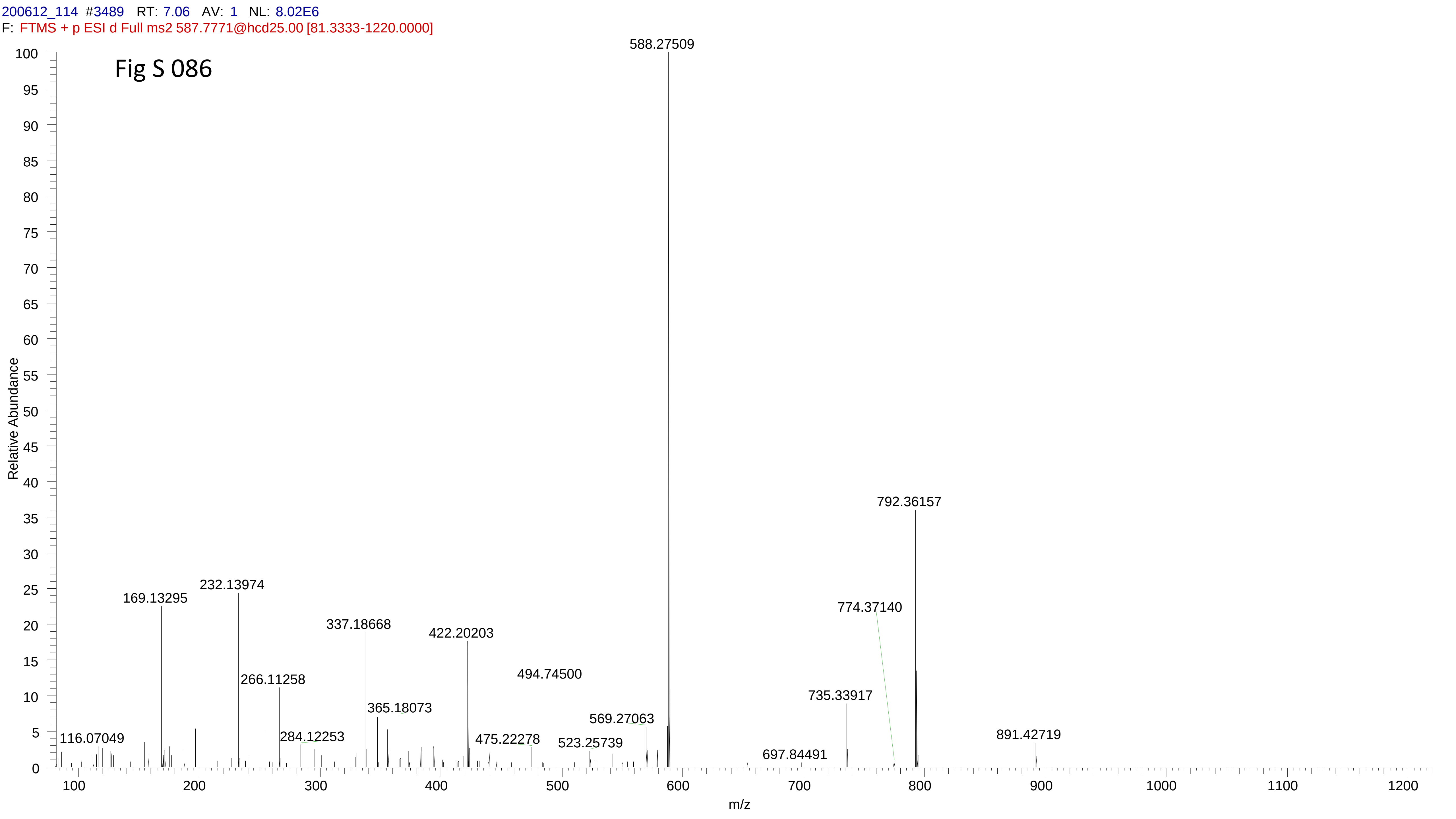
200612_114 #3489 RT: 7.06 AV: 1 NL: 8.02E6
F: FTMS + p ESI d Full ms2 587.7771@hcd25.00 [81.3333-1220.0000]
Fig S 086
588.27509
792.36157
774.37140
232.13974
169.13295
337.18668
422.20203
266.11258
494.74500
365.18073
735.33917
569.27063
116.07049
284.12253
475.22278
523.25739
891.42719
697.84491
Relative Abundance
m/z

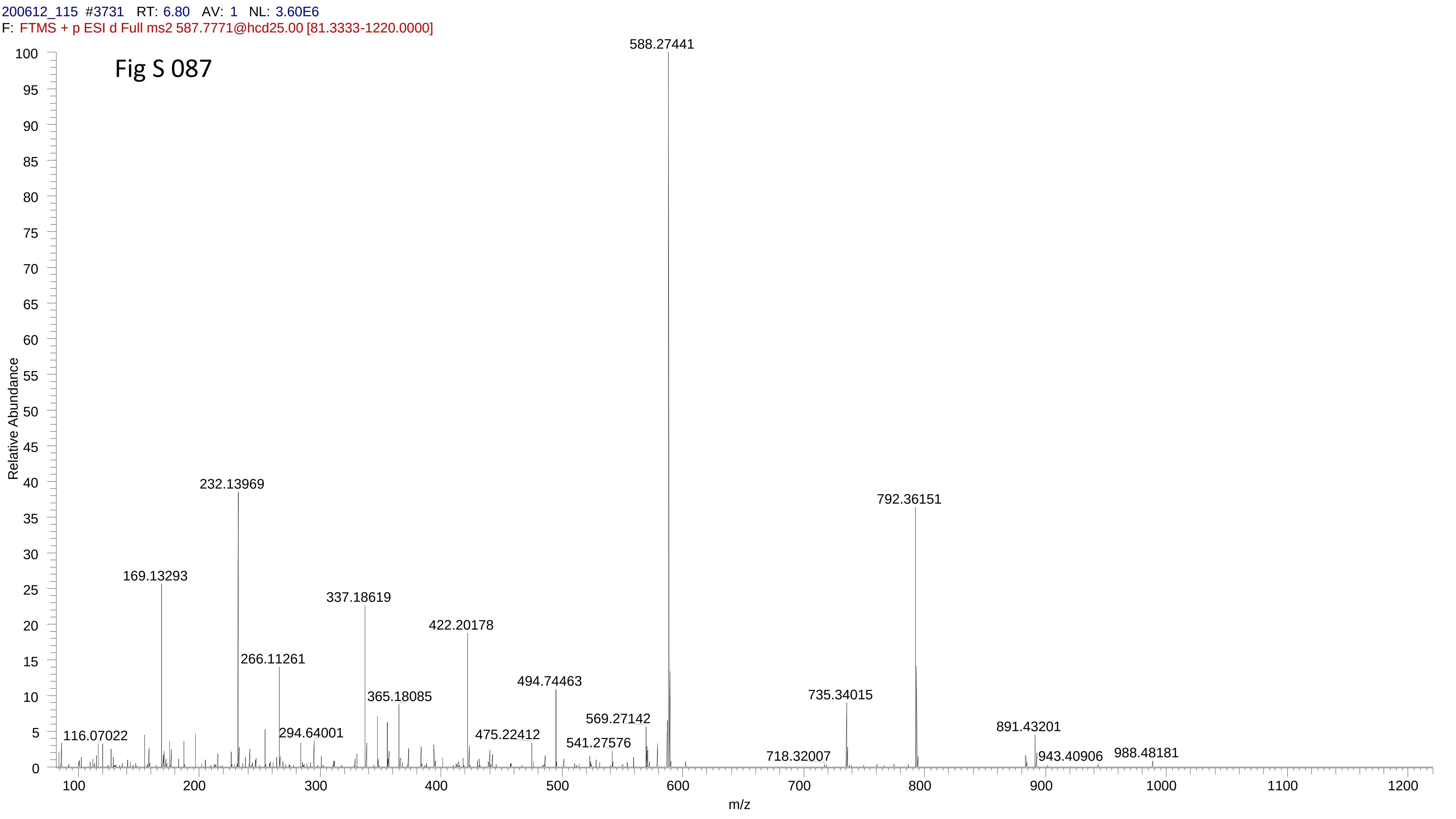
200612_115 #3731 RT: 6.80 AV: 1 NL: 3.60E6
F: FTMS + p ESI d Full ms2 587.7771@hcd25.00 [81.3333-1220.0000]
Fig S 087
588.27441
792.36151
232.13969
169.13293
337.18619
422.20178
266.11261
494.74463
365.18085
735.34015
569.27142
116.07022
294.64001
475.22412
541.27576
891.43201
718.32007
943.40906
988.48181
Relative Abundance
m/z

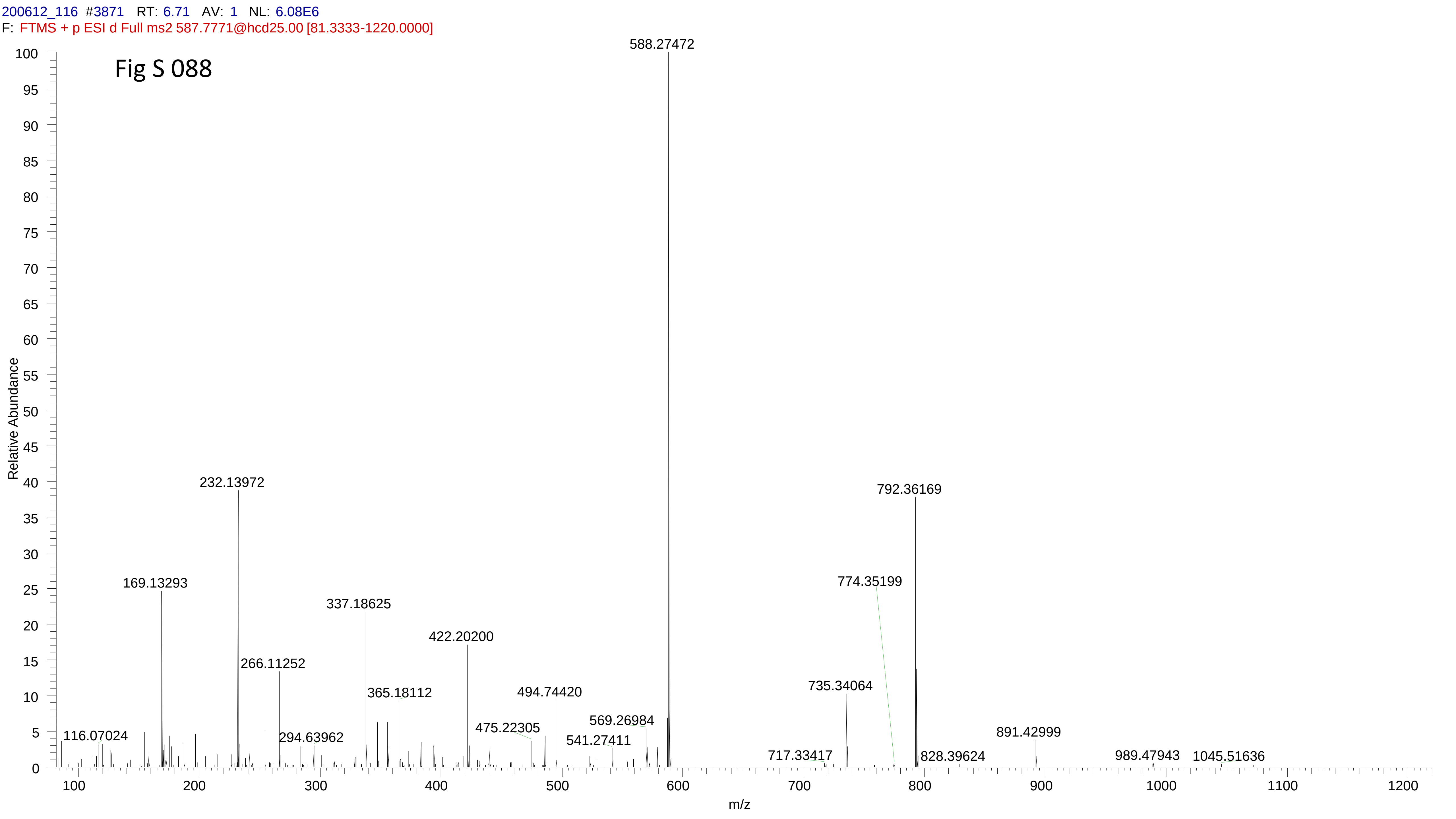
200612_116 #3871 RT: 6.71 AV: 1 NL: 6.08E6
F: FTMS + p ESI d Full ms2 587.7771@hcd25.00 [81.3333-1220.0000]
Fig S 088
Relative Abundance
m/z
588.27472
232.13972
792.36169
169.13293
774.35199
337.18625
422.20200
266.11252
365.18112
494.74420
735.34064
475.22305
569.26984
116.07024
294.63962
541.27411
891.42999
717.33417
828.39624
989.47943
1045.51636

200612_117 #3296 RT: 6.96 AV: 1 NL: 3.46E7
F: FTMS + p ESI d Full ms2 587.7771@hcd25.00 [81.3333-1220.0000]

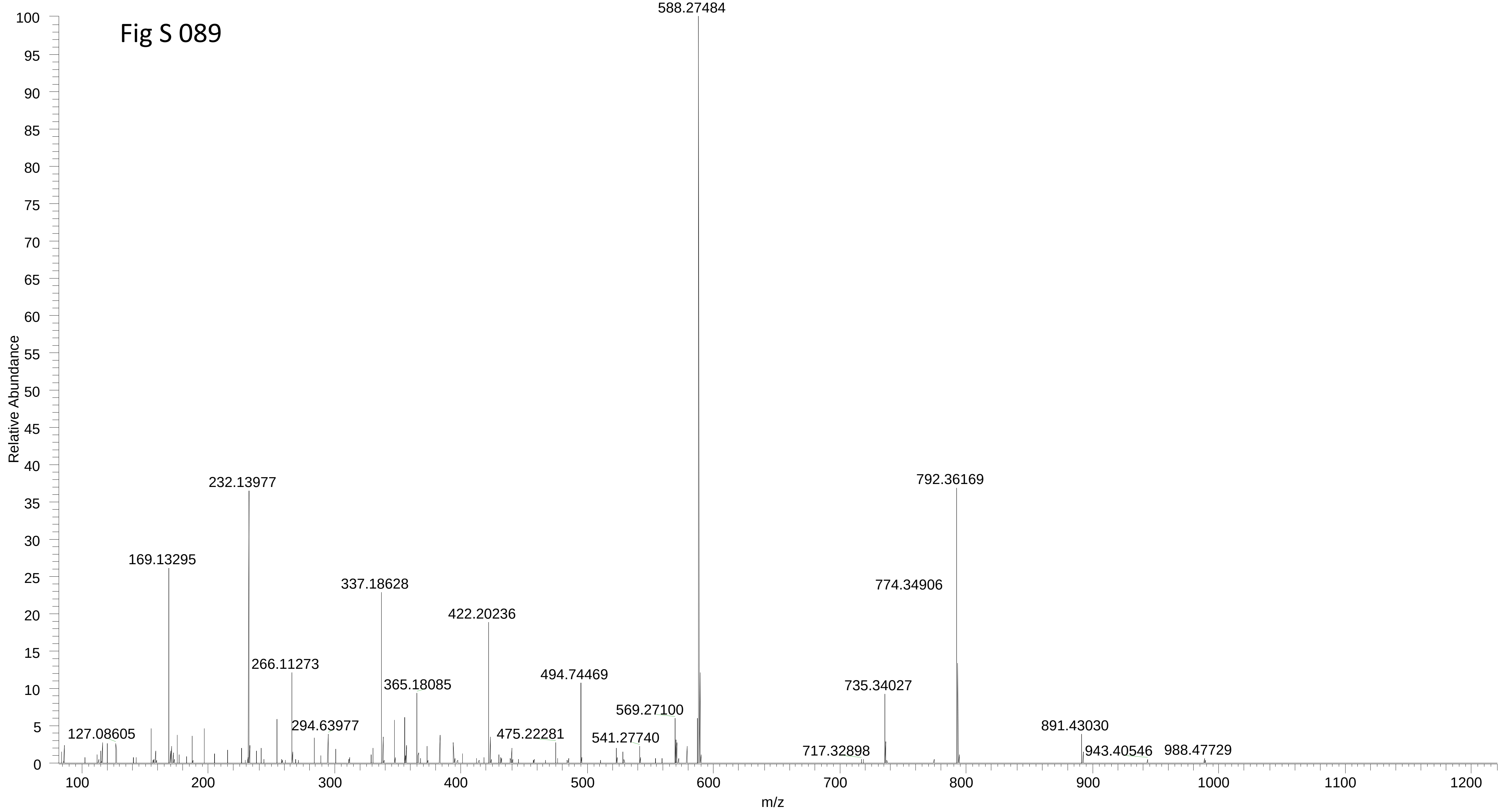

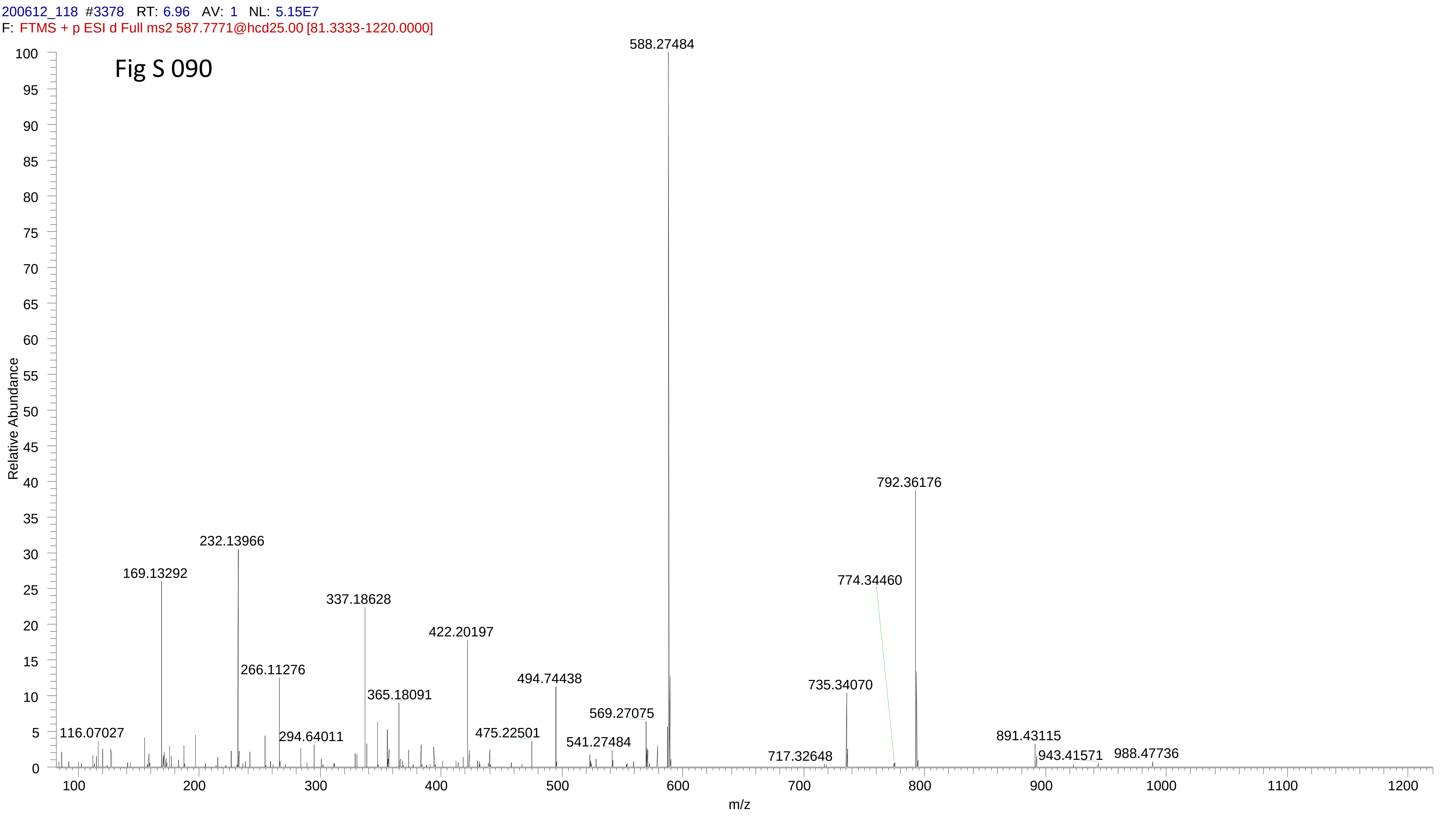
200612_118 #3378 RT: 6.96 AV: 1 NL: 5.15E7
F: FTMS + p ESI d Full ms2 587.7771@hcd25.00 [81.3333-1220.0000]
Fig S 090
Relative Abundance
m/z
588.27484
792.36176
232.13966
169.13292
774.34460
337.18628
422.20197
266.11276
494.74438
735.34070
365.18091
569.27075
116.07027
294.64011
475.22501
541.27484
891.43115
717.32648
943.41571
988.47736

S2 File. Chromatograms and MS/MS spectra.

Raw data chromatograms (deamidated) VGPIGPAGNR

Fig S 091: chicken soup
Fig S 092: chicken broth A
Fig S 093: chicken broth B
Fig S 094: beef broth

Remarks:
-Extracted m/z range 469.75-469.77
-The retention time and m/z of the base peak are provided per peak and the provided intensity is of the highest peak.
-Data recorded in October 2021.

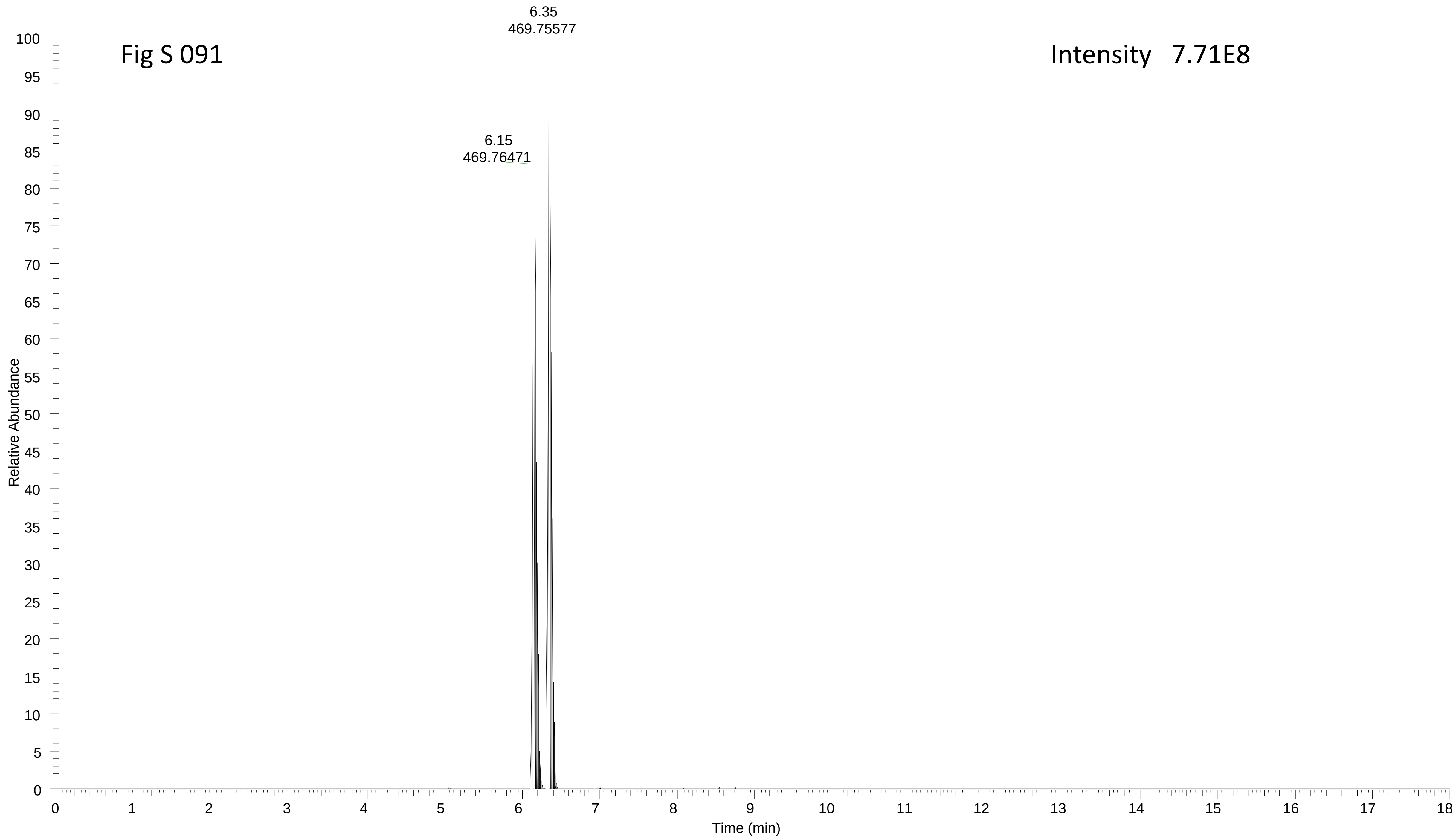

Fig S 091
Intensity 7.71E8
6.35
469.75577
6.15
469.76471
Relative Abundance
Time (min)

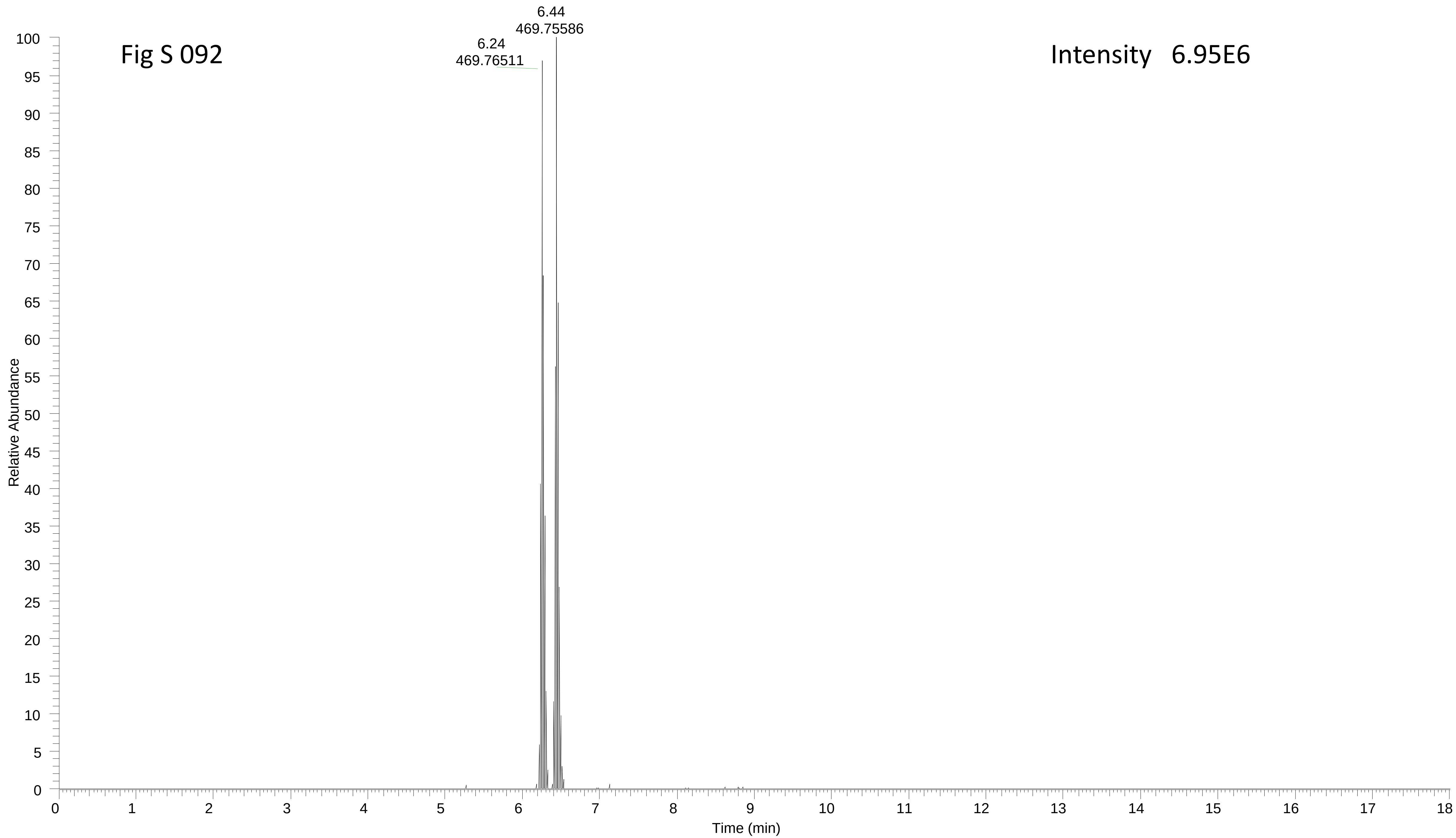
Fig S 092
Intensity 6.95E6
6.44
469.75586
6.24
469.76511
Relative Abundance
Time (min)
0
5
10
15
20
25
30
35
40
45
50
55
60
65
70
75
80
85
90
95
100
1
2
3
4
5
6
7
8
9
10
11
12
13
14
15
16
17
18

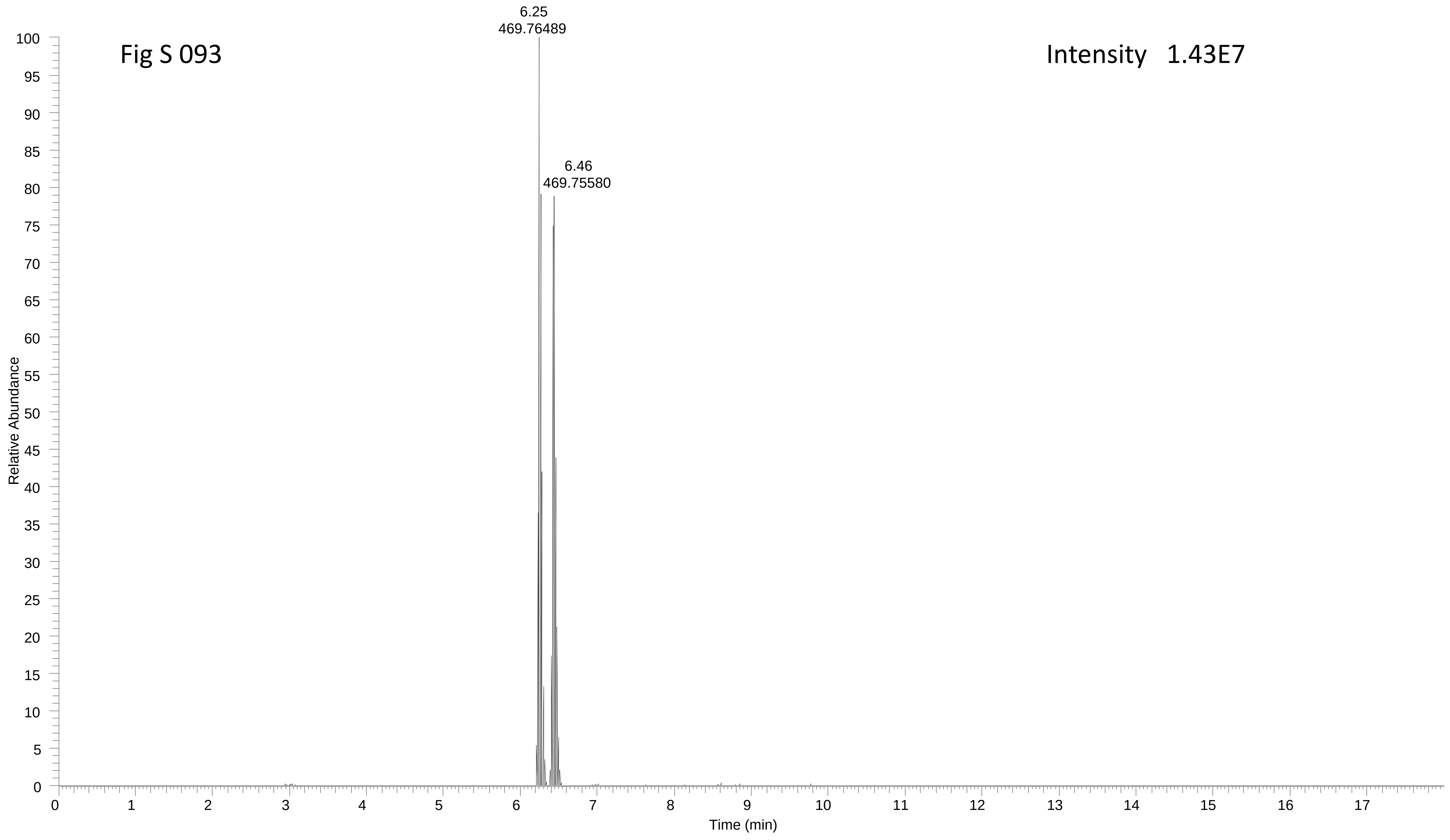
Fig S 093
Intensity 1.43E7
6.25
469.76489
6.46
469.75580
Relative Abundance
Time (min)
0
5
10
15
20
25
30
35
40
45
50
55
60
65
70
75
80
85
90
95
100
0
1
2
3
4
5
6
7
8
9
10
11
12
13
14
15
16
17

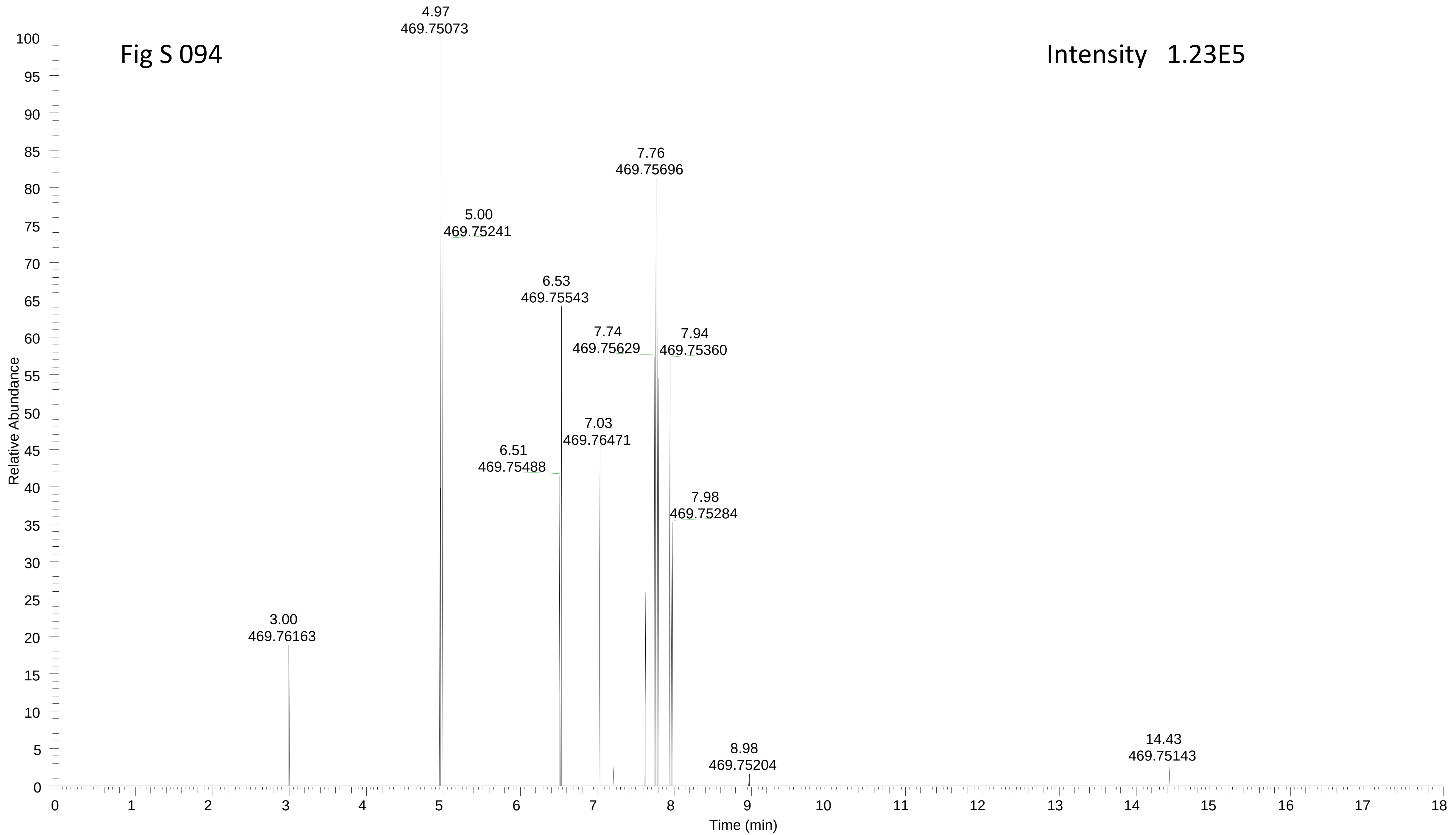
Fig S 094
Intensity 1.23E5
4.97
469.75073
5.00
469.75241
6.53
469.75543
6.51
469.75488
7.03
469.76471
7.76
469.75696
7.74
469.75629
7.94
469.75360
7.98
469.75284
3.00
469.76163
8.98
469.75204
14.43
469.75143
Relative Abundance
Time (min)

S2 File. Chromatograms and MS/MS spectra.

Raw data MS/MS spectra VGPIGPAGNR

Fig S 095: chicken soup
Fig S 096: chicken broth A
Fig S 097: chicken broth B

Remarks:
-Precursor m/z ≈ 469.26
-Beef broth did not provide MS/MS spectra for this peptide.
-Data recorded in October 2021.
-See Fig 4a in the main document for peak annotation.

Fig S 095

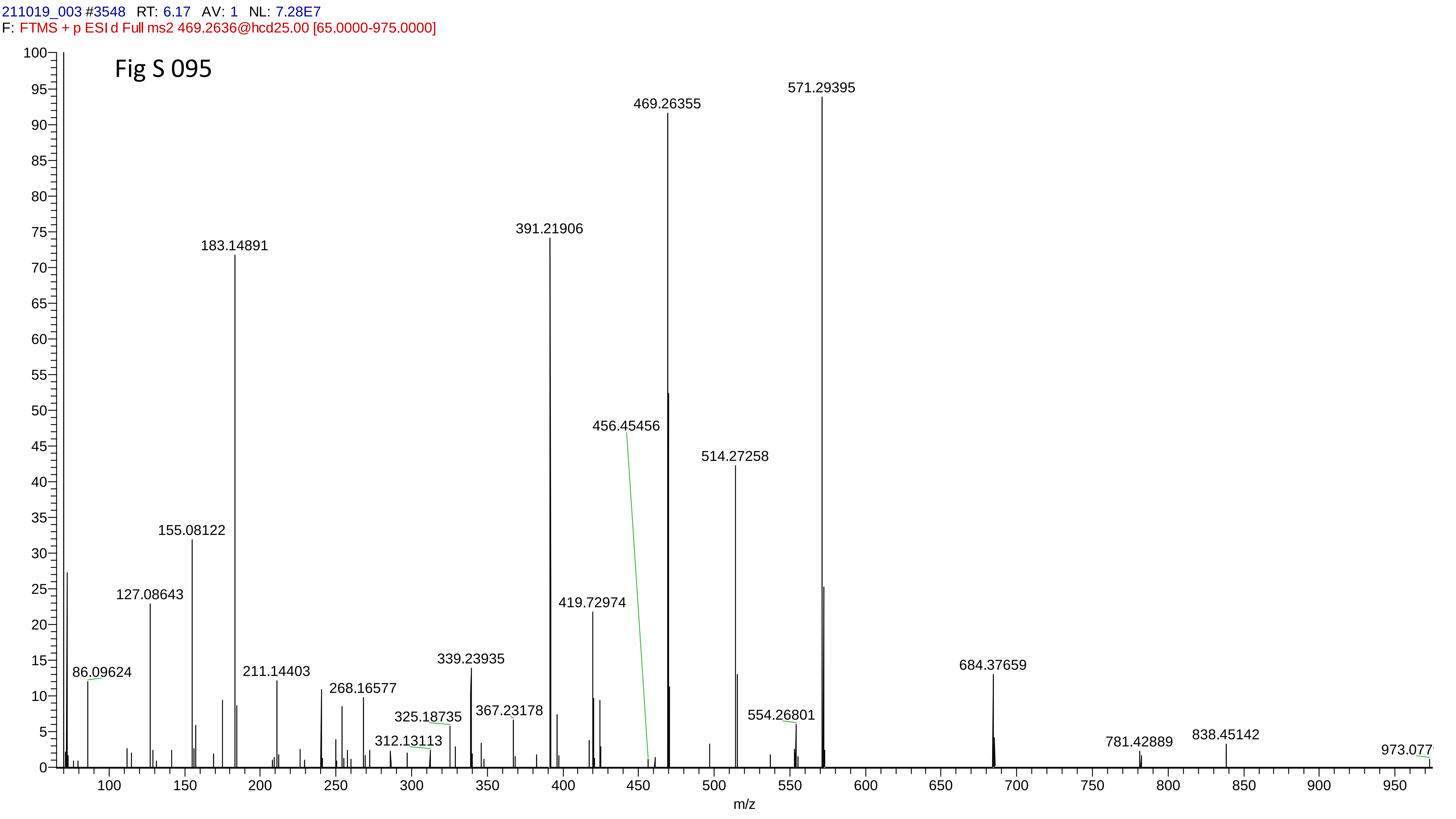
211019_003 #3548 RT: 6.17 AV: 1 NL: 7.28E7
F: FTMS + p ESI d Full ms2 469.2636@hcd25.00 [65.0000-975.0000]
571.29395
469.26355
391.21906
183.14891
456.45456
514.27258
155.08122
127.08643
419.72974
339.23935
86.09624
211.14403
268.16577
684.37659
325.18735
367.23178
554.26801
312.13113
781.42889
838.45142
m/z

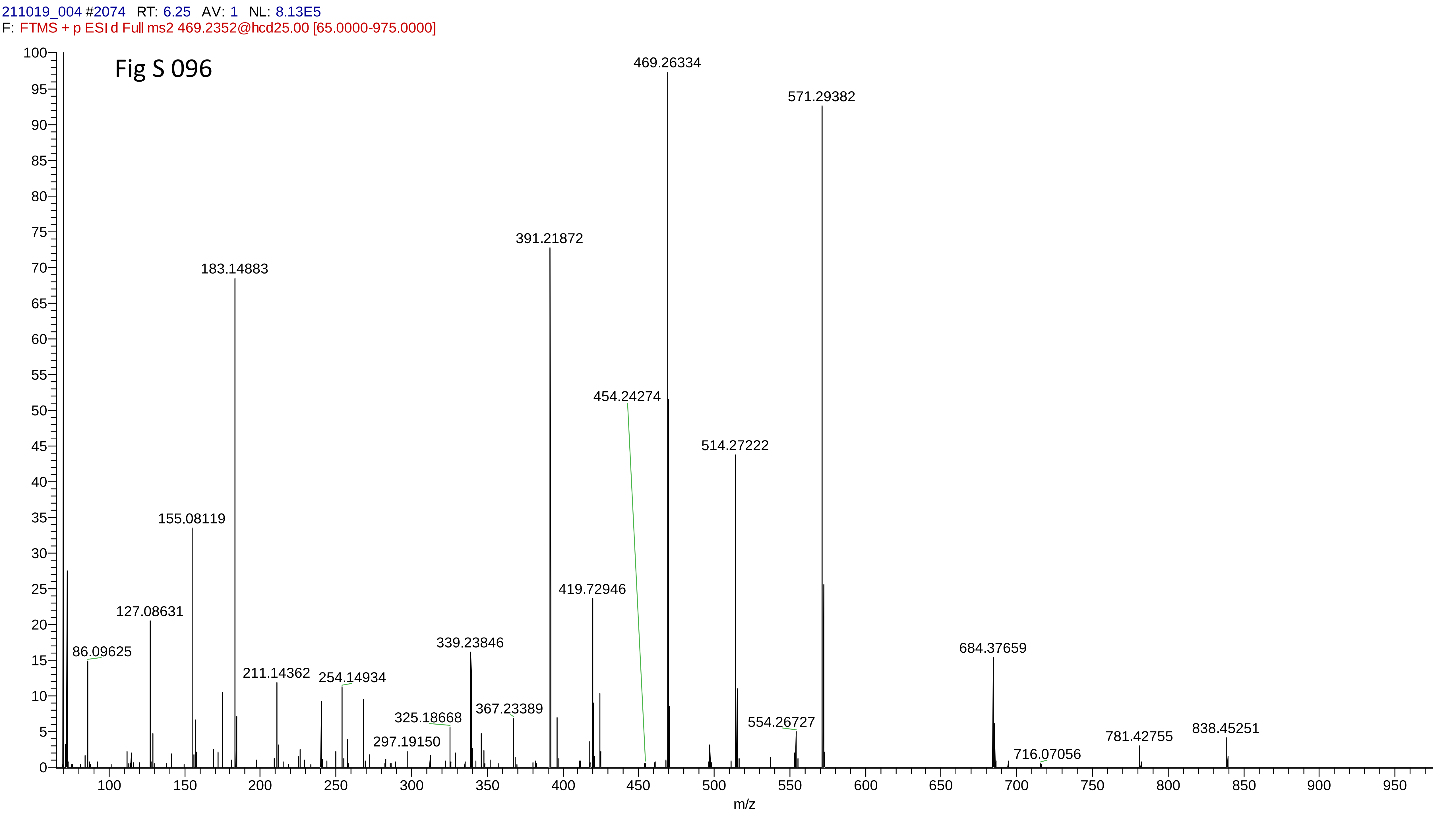
211019_004 #2074 RT: 6.25 AV: 1 NL: 8.13E5
F: FTMS + p ESI d Full ms2 469.2352@hcd25.00 [65.0000-975.0000]
Fig S 096
469.26334
571.29382
391.21872
183.14883
454.24274
514.27222
155.08119
419.72946
127.08631
86.09625
339.23846
684.37659
211.14362
254.14934
367.23389
325.18668
554.26727
297.19150
781.42755
838.45251
716.07056
m/z

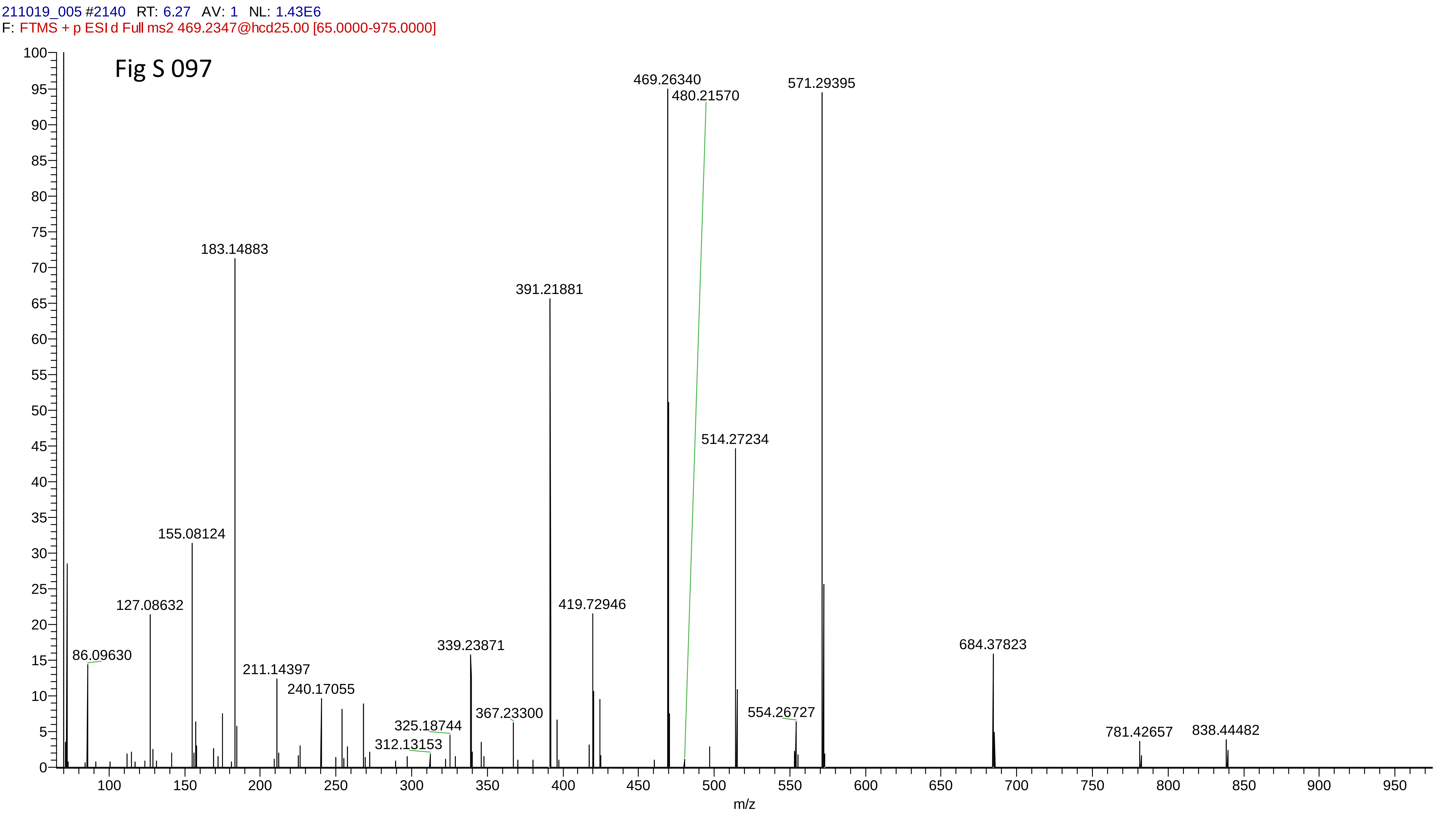
211019_005 #2140 RT: 6.27 AV: 1 NL: 1.43E6
F: FTMS + p ESI d Full ms2 469.2347@hcd25.00 [65.0000-975.0000]
Fig S 097
469.26340
480.21570
571.29395
183.14883
391.21881
514.27234
155.08124
127.08632
419.72946
86.09630
339.23871
684.37823
211.14397
240.17055
367.23300
554.26727
325.18744
312.13153
781.42657
838.44482
m/z

S2 File. Chromatograms and MS/MS spectra.

Raw data MS/MS spectra deamidated VGPIGPAGNR

Fig S 098: chicken soup
Fig S 099: chicken broth A
Fig S 100: chicken broth B

Remarks:
-Precursor m/z ≈ 469.76
-Beef broth did not provide MS/MS spectra for this peptide.
-Data recorded in October 2021.
-See Fig 4a in the main document for peak annotation, corrected for the presence of deamidated N9 (+ 0.984 Da).

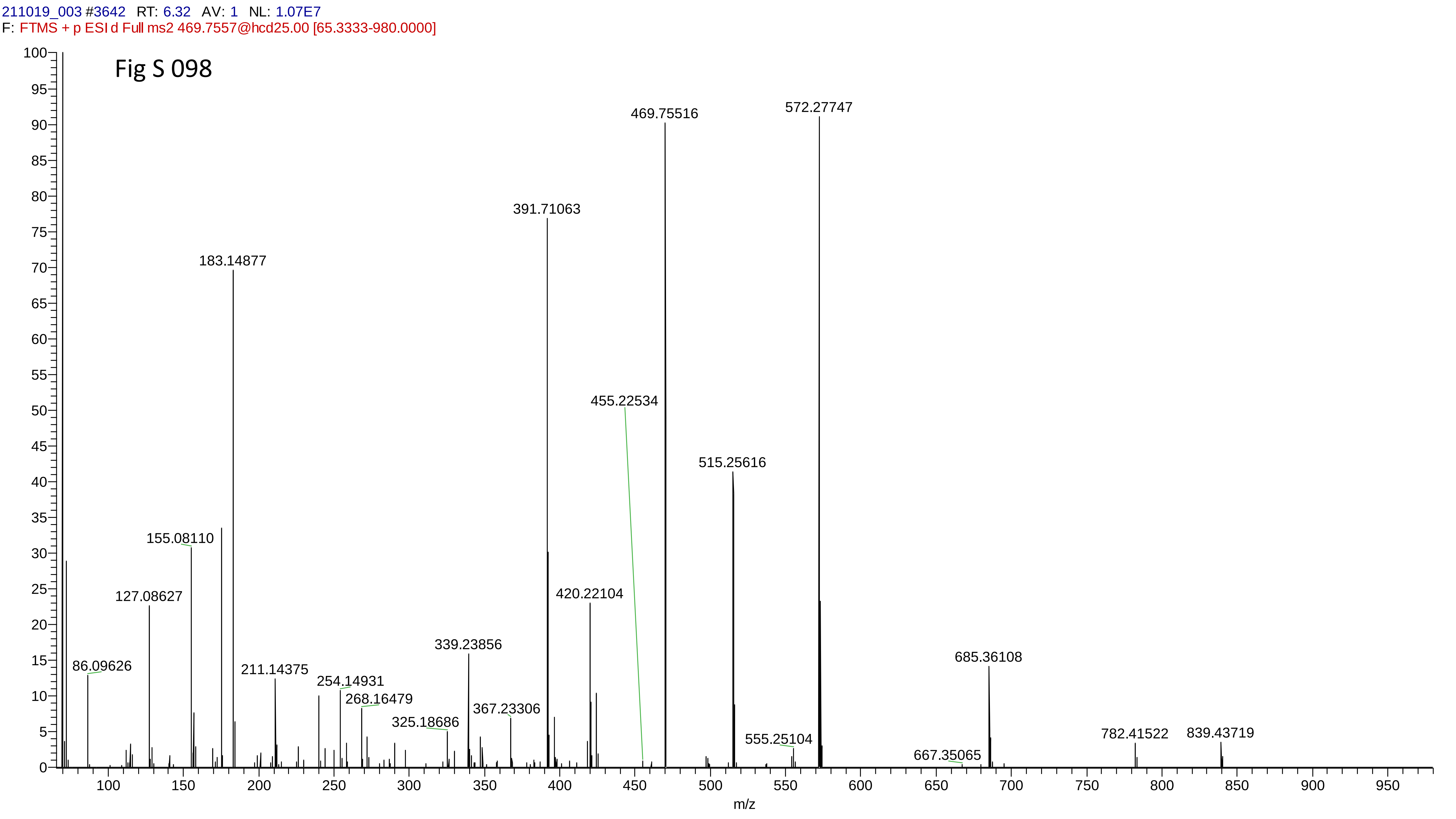
211019_003 #3642 RT: 6.32 AV: 1 NL: 1.07E7
F: FTMS + p ESI d Full ms2 469.7557@hcd25.00 [65.3333-980.0000]
Fig S 098
572.27747
469.75516
391.71063
183.14877
455.22534
515.25616
155.08110
127.08627
420.22104
339.23856
86.09626
211.14375
254.14931
268.16479
325.18686
367.23306
685.36108
555.25104
667.35065
782.41522
839.43719
m/z

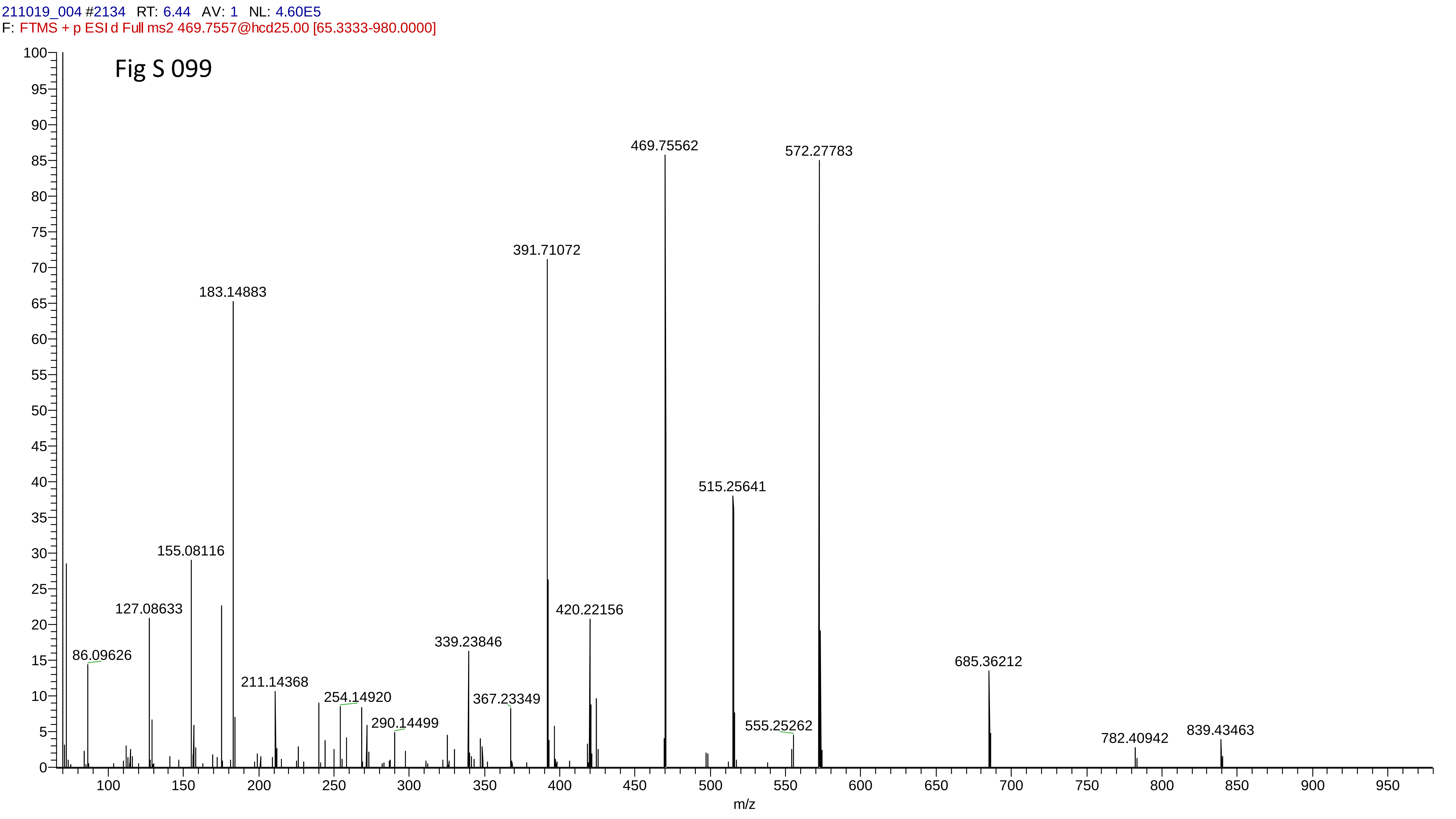
211019_004 #2134 RT: 6.44 AV: 1 NL: 4.60E5
F: FTMS + p ESI d Full ms2 469.7557@hcd25.00 [65.3333-980.0000]
Fig S 099
469.75562
572.27783
391.71072
183.14883
515.25641
155.08116
127.08633
420.22156
339.23846
86.09626
685.36212
211.14368
254.14920
367.23349
290.14499
555.25262
839.43463
782.40942
m/z

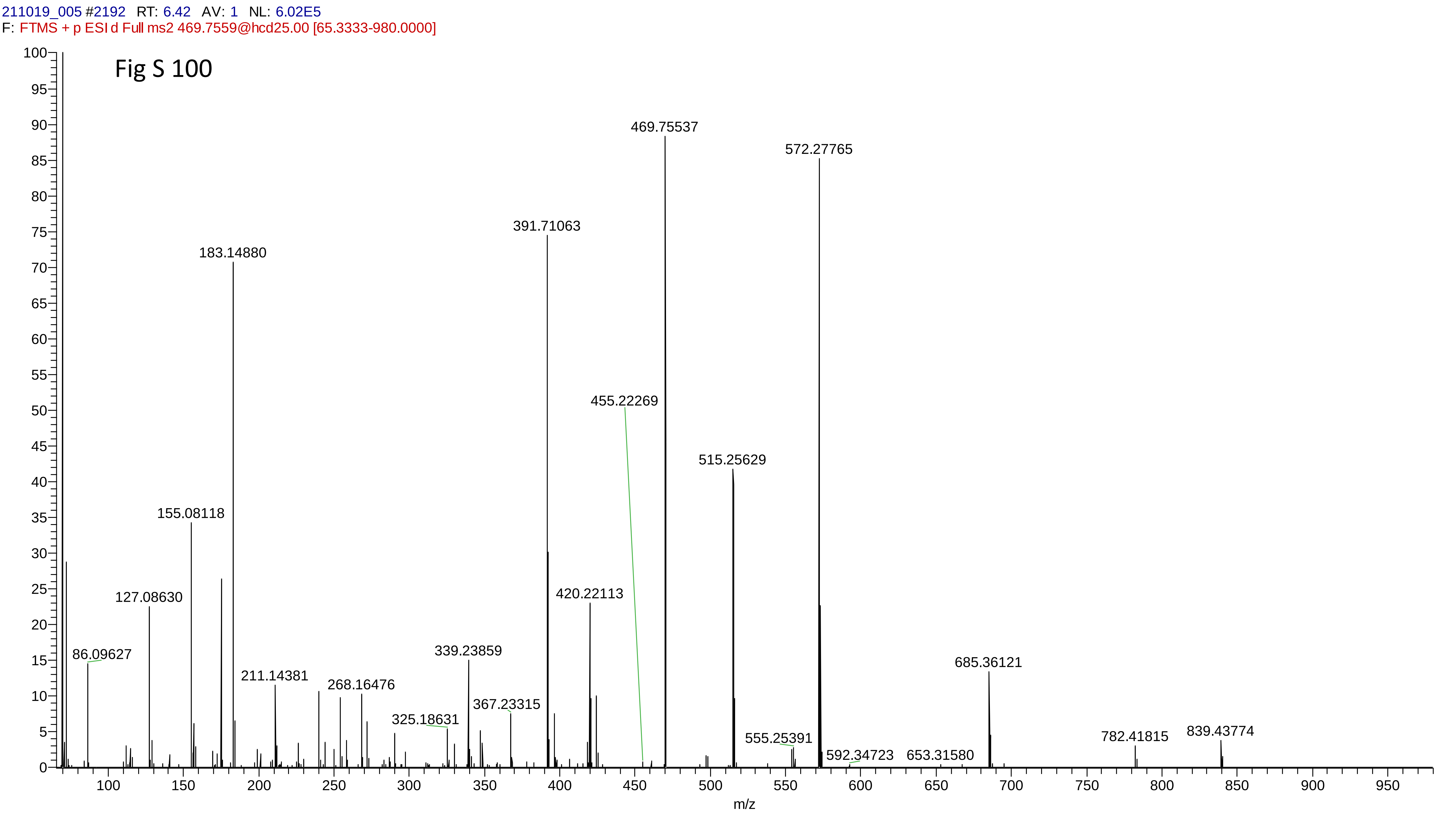
211019_005 #2192 RT: 6.42 AV: 1 NL: 6.02E5
F: FTMS + p ESI d Full ms2 469.7559@hcd25.00 [65.3333-980.0000]
Fig S 100
469.75537
572.27765
391.71063
183.14880
455.22269
515.25629
155.08118
127.08630
420.22113
86.09627
339.23859
685.36121
211.14381
268.16476
367.23315
325.18631
555.25391
782.41815
839.43774
592.34723
653.31580
m/z

S2 File. Chromatograms and MS/MS spectra.

Raw data chromatograms EGPVGF**p**GADGR

Fig S 101: chicken soup
Fig S 102: chicken broth A
Fig S 103: chicken broth B
Fig S 104: beef broth

Remarks:
-Extracted m/z range 587.775-587.785
-The retention time and m/z of the base peak are provided per peak and the provided intensity is of the highest peak.
-Data recorded in October 2021.

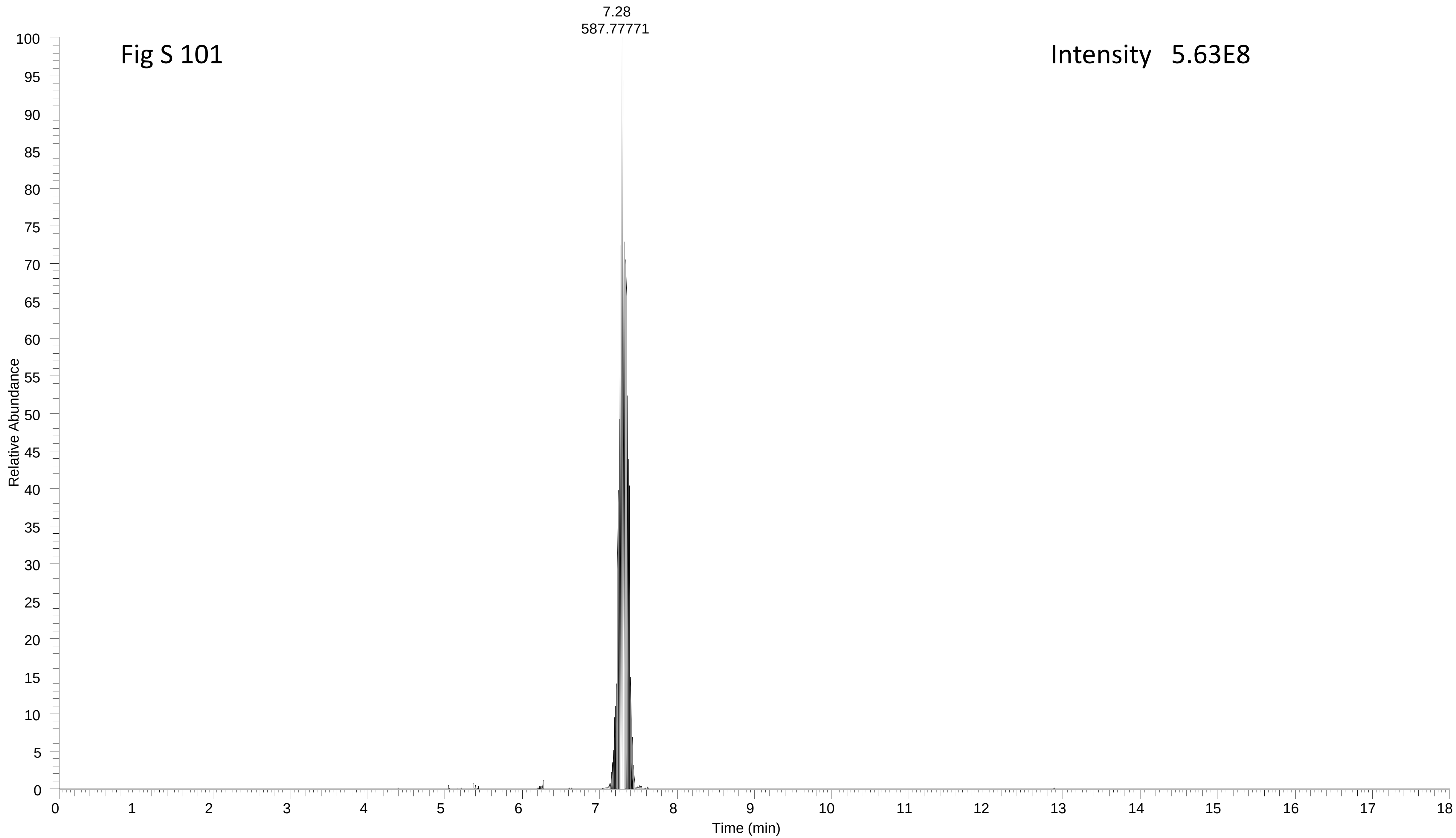
7.28
587.77771
Fig S 101
Intensity 5.63E8
Relative Abundance
Time (min)
0
5
10
15
20
25
30
35
40
45
50
55
60
65
70
75
80
85
90
95
100
0
1
2
3
4
5
6
7
8
9
10
11
12
13
14
15
16
17
18

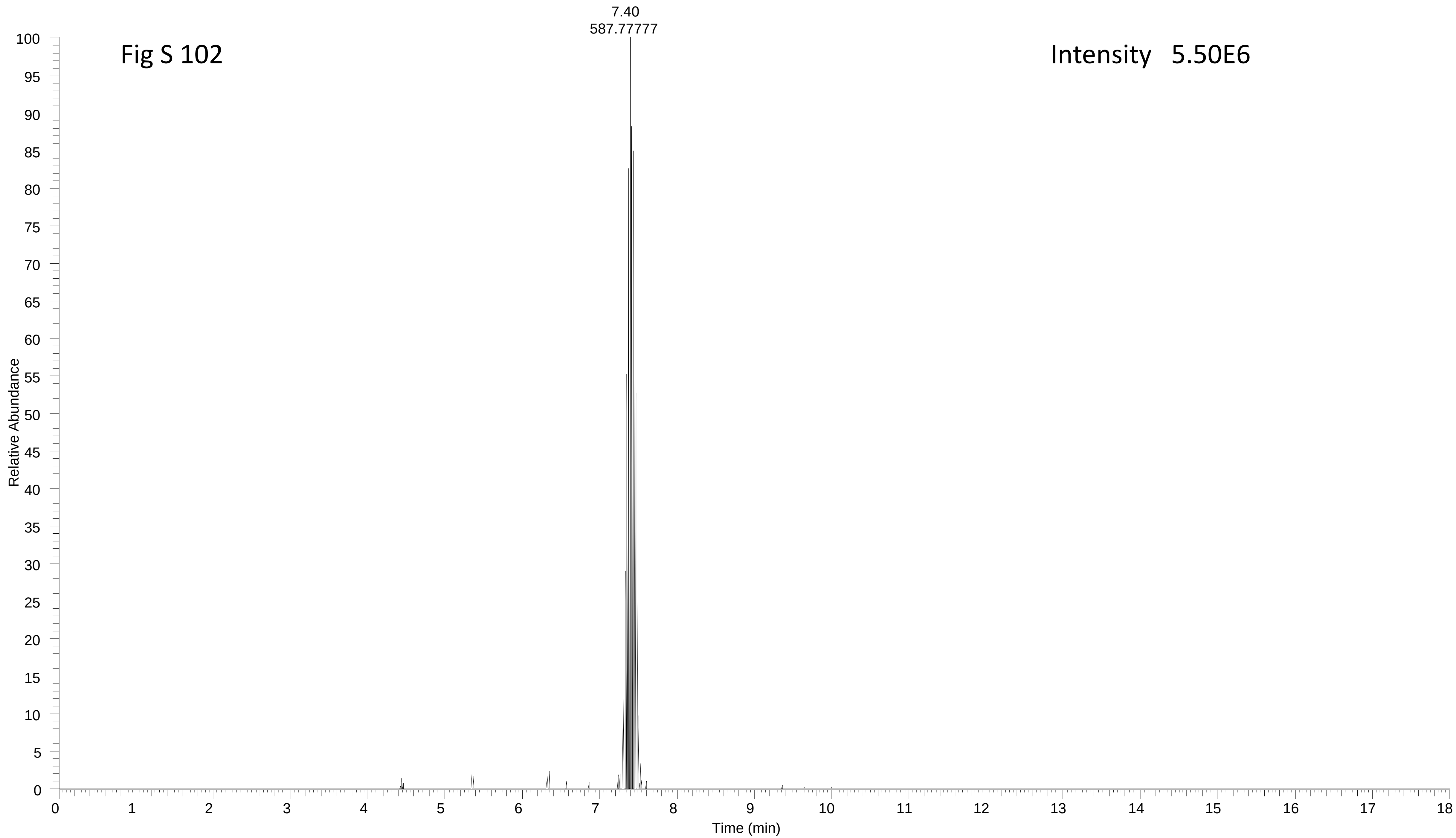
Fig S 102
Intensity 5.50E6
7.40
587.77777
Relative Abundance
Time (min)
0
5
10
15
20
25
30
35
40
45
50
55
60
65
70
75
80
85
90
95
100
1
2
3
4
5
6
7
8
9
10
11
12
13
14
15
16
17
18

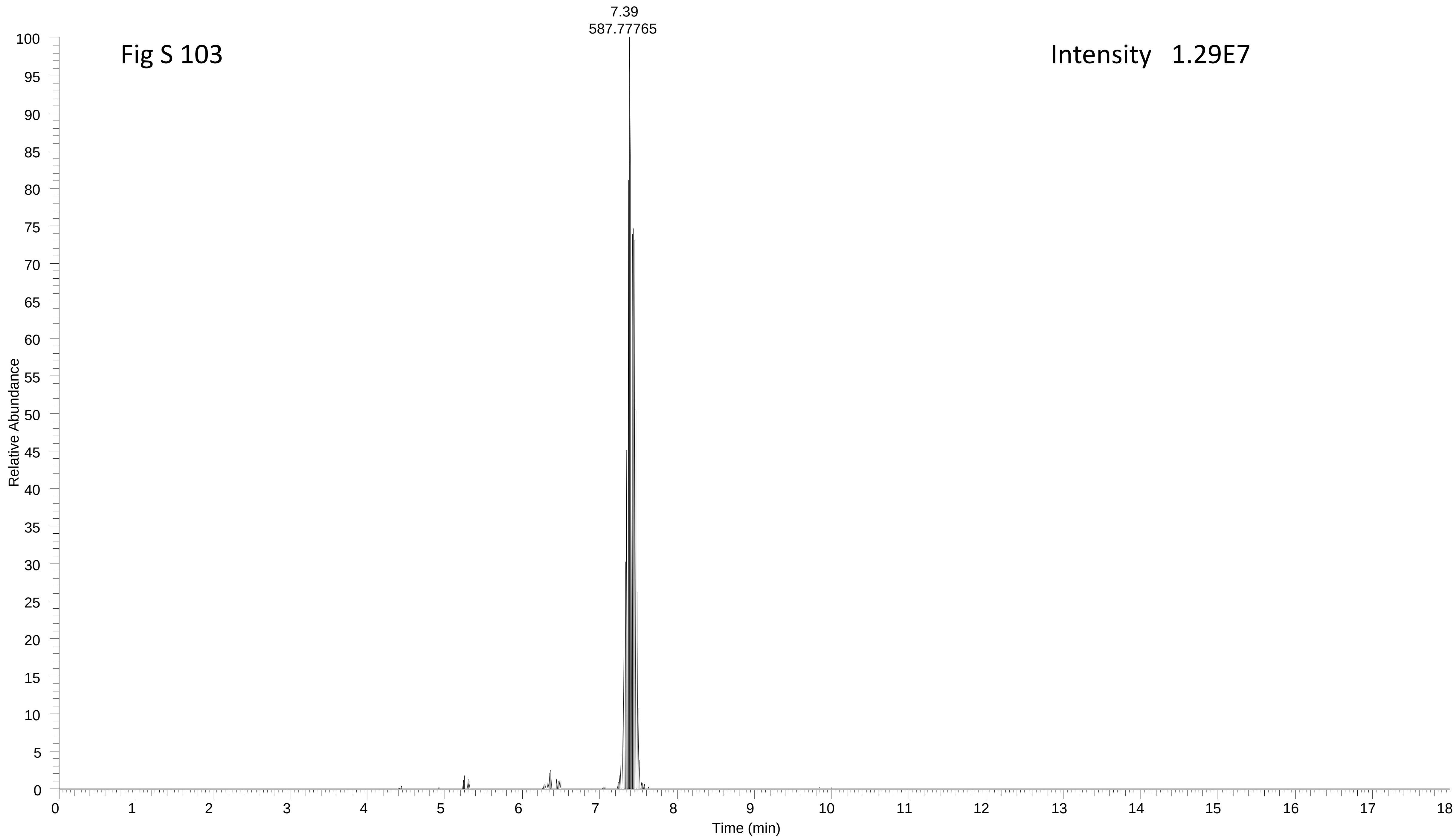
Fig S 103
Intensity 1.29E7
7.39
587.77765
Relative Abundance
Time (min)
0
5
10
15
20
25
30
35
40
45
50
55
60
65
70
75
80
85
90
95
100
0
1
2
3
4
5
6
7
8
9
10
11
12
13
14
15
16
17
18

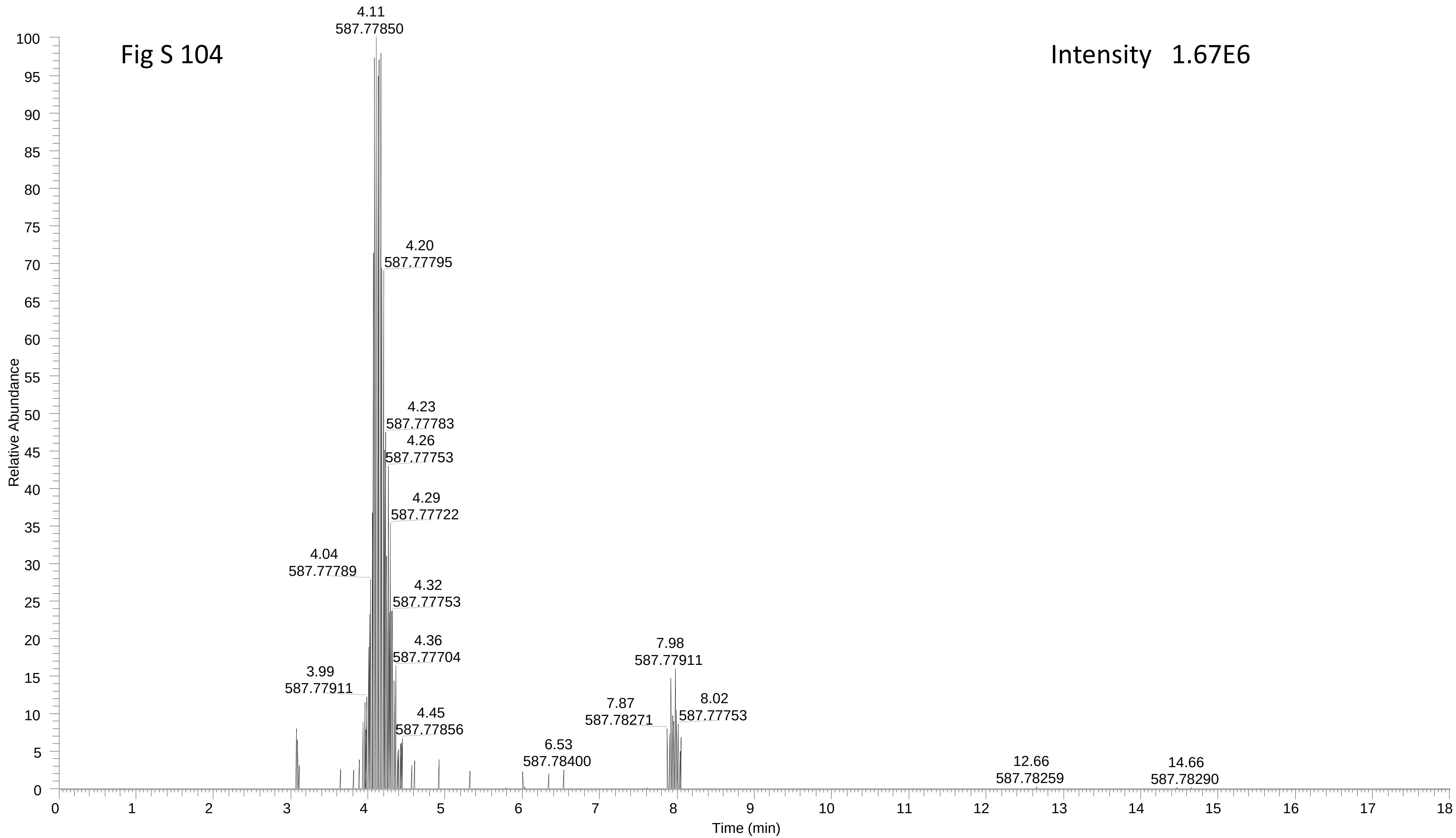
Fig S 104
Intensity 1.67E6
4.11
587.77850
4.20
587.77795
4.23
587.77783
4.26
587.77753
4.29
587.77722
4.04
587.77789
4.32
587.77753
4.36
587.77704
3.99
587.77911
4.45
587.77856
7.98
587.77911
7.87
587.78271
8.02
587.77753
6.53
587.78400
12.66
587.78259
14.66
587.78290
Relative Abundance
Time (min)
0
1
2
3
4
5
6
7
8
9
10
11
12
13
14
15
16
17
18
0
5
10
15
20
25
30
35
40
45
50
55
60
65
70
75
80
85
90
95
100

S2 File. Chromatograms and MS/MS spectra.

Raw data MS/MS spectra EGPVGF**p**GADGR

Fig S 105: chicken soup
Fig S 106: chicken broth A
Fig S 107: chicken broth B

Remarks:
-Precursor m/z ≈ 587.78
-Beef broth did not provide MS/MS spectra for this peptide.
-Data recorded in October 2021.
-See Fig 6 in the main document for peak annotation.

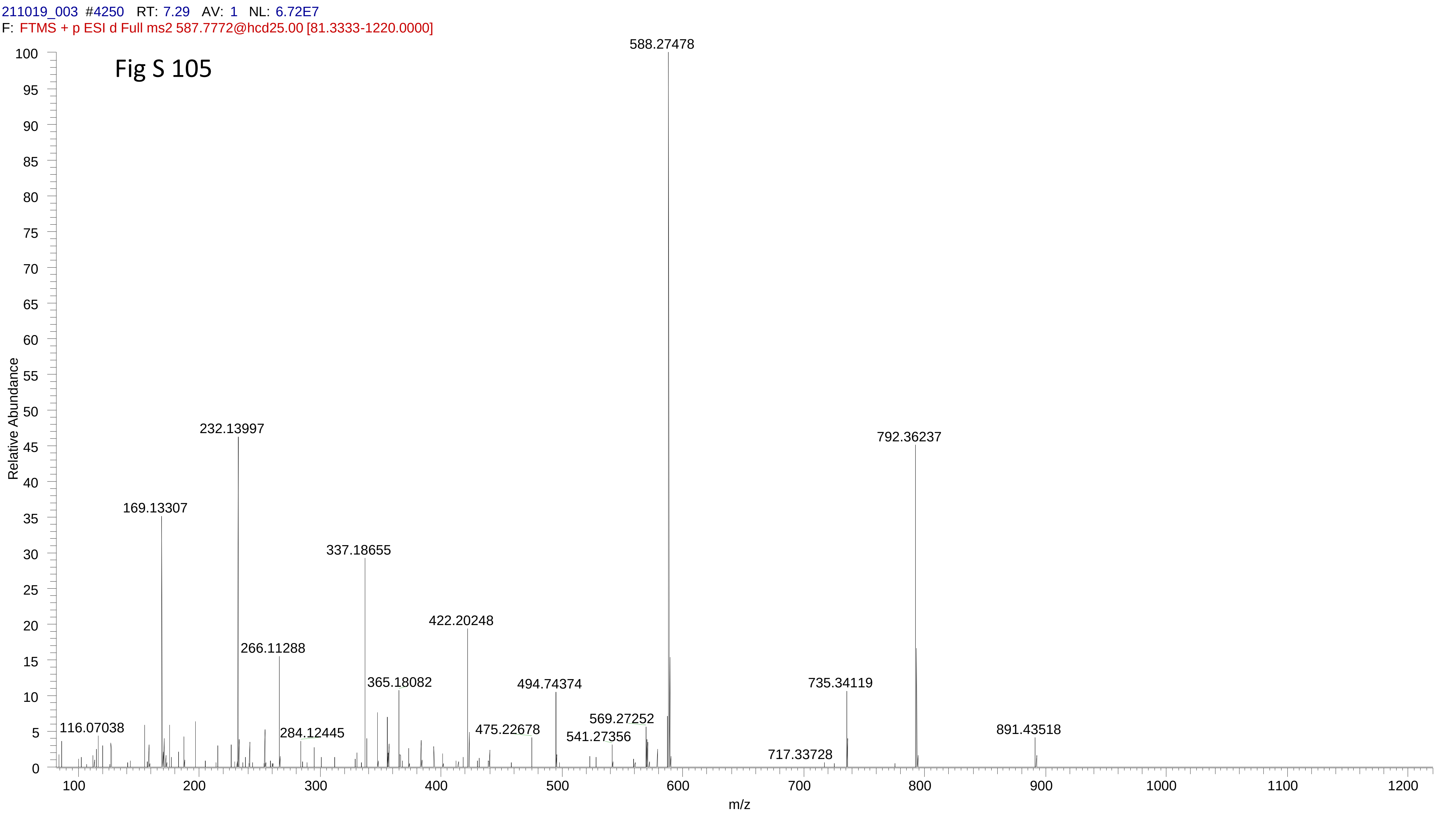
211019_003 #4250 RT: 7.29 AV: 1 NL: 6.72E7
F: FTMS + p ESI d Full ms2 587.7772@hcd25.00 [81.3333-1220.0000]
Fig S 105
588.27478
792.36237
232.13997
169.13307
337.18655
422.20248
266.11288
365.18082
494.74374
735.34119
569.27252
116.07038
284.12445
475.22678
541.27356
891.43518
717.33728
Relative Abundance
m/z

211019_004 #2427 RT: 7.34 AV: 1 NL: 2.55E5
F: FTMS + p ESI d Full ms2 587.7776@hcd25.00 [81.3333-1220.0000]

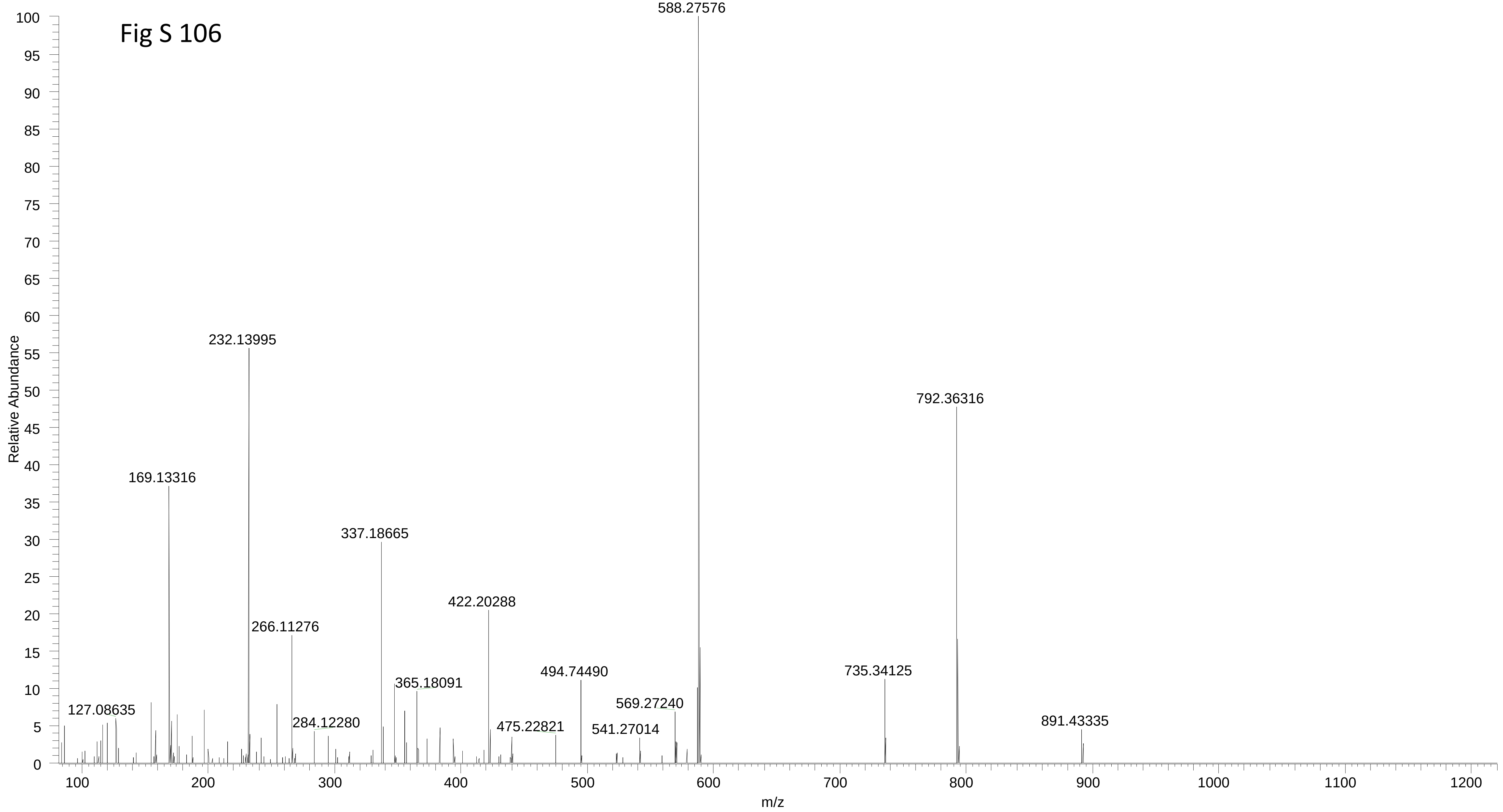

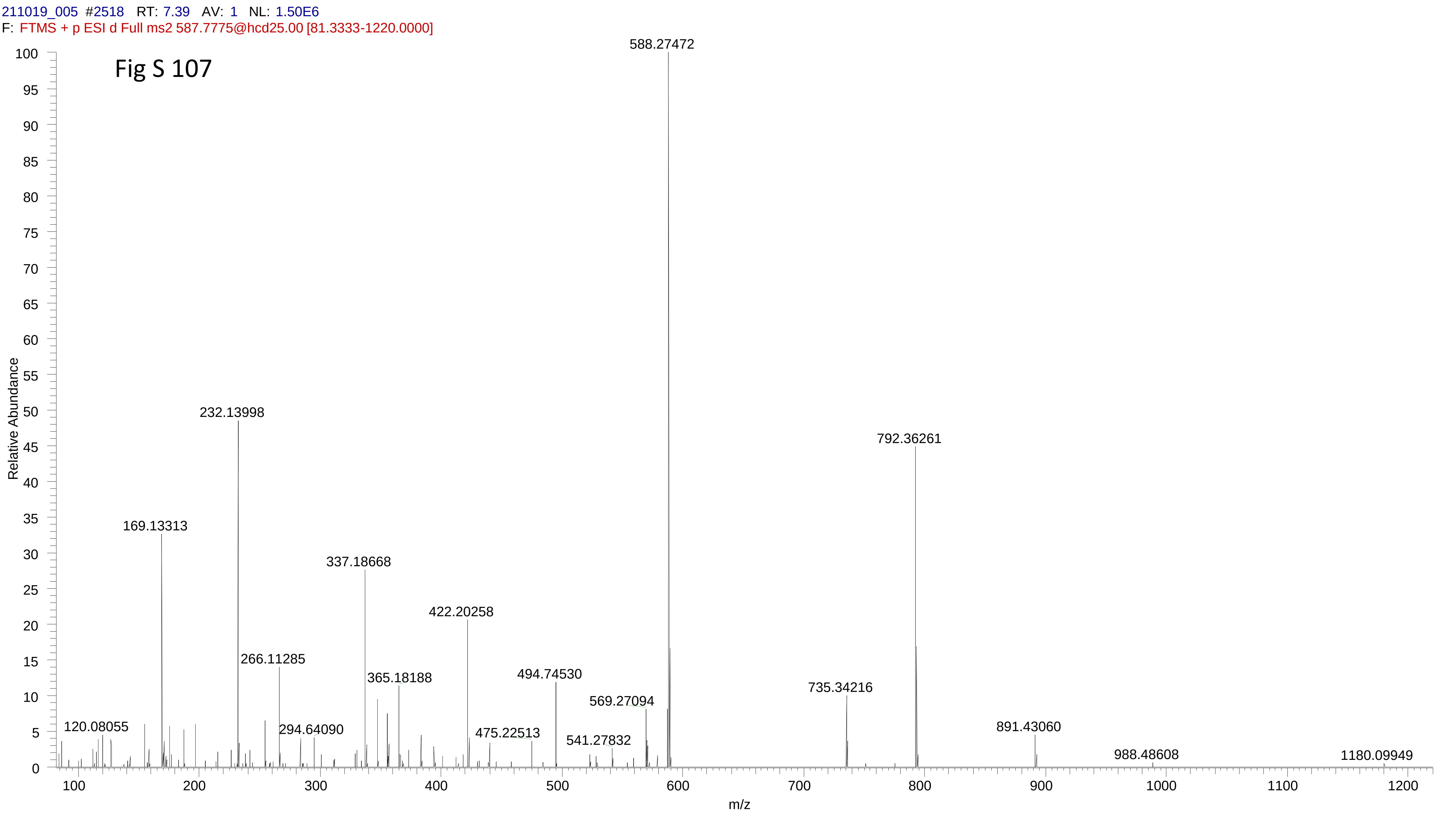

211019_005 #2518 RT: 7.39 AV: 1 NL: 1.50E6
F: FTMS + p ESI d Full ms2 587.7775@hcd25.00 [81.3333-1220.0000]
Fig S 107
Relative Abundance
m/z
588.27472
232.13998
792.36261
169.13313
337.18668
422.20258
266.11285
365.18188
494.74530
735.34216
569.27094
120.08055
294.64090
475.22513
541.27832
891.43060
988.48608
1180.09949

S2 File. Chromatograms and MS/MS spectra.

Raw data beef broth

Fig S 108: raw data chromatogram GETGPAGPAGPIGPVGAR
Fig S 109: raw data MS/MS spectrum GETGPAGPAGPIGPVGAR
Fig S 110: raw data chromatogram GI**p**GEFGL**p**GPAGAR
Fig S 111: raw data MS/MS spectrum GI**p**GEFGL**p**GPAGAR

Remarks:
-Fig S 108 extracted m/z range 780.905-780.915
-Fig S 110 extracted m/z range 714.365-714.375
-The retention time and m/z of the base peak are provided per peak and the provided intensity is of the highest peak.
-Data recorded in October 2021.

-Fig S 109 precursor m/z ≈ 780.91
-Fig S 110 precursor m/z ≈ 714.37
-Peak annotations were added to the spectra.
-GETGPAGPAGPIGPVGAR occurs in bovine collagen 1α1.
-GI**p**GEFGL**p**GPAGAR occurs in bovine collagen 1α2.

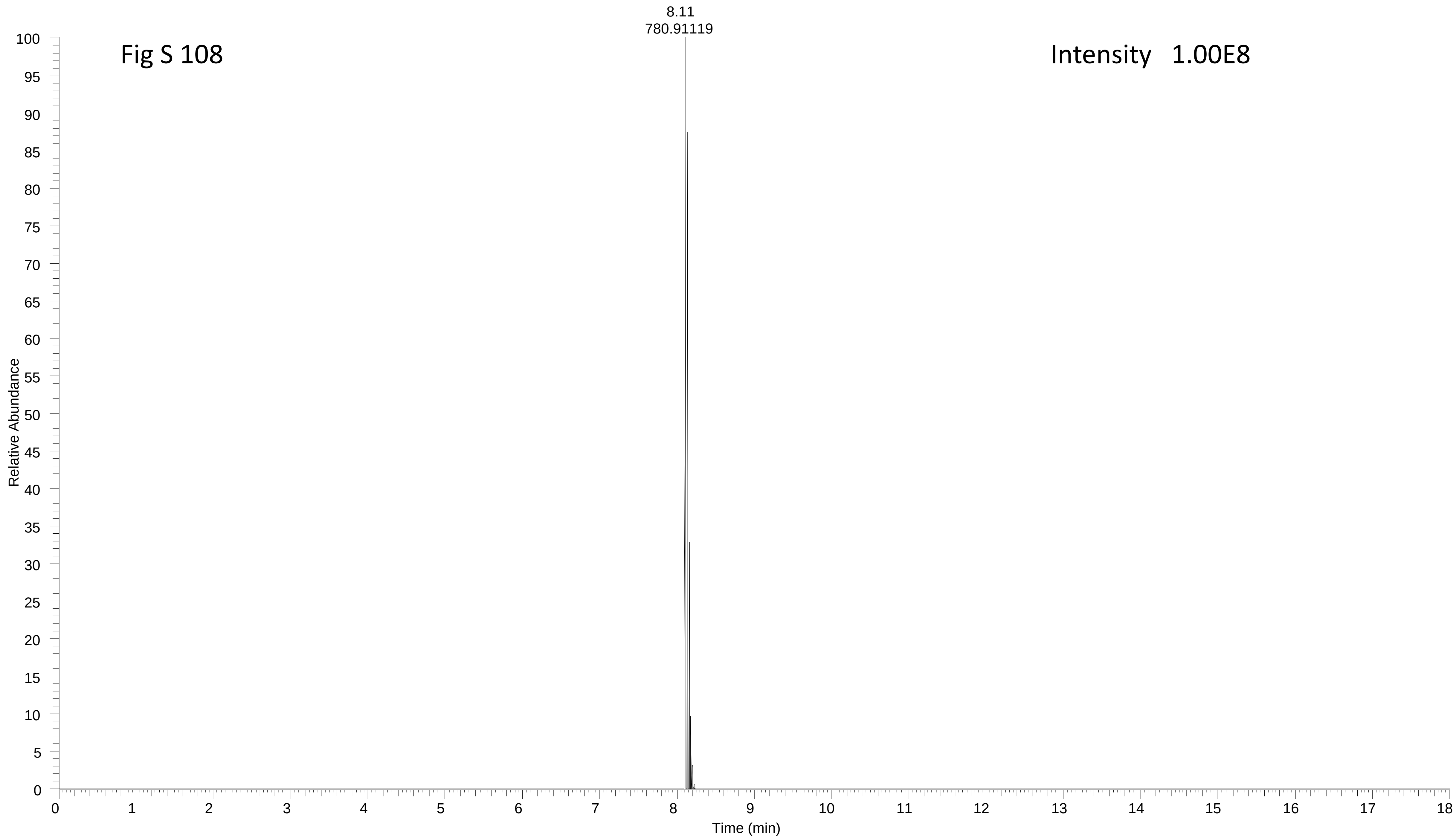

Fig S 108
Intensity 1.00E8
8.11
780.91119
Relative Abundance
Time (min)
0
5
10
15
20
25
30
35
40
45
50
55
60
65
70
75
80
85
90
95
100
1
2
3
4
6
7
8
9
11
12
13
14
16
17
18

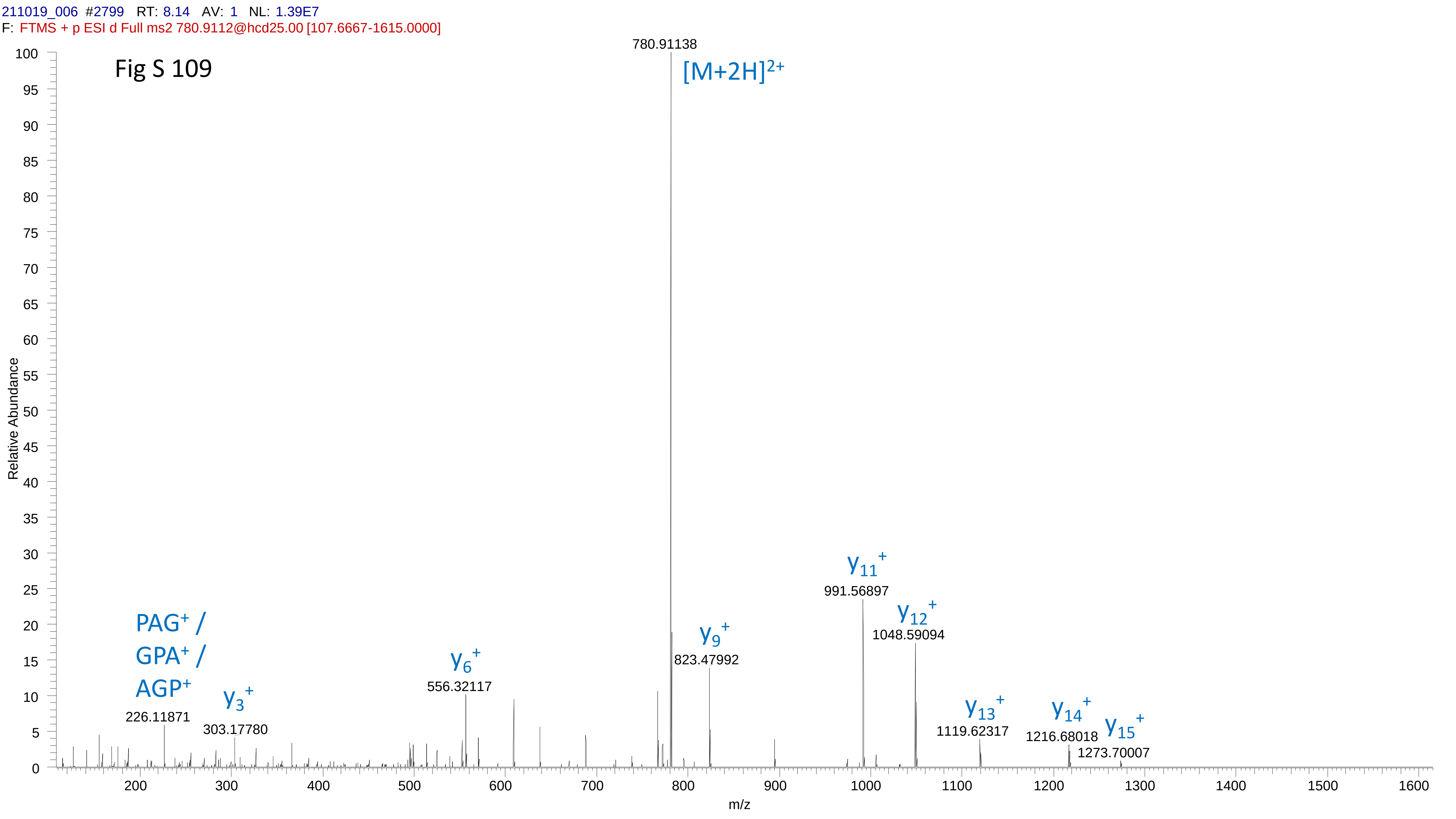
211019_006 #2799 RT: 8.14 AV: 1 NL: 1.39E7
F: FTMS + p ESI d Full ms2 780.9112@hcd25.00 [107.6667-1615.0000]
Fig S 109
780.91138
[M+2H]2+
PAG+ /
GPA+ /
AGP+
226.11871
y3+
303.17780
y6+
556.32117
y9+
823.47992
y11+
991.56897
y12+
1048.59094
y13+
1119.62317
y14+
1216.68018
y15+
1273.70007
Relative Abundance
m/z

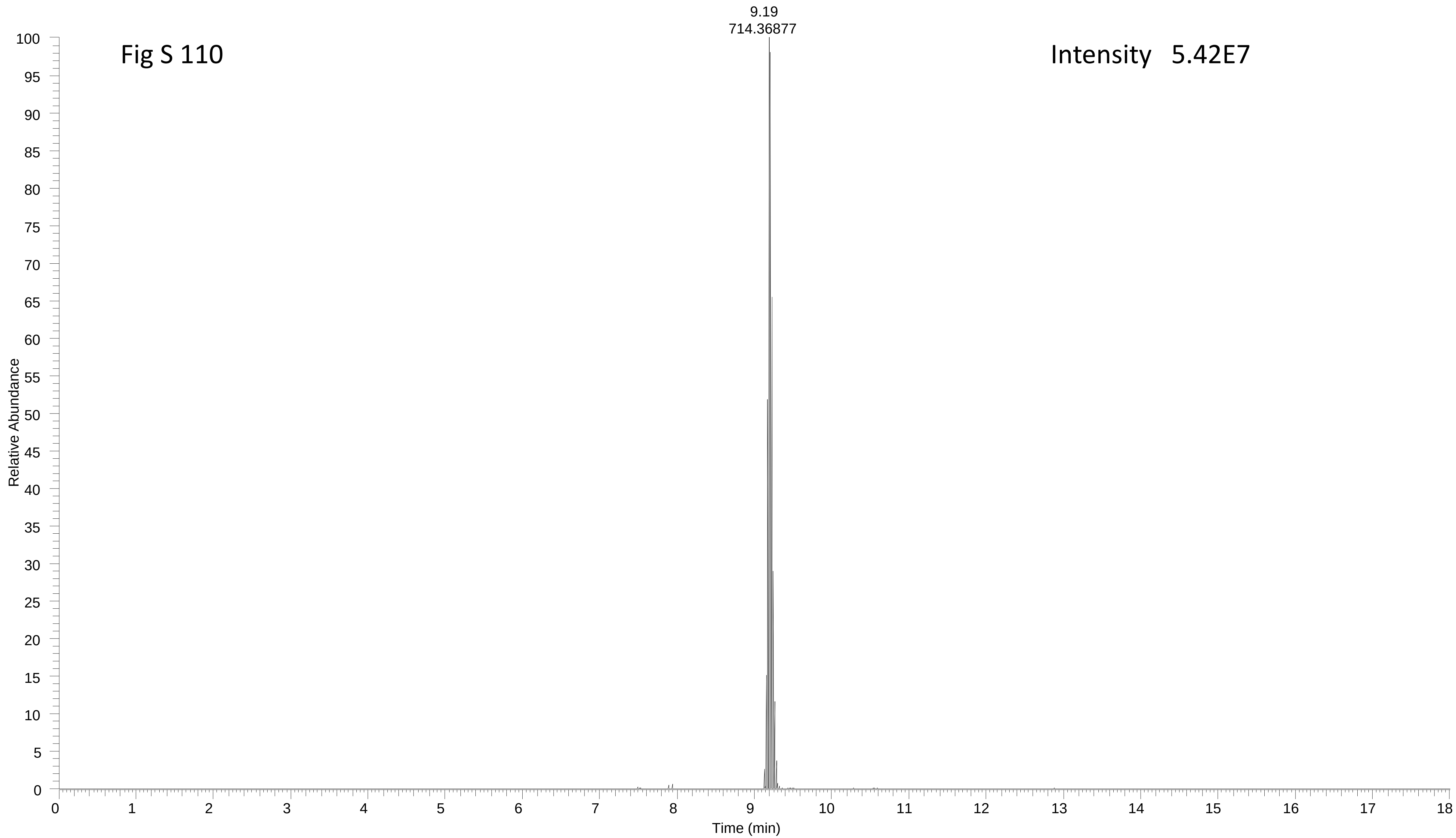
9.19
714.36877
Fig S 110
Intensity 5.42E7
Relative Abundance
Time (min)

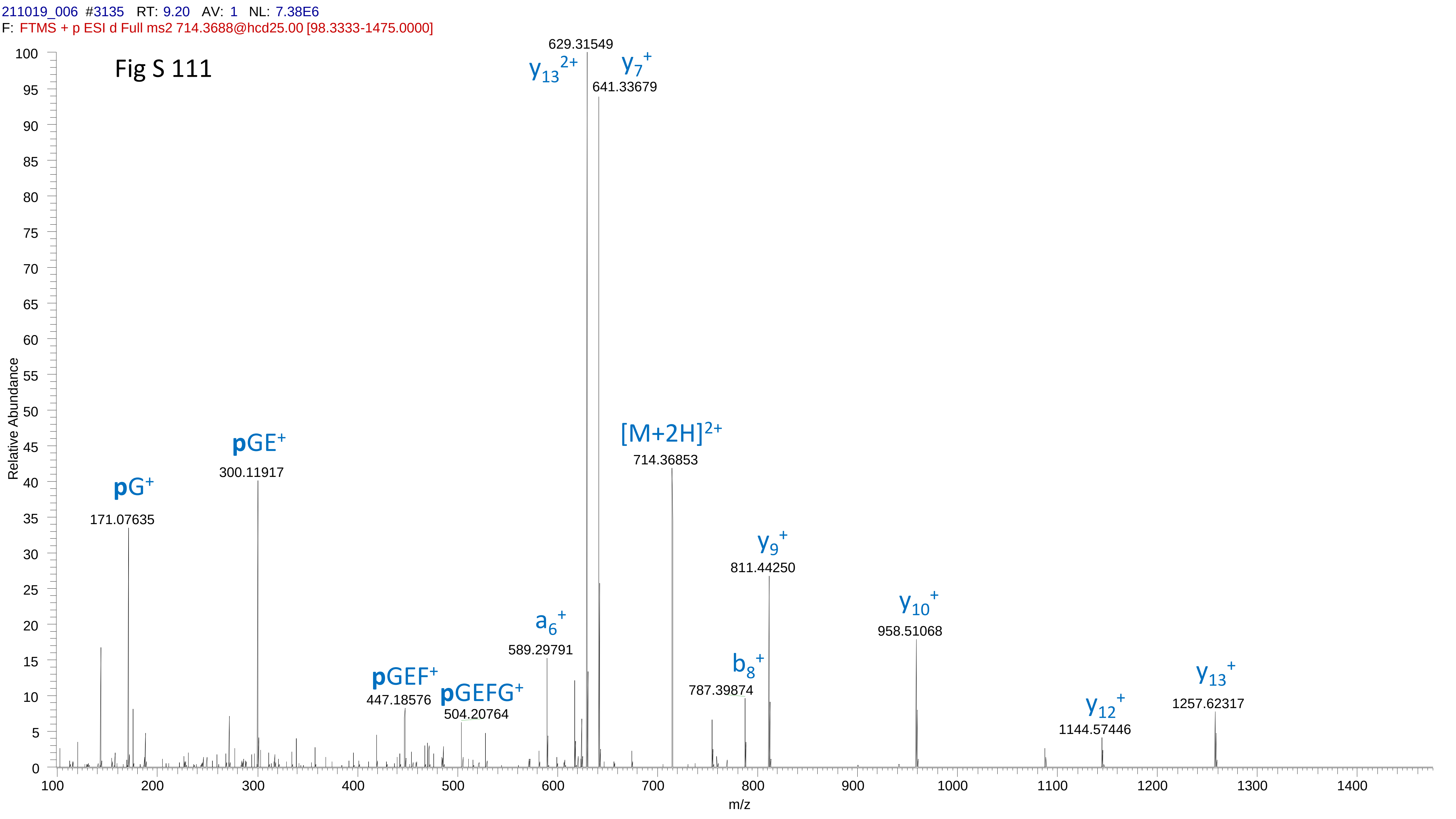
211019_006 #3135 RT: 9.20 AV: 1 NL: 7.38E6
F: FTMS + p ESI d Full ms2 714.3688@hcd25.00 [98.3333-1475.0000]
Fig S 111
629.31549
y13 2+
y7 +
641.33679
[M+2H]2+
714.36853
pGE+
300.11917
pG+
171.07635
y9 +
811.44250
y10 +
958.51068
a6 +
589.29791
b8 +
787.39874
pGEF+
447.18576
pGEFG+
504.20764
y13 +
1257.62317
y12 +
1144.57446
Relative Abundance
m/z
