# Supplementary material for: The quest for a generic bird target to detect the presence of bird in food products and considerations for paleoprotein analysis
Source: PLoS One. 2022 Dec 20;17(12):e0279369. doi: 10.1371/journal.pone.0279369 (PMC9767367; doi:10.1371/journal.pone.0279369)
Supplement: Attachment — Submitted filename: Response to reviewers.docx [file pone.0279369.s003.docx]

PONE-D-22-25317

The quest for a generic bird target to detect the presence of bird in food products and considerations for paleoprotein analysis

PLOS ONE

Dear Dr. Kleinnijenhuis,

Thank you for submitting your manuscript to PLOS ONE. After careful consideration, we feel that it has merit but does not fully meet PLOS ONE’s publication criteria as it currently stands. Therefore, we invite you to submit a revised version of the manuscript that addresses the points raised during the review process.

==============================

ACADEMIC EDITOR: Would you please go through the comments raised by the diligent reviewers and amend the MS accordingly. All corrections should be highlighted in red..

==============================

Please submit your revised manuscript by Nov 18 2022 11:59PM. If you will need more time than this to complete your revisions, please reply to this message or contact the journal office at plosone@plos.org. When you're ready to submit your revision, log on to https://www.editorialmanager.com/pone/ and select the 'Submissions Needing Revision' folder to locate your manuscript file.

Please include the following items when submitting your revised manuscript:

•	A rebuttal letter that responds to each point raised by the academic editor and reviewer(s). You should upload this letter as a separate file labeled 'Response to Reviewers'.

•	A marked-up copy of your manuscript that highlights changes made to the original version. You should upload this as a separate file labeled 'Revised Manuscript with Track Changes'.

•	An unmarked version of your revised paper without tracked changes. You should upload this as a separate file labeled 'Manuscript'.

If you would like to make changes to your financial disclosure, please include your updated statement in your cover letter. Guidelines for resubmitting your figure files are available below the reviewer comments at the end of this letter.

If applicable, we recommend that you deposit your laboratory protocols in protocols.io to enhance the reproducibility of your results. Protocols.io assigns your protocol its own identifier (DOI) so that it can be cited independently in the future. For instructions see: https://journals.plos.org/plosone/s/submission-guidelines#loc-laboratory-protocols. Additionally, PLOS ONE offers an option for publishing peer-reviewed Lab Protocol articles, which describe protocols hosted on protocols.io. Read more information on sharing protocols at https://plos.org/protocols?utm_medium=editorial-email&utm_source=authorletters&utm_campaign=protocols.

We look forward to receiving your revised manuscript.

Kind regards,

A. M. Abd El-Aty

Academic Editor

PLOS ONE

**Reply E.0:** The authors want to thank the Editor for arranging the review of our manuscript. Our replies to your comments are provided in green text. After each reply we indicated the line numbers when changes were made to the manuscript. The line numbers correspond to the revised manuscript file with track changes.

Journal Requirements:

When submitting your revision, we need you to address these additional requirements.

1. Please ensure that your manuscript meets PLOS ONE's style requirements, including those for file naming. The PLOS ONE style templates can be found at

https://journals.plos.org/plosone/s/file?id=wjVg/PLOSOne_formatting_sample_main_body.pdf and

<https://journals.plos.org/plosone/s/file?id=ba62/PLOSOne_formatting_sample_title_authors_affiliations.pdf>

**Reply E.1:** Several changes were made to the manuscript to meet the additional PLOS ONE requirements.

Several changes throughout manuscript.

2. Thank you for stating the following financial disclosure:

"The authors received no specific funding for this work. The work was financed by Triskelion. No external funding was used."

At this time, please address the following queries:

a) Please clarify the sources of funding (financial or material support) for your study. List the grants or organizations that supported your study, including funding received from your institution.

b) State what role the funders took in the study. If the funders had no role in your study, please state: “The funders had no role in study design, data collection and analysis, decision to publish, or preparation of the manuscript.”

c) If any authors received a salary from any of your funders, please state which authors and which funders.

d) If you did not receive any funding for this study, please state: “The authors received no specific funding for this work.”

Please include your amended statements within your cover letter; we will change the online submission form on your behalf.

**Reply E.2:** The funding statement is changed to “The authors received no specific funding for this work.”

Change in lines 488-489.

3. In your Data Availability statement, you have not specified where the minimal data set underlying the results described in your manuscript can be found. PLOS defines a study's minimal data set as the underlying data used to reach the conclusions drawn in the manuscript and any additional data required to replicate the reported study findings in their entirety. All PLOS journals require that the minimal data set be made fully available. For more information about our data policy, please see http://journals.plos.org/plosone/s/data-availability.

"Upon re-submitting your revised manuscript, please upload your study’s minimal underlying data set as either Supporting Information files or to a stable, public repository and include the relevant URLs, DOIs, or accession numbers within your revised cover letter. For a list of acceptable repositories, please see http://journals.plos.org/plosone/s/data-availability#loc-recommended-repositories. Any potentially identifying patient information must be fully anonymized.

Important: If there are ethical or legal restrictions to sharing your data publicly, please explain these restrictions in detail. Please see our guidelines for more information on what we consider unacceptable restrictions to publicly sharing data: http://journals.plos.org/plosone/s/data-availability#loc-unacceptable-data-access-restrictions. Note that it is not acceptable for the authors to be the sole named individuals responsible for ensuring data access.

We will update your Data Availability statement to reflect the information you provide in your cover letter.

**Reply E.3:** The authors prefer to make the requested additional data available as supporting information. Calculations and data regarding the generic targets, the common bird ancestor and the distance table have been added in the file “S1 file.xlsx” in 3 sheets. Relevant chromatograms and MS/MS spectra have been added in “S2 file.pdf” Fig S 001 to Fig S 111.

Change by addition of supplementary files.

[Note: HTML markup is below. Please do not edit.]

Reviewers' comments:

Reviewer's Responses to Questions

Comments to the Author

1. Is the manuscript technically sound, and do the data support the conclusions?

The manuscript must describe a technically sound piece of scientific research with data that supports the conclusions. Experiments must have been conducted rigorously, with appropriate controls, replication, and sample sizes. The conclusions must be drawn appropriately based on the data presented.

Reviewer #1: Yes

**Reply 1.1:** The authors want to thank reviewer 1 for providing comments that were very useful for improving the manuscript. Our replies to your comments are provided in green text. After each reply we indicated the line numbers when changes were made to the manuscript. The line numbers correspond to the revised manuscript file with track changes.

No changes were made based on this comment.

Reviewer #2: Partly

**Reply 2.1:** The authors want to thank reviewer 2 for providing comments that were very useful for improving the manuscript. Our replies to your comments are provided in green text. After each reply we indicated the line numbers when changes were made to the manuscript. The line numbers correspond to the revised manuscript file with track changes. The authors made the requested additional data available as supporting information. Calculations and data regarding the generic targets, the common bird ancestor and the distance table have been added in the file “S1 file.xlsx” in 3 sheets. Relevant chromatograms and MS/MS spectra have been added in “S2 file.pdf” Fig S 001 to Fig S 111.

Change by addition of supplementary files.

________________________________________

2. Has the statistical analysis been performed appropriately and rigorously?

Reviewer #1: Yes

**Reply 1.2:** Ok

No changes were made based on this comment.

Reviewer #2: N/A

**Reply 2.2:** Ok.

No changes were made based on this comment.

________________________________________

3. Have the authors made all data underlying the findings in their manuscript fully available?

The PLOS Data policy requires authors to make all data underlying the findings described in their manuscript fully available without restriction, with rare exception (please refer to the Data Availability Statement in the manuscript PDF file). The data should be provided as part of the manuscript or its supporting information, or deposited to a public repository. For example, in addition to summary statistics, the data points behind means, medians and variance measures should be available. If there are restrictions on publicly sharing data—e.g. participant privacy or use of data from a third party—those must be specified.

Reviewer #1: Yes

**Reply 1.3:** Ok. However, the authors made additional data available as supporting information. Calculations and data regarding the generic targets, the common bird ancestor and the distance table have been added in the file “S1 file.xlsx” in 3 sheets. Relevant chromatograms and MS/MS spectra have been added in “S2 file.pdf” Fig S 001 to Fig S 111.

Change by addition of supplementary files.

Reviewer #2: No

**Reply 2.3:** As stated in a previous reply, the authors made the requested additional data available as supporting information. Calculations and data regarding the generic targets, the common bird ancestor and the distance table have been added in the file “S1 file.xlsx” in 3 sheets. Relevant chromatograms and MS/MS spectra have been added in “S2 file.pdf” Fig S 001 to Fig S 111.

Change by addition of supplementary files.

________________________________________

4. Is the manuscript presented in an intelligible fashion and written in standard English?

PLOS ONE does not copyedit accepted manuscripts, so the language in submitted articles must be clear, correct, and unambiguous. Any typographical or grammatical errors should be corrected at revision, so please note any specific errors here.

Reviewer #1: Yes

**Reply 1.4:** Ok

No changes were made based on this comment.

Reviewer #2: No

**Reply 2.4:** Throughout the text several changes were applied to make the language more clear, correct and unambiguous.

Several changes throughout manuscript.

________________________________________

5. Review Comments to the Author

Please use the space provided to explain your answers to the questions above. You may also include additional comments for the author, including concerns about dual publication, research ethics, or publication ethics. (Please upload your review as an attachment if it exceeds 20,000 characters)

Reviewer #1: The article needs some revision to be suitable for publication

-The abstract should be informative and include the main findings.

**Reply 1.5.1:** The abstract was rephrased to make it more informative and better reflect the main findings.

Changes in lines 25-45.

-Language should be revised.

**Reply 1.5.2:** Throughout the text several changes were applied to make the language more clear, correct and unambiguous.

Several changes throughout manuscript.

-Figures should be represented in higher resolution

**Reply 1.5.3:** The authors wonder whether the individual uploaded figure files have been reviewed. We uploaded the figures in sufficiently high resolution, but unfortunately the resolution was much lower in the built pdf. The resolution of Fig 3 was however increased (as it was on the low side) and all figure files were uploaded as .tif files.

The resolution of Fig 3 was increased.

- The introduction should be enriched with recent references (2016-2022)

**Reply 1.5.4:** The work described in the manuscript mainly builds on older scientific work and our own recent work. We increased the number of references by approximately 13% with recent references to enrich the introduction.

Changes in lines 67, 79, 92, 95 and 554-566.

Reviewer #2: The manuscript “The quest for a generic bird target to detect the presence of bird in food products and considerations for paleoprotein analysis” by Kleinnijenhuis and van Holthoon is proposed as a typical untargeted approach for “protein biomarkers” identification.

However, with respect to the previous published papers (DOI: 10.1016/j.foodchem.2017.09.104 and 10.1016/j.fochx.2022.100333) some crucial steps are missing. In a classical untargeted approach, the “in silico” analysis is followed by an experimental validation of the candidate biomarkers. Here, the protein identification following tryptic digestion is missing. The authors suggest Xcalibur as software for data analysis even though it is only a software for raw data visualization. Some major points need to be addressed by the authors to improve the manuscript quality:

-The authors must perform a protein identification via Proteome Discover as in the already published papers by using a custom database. This step is crucial also to check the efficacy of the collagen extraction process;

**Reply 2.5.1:** During this study the analysis of the LC-MS/MS data was performed manually to ensure a high quality of the analysis. Therefore Proteome Discoverer was not used. We added the sentence “Data analysis was performed manually.” In addition, the experimental detection of the generic peptides was preceded by a theoretical prediction using the full collagen sequences of 83 bird species. Also the uniqueness of the target sequences was investigated using protein blast. The authors do not see added value in performing a Proteome Discoverer analysis, given the analyses that were performed. As the identification of bird in food products is qualitative, it will not be affected by the extraction efficiency. In supplementary S2 File the intensity of the chromatographic peaks was added to give an indication of how much target peptide was detected.

Change in line 164 + addition of S2 File.

-The authors clarify how they placed several grams per product in milliQ water for 2 days at 100°C and why they didn’t perform reduction/alkylation;

**Reply 2.5.2:** We clarified by changing the sentence to: “Collagen from these birds was extracted by placing several grams per product in milliQ water in an oven at 100°C for 2 days.” We did not perform reduction and alkylation in this study, because it is not strictly necessary as the collagen GXY domain, from which the peptide targets of interest are cleaved, does not contain disulfide bridges. Sentence changed to: “Aliquots were digested with trypsin without reduction or alkylation, because the collagen GXY domain, from which the peptide targets of interest were cleaved, does not contain disulfide bridges.”

Changes in lines 140 and 143-144.

-MS/MS fragmentation spectra captions showing the precursor m/z are missing. Did you manually assign the sequence?

**Reply 2.5.3:** Yes, the sequences were manually assigned. In the legends of Figures 4 and 6 the precursor m/z values were added.

Changes in lines 328, 329 and 360.

-A very careful reading of the manuscript and a review of the English language is recommended.

**Reply 2.5.4:** Throughout the text several changes were applied to make the language more clear, correct and unambiguous.

Several changes throughout manuscript.

________________________________________

6. PLOS authors have the option to publish the peer review history of their article (what does this mean?). If published, this will include your full peer review and any attached files.

If you choose “no”, your identity will remain anonymous but your review may still be made public.

Do you want your identity to be public for this peer review? For information about this choice, including consent withdrawal, please see our Privacy Policy.

Reviewer #1: No

Reviewer #2: No

________________________________________
